# Synthesis of complex bicyclic scaffolds by intermolecular photosensitized dearomative cycloadditions of activated alkenes and naphthalenes†

**DOI:** 10.1039/d2sc04789f

**Published:** 2022-10-14

**Authors:** Wang Wang, Yanyao Cai, Renyu Guo, M. Kevin Brown

**Affiliations:** Department of Chemistry, Indiana University 800 E. Kirkwood Ave Bloomington IN 47405 USA brownmkb@indiana.edu

## Abstract

The rapid buildup of molecular complexity from simple precursors is a key goal in organic chemistry. One strategy to achieve this is through a dearomative cycloaddition wherein a 2D arene and alkene is converted to a 3D structure. In many cases this type of reactivity has been achieved with photochemistry. Despite the prospect of such a reaction, most known variants are intramolecular, which greatly limits the scope of chemical space that can be accessed. Intermolecular variants are known but are generally limited to heterocyclic systems such as indoles or quinolines. Herein, a method for intermolecular dearomative cycloaddition of simple naphthalenes with alkenes is presented. The reactions operate by a photoinduced sensitization of the arene. The bridged bicyclic products are generated with control of regiochemistry and function for a range of alkenes. In addition, the products can serve as useful intermediates as demonstrated in the synthesis of a biologically active benzazapine analog. Mechanistic studies are also included, which support reaction *via* a triplet excited state and that the selectivity can be rationalized by spin-density calculations.

## Abstract 2

An intermolecular dearomative cycloaddition of activated alkenes and naphthalenes is presented. The reaction provides access to complex 3D scaffolds from simple 2D arenes.

## Graphical abstract

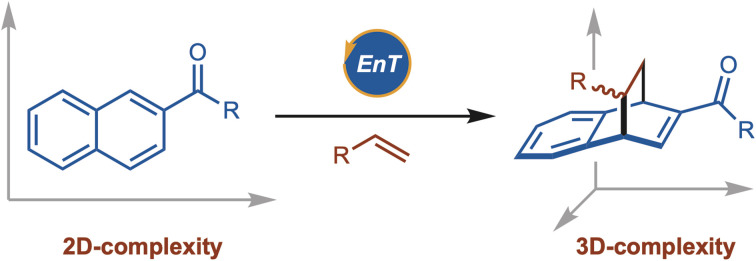

## Introduction

**Scheme 1:**
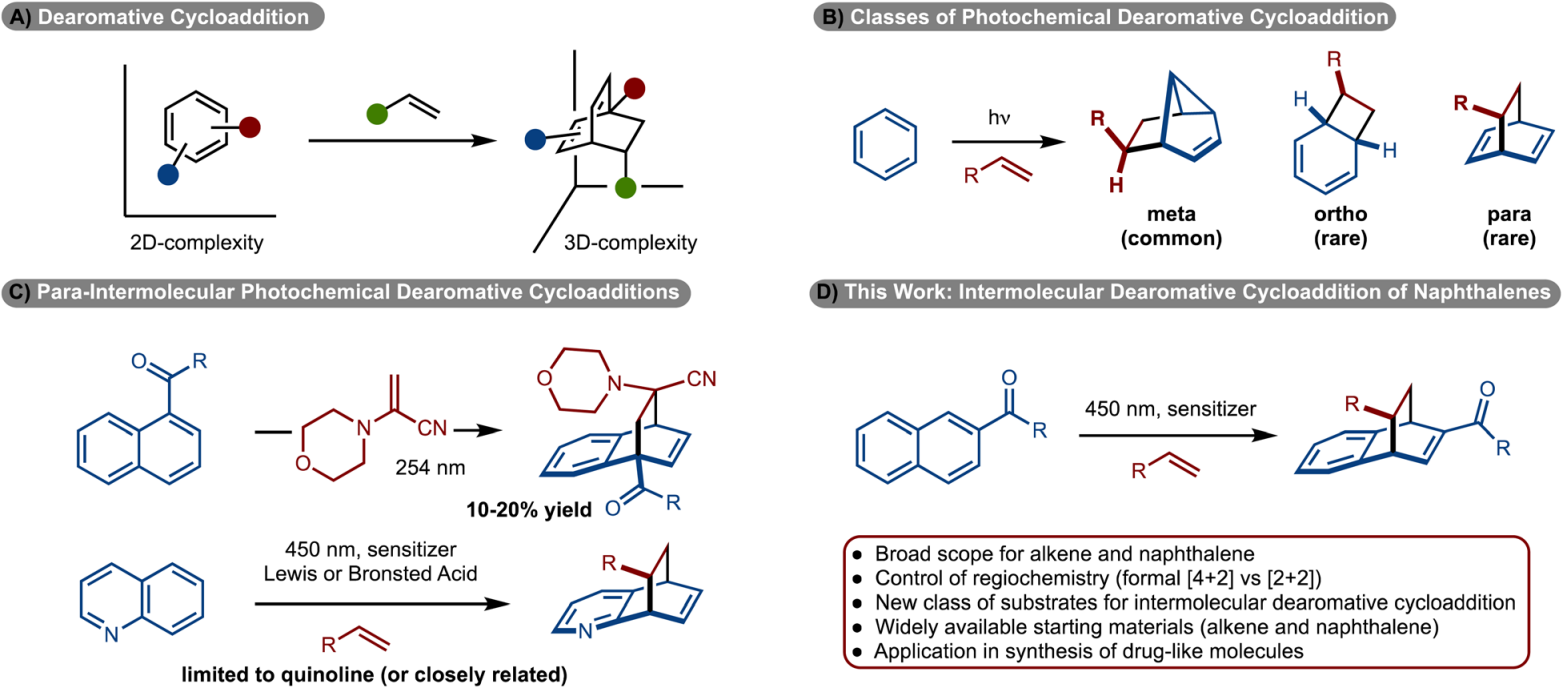
Dearomative cycloadditions.

Cycloadditions are one of the most significant classes of reactions in chemical synthesis because simple π-systems can be converted to complex rings in a single step.^1^ The variation of such methods is vast. Significant effort has been directed towards development of [4 + 2], [3 + 2] and [2 + 2]-cycloadditions.^1^ Despite these efforts, engaging arenes in cycloaddition reactions is challenging due to overcoming the energy of aromaticity.^2,3^ However, development of these reactions is important, as widely available arenes can be used to generate complex, 3D-rich scaffolds that are of general high value in medicinal chemistry (Scheme 1A).^4–6^

While dearomative cycloaddition has been achieved in several contexts,^7^ the reactions are generally endergonic and thus thermally induced processes are not feasible. An emerging strategy to overcome the energy of aromaticity involves photochemical cycloadditions.^8–10^ Since the reaction operates, in part, on an excited state energy surface, photochemically induced retro-cycloaddition is generally not observed because the photophysical properties of the product have changed relative to the starting materials. Most commonly, an arene is irradiated with UV light, which allows access to either the excited singlet or triplet state. Generally, with direct irradiation of arenes the excited singlet state is generated, which upon reaction with an alkene results in formation of a *meta*-cycloadduct (Scheme 1B).^11–13^ Accordingly, this area of research has been extensively investigated. Reactions to prepare either *ortho*-, or *para*-cycloadducts, generally require reaction *via* the excited triplet state.^11–13^ Despite the possibility of such a reaction, few examples have been reported and most are limited to intramolecular variants.^8–10,14–23^ Development of the more challenging intermolecular cycloadditions has seen limited development. With respect to *ortho*-cycloadditions, Bach reported the enantioselective dearomative cycloaddition of phenanthrenes.^24^

More recently, *ortho*-cycloaddition of indoles,^26–28^ benzofuran,^25^ and benzothiophene^25^ have also been developed. Only two examples are known for *para*-cycloadditions between alkenes and arenes. In 1985 Döpp disclosed reaction of 1-acyl napthalenes with 1-morpholinoacrylonitrile to generate the products, albeit in only ∼10–20% yields (Scheme 1C).^29^ Recently our lab, in collaboration with the Glorius and Houk groups, and subsequently Kano, disclosed an intermolecular dearomative cycloaddition between alkenes and quinolines (Scheme 1C).^30,31^ While these studies represent significant steps forward, the reactions require the use of specific heterocyclic rings. Despite these advances, the photosensitized intermolecular cycloaddition of arenes with alkenes remains rare and underdeveloped. In this manuscript, a new class of substrates for intermolecular cycloaddition of arenes with alkenes is disclosed (Scheme 1D). In particular, the use of a pyrazole substituted naphthyl group allowed for the synthesis of diverse products.

## Results and discussion

**Scheme 2:**
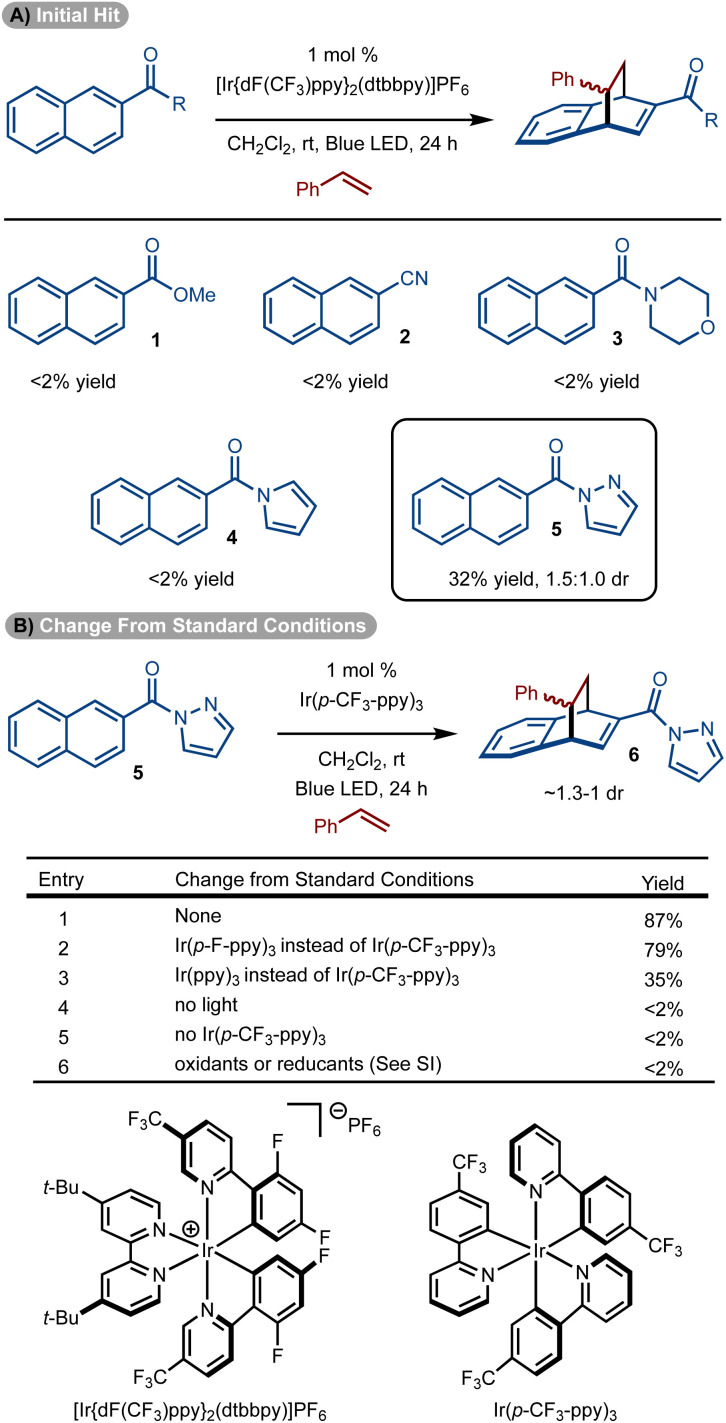
Initial studies and optimization.

Initial studies focused on the use of electron-deficient naphthalenes in a reaction with styrene.^14–23,32^ The choice to use these substrates stems from earlier work that suggest the triplet diradical is more capable of engaging an alkene if electron-withdrawing groups are present.^30,31^ Reaction with ester (1), cyano (2), and amide-substituted (3 and 4) naphthalenes did not allow for product formation. However, the use of pyrazole substrate 5 allowed for formation of 6 in 32% yield (Scheme 2A).^25^ While a single regioisomer was observed, a mixture of diastereomers was generated (1.5 : 1.0 dr). While the low diastereoselectivity is not ideal, the isomers are often easily separated. In addition, the chemistry is well suited for discovery chemistry where the generation of multiple diastereomers is often desirable so as to fully evaluate the chemical space of a scaffold.

Standard optimization of the reaction was conducted by evaluation of solvent, concentration, and photosensitizer to arrive at the conditions shown in Scheme 2B, entry 1.^33–36^ It was identified that homoleptic Ir-catalysts were superior to cationic complexes (see the ESI† for details). Within the series of homoleptic Ir-complexes evaluated, the highest yield was observed with [Ir(p-CF_3_-ppy)_3_] (*E*_T_ = 56.4 kcal mol^−1^) (Scheme 2B, entry 1). Similar yield was observed with a higher energy sensitizer [Ir(p-F-ppy)_3_] (*E*_T_ = 58.6 kcal mol^−1^) (Scheme 2B, entry 2). However, yield diminished with [Ir(ppy)_3_] which has a lower triplet energy (*E*_T_ = 55.2 kcal mol^−1^) (Scheme 2B, entry 3). Finally, control experiments without sensitizer, light or under other conditions failed to deliver the desired product (Scheme 2B, entries 4–6).

**Scheme 3:**
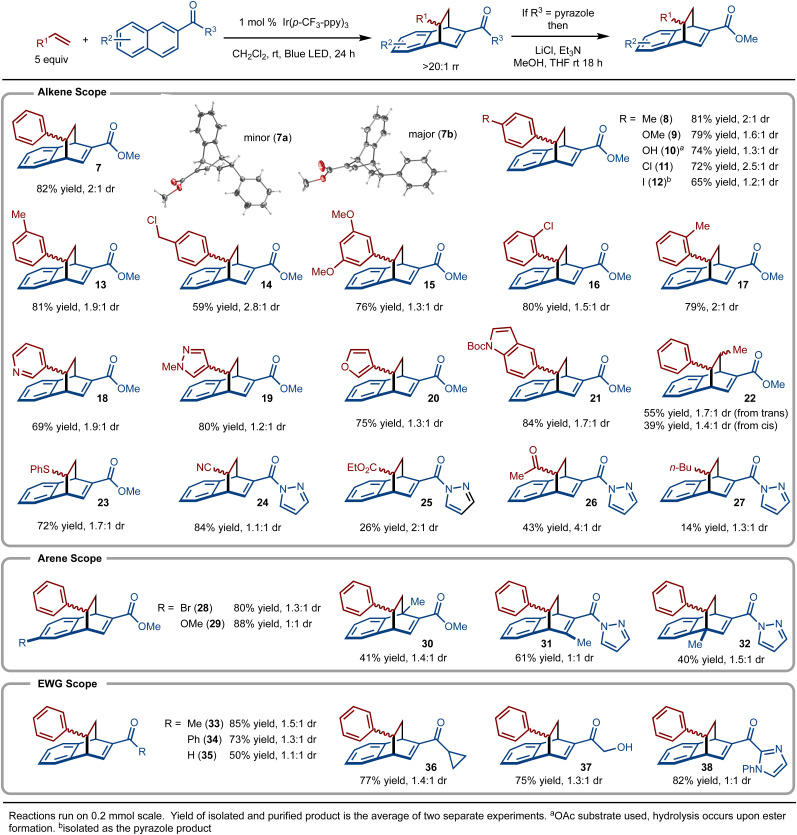
Substrate scope.

Under the optimal conditions, the scope of the method was evaluated (Scheme 3). It was found that styrene derivatives that bear electron-donating (product 9) and electron-withdrawing groups (product 11) were well tolerated. In addition, sterically demanding substituents (product 17) did not impede the reaction. Reactive functionality such as benzyl chlorides (product 14) and esters (product 10) were also tolerated. Various five- and six-membered heterocyclic substrates worked well (products 18–21). *trans*-β-Methylstyrene was investigated and gave rise to a mixture of anti-products regardless if the *cis*- or *trans*-alkene isomer was used (product 22). Substrates beyond styrene derivatives also functioned smoothly. For example, phenyl vinyl sulfide allowed for formation of 23. In addition, alkenes bearing electron withdrawing groups such as cyano (product 24), ester (product 25), and ketone (product 26) all allowed for product formation. Unactivated alkenes, did lead to product formation, albeit in low yield (product 27).

Substitution of the naphthyl ring was also explored (Scheme 3). For example, substitution at the 5-position with Br and OMe was well tolerated (products 28 and 29, respectively). Substrates with a methyl group at the 1-, 3-, and 4- positions were investigated and in all cases the products (products 30–32) were generated. For these examples, it is clear that the pyrazole amide substituent is the dominate group that controls regioselectivity.

During our investigations, it was discovered that ketone and aldehyde (product 35) derived naphthalenes also allowed for product formation (Scheme 3). For example, methyl and phenyl ketone derived substrates provided 33 and 34 in good yield, respectively. Additional examples demonstrate tolerance of strained rings (product 36),^32^ unprotected alcohols (product 37), and an imidazole heterocycle (product 38). It should also be noted that reaction with unactivated alkenes did not work with the ketone derived substrates, indicating that the pyrazole naphthalenes provide superior reactivity.

**Scheme 4:**
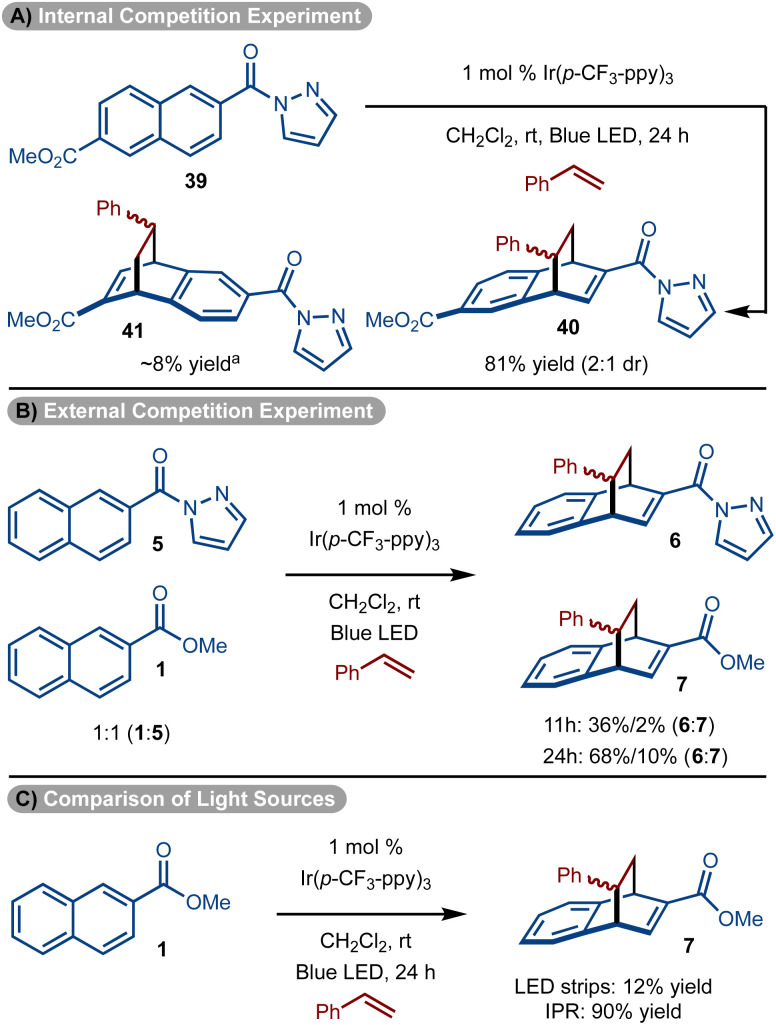
Relative reactivities. ^*a*^Determined by analysis of ^1^H NMR spectrum of the unpurified reaction mixture.

To further underscore the special reactivity of the pyrazole, a series of experiments were conducted. First substrate (39) that bears a pyrazole and ester substituent was subjected to the standard reaction conditions (Scheme 4A). The cycloaddition occurs with ∼10 : 1 preference for reaction proximal to the pyrazole unit to generate 40 as the major product. In addition, a competition experiment between 1 and 5 also showed pyrazole substrate 5 reacts significantly faster than the ester substrate 1 (Scheme 4B). Finally, it should be noted that with our standard reaction conditions and setup using our typical LED strips, the ester substrate reacted poorly (Scheme 4C). However, when an Integrated Photochemical Reactor (IPR)^37^ which supplies a significantly higher photon flux was used, ester substrate 1 did undergo productive reaction (Scheme 4C).

**Scheme 5:**
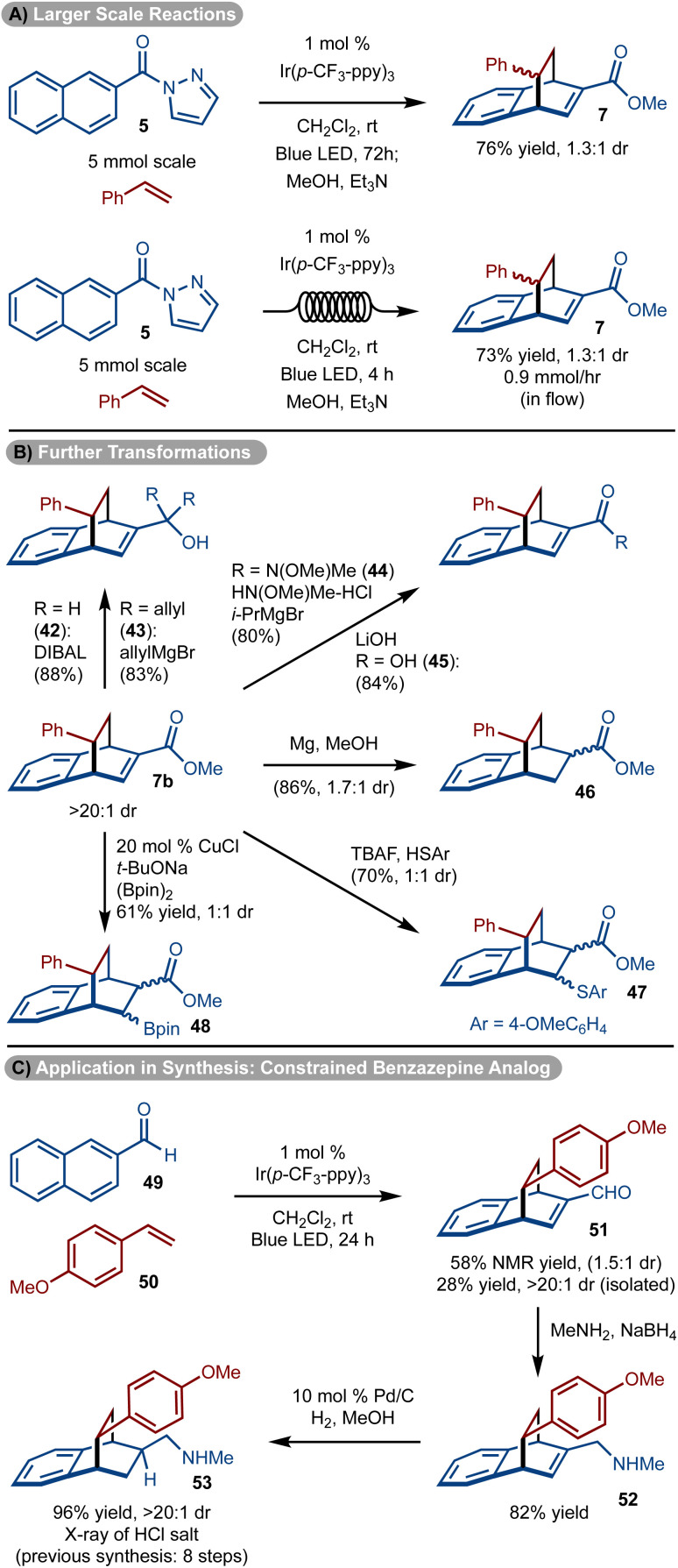
Larger scale and further functionalizations.

The reaction was easily scaled up to 5.0 mmol to allow for isolation of gram quantities of 7, however, the reaction time needed to be extended to 72 hours (Scheme 5A). To reduce the reaction time, the process was also conducted in flow and a similar quantity of product 7 could be prepared in only 4 hours (Scheme 5A).

The utility of the products was demonstrated in various ways with 7b (>20 : 1 dr, diastereomers separated) (Scheme 5B). The ester could be manipulated to provide access to either primary (product 42) or oxygen substituted tertiary carbons (product 43), *via* reduction and addition reactions, respectively. In addition, facile conversion to the Weinreb amide (product 44) and acid (product 45) could be achieved. Manipulation of the double bond could be carried out by conjugate addition of PhSH^38^ or (Bpin)_2_ ^39^ to generate 47 and 48 as a mixture of diastereomers, respectively. In addition, treatment with Mg/MeOH resulted in the reduction of the alkene to form 46. Finally, the products could be elaborated to drug like molecules (Scheme 5C). For example, amine 53 has shown promise as a conformationally constrained analog of a 1-benzazepin-2-one and was previously prepared *via* an eight-step sequence.^40^ Utilizing, the methods shown, a three-step route consisting of cycloaddition, reductive amination, and hydrogenation was accomplished.

**Scheme 6:**
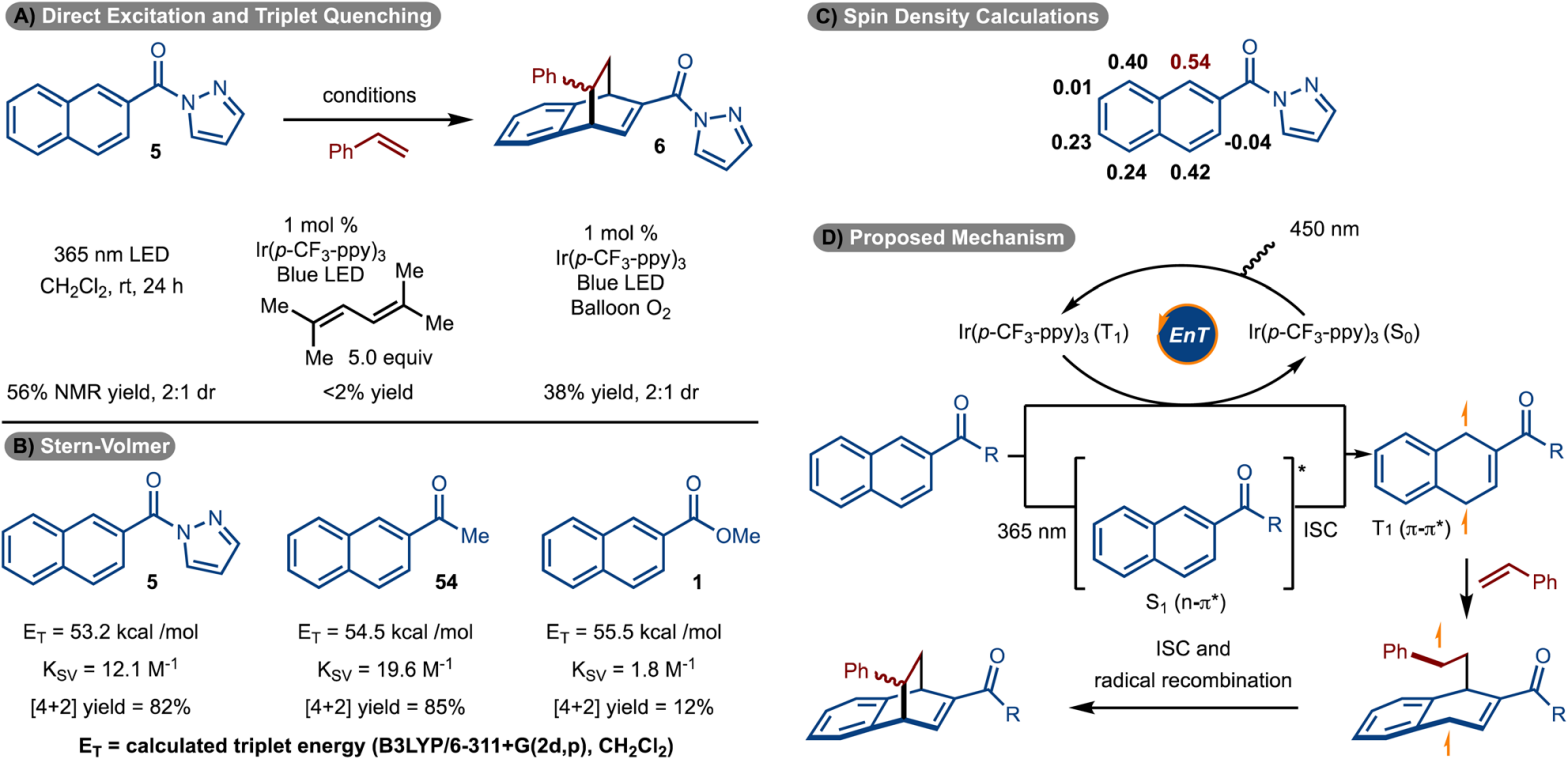
Mechanistic studies.

With respect to the mechanism, it was discovered that the reaction could proceed in the absence of sensitizer when 365 nm LEDs were used (Scheme 6A). This data strongly suggests that the reaction proceeds *via* excited state intermediates. In addition, when triplet state quenchers 1,1,4,4-tetramethyl butadiene and O_2_ were introduced, the reaction was suppressed.

Stern–Volmer quenching studies were conducted (Scheme 6B). It was found that pyrazole naphthalene 5 quenched the excited state of Ir(p-CF_3_-ppy)_3_ with a quenching constant of *K*_SV_ = 12.1 M^−1^. It was also found that ketone naphthalene 54 was also an effective quencher (*K*_SV_ = 19.6 M^−1^). In both cases, the cycloaddition reaction proceeded effectively (see products 6/7 and 33). Conversely, ester naphthalene 1 was poorly reactive under the standard reaction conditions and is consistent with it being a poor quencher (*K*_SV_ = 1.8 M^−1^). We suspect the increased electron withdrawing capability of the pyrazole and ketone relative to an ester is the source of the increased quenching of 5 and 54 relative to 1. Interestingly, the calculated triplet energies of 54 and 1, are similar, yet the quenching rate is an order of magnitude different. Finally, a spin density calculation was conducted on the triplet excited state of 5 (Scheme 6C). It was found that the C1 position had the highest site of spin density.

On the basis of the data illustrated in Scheme 6A–C, a mechanism is proposed in Scheme 6D. Photoinduced excitation of Ir(p-CF_3_-ppy)_3_ occurs to ultimately generate the triplet state. Dexter energy transfer of the naphthalene then occurs to form the T_1_ state. This intermediate can also be accessed *via* direct absorption by the naphthalene and ISC. Capture of the T_1_ state with the alkene occurs at the site of highest spin density (see Scheme 6C) to generate a triplet diradical. Finally, ISC and radical recombination leads to formation of the product. The ∼1 : 1 diastereoselectivity is a result of a non-selective radical recombination.

## Conclusions

In summary, a new intermolecular photochemical dearomatization reaction has been introduced. The reaction is notable in that it is intermolecular and does not require the use of specific heterocyclic scaffolds. Key to development of this reaction was the identification of a pyrazole amide that allowed by efficient energy transfer and cycloaddition. Due to the simplicity of the starting materials, a wide array of chemical diversity can be readily accessed. Finally, the bridged bicyclic products are useful intermediates and can aid in the synthesis of biologically significant molecules.^41^

## Data availability

The ESI† contains method description, product characterization data, and NMR spectra.

## Author contributions

MKB, WW and RG conceptualized the project. MKB supervised the investigation. WW and YYC performed the research. MKB wrote the paper. All authors analyzed the data, discussed the results, and commented on the manuscript.

## Conflicts of interest

There are no conflicts to declare.

## Supplementary Material

SC-013-D2SC04789F-s001

SC-013-D2SC04789F-s002
