# Supplementary material for: Synthesis of complex bicyclic scaffolds by intermolecular photosensitized dearomative cycloadditions of activated alkenes and naphthalenes
Source: Chem Sci. 2022 Oct 14;13(45):13582–7. doi: 10.1039/d2sc04789f (PMC9682912; doi:10.1039/d2sc04789f)
Supplement: SC-013-D2SC04789F-s001 [file SC-013-D2SC04789F-s001.pdf]

Electronic Supplementary Material (ESI) for Chemical Science.
This journal is © The Royal Society of Chemistry 2022

Synthesis of Complex Bicyclic Scaffolds by Intermolecular Photosensitized Dearomative Cycloadditions of Activated Alkenes and Naphthalenes

Wang Wang, Yanyao Cai, Renyu Guo, and M. Kevin Brown*

Indiana University, Department of Chemistry, 800 E. Kirkwood Avenue, Bloomington, IN 47405

Supplementary Information

Table of Contents

1. General..2

2. Reagents..3

3. Reaction Optimizations..5

4. Mechanistic Investigations..7

5. Experimental Procedures..19

6. Relative Reactivities..55

7. Gram scale synthesis..57

8. Further Derivatizations..58

9. Synthesis of drug molecules..63

10. X-ray structure..64

11. NMR Spectra..70

Supplementary References..142

1. General

NMR: ^{1}H NMR spectra were recorded at room temperature on a Varian I400 (400 MHz), Varian VXR400 (400 MHz), Varian I500 (500 MHz), Bruker 500 (500 MHz) or a Varian I600 (600 MHz) spectrometer. Chemical shifts are reported in ppm from tetramethylsilane with the residual solvent resonance as the internal standard ($CDCl_3$: 7.26 ppm). Data are reported as follows: chemical shift, multiplicity (s = singlet, d = doublet, t = triplet, q = quartet, br = broad, m = multiplet), coupling constants (Hz), and integration. ^{13}C NMR spectra were recorded on a Varian I400 (101 MHz), Varian I500 (126 MHz) or Bruker 500 (126 MHz) spectrometer with complete proton decoupling. Chemical shifts are reported in ppm from tetramethylsilane with the solvent resonance as the internal standard ($CDCl_3$: 77.16 ppm , $CDCl_2$: 53.84 ppm). ^{19}F NMR spectra were recorded on a Varian VXR400 (376 MHz) spectrometer.

IR: Infrared (IR) spectra were recorded on a Bruker Tensor II FT-IR Spectrometer, ν_{max} in cm^{-1}. Bands are characterized as broad (br), strong (s), medium (m), and weak (w).

HRMS: High Resolution Mass Spectrometry (HRMS) analysis was obtained using Electrospray Ionization (ESI) and reported as m/z (relative intensity). ESI was acquired using a Waters/Micromass LCT Classic (ESI-TOF).

Solvents: Dichloromethane (DCM), Tetrahydrofuran (THF), Diethyl ether (Et_2O), dioxane and Dimethylformamide (DMF) were purified under a positive pressure of dry argon by passage through two columns of activated alumina. Toluene (PhMe) was purified under a positive pressure of dry argon by passage through columns of activated alumina and Q5 (Grubbs apparatus).

Reactions: Unless otherwise noted, all reactions have been carried out with distilled and degassed solvents under an atmosphere of dry N_2 in oven- (150 °C) and flame-dried glassware with standard vacuum-line techniques. All work-up and purification procedures were carried out with reagent grade solvents in air.

Purification: Standard flash column chromatography (FCC) techniques using ZEOprep 60/40-63 µm silica gel were used for purification. For difficult separations, medium-pressure liquid chromatography (MPLC) was performed using a Teledyne ISCO CombiFlash Rf 150 instrument.

Light source: Double-density sapphire blue LEDs strip (450 nm) was purchased from Creative Lighting Solutions[1]. realUV™ LED Strip Lights (365 nm) was purchased from Waveform Lighting[2]. The strip was wrapped around a 125 × 65 mm crystallization dish 4-5 times. Reactions were generally placed ~1 cm away from the lights (the walls of the dish), by placing them in a vial holder (see Experimental sections for details).

Flow System: Gram-scale reactions were performed on Vapourtec E-series Flow Chemistry System with a UV-150 photochemical reactor, equipped with a 150 W 400

nm LED (see Experimental sections for details)[3].

2. Reagents

Allylmagnesium bromide was purchased from Sigma-Aldrich and titrated before every use.

2-Acetonaphthone was purchased from Ambeed.

Ammonium cerium(IV) nitrate (CAN) was purchased from Sigma-Aldrich.

1-Bromo-2-naphthoic acid was purchased from Ambeed.

6-Bromo-2-Naphthoic acid was purchased from Oakwood.

***n*-Butyllithium solution (*n*BuLi)** was purchased from Sigma-Aldrich and titrated before every use.

Copper (I) chloride (CuCl) was purchased from Strem and used as received.

Cyclopropylmagnesium bromide (*c*-PropylMgBr) was purchased from Sigma-Aldrich.

CPhos was purchased from Strem.

2,3-Dimethyl-1,3-butadiene was purchased from TCI and filtered through a pad of silica before use.

DiisobutylaluMinuM hydride (DIBAL) was purchased from Sigma-Aldrich.

4-Dimethylamino pyridine (DMAP) was purchased from Oakwood.

Di-tert-butyl decarbonate ($(Boc)_2O$) was purchased from Oakwood.

1-Ethyl-3-(3′-dimethylaminopropyl)carbodiimide hydrochloride (EDC) was purchased from Oakwood.

3-Furancarboxaldehyde was purchased from Sigma-Aldrich.

1H-Pyrazole was purchased from Oakwood.

Lithium hydroxide monohydrate ($LiOH·H_2O$) was purchased from Sigma-Aldrich and used as received.

2-Isopropylthioxanthone (ITX) was purchased from CombiBlocks.

Iron(III) acetylacetonate ($Fe(acac)_3$) was purchased from Sigma-Aldrich.

Iridium(III) Chloride Hydrate ($IrCl_3·nH_2O$) was purchased from TCI.

6-Methoxy-2-Naphthoic acid was purchased from Ambeed.

6-(Methoxycarbonyl)-2-Naphthoic acid was purchased from Ambeed.

Methanamine (40% in Methanol) was purchased from TCI.

Magnesium powder (Mg) was purchased from Strem.

Morpholine was purchased from Alfa Aesar.

***cis-β*-Methylstyrene** was purchased from Oakwood and filtered through a pad of silica before use.

***trans-β*-Methylstyrene** was purchased from TCI and filtered through a pad of silica before use.

Methyltriphenylphosphonium bromide ($MePPh_3Br$) was purchased from Ambeed and used as received.

4-Methoxystyrene was purchased from Oakwood and filtered through a pad of silica before use.

Methylmagnesium bromide (MeMgBr, 3M in ether) was purchased from Alfa Aesar.

1-Methyl-1H-pyrazole-4-carbaldehyde was purchased from Oakwood.

2-Naphthoic acid was purchased from Ambeed.

2-Naphthaldehyde was purchased from Ambeed.
2-Naphthonitrile was purchased from Ambeed.
***N,O*-Dimethylhydroxylamine hydrochloride [MeNH(OMe)·HCl]** was purchased from Oakwood and used as received.
Oxalyl chloride [$(COCl)_2$] was purchased from Oakwood and used as received.
Palladium on activated charcoal (Pd/C) was purchased from Sigma-Aldrich.
Pyrrole was purchased from Sigma-Aldrich.
Potassium *tert*-butoxide (*t*BuOK) was purchased from Strem and used as received.
Pyridinium chlorochromate (PCC) was purchased from Fluka.
3-Pyridinecarboxaldehyde was purchased from Sigma-Aldrich.
Samarium(II) iodide solution (0.1M in THF) (SmI_2) was purchased from Sigma-Aldrich.
Sodium tert-butoxide (*t*BuONa) was purchased from Strem.
Sodium borohydride ($NaBH_4$) was purchased from Sigma-Aldrich and used as received.
Sodium carbonate (Na_2CO_3) was purchased from Fisher Scientific.
Sodium formate (HCOONa) was purchased from Sigma-Aldrich.
Tetrakis(triphenylphosphine)palladium ($Pd(PPh_3)_4$) was purchased from Strem.
Tetrakis(dimethylamino)ethylene (TDAE) was purchased from Sigma-Aldrich.
Tetrabutylammonium Fluoride (TBAF) was purchased from Sigma-Aldrich.
Thiophenol (PhSH) was purchased from Alfa Aesar.
Triethylamine (Et_3N) was purchased from VWR and distilled over CaH_2 before use.
Triphenylphosphine (PPh_3) was purchased from Oakwood and used as received.
2-Vinylnaphthalene was purchased from Alfa Aesar and used as received.
Vinyl acetate was purchased from Oakwood and used as received.
Zinc Chloride ($ZnCl_2$) was purchased from Alfa Aesar.

Photocatalysts:
$[Ir(dF(CF_3)ppy)_2(dtbbpy)]PF_6$ was purchased from Strem and used as received.
$[Ir(dF(CF_3)ppy)_2(bpy)]PF_6$ was purchased from Strem and used as received.
$[Ir(dFppy)_2dtbbpy]PF_6$ was purchased from Strem and used as received.
[Ir(*p*-F(Me)ppy)$_2$dtbbpy]PF_6 was purchased from Strem and used as received.
$[Ir(ppy)_2dtbbpy]PF_6$ was purchased from Strem and used as received.
$[Ir(dtbppy)_2dtbbpy]PF_6$ was purchased from Strem and used as received.
[Ir(*p*-F(Me)ppy)$_2$dtbbpy]PF_6 was purchased from Strem and used as received.
$[Ir(ppy)_2dtbbpy]PF_6$ was purchased from Strem and used as received.
$[Ir(dtbppy)_2dtbbpy]PF_6$ was purchased from Strem and used as received.
$Ru(bpy)_3(PF_6)_2$ was purchased from Strem.
***fac*-Ir(dFppy)$_3$** was purchased from Strem and used as received.
***fac*-Ir(*p*-Fppy)$_3$** was purchased from Sigma Aldrich and used as received.
***fac*-Ir(*p*-CF$_3$ppy)$_3$** was purchased from Strem and used as received.
***fac*-Ir(ppy)$_3$** was purchased from Strem and used as received.

3. Reaction Optimizations

Table S1. Screening different activating groups[a]

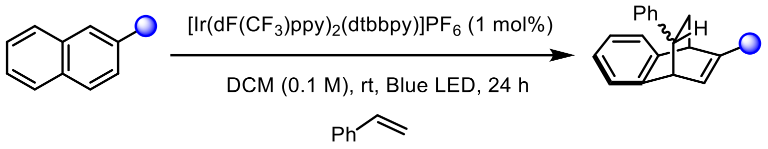

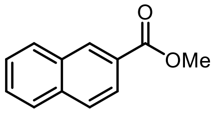

1

<2% yield

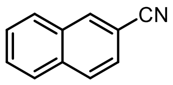

2

<2% yield

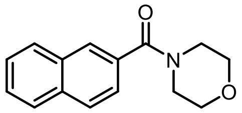

3

<2% yield

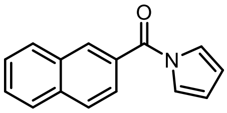

4

<2% yield

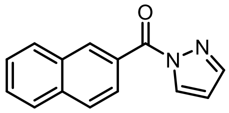

5

32% yield, dr=1.5:1

a) Reactions were run on 0.1 mmol scale; Yields were determined by crude ^{1}H-NMR using $Cl_2CHCHCl_2$ as the internal standard.

Comments: *Initial screening of different activating groups such as nitrile, ester and amide failed to detect any products. Desired cycloaddition product could be observed with acyl pyrazole as activating group.*

Table S2. Photosensitizer screening

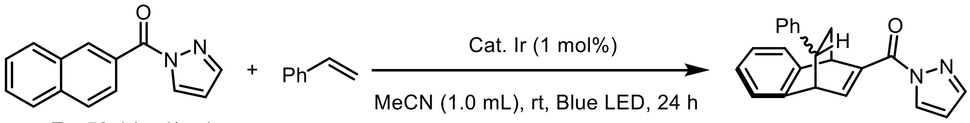

Eτ=53.1 kcal/mol

5

6

Entry[a]	Cat. Ir	Eτ (kcal/mol)	SM recovered (%)	yield (%)	*dr*
1	[Ir{dF(CF$_3$)ppy}$_2$(dtbpy)]PF$_6$	60.1	65	32	1.3:1
2	[Ir{dFCF$_3$ppy}$_2$(bpy)]PF$_6$	60.4	73	28	1.4:1
3	[Ir(dFppy)$_2$(dtbbpy)]PF$_6$	57.1	70	29	1.6:1
4	Ir(dtbppy)$_2$(dtbbpy)PF$_6$	51.1	72	14	1.9:1
5	[Ir(p-F(Me)ppy)$_2$-(4,4'-dtbbpy)]PF$_6$	53.3	28	71	1.3:1
6	[Ir(ppy)$_2$(dtbbpy)]PF$_6$	49.2	33	58	1.5:1
7	Ir(ppy)$_3$	55.2	68	35	1.2:1
8	Ir(p-CF$_3$-ppy)$_3$	56.4	8	87	1.4:1
9	Ir(p-F-ppy)$_3$	58.6	11	79	1.3:1
10	Ru(bpy)$_3$(PF$_6$)$_2$	46.0	100	0	0
11[b]	Ir(p-CF$_3$-ppy)$_3$	56.4	100	0	-
12[c]	-		100	0	-

a) Reactions were run on 0.1 mmol scale; Yields were determined by crude ^{1}H-NMR using $Cl_2CHCHCl_2$ as the internal standard. b) no light. c) no photosensitizer

The triplet state energy of photosensitizer was adapted from known literature[4].

Comments: *Our initial screening of different photosensitizers found Ir(p-CF$_3$-ppy)$_3$ (entry 8) gave the desired product with a good yield. Most of the low yields are due to low conversion of start material and dimerization of styrene. Entries 11 and 12 show that the photosensitizers and Blue LED are essential for this reaction.*

Table S3. Screening of solvent

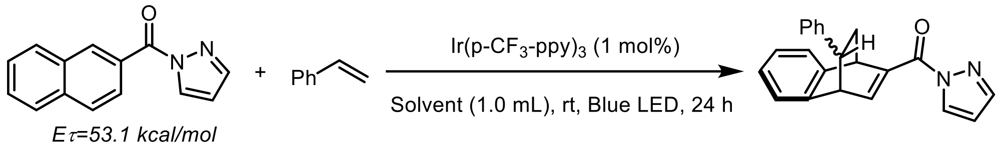

5

6

Entry[a]	Solvent	SM recovered (%)	yield (%)	*dr*
1	THF	21	76	1.5:1
2	Dioxane	23	72	1.2:1
3	Et_2O	55	45	1.5:1
4	DCE	10	82	1.3:1
5	DCM	5	89	1.4:1
6	Toluene	32	60	1.3:1
7	Acetone	36	58	1.7:1
8	DMF	28	65	1.5:1

a) Reactions were run on 0.1 mmol scale; Yields were determined by crude ^{1}H-NMR using $Cl_2CHCHCl_2$ as the internal standard.

Comments: *Most common solvents gave desired product with varied yield and dr. DCM showed the best conversion.*

Unsuccessful examples

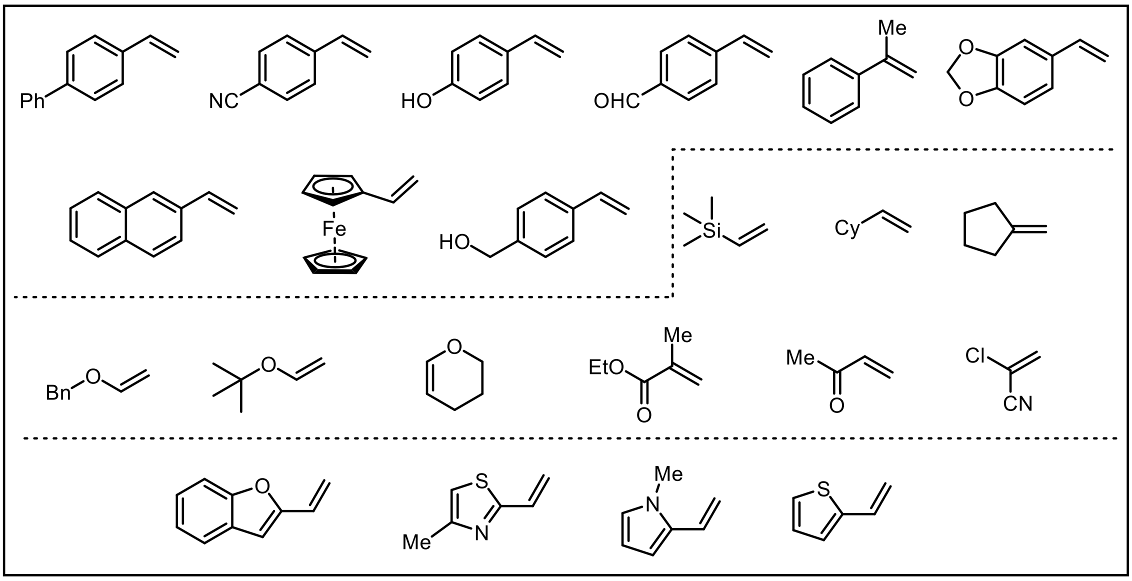

4. Mechanistic Investigations

4.1 Stern-Volmer luminescence quenching experiment

Stern-Volmer luminescence quenching experiment was conducted by using fluorescence spectrophotometer (Edinburgh Instruments FLS-1000). Linear regression of I_0/I against concentration was performed in Excel. I_0 is the luminescence intensity without the quencher, I is the intensity in the presence of the quencher. All samples for luminescence quenching experiment were prepared in 3.0 mL sealed quartz cuvettes inside nitrogen filled glovebox and done at room temperature.

The solution of Ir[*p*-CF_3-ppy]$_3$ (0.05 mM in dichloromethane) was excited at λ_{ex}= 400 nm and the emission was collected at 553 or 580 nm. The substrates were dissolved in dichloromethane (300 mM) respectively. For each quenching experiment, 2 μL of the stock solution were titrated to a solution (3.0 mL) of Ir[*p*-CF_3-ppy]$_3$ in a screw-top 10.0 mm quartz cuvette. The addition of 2 μL stock solution refers to an increase of the quencher concentration of 0.2 mM. (The change of total volume of the solution was ignored.)

	C (mM)	0	2	4	6	8
Amide	Intensity I	6637063	6395810.5	6245987	1.104372	6009807
**(5)**	I_0/I	1.000000	1.037720	1.062612	1.087231	1.104372
Styrene	Intensity I	6836389	6870773	6859446	6851082	6802076
	I_0/I	1.000000	0.994996	0.996639	0.997855	1.005044
Ketone	Intensity I	8913165	8517746	8121370.5	7828676	7511035
**(54)**	I_0/I	1.000000	1.046423	1.097495	1.138528	1.186676
Ester	Intensity I	9182074	9289974	9282240	9244616	9194074
**(1)**	I_0/I	1.000000	0.988385	0.989209	0.993235	0.998695

	C (mM)	10	12	16	20
Amide **(5)**	Intensity I	5846672	5707330	5548666	5305915
	I_0/I	1.135186	1.162902	1.196155	1.250880
Styrene	Intensity I	6767105	6730828	6649732	6498386
	I_0/I	1.010238	1.015683	1.028070	1.052013
Ketone **(54)**	Intensity I	7201679	7074865.5	6723652	6404440
	I_0/I	1.237651	1.259835	1.325643	1.391717
Ester **(1)**	Intensity I	9184472	9121972	9038912	8925913
	I_0/I	0.999739	1.006589	1.015838	1.028699

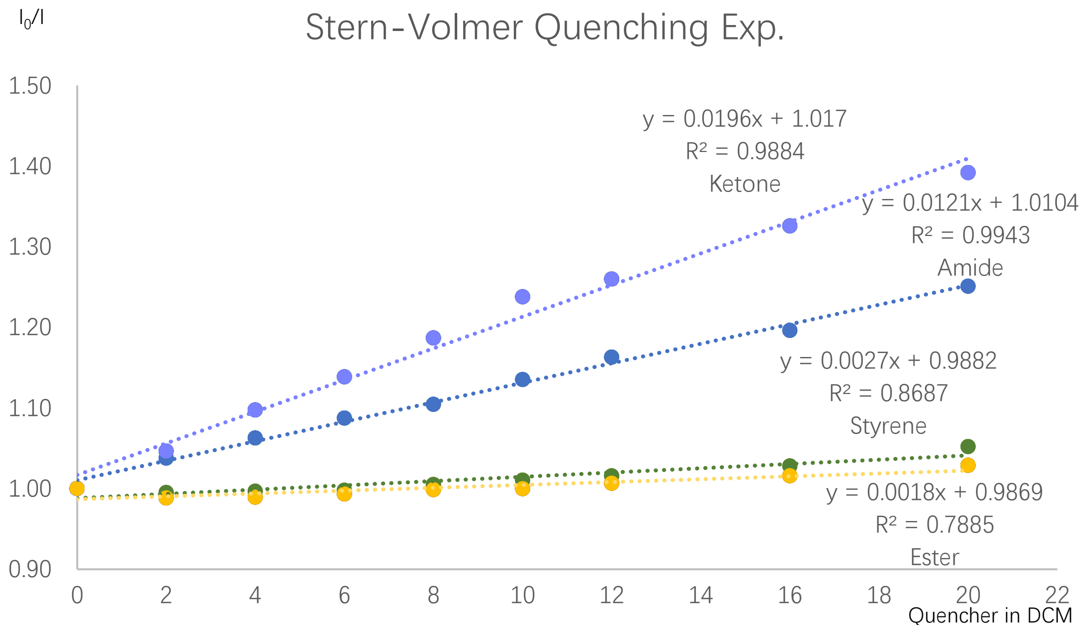

Figure S1. Stern-Volmer luminescence quenching experiment

Comments: *Compared with ester and styrene, Amide* ***(5)*** *and ketone* ***(54)*** *show stronger quenching ability to Ir catalyst under our reaction condition.*

4.2 UV/vis spectrums

Ultraviolet-visible (UV-Vis) spectra was measured on a Varian Cary 5000 UV-vis NIR spectrophotometer at room temperature with solution in 3 mL cuvettes.

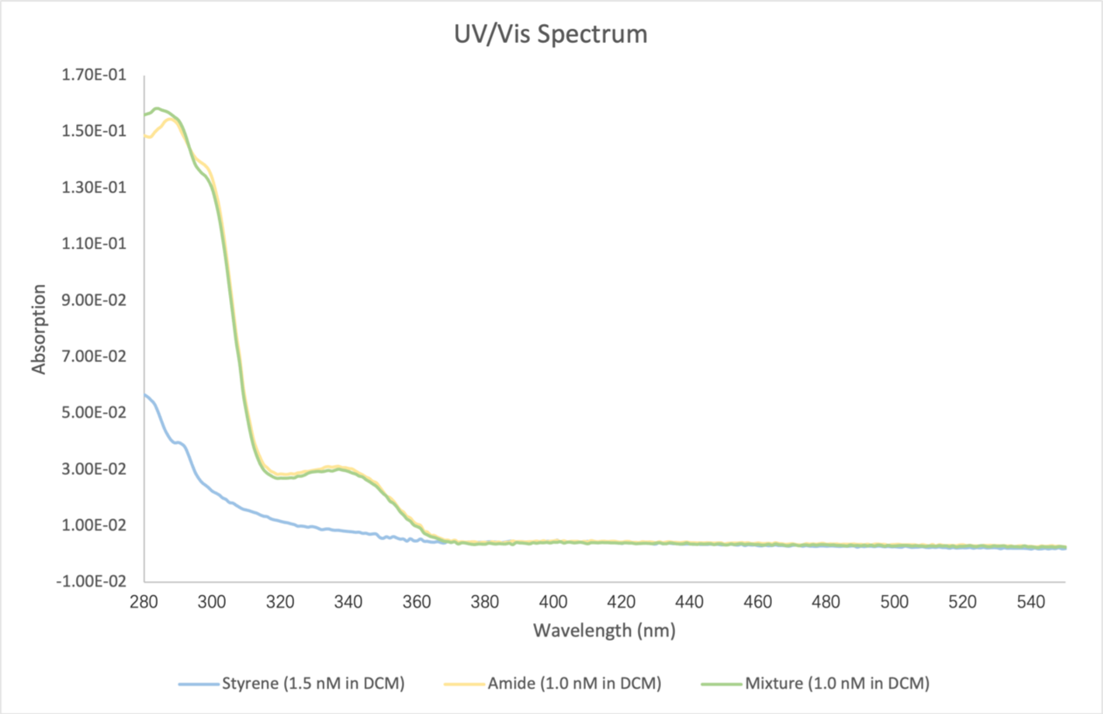

Figure S2. UV/vis spectrums

Comments: *There is no differences of absorption between the mixture (amide* ***(5)*** */styrene) and amide* ***(5)*** *for wavelength range (>300 nm), which rules out the formation of donor-acceptor (EDA) complex intermediate.*

4.3 Electrochemical measurements

Electrochemical measurements were recorded on a CHI 620E electrochemical analyzer (CH Instruments). Cyclic voltammetry experiments were carried out in a single compartment cell. All CV experiments performed in non-aqueous solvents were carried out under a nitrogen atmosphere in an MBraun Labmaster glovebox. Working electrode: glassy carbon electrode (3 mm diameter, CH Instruments). Auxiliary electrode: platinum wire (Alfa Aesar, 99.99%). Pseudo-reference electrode: Ag wire (Alfa Aesar, 99.99%). The final redox potentials measured under non-aqueous conditions were calculated by comparing the measured potentials with the $E_{1/2}$ of Fc/Fc^{+} in the same solution and then converted to a potential *vs*. SCE by adding 0.38 V. The reproducibility of all cyclic voltammetry experiments was verified by several repeated scans. All measurements were performed in 1.0 M $TBAPF_6$/THF with 2 mM compound and 2 mM ferrocene as reference.

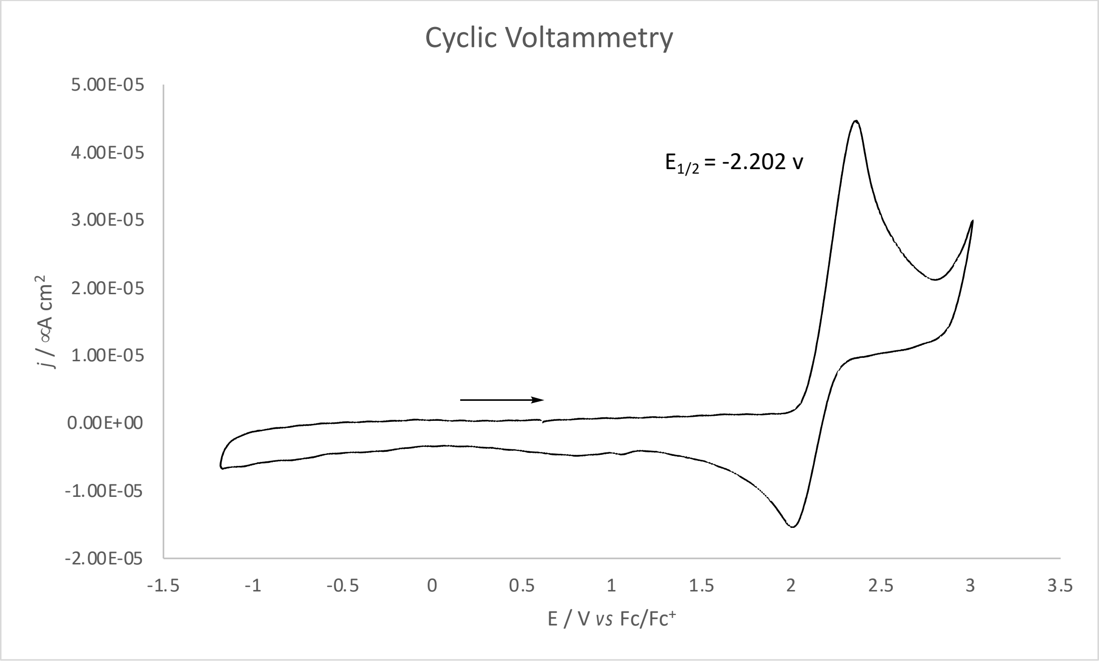

Figure S3. Electrochemical measurements

Comments: *The oxidation potential of Ir(p-CF$_3$-ppy)$_3$ (-1.70 V)[4a] was lower than the reduction potential of amide (-1.82 V), which indicates the reduction of amide is unfavorable. No oxidation potential of amide was observed in the reductive region. The above data suggests photoredox here is unlikely to happen.*

4.4 Control Experiments

Procedure for control experiment (A): An oven-dried 4 mL screw-cap vial equipped with a stir bar was cooled under vacuum. After being evacuated/backfilled with N_2 (x 3) and capped with a septum, the vial was transferred into the glovebox and charged with ITX (5.1 mg, 20 mol%) and acyl pyrazole (22 mg, 0.10 mmol, 1.0 eq.). After moved out, styrene (115 μL, 1.00 mmol, 10.0 eq.) and dry DCM (1 mL) were sequentially added to the vial. Then the septum was replaced with a cap. The vial was capped and the reaction was stirred under blue LED irradiation with a cooling fan to keep room temperature. After 24 h, the solution was concentrated by rotary evaporation. 1,1,2,2-tetrachlorethan (10.5 μL, 0.100 mmol) was added to the residue for crude ^{1}H-NMR to determine dr (54% NMR yield).

A: *Organic triplet sensitizers replicate [4+2] cycloaddition, albeit with slower rate*

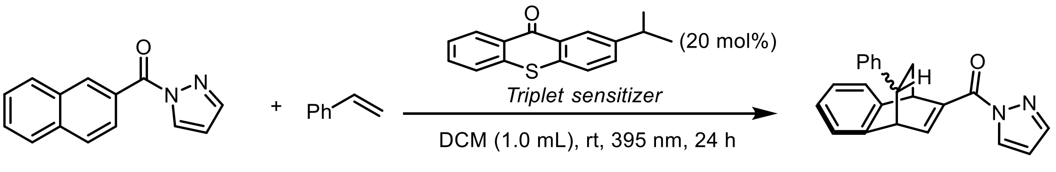

5 **6:** 54% NMR yield

Procedure for control experiment (B): An oven-dried 4 mL screw-cap vial equipped with a stir bar was cooled under vacuum. After being evacuated/backfilled with N_2 (x 3) and capped with a septum, the vial was transferred into the glovebox and charged with chemical reducant SmI_2 (100 μL , 0.10 mmol, 1.0 eq., 0.1M in THF) and acyl pyrazole (22 mg, 0.10 mmol, 1.0 eq.). After moved out, styrene (115 μL, 1.00

mmol, 10.0 eq.) and dry DCM (1 mL) were sequentially added to the vial. Then the septum was replaced with a cap. The reaction was stirred under room temperature. After 24 h, the solution was concentrated by rotary evaporation. 1,1,2,2-tetrachlorethan (10.5 μL, 0.100 mmol) was added to the residue for crude ^{1}H-NMR to determine dr (No product detected).

B: No reaction with chemical reducant

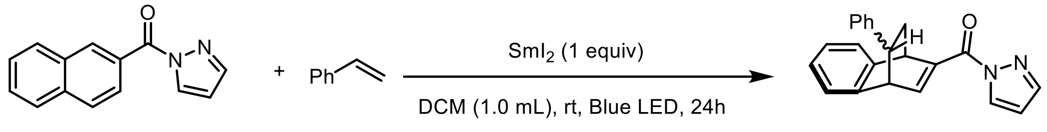

5

6: no product detected

Procedure for control experiment (C): An oven-dried 4 mL screw-cap vial equipped with a stir bar was cooled under vacuum. After being evacuated/backfilled with N_2 (x 3) and capped with a septum, the vial was transferred into the glovebox and charged with chemical oxidant CAN (54.8 mg, 0.100 mmol, 1.00 eq.) and acyl pyrazole (22 mg, 0.10 mmol, 1.0 eq.). After moved out, styrene (115 μL, 1.00 mmol, 10.0 eq.) and dry DCM (1 mL) were sequentially added to the vial. Then the septum was replaced with a cap. The reaction was stirred under room temperature. After 24 h, filtered through a short plug and concentrated by rotary evaporation. 1,1,2,2-tetrachlorethan (10.5 μL, 0.100 mmol) was added to the residue for crude ^{1}H-NMR to determine dr (No product detected).

C: No reaction with chemical oxidant

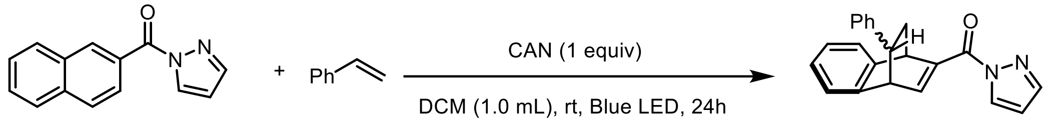

5

6: no product detected

Procedure for control experiment (D): An oven-dried 4 mL screw-cap vial equipped with a stir bar was cooled under vacuum. After evacuated/backfilled with N_2 (x 3) and capped with a septum, the vial was transferred into glovebox and charged with acyl pyrazole (22 mg, 0.10 mmol, 1.0 eq.). After moved out, styrene (115 μL, 1.00 mmol, 10.0 eq.) and dry DCM (1 mL) were sequentially added to the vial. Then the septum was replaced with a cap. The vial was capped and the reaction was stirred under UV irradiation with a cooling fan to keep room temperature. After 24 h, filtered through a short plug and concentrated by rotary evaporation. 1,1,2,2-tetrachlorethan (10.5 μL, 0.100 mmol) was added to the residue for crude ^{1}H-NMR to determine dr (56% NMR yield).

D: Reaction under UV conditions

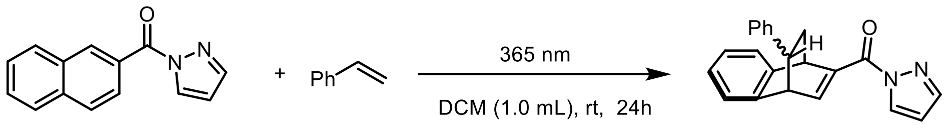

5

6: 56% NMR yield

Procedure for control experiment (E): An oven-dried 4 mL screw-cap vial equipped with a stir bar was cooled under vacuum. After being evacuated/backfilled with N_2 (x 3) and capped with a septum, the vial was transferred into the glovebox and charged with Ir(*p*-CF_3-ppy)$_3$ (0.8 mg, 1 mol%) and acyl pyrazole (22 mg, 0.10 mmol, 1.0 eq.). After moved out, styrene (115 μL, 1.00 mmol, 10.0 eq.), 2,5-Dimethyl-2,4-hexadiene (42 μL, 0.30 mmol, 3.0 eq.) and dry DCM (1 mL) were sequentially added to the vial. Then the septum was replaced with a cap. The vial was capped and the reaction was stirred under blue LED irradiation with a cooling fan to keep room temperature. After 24 h, the solution was concentrated by rotary evaporation. 1,1,2,2-tetrachlorethan (10.5 μL, 0.100 mmol) was added to the residue for crude ^{1}H-NMR to determine dr (< 2% NMR yield).

E: *Triplet quencher great impact on productivity of [4+2] cycloaddition*

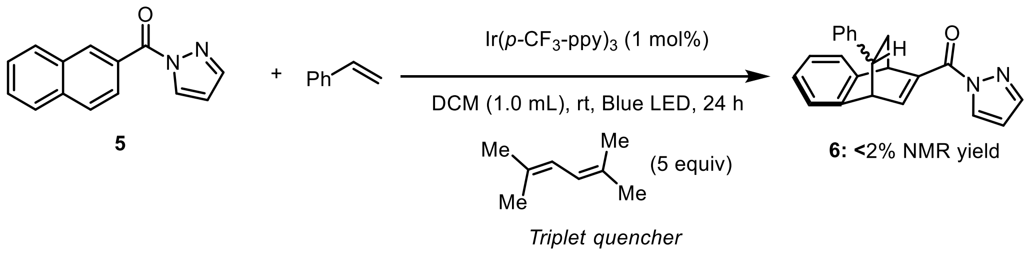

Procedure for control experiment (F): An oven-dried 4 mL screw-cap vial equipped with a stir bar was cooled under vacuum. After being evacuated/backfilled with N_2 (x 3) and capped with a septum, the vial was transferred into the glovebox and charged with Ir(*p*-CF_3-ppy)$_3$ (0.8 mg, 1 mol%) and acyl pyrazole (22 mg, 0.10 mmol, 1.0 eq.). After moved out, styrene (115 μL, 1.00 mmol, 10.0 eq.), dry DCM (1 mL) were sequentially added to the vial. Then the septum was filled with oxygen balloons. The reaction was stirred under blue LED irradiation with a cooling fan to keep room temperature. After 24 h, the solution was concentrated by rotary evaporation. 1,1,2,2-tetrachlorethan (10.5 μL, 0.100 mmol) was added to the residue for crude ^{1}H-NMR to determine dr (38% NMR yield).

F: *Triplet quencher great impact on productivity of [4+2] cycloaddition*

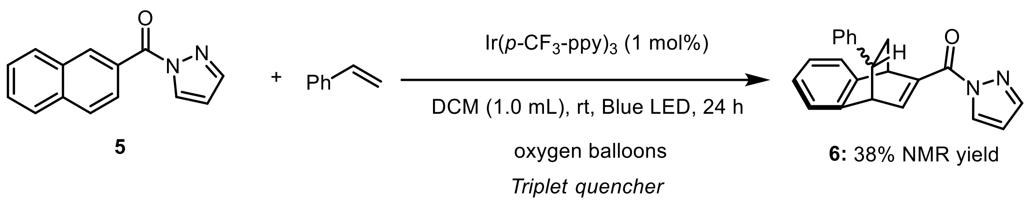

Comments: *All the outcomes of control experiments support the hypothesis that the iridium catalyzed the [4+2] cycloaddition reaction proceeds through a triplet energy transfer process rather than an electron transfer mechanism.*

4.5 Triplet energy calculation

Triplet energies were calculated in accordance with previous reports by Yoon[5] and

Houk[6]. All the calculations were performed using Gaussian 16 quantum chemistry program package[7]. Both the singlet and triplet states of naphthalen-2-yl(1H-pyrazol-1-yl)methanone were optimized using B3LYP[8]/6-311+G(2d,p)[9] with the SMD solvent=DiChloroMethane model. Frequency analysis using a simple harmonic oscillation model was performed on the optimized structures to confirm that both structures were stationary on the potential energy surface. The analysis also gave the free energies for the two structures. The S–T gap was computed as the difference between the two free energies (ΔG).

Compound	S_0	T_1	T_1 - S_0 (E_T)
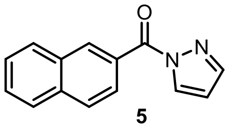 **5**	-724.293540	-724.208723	0.084817 = 53.2 kcal/mol
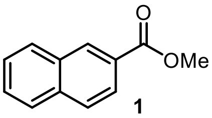 **1**	-613.818311	-613.729903	0.088408 = 55.5 kcal/mol
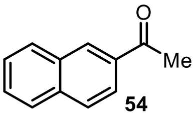 **54**	-538.566873	-538.480030	0.086843 = 54.5 kcal/mol

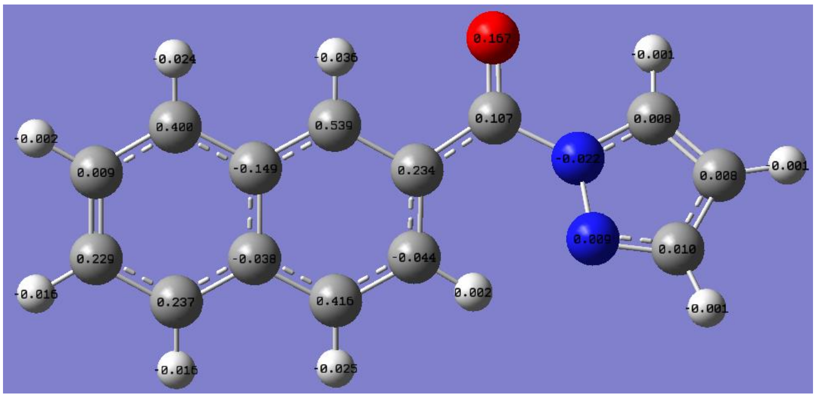

Figure S4. Spin density of Naphthalen-2-yl(1H-pyrazol-1-yl)methanone T_1 in DCM

Energies and coordinats:

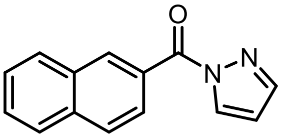

5

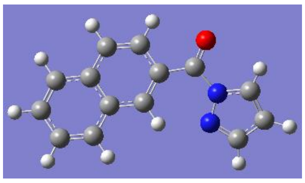

S0 in DCM

Zero-point correction= 0.207347 (Hartree/Particle)

Thermal correction to Energy= 0.219890

Thermal correction to Enthalpy= 0.220834

Thermal correction to Gibbs Free Energy= 0.167090
Sum of electronic and zero-point Energies= -724.253284
Sum of electronic and thermal Energies= -724.240741
Sum of electronic and thermal Enthalpies= -724.239797
Sum of electronic and thermal Free Energies= -724.293540
0 1

C	-9.10397505	0.29321171	1.95921926
C	-9.31370385	-0.97347433	1.57342810
C	-8.40026989	-1.59761494	0.81463698
C	-7.27605538	-0.96203284	0.43706887
C	-7.06121413	0.31024722	0.82297568
C	-7.98315085	0.92886930	1.58486410
C	-6.35000664	-1.57420017	-0.31926340
C	-5.23088209	-0.93368928	-0.69109558
C	-4.99962600	0.34240400	-0.32586300
C	-5.93342523	0.94034473	0.44277070
C	-3.85854200	1.00547500	-0.69892100
O	-3.01792560	0.46093152	-1.39340095
N	-3.61960167	2.20745126	-0.35138146
C	-3.22413889	3.14948241	-1.11309260
C	-2.96665592	4.21478798	-0.34648392
C	-3.21855921	3.76709893	0.88877392
N	-3.59530149	2.56039711	0.83848055
H	-9.85210530	0.81010502	2.58351042
H	-10.23353418	-1.49924511	1.87987531
H	-8.59196805	-2.64040172	0.50909083
H	-7.83825697	1.97083562	1.91818198
H	-6.49234041	-2.62068384	-0.64071156
H	-4.51350542	-1.51628809	-1.29061083
H	-5.79964572	1.98141704	0.78070519
H	-3.12193496	3.05142942	-2.20061816
H	-2.61361214	5.20543583	-0.65376403
H	-3.11810338	4.32910179	1.82582691

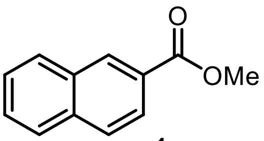

1

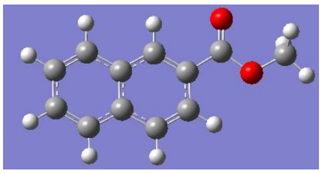

S0 in DCM
Zero-point correction= 0.189208 (Hartree/Particle)
Thermal correction to Energy= 0.200609
Thermal correction to Enthalpy= 0.201553
Thermal correction to Gibbs Free Energy= 0.151127
Sum of electronic and zero-point Energies= -613.780229

Sum of electronic and thermal Energies= -613.768829
Sum of electronic and thermal Enthalpies= -613.767885
Sum of electronic and thermal Free Energies= -613.818311
0 1

C	-1.35890800	-0.56115800	0.00000000
C	0.00491900	-0.92804200	0.00000100
C	-1.70153100	0.82625100	0.00000000
C	-0.65758700	1.78595800	0.00000000
C	0.65621100	1.40219600	0.00000100
C	0.99809800	0.02522900	0.00000100
C	2.41202200	-0.44097600	0.00000100
O	2.75424200	-1.60077800	0.00000000
C	-2.39787600	-1.52643900	0.00000000
C	-3.71286000	-1.13939900	0.00000000
C	-4.05101200	0.23259000	-0.00000100
C	-3.06971800	1.19129200	0.00000000
O	3.29289000	0.58381200	0.00000000
C	4.67942300	0.20638500	-0.00000200
H	0.28381000	-1.97478000	0.00000100
H	-0.91624400	2.83897000	0.00000000
H	1.44435300	2.14197100	0.00000100
H	-2.13347800	-2.57788200	0.00000000
H	-4.49938700	-1.88444200	-0.00000100
H	-5.09433400	0.52530100	-0.00000100
H	-3.33161500	2.24352500	0.00000000
H	5.23488000	1.14082200	-0.00000600
H	4.91393700	-0.38053400	0.88771200
H	4.91393300	-0.38054000	-0.88771200

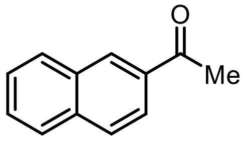

54

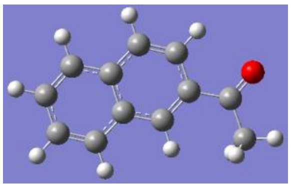

S0 in DCM
Zero-point correction= 0.183725 (Hartree/Particle)
Thermal correction to Energy= 0.194144
Thermal correction to Enthalpy= 0.195089
Thermal correction to Gibbs Free Energy= 0.147322
Sum of electronic and zero-point Energies= -538.530470
Sum of electronic and thermal Energies= -538.520050
Sum of electronic and thermal Enthalpies= -538.519106
Sum of electronic and thermal Free Energies= -538.566873
0 1

C	0.89876800	-0.54806500	-0.00001400
C	-0.48734900	-0.83386700	-0.00000300
C	1.32179300	0.81539200	-0.00004200
C	0.33404500	1.83525000	-0.00006500
C	-0.99703400	1.52698100	-0.00002800
C	-1.42824500	0.17333400	0.00000900
C	1.87862700	-1.57225800	0.00002800
C	3.21493300	-1.26385900	0.00004800
C	3.63274700	0.08514000	0.00002600
C	2.70820600	1.09960800	-0.00002100
C	-2.89935500	-0.10150300	0.00005200
O	-3.69774500	0.81628400	0.00014700
C	-3.38417100	-1.53838700	-0.00009400
H	-0.79184000	-1.87345800	-0.00002300
H	0.65428600	2.87132200	-0.00012100
H	-1.75366400	2.30038300	-0.00002700
H	1.55470200	-2.60722600	0.00004900
H	3.95577800	-2.05428600	0.00008200
H	4.69126600	0.31658600	0.00005300
H	3.03064800	2.13481700	-0.00003400
H	-3.02275900	-2.07361100	-0.88181800
H	-3.02229200	-2.07407300	0.88113700
H	-4.47195100	-1.53732900	0.00015000

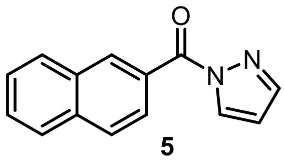

5

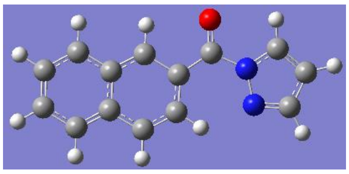

T1 in DCM

Zero-point correction= 0.203372 (Hartree/Particle)
Thermal correction to Energy= 0.216409
Thermal correction to Enthalpy= 0.217353
Thermal correction to Gibbs Free Energy= 0.161665
Sum of electronic and zero-point Energies= -724.167016
Sum of electronic and thermal Energies= -724.153979
Sum of electronic and thermal Enthalpies= -724.153035
Sum of electronic and thermal Free Energies= -724.208723
0 3

C	-9.37699007	-1.06470522	-1.28941340
C	-9.45872696	-1.66045972	-0.09120218
C	-8.41486025	-1.60895746	0.74984888

C	-7.28646529	-0.96505255	0.39986055
C	-7.20045596	-0.36473455	-0.80263605
C	-8.25316156	-0.42104531	-1.64035592
C	-6.23397717	-0.90107722	1.23204413
C	-5.11224418	-0.25622556	0.87590573
C	-4.99962600	0.34240400	-0.32586300
C	-6.06876464	0.27915883	-1.14638387
C	-3.85854200	1.00547500	-0.69892100
O	-3.78163800	1.55556600	-1.78379500
N	-2.83537200	1.08677900	0.05549000
C	-2.17170100	2.14283800	0.31682800
C	-1.10305300	1.78657900	1.03789200
C	-1.22027800	0.45621700	1.11623700
N	-2.26343900	0.08150400	0.50632000
H	-10.23299283	-1.10414955	-1.98390634
H	-10.38118898	-2.18981346	0.20082709
H	-8.50347995	-2.10491514	1.73152266
H	-8.21576190	0.05999317	-2.63282360
H	-6.27920510	-1.37286075	2.22908507
H	-4.30635112	-0.23155041	1.62650329
H	-6.04220328	0.75520956	-2.14066674
H	-2.46341100	3.14482100	-0.02037700
H	-0.31249800	2.42644700	1.44536700
H	-0.53299200	-0.23673300	1.61770000

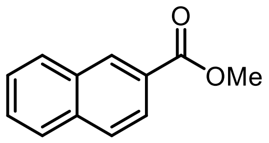

1

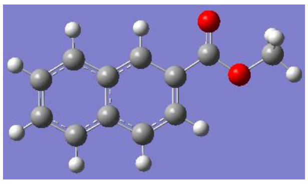

T1 in DCM

Zero-point correction= 0.184517 (Hartree/Particle)
Thermal correction to Energy= 0.196519
Thermal correction to Enthalpy= 0.197463
Thermal correction to Gibbs Free Energy= 0.145070
Sum of electronic and zero-point Energies= -613.690455
Sum of electronic and thermal Energies= -613.678453
Sum of electronic and thermal Enthalpies= -613.677509
Sum of electronic and thermal Free Energies= -613.729903

0 3

C	-1.37099600	-0.57627200	0.00000000
C	-0.02586700	-0.94899000	-0.00000100
C	-1.71392200	0.82600400	0.00000000
C	-0.65227700	1.77960700	0.00000000

C	0.69884200	1.38263300	-0.00000100
C	1.04031800	0.05269200	-0.00000100
C	2.42825400	-0.41947400	-0.00000100
O	2.74568900	-1.59346000	0.00000000
C	-2.42691900	-1.51644300	0.00000000
C	-3.78577100	-1.10210800	0.00000000
C	-4.10363600	0.22546800	0.00000000
C	-3.04974400	1.20158200	0.00000000
O	3.34008900	0.58693600	0.00000000
C	4.71285600	0.17031600	0.00000100
H	0.25981500	-1.99206300	-0.00000100
H	-0.89853300	2.83486000	0.00000000
H	1.47603200	2.13503400	-0.00000100
H	-2.18852100	-2.57329200	0.00000000
H	-4.56654700	-1.85318900	0.00000100
H	-5.13523500	0.55267800	0.00000100
H	-3.30310900	2.25577600	0.00000000
H	5.29562100	1.08840800	-0.00000100
H	4.93371300	-0.42304900	0.88739000
H	4.93371300	-0.42305300	-0.88738500

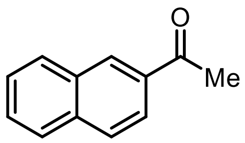

54

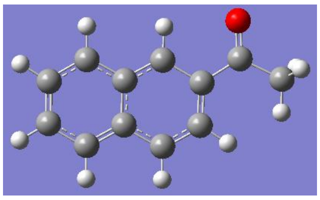

T1 in DCM

Zero-point correction=	0.179381 (Hartree/Particle)
Thermal correction to Energy=	0.189621
Thermal correction to Enthalpy=	0.190565
Thermal correction to Gibbs Free Energy=	0.142311
Sum of electronic and zero-point Energies=	-538.442960
Sum of electronic and thermal Energies=	-538.432720
Sum of electronic and thermal Enthalpies=	-538.431776
Sum of electronic and thermal Free Energies=	-538.480030

0 3

C	-0.95374700	-0.57066300	0.00000100
C	0.39602300	-0.93132200	0.00000900
C	-1.31114700	0.82502300	0.00000400
C	-0.25027000	1.79051200	0.00001200
C	1.09467200	1.41013500	0.00001400
C	1.46841700	0.07796700	0.00001500
C	-1.99996400	-1.52013700	-0.00000600
C	-3.35475200	-1.11890600	-0.00001200

C	-3.68556400	0.21112800	-0.00001100
C	-2.64495900	1.19130400	-0.00000200
C	2.85086000	-0.39705400	0.00005300
O	3.07532100	-1.60990200	0.00000200
C	4.02777900	0.56103600	-0.00005300
H	0.69435600	-1.97038400	0.00001200
H	-0.50779800	2.84342100	0.00001300
H	1.84517300	2.18911900	0.00002600
H	-1.75125000	-2.57471400	-0.00000700
H	-4.13077900	-1.87501700	-0.00001900
H	-4.72061500	0.52740400	-0.00001600
H	-2.90644300	2.24378900	-0.00000100
H	3.76568200	1.61707600	-0.00063600
H	4.64224800	0.35261400	0.87867300
H	4.64277300	0.35176000	-0.87820200

5. Experimental Procedures

5.1 Photocatalyst synthesis

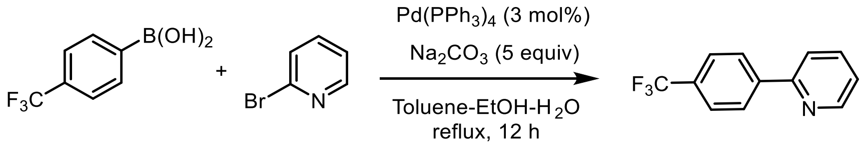

An oven-dried 250 mL flask equipped with a stir bar was cooled under vacuum. After evacuated/backfilled with N_2 (x 3) and capped with a septum, Na_2CO_3 (5.3 g, 50 mmol, 5.0 eq.), $Pd(PPh_3)_4$ (345 mg, 0.300 mmol, 0.0300 eq.), (4-(trifluoromethyl)phenyl)boronic acid (2.5 g, 13 mmol, 1.3 eq.) were added. Under N_2 atmosphere, 2-bromopyridine (1.60 g, 10.0 mmol, 1.00 eq.) in toluene (35 mL), ethanol (7.5 mL) and H_2O (35 mL) were added. The reaction mixture was refluxed in an oil bath for 12 h and then cooled to room temperature. The reaction mixture was quenched with aqueous sat. NH_4Cl (30 mL). The aqueous layer was extracted with EtOAc (3 x 30 mL). The combined organic layers were washed with brine (40 mL), dried over anhydrous Na_2SO_4, filtered and concentrated by rotary evaporation. The residue was purified by FCC (Hex:EtOAc = 19:1 to 8:1) to affard the aryl pyridines product (1.9 g, 86% yield). Spectral data matched that reported in the literature[10].

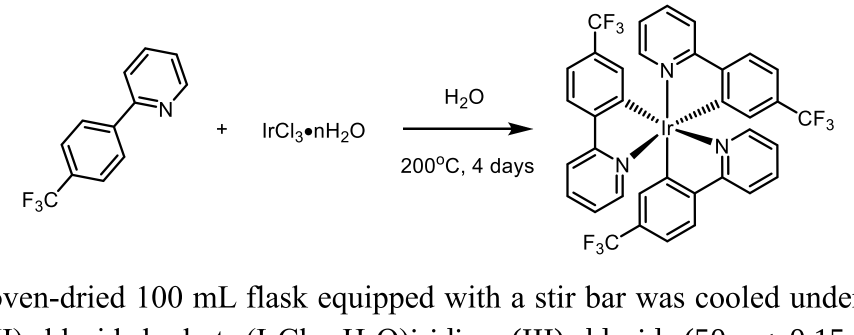

An oven-dried 100 mL flask equipped with a stir bar was cooled under vacuum, Iridium(III) chloride hydrate ($IrCl_3 \cdot nH_2O$)iridium (III) chloride (50 mg, 0.15 mmol, 1.0

eq.), 2-(4-(trifluoromethyl) phenyl) pyridine (448 mg, 2.00 mmol, 13.3 eq.) and distilled water (50 mL) were added, and then depressurized with N_2 and finally charged again with N_2 before sealing. The reaction mixture was heated to 200 °C in sand bath for 4 days. After cooled to room temperature, the reaction mixture was quenched with aqueous sat. NH_4Cl (20 mL). The aqueous layer was extracted with EtOAc (3 x 30 mL). The combined organic layers were washed with brine (20 mL), dried over anhydrous Na_2SO_4, filtered and concentrated by rotary evaporation. The residue was purified by FCC (Hex/EtOAc=5:1 to 1:1) to affard the corresponding product as a yellow solid (81 mg, 63% yield). Spectral data matched that reported in the literature[11].

5.2 Alkene synthesis:

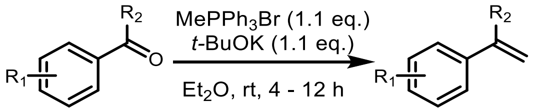

General Wittig reaction procedure for styrene synthesis: An oven-dried 100 mL flask equipped with a stir bar was cooled under vacuum. After evacuated/backfilled with N_2 (x 3) and capped with a septum, *t*-BuOK (1.23 g, 11.0 mmol, 1.10 eq.) and THF (50 mL) were added. The suspension was placed in a room temperature ice/water bath and $MePPh_3Br$ (3.93 g, 11.0 mL, 1.10 eq.) was added. The mixture was stirred at the same temperature for 0.5 h before aldehyde or ketone (10 mmol, 1.0 eq.) was added. The reaction was stirred for 4 - 12 h (monitored by TLC) and then concentrated by rotary evaporation. The crude was precipitated by the addition of pentane (50 mL) and sonication. The resulting suspension was filtered through a pad of silica gel and flushed with Hex:Et_2O (ratio based on TLC) to afford the corresponding styrene.

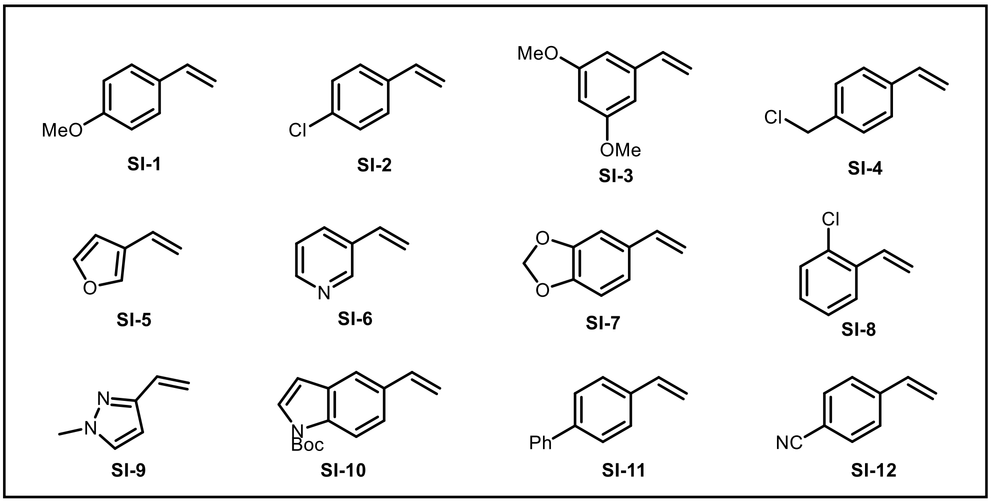

Alkenes **SI-1**[12], **SI-2**[12], **SI-3**[13], **SI-5**[14], **SI-6**[15], **SI-7**[16], **SI-9**[12], **SI-11**[17], **SI-12**[18] were prepared according to the general procedure. Alkenes **SI-4**[19], **SI-8**[20] and **SI-10**[16] were prepared according to known literature. All spectral data matched that reported in the literature.

5.3 Acyl pyrazole synthesis

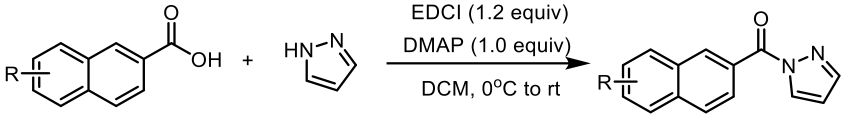

An oven-dried 250 mL flask equipped with a stir bar was cooled under vacuum. After evacuated/backfilled with N_2 (x 3) and capped with a septum. Different naphthalene carboxylic acid (5.0 mmol, 1.0 eq.), pyrazole (375 mg, 5.50 mmol, 1.10 eq.) and DMAP (610 mg, 5.00 mmol, 1.00 eq.) and anhydrous CH_2Cl_2 (50 mL) were added to the flask. Under N_2 atmosphere, EDCI (1.2 g, 6.0 mmol, 1.2 eq.) was added at 0°C. After the reaction was stirred at room temperature for 12 h, the reaction was diluted with dichloromethane, washed with 1N $NaHCO_3$, dried over Na_2SO_4, and evaporated in vacuum to afford the crude product, which was purified by flash chromatography on silica gel with hexanes/EtOAc to afford the corresponding acyl give acyl.

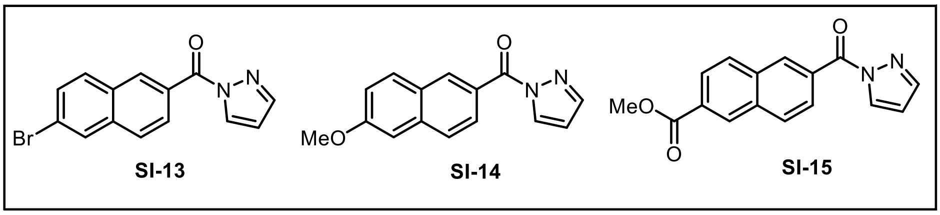

SI-13 SI-14 SI-15

SI-13, **SI-14** and **SI-15** were prepared according to above procedure. All spectral data matched that reported in the literature [21].

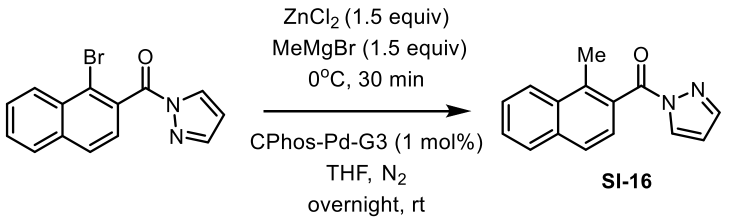

SI-16

(1-methylnaphthalen-2-yl)(1*H*-pyrazol-1-yl)methanone (SI-16): An oven-dried 20mL flask equipped with a stir bar was cooled under vacuum. After evacuated/backfilled with N_2 (x 3), containing anhydrous $ZnCl_2$ (408 mg, 3.00 mmol, 1.50 eq.) and distilled THF (5 mL), MeMgBr (1.0 mL, 3.0 M in Et_2O, 1.5 eq.) was added dropwise in an ice/water bath. After stirred at the same terperature for 30 min, the THF (10 mL) solution of (1-bromonaphthalen-2-yl)(1*H*-pyrazol-1-yl)methanone (602 mg, 2.00 mmol, 1.00 eq) and CPhos-Pd-G3 (16 mg, 0.020 mmol, 0.010 eq.) was added via syringe. The ice bath was removed and the mixture was stirred overnight. The reaction was quenched with sat. NH_4Cl (aq.) (5 mL), extracted with EtOAc (3 × 10 mL). The combined organic layers were washed with brine (10 mL), dried over with anhydrous Na_2SO_4, filtered and concentrated by rotary evaporation. The residue was purified by FCC (Hex: EtOAc = 100:1 to 10:1) to afford (1-methylnaphthalen-2-yl)(1*H*-pyrazol-1-yl)methanone (175 mg, 37% yield) as a white soild [22].

^{1}H NMR (500 MHz, $CDCl_3$) δ 8.42 (d, *J* = 2.9 Hz, 1H), 8.13 (dd, *J* = 8.2, 1.5 Hz, 1H), 7.91 – 7.87 (m, 1H), 7.80 (d, *J* = 8.5 Hz, 1H), 7.76 (d, *J* = 1.5 Hz, 1H), 7.63 – 7.56 (m, 2H), 7.51 (d, *J* = 8.5 Hz, 1H), 6.54 (dd, *J* = 2.9, 1.5 Hz, 1H), 2.68 (s, 3H).

^{13}C NMR (126 MHz, CDCl$_3$) δ 168.9, 145.1, 134.5, 134.4, 132.5, 130.3, 129.8, 128.8, 127.4, 126.9, 126.2, 125.0, 124.8, 110.1, 16.5.
HRMS (ESI, *m/z*): Calcd for $C_{15}H_{12}ON_2Na^+$ [M+H]$^+$: 259.0842, found: 259.0842.
IR: 2926 (s), 1715 (s), 1379 (s), 1342 (s), 1244 (s).

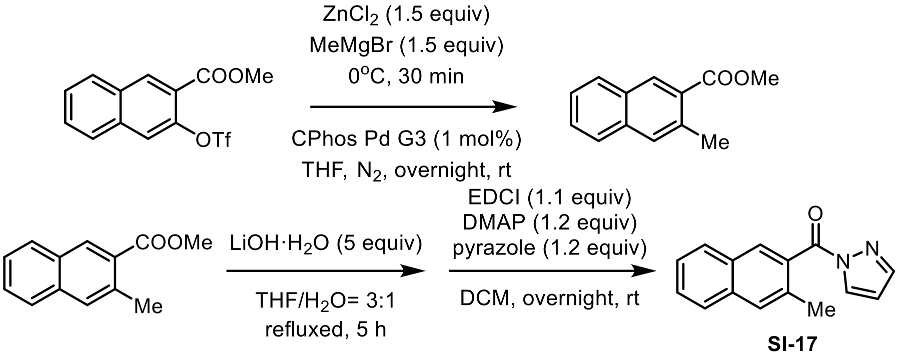

SI-17

(3-methylnaphthalen-2-yl)(1*H*-pyrazol-1-yl)methanone (SI-17): An oven-dried 100 mL flask equipped with a stir bar was cooled under vacuum. After evacuated/backfilled with N_2 (x 3), containing anhydrous $ZnCl_2$ (408 mg, 3.00 mmol, 1.50 eq.) and distilled THF (5 mL), MeMgBr (1.0 mL, 3.0 M in Et_2O, 1.5 eq.) was added dropwise in an ice/water bath. After stirred at the same terperature for 30 min, the THF solution of methyl 3-(((trifluoromethyl)sulfonyl)oxy)-2-naphthoate (669 mg, 2.00 mmol, 1.00 eq.) and CPhos-Pd-G3 (16 mg, 0.020 mmol, 0.010 eq.) was added dropwise via syringe. The ice/water bath was removed and the mixture was stirred overnight. The reaction was quenched with sat. NH_4Cl (aq) (5 mL), extracted with EtOAc (3 × 10 mL). The combined organic layers were washed with brine (10 mL), dried over with anhydrous Na_2SO_4, filtered and concentrated by rotary evaporation. The residue was purified by FCC (Hex: EtOAc = 30:1 to 10:1) to afford methyl 3-methyl-2-naphthoate (698 mg, 81% yield) as a white soild.

An oven-dried 100 mL flask equipped with a stir bar was cooled under vacuum, the LiOH·H_2O (336 mg, 8.00 mmol, 5.00 eq.) was added to the flask, under N_2 atmosphere, containing the THF/H_2O (3:1, v/v, 0.4 M) solution of methyl 3-methyl-2-naphthoate was add. And the mixture was refluxed in an oil bath for 5 hours. After cooled down to room temperature, the mixture was diluted with sat. NH_4Cl (aq) (20 mL) and extracted with EtOAc (3 × 20 mL). The combined organic layer was dried over with anhydrous Na_2SO_4, filtered and concentrated by rotary evaporation. The residue was dissolved in DCM (50 mL), DMAP(235 mg, 1.92 mmol, 1.20 eq.), and pyrazole (131 mg, 1.92 mmol, 1.20 eq.) and EDCI (338 mg, 1.67 mmol, 1.10 eq.) were added into the solution. The mixture was stirred overnight, quenched with sat. NH_4Cl (aq.) (20 mL), extracted with DCM (3 × 20 mL). The combined organic layers were washed with brine (25 mL), dried over with anhydrous Na_2SO_4, filtered and concentrated by rotary evaporation. The residue was purified by FCC (Hex: EtOAc = 30:1 to 20:1) to afford (3-methylnaphthalen-2-yl)(1*H*-pyrazol-1-yl)methanone (268 mg, 71% yield) as a white soild. Spectral data matched that reported in the literature[22].
^{1}H NMR (500 MHz, CDCl$_3$) δ 8.43 (d, *J* = 2.9 Hz, 1H), 8.06 (s, 1H), 7.85 (d, *J* = 7.2 Hz, 1H), 7.82 – 7.76 (m, 2H), 7.74 (s, 1H), 7.57 – 7.52 (m, 1H), 7.47 (td, *J* = 7.5, 1.3 Hz, 1H), 6.55 (dd, *J* = 2.9, 1.5 Hz, 1H), 2.50 (s, 3H).

^{13}C NMR (126 MHz, CDCl$_3$) δ 168.1, 145.1, 134.8, 133.3, 131.3, 130.7, 130.1, 129.9, 129.1, 128.6, 128.1, 127.3, 126.2, 110.1, 20.1.
HRMS (ESI, *m/z*): Calcd for $C_{15}H_{12}ON_2Na^+$ [M+Na]$^+$: 259.0842, found: 259.0843.
IR: 2924 (m), 1708 (s), 1379 (s), 1341 (s), 1201 (s).

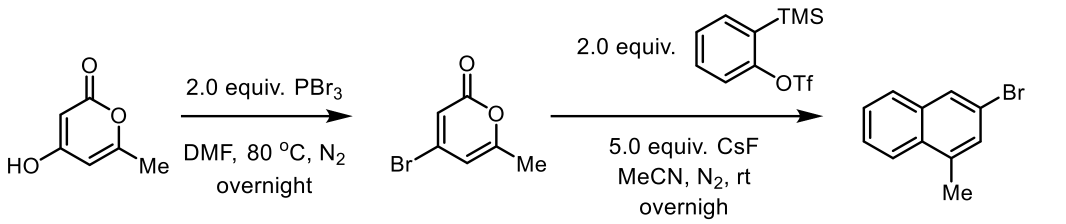

To an oven-dried 100 ml flask equipped with a stir bar containing 4-bromo-6-methyl-2H-pyran-2-one (500 mg, 2.65 mmol, 1.00 equiv.) and 2-(trimethylsilyl)phenyl trifluoromethanesulfonate (1.58 g, 5.30 mmol, 2.00 equiv.), the dry MeCN (50 ml) and anhydrous CsF (2.01 g, 13.2 mmol, 5.00 equiv.) were added successively under nitrogen atmosphere. Then the reaction was purged with nitrogen for ten minutes before being sealed with a sleeve stopper and left to stir at 23 °C overnight. The reaction mixture was filtered through a short pad of celite with EtOAc (10 mL) as the eluent and then concentrated under reduced pressure. The crude product was further purified by flash column chromatography on silica gel (Hex:EtOAc = 20:1) to afford 3-bromo-1-methylnaphthalene as a light yellow oil (392 mg, 2.65 mmol, 67% yield).

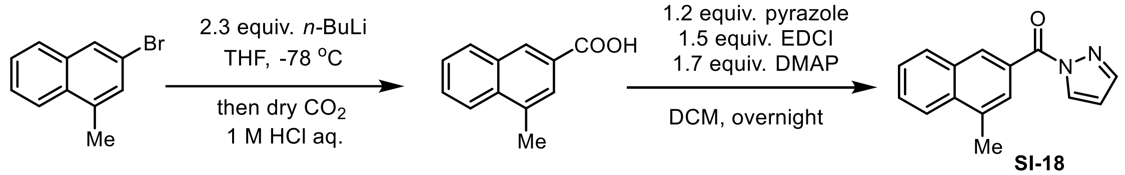

SI-18

(4-methylnaphthalen-2-yl)(1H-pyrazol-1-yl)methanone (SI-18): To an oven-dried 50 ml flask equipped with a stir bar containing 3-bromo-1-methylnaphthalene (390 mg, 2.65 mmol, 1.00 equiv.), the dry THF (25 ml) was added. Then the reaction was cooled to -78 °C (dry ice/acetone) and stirred for 15 min. The *n*-BuLi (2.0 M in Hexane) (3.00 ml, 6.00 mmol, 2.26 equiv.) was added dropwise by using a syringe pump in 30 min. The reaction was stirred for two more hours at -78 °C and purged with dry CO_2 (produced by dry ice and dried by anhydrous $CaSO_4$ dry tube) for 30 min. The reaction was warmed up to room temperature, quenched with 1 M HCl, extracted with EtOAc (20 ml × 3). The organic phase was combined, dried with anhydrous Na_2SO_4, concentrated under reduced pressure. Then to the 100 ml flask equipped with a stir bar containing crude product, pyrazole (245mg, 3.60 mmol, 1.20 equiv.), EDCI (762 mg, 4.50 mmol, 1.50 equiv.), DMAP (623 mg, 5.10 mmol, 1.70 equiv.) and DCM (50 ml) were added successively. The mixture was stirred overnight at room temperature. Upon completion (monitored by TLC), the reaction was quenched with distilled water (50 ml), extracted with DCM (50 ml × 3). The organic phase was combined, dried by anhydrous Na_2SO_4 and concentrated under reduced pressure. The crude product was further purified by flash column chromatography (Hex:EtOAc =

100:1) to afford the (4-methylnaphthalen-2-yl)(1*H*-pyrazol-1-yl)methanone (**SI-18**) (90 mg, 0.40 mmol, 14% yield).

^{1}H NMR (500 MHz, Chloroform-*d*) δ 8.63 – 8.60 (m, 1H), 8.49 (dd, *J* = 2.7, 0.7 Hz, 1H), 8.05 (d, *J* = 7.4 Hz, 1H), 7.99 (d, *J* = 8.1 Hz, 1H), 7.95 (s, 1H), 7.85 (s, 0H), 7.70 – 7.62 (m, 1H), 7.61 – 7.54 (m, 1H), 6.56 (dd, *J* = 2.9, 1.5 Hz, 1H), 2.76 (s, 3H) ppm.

^{13}C NMR (126 MHz, Chloroform-*d*) δ 166.8, 144.6, 134.9, 134.8, 132.5, 132.4, 130.7, 130.5, 128.7, 128.4, 127.1, 126.6, 124.2, 109.5, 19.6 ppm.

HRMS (ESI, *m*/*z*): Calcd for $C_{15}H_{12}ON_2Na^+$ [M+Na]$^+$: 259.0842, found: 259.0843.

IR: 2348 (m), 2314 (s), 2183 (s), 1347 (s), 1210 (s).

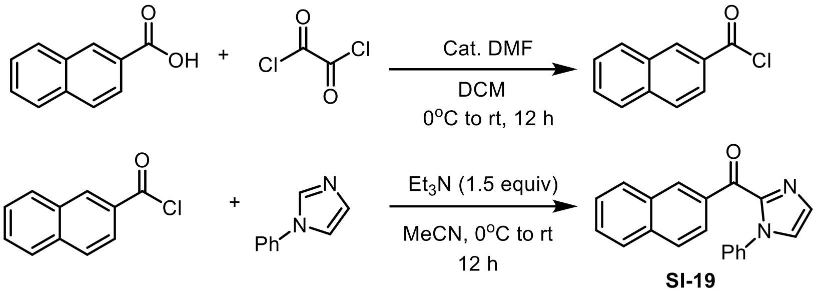

Naphthalen-2-yl(1-phenyl-1H-imidazol-2-yl)methanone (SI-19): An oven-dried 50 mL flask equipped with a stir bar was cooled under vacuum. 2-Naphthoic acid (861 mg, 5.00 mmol, 1.00 eq.) was added to the flask. After evacuated/backfilled with N_2 (x 3), DCM (8 mL) and catalytic amount of DMF were added to this solution. Then oxalyl chloride (424 μL, 5.00 mmol, 1.00 eq.) was added dropwise to this reaction mixture in an ice/water bath under nitrogen atmosphere with continuous stirring. After completion of addition, the reaction mixture was allowed to stir at room temperature for 12 h. Then oxalyl chloride was removed under reduced pressure. The residue of acyl chloride was used directly for next step without further purification[23].

An oven-dried 50 mL flask equipped with a stir bar was cooled under vacuum. 1-phenyl-1H-imidazole (288 mg, 2.00 mmol, 1.00 eq.) was added to the flask. After evacuated/backfilled with N_2 (x 3), CH_3CN (8 mL) was added, and then acyl chloride (570 mg, 3.00 mmol, 1.30 eq.) was added at 0 °C in an ice/water bath, followed by addition of Et_3N (420 μL, 3.00 mmol, 1.50 eq.). Then the reaction was stirred at room temperature for 12 h. The reaction was then quenched with H_2O (50 mL), and extracted with EtOAc (3 × 20 mL). The combined organic layers were collected and dried over anhydrous Na_2SO_4, filtered and concentrated by rotary evaporation. The residue was purified by FCC (Hex: EtOAc = 20:1 to 8:1) to afford the product (429 mg, 72% yield) as a white soild. Spectral data matched that reported in the literature[24].

5.4 Naphthyl ketone synthesis

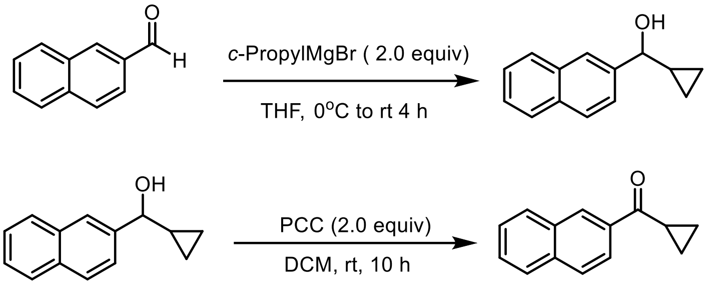

SI-20

Cyclopropyl(naphthalen-2-yl)methanone (SI-20): An oven-dried 250 mL flask equipped with a stir bar was cooled under vacuum. 2-Naphthaldehyde (2.76 g, 10.0 mmol, 1.00 eq.) was added to the flask.After evacuated/backfilled with N_2 (x 3), THF (40 mL) was addedand 1.0 M *c*-PropylMgBr (20 mL, 20 mmol, 2.0 eq.) was added at 0 °C over 30 min in ice/water bath. The reaction mixture was allowed to warm to room temperature and stirred for 4 h. Afterwards, the reaction was quenched with sat. NH_4Cl (40 mL), and extracted with EtOAc (3 × 30 mL). The combined organic layers were collected, dried over anhydrous Na_2SO_4, filtered and concentrated by rotary evaporation to afford cyclopropyl(naphthalen-2-yl)methanol as a white solid (1.8 g, 90% yield), which was used for next step without further purification[25].

An oven-dried 250 mL flask equipped with a stir bar was cooled under vacuum. Cyclopropyl(naphthalen-2-yl)methanol (0.99 g, 5.0 mmol, 1.0 eq.) was added to the flask After evacuated/backfilled with N_2 (x 3), dry DCM (20 mL) was added, and then cooled to 0 °C in an ice/water bath.PCC (3.7 g, 10 mmol, 2.0 eq.) was added and the reaction was stirred for 10 h at room temperature. Afterwards, the reaction was quenched with sat. NH_4Cl (20 mL) aqueous solution, and extracted with EtOAc (3 × 50 mL). The organic layers were collected, dried over anhydrous Na_2SO_4, filtered and concentrated by rotary evaporation. The residue was purified by FCC (Hex: EtOAc = 20:1 to 10:1) to afford the product (813 mg, 83%) as a white solid. Spectral data matched that reported in the literature[26].

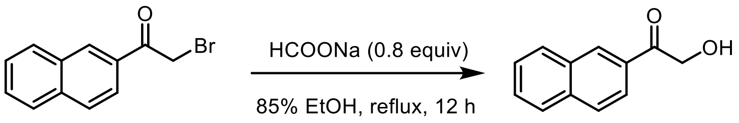

SI-21

2-hydroxy-1-(naphthalen-2-yl)ethan-1-one (SI-21): An oven-dried 100 mL flask equipped with a stir bar was cooled under vacuum. 2-Bromo-1-(naphthalen-2-yl)ethan-1-one (2.48 g, 10.0 mmol, 1.00 eq.) and sodium formate (6 g, 8 mmol, 0.8 eq.) were added to the flask. After evacuated/backfilled with N_2 (x 3), 85% ethanol (40 mL) was added, then the reaction was refluxed in an oil bath for 12 h. Afterwards, the reaction was quenched with sat. NH_4Cl (20 mL) aqueous solution, and extracted with EtOAc (3 × 20 mL). The combined organic layers were collected and dried over anhydrous Na_2SO_4, filtered and concentrated by rotary evaporation. The residue was purified by FCC (Hex: EtOAc = 10:1 to 5:1) to afford the product (1.6 g, 86% yield) as a white solid.Spectral data matched that reported in the literature[27].

5.5 Photochemical cycloaddition

General procedure

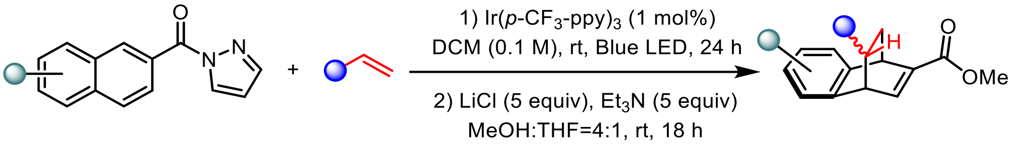

An oven-dried 4 mL screw-cap vial equipped with a stir bar and a septum was cooled under vacuum. After evacuated/backfilled with N_2 (x 3), the vial was transferred into glovebox and charged with Ir(*p*-CF_3-ppy)$_3$ (1.6 mg, 1.0 mol%) and acyl pyrazole (0.20 mmol, 1.0 eq.). After moved out, alkene (2.0 mmol, 10 eq.) and dry DCM (2 mL) were sequentially added to the vial. Then the septum was replaced with a cap. The vial was capped and the reaction was stirred under blue LED irradiation with a cooling fan to keep room temperature (see following pictures for set up). After 24 h, the solution was concentrated by rotary evaporation. 1,1,2,2-Tetrachlorethane (21 μL, 0.20 mmol) was added to the residue for crude ^{1}H-NMR to determine d.r.. Afterwards, the crude product was transformed into the corresponding methyl ester through the following procedure: The crude mixture was transferred into a 10 mL round bottom flask and dissolved in anhydrous methanol (4 mL) and THF (1 mL). To this solution was added LiCl (42 mg, 1.0 mmol, 5.0 eq.) and Et_3N (140 μL, 1.00 mmol, 5.00 eq.) at room temperature. After 18 hours of stirring, the reaction mixture was diluted with CH_2Cl_2. The combined organic solution was concentrated under reduced pressure and the crude was purified by FCC to give corresponding cycloadducts.

(***Note:*** *Some products were isolatedwithout transformation to the corresponding methyl ester*)

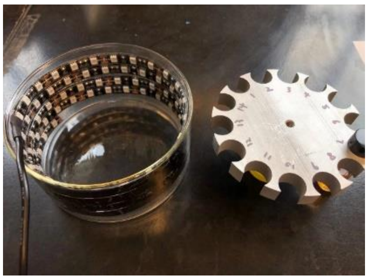

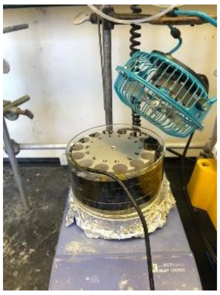

Figure S5. Reaction devices

5.6 NMR Spectra datas

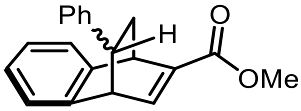

7

The title compound was prepared according to the general procedure using naphthalen-2-yl(1H-pyrazol-1-yl)methanone (44.4 mg, 0.200 mmol, 1.00 eq.), styrene (0.23 mL, 2.0 mmol, 10 eq.) and *fac*-Ir(*p*-CF_3-ppy)$_3$ (1.7 mg, 2.0 mol%) in DCM (2.0 mL, 0.1 M) under nitrogen atmosphere with blue LEDs irridiation for 24 hoursThe d.r. value was determined through ^{1}H NMR of crude materials as 1.4:1. Afterwards, the crude product was transformed into the corresponding methyl ester andthe crude was purified by FCC (Hex:EtOAc = 30:1 to 10:1) to afford **7** (47.5 mg, 82% total yield;

minor: 15.8 mg, 27.0% yield, major: 31.7 mg, 55.0% yield) as two separable diastereoisomers as a colorless solid.

Note: The yield was reported as an average of two runs.

The minor diastereoisomer:

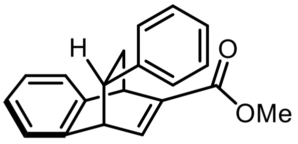

7a

Methyl *(1S,4R,10S)*-10-phenyl-1,4-dihydro-1,4-ethanonaphthalene-3-carboxylate (7a):

^{1}H NMR (500 MHz, Chloroform-*d*) δ 7.34 (d, *J* = 6.3 Hz, 1H), 7.24 – 7.12 (m, 5H), 7.07 – 7.06 (m, 4H), 4.57 (s, 1H), 4.11 (d, *J* = 6.3 Hz, 1H), 3.71 (s, 3H), 3.02-2.99 (m, 1H), 2.13-2.08 (m, 1H), 1.76-1.72 (m, 1H).

^{13}C NMR (126 MHz, Chloroform-*d*) δ 165.3, 144.8, 144.3, 143.5, 142.9, 141.1, 128.6, 127.9, 126.6, 126.0, 125.8, 123.8, 123.0, 51.9, 48.9, 45.5, 40.8, 35.6 ppm.

HRMS (ESI, *m*/*z*): Calcd for $C_{20}H_{18}O_2Na^+$ $[M+Na]^+$: 313.1199, found: 313.1197.

IR: 2949 (s), 1712 (s), 1621 (s), 1282 (s), 1074 (s).

The major diastereoisomer:

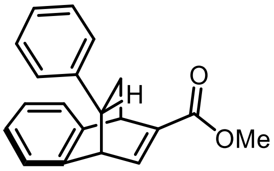

7b

Methyl *(1S,4R,10R)*-10-phenyl-1,4-dihydro-1,4-ethanonaphthalene-3-carboxylate (7b):

^{1}H NMR (500 MHz, Chloroform-*d*) δ 7.65 (d, *J* = 6.5 Hz, 1H), 7.37 (d, *J* = 7.3 Hz, 1H), 7.20 (t, *J* = 7.5 Hz, 1H), 7.09 – 7.04 (m, 4H), 6.85 (d, *J* = 7.3 Hz, 1H), 6.46 (d, *J* = 6.8 Hz, 2H), 4.65 (s, 1H), 3.94 (d, *J* = 6.6 Hz, 1H), 3.78 (s, 3H), 3.17-3.14 (m, 1H), 2.23-2.18 (m, 1H), 1.62 – 1.59 (m, 1H).

^{13}C NMR (126 MHz, Chloroform-*d*) δ 165.6, 147.1, 144.1, 143.6, 139.5 138.7, 128.1, 126.4, 126.24, 126.22, 125.5, 122.9, 51.8, 49.5, 43.8, 40.4, 34.6 ppm.

HRMS (ESI, *m*/*z*): Calcd for $C_{20}H_{18}O_2Na^+$ $[M+Na]^+$: 313.1199, found: 313.1199.

IR: 2950 (m), 1712 (s), 1622 (s), 1280 (m), 1076 (s).

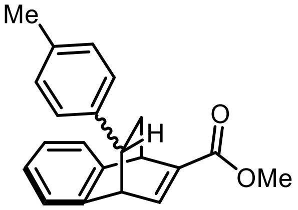

8

Methyl (1R,4S)-9-(p-tolyl)-1,4-dihydro-1,4-ethanonaphthalene-2-carboxylate (8): The title compound was prepared according to the general procedure using

naphthalen-2-yl(1H-pyrazol-1-yl)methanone (44.4 mg, 0.200 mmol, 1.00 eq.), 1-methyl-4-vinylbenzene (236 mg, 2.00 mmol, 10.0 eq.) and *fac*-Ir(*p*-CF_3-ppy)$_3$ (1.7 mg, 2.0 mol%) in DCM (2.0 mL, 0.10 M) under nitrogen atmosphere with blue LEDs irridiation for 24 hours. The d.r. value was determined through ^{1}H NMR of crude materials as 1.4:1. Afterwards, the crude product was transformed into the corresponding methyl ester, the crude was purified by FCC (Hex:EtOAc = 20:1 to 10:1) to afford **8** (49.2 mg, 81% total yield) as a mixture of two diastereosiomers as a colorless oil.

Note: The yield was reported as an average of two runs.

^{1}H NMR (500 MHz, Chloroform-*d*) δ 7.66 (d, *J* = 6.2 Hz, 1H, major), 7.43 (d, *J* = 6.3 Hz, 1H, minor), 7.37 (d, *J* = 7.3 Hz, 1H, major), 7.33 – 7.31 (m, 1H, minor), 7.25 – 7.04 (m, 9H, mix), 6.91-6.87 (m, 3H, mix), 6.36 (d, *J* = 6.2 Hz, 2H, major), 4.65 (s, 2H, mix), 4.18 (d, *J* = 6.2 Hz, 1H, minor), 3.93 (d, *J* = 6.5 Hz, 1H, major), 3.80 (s, 3H, minor), 3.79 (s, 3H, major), 3.14-3.11 (m, 1H, major), 3.08-3.05 (m, 1H, minor), 2.34 (s, 3H, minor), 2.25 (s, 3H, major), 2.23-2.16 (m, 2H, mix), 1.84-1.79 (m, 1H, minor), 1.60-1.56 (m, 1H, major).

^{13}C NMR (126 MHz, Chloroform-*d*) δ 165.6(major), 165.3(minor), 147.2(major), 144.4(minor), 144.2(major), 143.5(minor), 142.9(minor), 141.7(minor), 140.9(minor), 140.5(major), 139.4(major), 138.8(major), 136.1(minor), 135.9(major), 129.3(minor), 128.8(major), 127.9(major), 127.8(minor), 126.23(major), 126.16(major), 125.9(minor), 125.8(minor), 125.5(major), 123.8(minor), 123.0(minor), 122.9(major), 51.9(minor), 51.8(major), 49.6(major), 49.0(minor), 45.0(minor), 43.4(major), 40.8(minor), 40.4(major), 35.6(minor), 34.7(major), 21.1(minor), 21.0(major) ppm.

HRMS (APCI, *m*/*z*): Calcd for $C_{21}H_{21}O_2^+$ [M+H]$^+$: 305.1536, found: 305.1531.

IR is not collected due to product is a mixture.

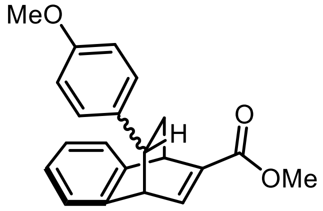

9

Methyl (1R,4S)-9-(4-methoxyphenyl)-1,4-dihydro-1,4-ethanonaphthalene-2-carboxylate (9): The title compound was prepared according to the general procedure using naphthalen-2-yl(1H-pyrazol-1-yl)methanone (44.4 mg, 0.200 mmol, 1.00 eq.), 1-methoxy-4-vinylbenzene (268 mg, 2.00 mmol, 10.0 eq.) and *fac*-Ir(*p*-CF_3-ppy)$_3$ (1.7 mg, 2.0 mol%) in DCM (2.0 mL, 0.10 M) under nitrogen atmosphere with blue LEDs irridiation for 24 hours. The d.r. value was determined through ^{1}H NMR of crude materials as 1.6:1. Afterwards, the crude product was transformed into the corresponding methyl ester, the crude was purified by FCC (Hex:EtOAc = 15:1 to 10:1) to afford **9** (50.6 mg, 79% total yield) as a mixture of two diastereosiomers as a colorless oil.

Note: The yield was reported as an average of two runs.

^{1}H NMR (500 MHz, Chloroform-*d*) δ 7.55 (d, *J* = 6.4 Hz, 1H, major), 7.33 (d, *J* = 6.4

Hz, 1H, minor), 7.28 (d, *J* = 7.3 Hz, 1H, major), 7.23 – 7.21 (m, 1H, minor), 7.16– 7.10 (m, 2H, mix), 7.06-7.05 (m, 2H, mix), 6.99-6.96 (m, 3H, mix), 6.79 – 6.74 (m, 3H, mix), 6.54 (d, *J* = 9.1 Hz, 2H, major), 6.28 (d, *J* = 8.1 Hz, 2H, major), 4.54 (s, 2H, mix), 4.06 (d, *J* = 6.3 Hz, 1H, minor), 3.82 (d, *J* = 6.3 Hz, 1H, major), 3.71 (s, 6H, mix), 3.69 (s, 3H, minor), 3.64 (s, 3H, major), 3.04-3.01 (m, 1H, major), 2.97-2.94 (m, 1H, minor), 2.13-2.06 (m, 2H, mix), 1.71-1.67 (m, 1H, minor), 1.47-1.43 (m, 1H, major).
^{13}C NMR (126 MHz, Chloroform-*d*) δ 165.6(major), 165.3(minor), 158.3, 158.1(major), 147.1(major), 144.4(major), 144.1, 143.5, 142.7, 141.0, 139.4, 138.8, 136.8, 135.6, 129.0(major), 128.9(minor), 126.3(major), 126.2(major), 125.9(minor), 125.8(minor), 125.5(major), 123.8(minor), 123.0(minor), 122.9(major), 114.0(minor), 113.4(major), 55.4(minor), 55.3(major), 51.9(minor), 51.8(major), 49.8(major), 49.2(minor), 44.7(minor), 43.0(major), 40.8(minor), 40.4(major), 35.7(minor), 34.8(major) ppm.
HRMS (ESI, *m*/*z*): Calcd for $C_{21}H_{20}O_3Na^+$[M+Na]$^+$: 343.1305, found: 343.1306.
IR is not collected due to product is a mixture.

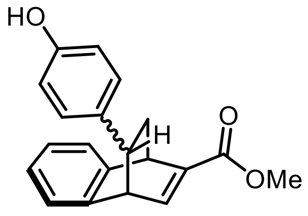

10

Methyl (1R,4S)-9-(4-hydroxyphenyl)-1,4-dihydro-1,4-ethanonaphthalene-2-carboxylate (10): The title compound was prepared according to the general procedure using naphthalen-2-yl(1H-pyrazol-1-yl)methanone (44.4 mg, 0.200 mmol, 1.00 eq.), 4-vinylphenyl acetate (324 mg, 2.00 mmol, 10.0 eq.) and *fac*-Ir(*p*-CF_3-ppy)$_3$ (1.7 mg, 2.0 mol%) in DCM (2.0 mL, 0.10 M) under nitrogen atmosphere with blue LEDs irridiation for 24 hours. The d.r. value was determined through ^{1}H NMR of crude materials as 1.2:1. Afterwards, the crude product was transformed into the corresponding methyl ester, the crude was purified by FCC (Hex:EtOAc = 20:1 to 8:1) to afford **10** (45 mg, 74% total yield) as a mixture of two diastereosiomers as a colorless oil.
Note: The yield was reported as an average of two runs. During hydrolysis, the acetyl group undergoes hydrolysis, and finally a hydroxyl group is formed.
^{1}H NMR (500 MHz, Chloroform-*d*) δ 7.65 (d, *J* = 6.2 Hz, 1H, major), 7.43 (d, *J* = 6.3 Hz, 1H, minor), 7.35 (d, *J* = 7.3 Hz, 1H, major), 7.30 – 7.29 (m, 1H, minor), 7.24 – 7.18 (m, 2H, mix), 7.15 – 7.13 (m, 2H, mix), 7.05 (t, *J* = 7.4 Hz, 1H, major), 7.00 (d, *J* = 8.5 Hz, 2H, mix), 6.85 (d, *J* = 7.3 Hz, 1H, major), 6.76 (d, *J* = 8.4 Hz, 2H, mix), 6.56 (d, *J* = 8.5 Hz, 2H, mix), 6.30 (d, *J* = 8.6 Hz, 2H, mix), 5.38 (s, 1H, minor), 5.12 (s, 1H, major), 4.62 (s, 2H, mix), 4.14 (d, *J* = 5.9 Hz, 1H, minor), 3.89 (d, *J* = 6.6 Hz, 1H, major), 3.80 (s, 3H, minor), 3.78 (s, 3H, major), 3.11-3.08 (m, 1H, major), 3.04 - 3.01(m, 1H, minor), 2.20 – 2.13 (m, 2H, mix), 1.78-1.74 (m, 1H, minor), 1.54 - 1.50 (m, 1H, major).
^{13}C NMR (126 MHz, Chloroform-*d*) δ 165.9(major), 165.7(minor), 154.4(minor), 154.2(major), 147.5(major), 144.9, 144.1, 143.4, 142.8, 140.9, 139.3, 138.7, 136.8,

135.6, 129.1(major), 129.0(minor), 126.3, 126.2, 125.9, 125.8, 125.5, 123.8, 123.0(minor), 122.9(major), 115.4(minor), 114.9(major), 52.1(minor), 51.9(major), 49.8(major), 49.3(minor), 44.6(minor), 42.9(major), 40.8(minor), 40.4(major), 35.7(minor), 34.7(major) ppm.
HRMS (APCI, *m*/*z*): Calcd for $C_{20}H_{19}O_3^+$ $[M+H]^+$: 307.1329, found: 307.1329.
IR is not collected due to product is a mixture.

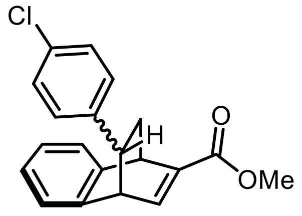

11

Methyl (1R,4S)-9-(4-chlorophenyl)-1,4-dihydro-1,4-ethanonaphthalene-2-carboxylate (11): The title compound was prepared according to the general procedure using naphthalen-2-yl(1H-pyrazol-1-yl)methanone (44.4 mg, 0.200 mmol, 1.00 eq.), 1-chloro-4-vinylbenzene (276 mg, 2.00 mmol, 10.0 eq.) and *fac*-Ir(p-CF_3-ppy)$_3$ (1.7 mg, 2.0 mol%) in DCM (2.0 mL, 0.10 M) under nitrogen atmosphere with blue LEDs irridiation for 24 hours. The d.r. value was determined through ^{1}H NMR of crude materials as 1.5:1. Afterwards, the crude product was transformed into the corresponding methyl ester, the crude was purified by FCC (Hex:EtOAc = 30:1 to 10:1) to afford **11** (46.7 mg, 72% total yield) as a mixture of two diastereosiomers as a colorless oil.
Note: The yield was reported as an average of two runs.
^{1}H NMR (500 MHz, Chloroform-*d*) δ 7.54 (d, *J* = 6.5 Hz, 1H, major), 7.28 (d, *J* = 7.6 Hz, 2H, mix), 7.24 – 7.11 (m, 4H, mix), 7.07 – 7.04 (m, 2H, mix), 7.00 – 6.95 (m, 4H, mix), 6.76 (d, *J* = 7.3 Hz, 2H, mix), 6.27 (d, *J* = 8.9 Hz, 3H, mix), 4.56 (s, 2H, mix), 4.06 (d, *J* = 6.3 Hz, 1H, minor), 3.81 (d, *J* = 6.1 Hz, 1H, major), 3.71 (s, 3H, minor), 3.70 (s, 3H, major), 3.06-3.03 (m, 1H, major), 2.99 – 2.96 (m, 1H, minor), 2.14-2.08 (m, 2H, mix), 1.70-1.66 (m, 1H, minor), 1.47-1.43 (m, 1H, major).
^{13}C NMR (126 MHz, Chloroform-*d*) δ 165.5(major), 165.2(minor), 146.7(major), 144.0(major), 143.9(minor), 143.2(minor), 143.1(minor), 142.8(minor), 142.1(major), 141.3, 139.7(major), 138.4(major), 132.2, 129.34(major), 129.25(minor), 128.7(minor), 128.6(minor), 128.2(major), 126.4(major), 126.3(major), 126.1(minor), 125.9(minor), 125.7(major), 123.9(minor), 123.03(minor), 122.99(major), 52.0(minor), 51.9(major), 49.4(major), 48.8(minor), 44.9(minor), 43.2(major), 40.7(minor), 40.3(major), 35.6(minor), 34.7(major) ppm.
HRMS (APCI, *m*/*z*): Calcd for $C_{20}H_{18}O_2Cl^+$$[M+H]^+$: 325.0990, found: 325.0989.
IR is not collected due to product is a mixture.

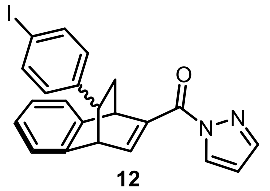

12

The title compound was prepared according to the general procedure using naphthalen-2-yl(1-phenyl-1H-imidazol-2-yl)methanone (59.6 mg, 0.200 mmol, 1.00 eq.), 1-iodo-4-vinylbenzene (460 mg, 2.00 mmol, 10.0 eq.) and *fac*-Ir(*p*-CF_3-ppy)$_3$ (1.7 mg, 2.0 mol%) in DCM (2.0 mL, 0.1 M) under nitrogen atmosphere with blue LEDs irridiation for 24 hours. The d.r. value was determined through ^{1}H NMR of crude materials as 1.2:1. Afterwards, the crude product was transformed into the corresponding methyl ester, the crude was purified by FCC (Hex:EtOAc = 20:1 to 8:1) to afford **12** (59 mg, 65% total yield; minor: 27 mg, 30% yield, major: 32 mg, 35% yield) as two separable diastereoisomers as a colorless oil.
Note: The yield was reported as an average of two runs.

The minor diastereoisomer:

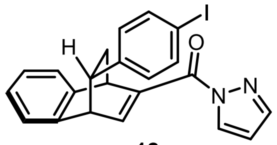

12a

***((1S,4R,10S)-10-*(*4*-iodophenyl)-*1,4*-dihydro-*1,4*-ethanonaphthalen-*3*-yl)(*1*H-pyrazol-*1*-yl)methanone (12a):**

^{1}H NMR (500 MHz, $CDCl_3$) δ 8.27 (dd, *J* = 2.9, 0.8 Hz, 1H), 7.84 (dd, *J* = 6.4, 1.9 Hz, 1H), 7.64 (s, 1H), 7.51 (d, *J* = 8.4 Hz, 2H), 7.26 – 7.24 (m, 1H), 7.20 – 7.19 (m, 1H), 7.10 – 7.07 (m, 2H), 6.91 (d, *J* = 8.5 Hz, 2H), 6.39 (dd, *J* = 2.9, 1.5 Hz, 1H), 4.77 (d, *J* = 2.3 Hz, 1H), 4.17 (dd, *J* = 6.4, 2.2 Hz, 1H), 3.03 – 3.00 (m, 1H), 2.18 – 2.13 (m, 1H), 1.95 – 1.91 (m, 1H) ppm.

^{13}C NMR (126 MHz, $CDCl_3$) δ 163.3, 150.0, 144.4, 144.3, 142.68, 142.67, 141.6, 137.6, 130.2, 130.1, 126.2, 126.1, 123.9, 123.3, 109.2, 91.8, 49.4, 45.5, 42.4, 35.7 ppm.

HRMS (ESI, *m*/*z*): Calcd for $C_{22}H_{17}ON_2INa^+$ [M+Na]$^+$: 475.0278, found: 475.0277.

IR: 2361 (s), 2185 (s), 1684 (s), 1411(s), 798 (s).

The major diastereoisomer:

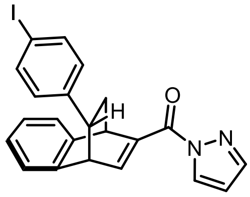

12b

***((1S,4R,10R)-10-*(*4*-iodophenyl)-*1,4*-dihydro-*1,4*-ethanonaphthalen-*3*-yl)(*1*H-pyrazol-*1*-yl)methanone (12b):**

^{1}H NMR (500 MHz, $CDCl_3$) δ 8.26 (d, *J* = 3.6 Hz, 1H), 8.21 (dd, *J* = 6.6, 1.9 Hz, 1H), 7.65 (s, 1H), 7.34 – 7.30 (m, 3H), 7.16 – 7.13 (m, 1H), 7.02 – 6.98 (m, 1H), 6.81 (d, *J* = 7.3 Hz, 1H), 6.38 (dd, *J* = 2.9, 1.5 Hz, 1H), 6.12 (d, *J* = 8.4 Hz, 2H), 4.80 (d, *J* = 2.3 Hz, 1H), 3.95 (dd, *J* = 6.6, 2.3 Hz, 1H), 3.14 – 3.11 (m, 1H), 2.33 – 2.27 (m, 1H), 1.52 – 1.48 (m, 1H) ppm.

^{13}C NMR (126 MHz, $CDCl_3$) δ 163.2, 153.1, 144.1, 143.8, 143.2, 139.8, 137.9, 137.1, 130.3, 130.1, 126.53, 126.45, 125.9, 123.1, 109.1, 91.8, 49.9, 43.4, 41.7, 34.8 ppm.

HRMS (ESI, *m*/*z*): Calcd for $C_{22}H_{17}ON_2INa^+$ [M+Na]$^+$: 475.0278, found: 475.0276.

IR: 2361 (s), 2157 (s), 1977 (s), 1683(s), 730 (s).

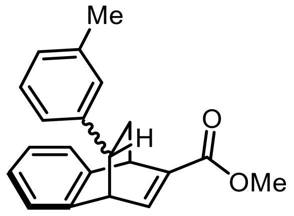

13

Methyl (1R,4S)-9-(m-tolyl)-1,4-dihydro-1,4-ethanonaphthalene-2-carboxylate (13): The title compound was prepared according to the general procedure using naphthalen-2-yl(1H-pyrazol-1-yl)methanone (44.4 mg, 0.200 mmol, 1.00 eq.), 1-methyl-3-vinylbenzene (236 mg, 2.00 mmol, 10.0 eq.) and *fac*-Ir(*p*-CF_3-ppy)$_3$ (1.7 mg, 2.0 mol%) in DCM (2.0 mL, 0.10 M) under nitrogen atmosphere with blue LEDs irridiation for 24 hours. The d.r. value was determined through ^{1}H NMR of crude materials as 1.3:1. Afterwards, the crude product was transformed into the corresponding methyl ester, the crude was purified by FCC (Hex:EtOAc = 40:1 to 20:1) to afford **13** (49.2 mg, 81% total yield) as a mixture of two diastereosiomers as a colorless oil.

Note: The yield was reported as an average of two runs.

^{1}H NMR (500 MHz, Chloroform-*d*) δ 7.65 (d, *J* = 6.5 Hz, 1H, major), 7.43 (d, *J* = 6.2 Hz, 1H, minor), 7.38 (d, *J* = 7.4 Hz, 1H, major), 7.34-7.31 (m, 1H, minor), 7.25 – 7.13 (m, 5H, mix), 7.07 – 7.03 (m, 2H, major), 6.99 – 6.91 (m, 4H, mix), 6.87 (d, *J* = 7.3 Hz, 1H, major), 6.27 (s, 1H, major), 6.21 (d, *J* = 7.7 Hz, 1H,major), 4.65 (s, 2H, mix), 4.19 (d, *J* = 6.3 Hz, 1H, minor), 3.93 (d, *J* = 6.3 Hz, 1H, major), 3.80 (s, 3H, minor), 3.79 (s, 3H, major), 3.13 – 3.10 (m, 1H, major), 3.07 – 3.04 (m, 1H, minor), 2.34 (s, 3H, minor), 2.22 – 2.19 (m, 2H, mix), 2.17 (s, 3H, major), 1.84 – 1.80 (m, 1H, minor), 1.61 – 1.57 (m, 1H, major).

^{13}C NMR (126 MHz, Chloroform-*d*) δ 165.5(major), 165.3(minor), 147.1(major), 144.7, 144.4, 144.1, 143.54(minor), 143.51, 142.9(minor), 140.9, 139.5, 138.8, 138.1(minor), 137.5, 129.1(major), 129.0(minor), 128.5(minor), 127.9, 127.3, 127.1(major), 126.3(major), 126.2(major), 125.9(minor), 125.8(minor), 125.4(major), 124.9(major), 124.7(minor), 123.8(minor), 123.0(minor), 122.9(major), 51.9(minor), 51.8(major), 49.5(major), 48.8(minor), 45.4(minor), 43.7(major), 40.8(minor), 40.4(major), 35.6(minor), 34.7(major), 21.7(minor), 21.5(major) ppm.

HRMS (APCI, *m*/*z*): Calcd for $C_{21}H_{21}O_2^+$ $[M+H]^+$: 305.1536, found: 305.1536.

IR is not collected due to product is a mixture.

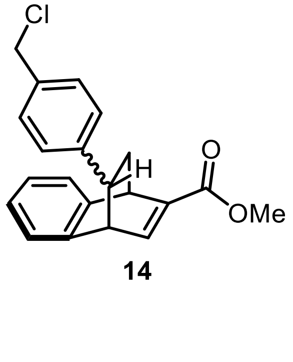

14

Methyl (1R,4S)-9-(4-(chloromethyl)phenyl)-1,4-dihydro-1,4-ethanonaphthalene-2-carboxylate (14): The title compound was prepared according to the general procedure using naphthalen-2-yl(1H-pyrazol-1-yl)methanone (44.4 mg, 0.200 mmol, 1.00 eq.), 1-(chloromethyl)-4-vinylbenzene (304 mg, 2.00 mmol, 10.0 eq.) and *fac*-Ir(*p*-CF_3-ppy)$_3$ (1.7 mg, 2.0 mol%) in DCM (2.0 mL, 0.10 M) under nitrogen atmosphere with blue LEDs irridiation for 24 hours. The d.r. value was determined through ^{1}H NMR of crude materials as 1.2:1. Afterwards, the crude product was transformed into the corresponding methyl ester, the crude was purified by FCC (Hex:EtOAc = 20:1 to 10:1) to afford **14** (39.9 mg, 59% total yield) as a mixture of two diastereosiomers as a colorless oil.

Note: The yield was reported as an average of two runs.

^{1}H NMR (500 MHz, Chloroform-*d*) δ 7.64 (d, *J* = 6.5 Hz, 1H, major), 7.40 (d, *J* = 6.5 Hz, 1H, minor), 7.37 (d, *J* = 7.3 Hz, 1H, major), 7.31 (d, *J* = 7.4 Hz, 3H, mix), 7.25 – 7.19 (m, 2H, mix), 7.15 – 7.04 (m, 6H, mix), 6.85 (d, *J* = 7.3 Hz, 2H, minor), 6.43 (d, *J* = 7.7 Hz, 2H, major), 4.64 (s, 2H, major), 4.57 (s, 2H, minor), 4.49 (s, 2H, mix), 4.17 (d, *J* = 6.3 Hz, 1H, minor), 3.93 (d, *J* = 6.4 Hz, 1H, major), 3.79 (s, 3H, minor), 3.78 (s, 3H, major), 3.17 – 3.13 (m, 1H, major), 3.10 – 3.07 (m, 1H, minor), 2.23 – 2.16 (m, 2H, mix), 1.82 – 1.78 (m, 1H, minor), 1.58 – 1.57 (m, 1H, major).

^{13}C NMR (126 MHz, Chloroform-*d*) δ 165.5(major), 165.3(minor), 146.9(major), 145.2(minor), 144.1(major), 144.0(major), 143.3(minor), 142.8(minor), 141.2(minor), 139.6(major), 138.5(major), 135.8(minor), 135.6(major), 128.9, 128.44(major), 128.35(major), 126.33(major), 126.26(major), 126.1(minor), 125.9(minor), 125.6(major), 123.9(minor), 123.0(major), 122.96, 52.0(minor), 51.9(major), 49.3(major), 48.8(minor), 46.2(major), 45.2(minor), 43.6(major), 40.8(minor), 40.3(major), 35.6(minor), 34.7(major), 29.9(minor) ppm.

HRMS (ESI, *m/z*): Calcd for $C_{21}H_{19}O_2ClNa^+$ $[M+Na]^+$: 361.0966, found: 361.0964.

IR is not collected due to product is a mixture.

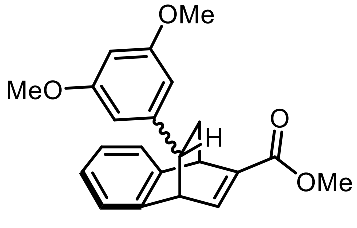

15

Methyl (1R,4S)-9-(3,5-dimethoxyphenyl)-1,4-dihydro-1,4-ethanonaphthalene-2-carboxylate (15): The title compound was prepared according to the general procedure using naphthalen-2-yl(1H-pyrazol-1-yl)methanone (44.4 mg, 0.200 mmol, 1.00 eq.), 1,3-dimethoxy-5-vinylbenzene (328 mg, 2.00 mmol, 10.0 eq.) and *fac*-Ir(*p*-CF_3-ppy)$_3$ (1.7 mg, 2.0 mol%) in DCM (2.0 mL, 0.10 M) under nitrogen atmosphere with blue LEDs irridiation for 24 hours. The d.r. value was determined through ^{1}H NMR of crude materials as 1.1:1. Afterwards, the crude product was transformed into the corresponding methyl ester, the crude was purified by FCC (Hex:EtOAc = 20:1 to 5:1) to afford **15** (53.2 mg, 76% total yield) as a mixture of two diastereosiomers as a colorless oil.

Note: The yield was reported as an average of two runs.
^{1}H NMR (500 MHz, Chloroform-*d*) δ 7.63 (dd, *J* = 6.5 Hz, 1H, minor), 7.43 (d, *J* = 6.1 Hz, 1H, major), 7.36 (d, *J* = 7.3 Hz, 1H, minor), 7.30 (d, *J* = 3.7 Hz, 1H, major), 7.25 – 7.18 (m, 2H), 7.14 – 7.13 (m, 2H), 7.07 (t, *J* = 7.4 Hz, 1H, major), 6.93 (d, *J* = 7.3 Hz, 1H, minor), 6.32 (s, 1H, major), 6.30 (s, 2H), 6.21 (s, 1H, minor), 5.61 (s, 2H), 4.63 (s, 2H, mix), 4.18 (d, *J* = 5.9 Hz, 1H, major), 3.95 (d, *J* = 6.6 Hz, 1H, minor), 3.78 (s, 13H, mix), 3.54 (s, 5H, mix), 3.09-3.06 (m, 1H, minor), 3.02 – 2.99 (m, 1H, major), 2.21-2.14 (m, 2H, mix), 1.80-1.76 (m, 1H, major), 1.58-1.54 (m, 1H, minor).
^{13}C NMR (126 MHz, Chloroform-*d*) δ 165.5(minor), 165.3(major), 160.9(major), 160.3(minor), 147.3, 146.9, 146.1(minor), 144.3(major), 144.1(minor), 143.3, 142.9, 140.8, 139.6, 139.1, 126.4, 126.2, 126.0, 125.8, 125.6, 123.8, 123.0, 122.96, 106.2(major), 105.9(minor), 99.0(minor), 98.2(major), 55.4(major), 55.2(minor), 51.89(major), 51.85(minor), 49.3(minor), 48.7(major), 45.7(major), 44.0(minor), 40.8(major), 40.3(minor), 35.7(major), 34.8(minor) ppm.
HRMS (APCI, *m*/*z*): Calcd for $C_{22}H_{23}O_4^+$ $[M+H]^+$: 351.1591, found: 351.1593.
IR is not collected due to product is a mixture.

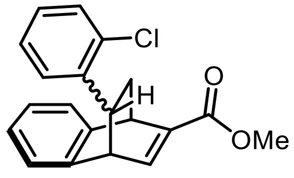

16

Methyl (1R,4S)-9-(2-chlorophenyl)-1,4-dihydro-1,4-ethanonaphthalene-2-carboxylate (16): The title compound was prepared according to the general procedure using naphthalen-2-yl(1H-pyrazol-1-yl)methanone (44.4 mg, 0.200 mmol, 1.00 eq.), 1-chloro-2-vinylbenzene (276 mg, 2.00 mmol, 10.0 eq.) and *fac*-Ir(*p*-CF_3-ppy)$_3$ (1.7 mg, 2.0 mol%) in DCM (2.0 mL, 0.10 M) under nitrogen atmosphere with blue LEDs irridiation for 24 hours. The d.r. value was determined through ^{1}H NMR of crude materials as 1.1:1. Afterwards, the crude product was transformed into the corresponding methyl ester, the crude was purified by FCC (Hex:EtOAc = 20:1 to 10:1) to afford **16** (51.8 mg, 80% total yield) as a mixture of two diastereosiomers as a colorless oil.
Note: The yield was reported as an average of two runs.
^{1}H NMR (500 MHz, Chloroform-*d*) δ 7.67 (d, *J* = 6.3 Hz, 1H, minor), 7.38 – 7.28 (m, 6H, mix), 7.21 – 7.13 (m, 5H, mix), 7.09 (d, *J* = 7.7 Hz, 1H, major), 7.02 (t, *J* = 7.6 Hz, 2H, mix), 6.81- 6.77 (m, 2H, mix), 5.72 (d, *J* = 8.0 Hz, 1H, minor), 4.67 (s, 2H, mix), 4.25 (d, *J* = 6.3 Hz, 1H, major), 3.99 (d, *J* = 6.6 Hz, 1H, minor), 3.92 (d, *J* = 5.1 Hz, 1H, minor), 3.79 (s, 6H, mix), 3.60 – 3.57 (m, 1H, major), 2.25-2.16 (m, 2H, mix), 1.82-1.77 (m, 1H, major), 1.64-1.62 (m, 1H, minor).
^{13}C NMR (126 MHz, Chloroform-*d*) δ 165.5(minor), 165.3(major), 146.8, 144.2(major), 143.9(minor), 143.1, 143.0, 141.7, 141.1, 139.8, 138.7, 134.7, 134.4, 129.8(major), 129.4, 129.3(minor), 128.2(minor), 128.1(major), 127.6(major), 127.5(minor), 126.9(major), 126.3(minor), 126.2(minor), 126.10(minor), 126.05, 125.92, 125.6(minor), 123.8, 123.2, 123.0(minor), 51.93(minor), 51.87(major),

47.3(minor), 46.9(major), 40.78(major), 40.73(major), 40.3(minor), 39.1(minor), 34.1(major), 33.6(minor) ppm.
HRMS (APCI, *m*/*z*): Calcd for $C_{20}H_{18}O_2Cl^+$[M+H]$^+$: 325.0990, found: 325.0991.
IR is not collected due to product is a mixture.

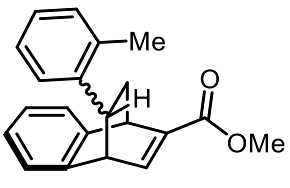

17

Methyl (1R,4S)-9-(o-tolyl)-1,4-dihydro-1,4-ethanonaphthalene-2-carboxylate (17): The title compound was prepared according to the general procedure using naphthalen-2-yl(1H-pyrazol-1-yl)methanone (44.4 mg, 0.200 mmol, 1.00 eq.), 1-methyl-2-vinylbenzene (236 mg, 2.00 mmol, 10.0 eq.) and *fac*-Ir(*p*-CF_3-ppy)$_3$ (1.7 mg, 2.0 mol%) in DCM (2.0 mL, 0.10 M) under nitrogen atmosphere with blue LEDs irridiation for 24 hours. The d.r. value was determined through ^{1}H NMR of crude materials as 1.3:1. Afterwards, the crude product was transformed into the corresponding methyl ester, the crude was purified by FCC (Hex:EtOAc = 20:1 to 10:1) to afford **17** (48 mg, 79% total yield) as a mixture of two diastereosiomers as a colorless oil.
Note: The yield was reported as an average of two runs.
^{1}H NMR (500 MHz, Chloroform-*d*) δ 7.58 (d, *J* = 6.5 Hz, 1H), 7.36 (d, *J* = 6.4 Hz, 1H, minor), 7.28 (d, *J* = 7.3 Hz, 1H), 7.24-7.23 (m, 1H, minor), 7.15 – 7.01 (m, 7H, mix), 6.96 - 6.88 (m, 4H, mix), 6.72 (d, *J* = 7.4 Hz, 1H), 6.66 (t, *J* = 7.7 Hz, 1H), 5.56 (d, *J* = 7.9 Hz, 1H), 4.57 (s, 2H, mix), 4.07 (d, *J* = 6.4 Hz, 1H, minor), 3.78 (d, *J* = 6.6 Hz, 1H, major), 3.70 (s, 6H, mix), 3.34 – 3.30 (m, 1H, major), 3.20 – 3.16 (m, 1H, minor), 2.33 (s, 3H, major), 2.23 (s, 3H, minor), 2.13 – 2.05 (m, 2H, mix), 1.69 – 1.65 (m, 1H, minor), 1.55 – 1.51 (m, 1H, major).
^{13}C NMR (126 MHz, Chloroform-*d*) δ 165.6(major), 165.3(minor), 147.2(major), 144.5, 144.0, 143.8(minor), 143.1(minor), 142.8(minor), 141.1, 140.8(minor), 139.6, 138.8, 136.4(minor), 136.0, 130.6, 130.2(major), 126.6(major), 126.31(minor), 126.28(major), 126.23(minor), 126.19, 126.09, 125.9, 125.8, 125.53(major), 125.52(major), 123.9(minor), 122.9(major), 122.8(minor), 51.9(minor), 51.8(major), 47.7(major), 47.3(minor), 41.4(minor), 40.8(minor), 40.4(major), 38.5(major), 34.7(minor), 34.0(major), 20.2(minor), 20.1(major) ppm.
HRMS (APCI, *m*/*z*): Calcd for $C_{21}H_{21}O_2^+$[M+H]$^+$: 305.1536, found: 305.1536.
IR is not collected due to product is a mixture.

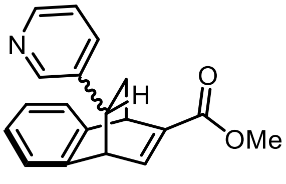

18

Methyl (10*S*)-10-(pyridin-3-yl)-1,4-dihydro-1,4-ethanonaphthalene-3-

carboxylate (18): The title compound was prepared according to the general procedure using naphthalen-2-yl(1H-pyrazol-1-yl)methanone (44.4 mg, 0.200 mmol, 1.00 eq.), 3-vinylpyridine (210 mg, 2.00 mmol, 10.0 eq.) and *fac*-Ir(*p*-CF_3-ppy)$_3$ (1.7 mg, 2.0 mol%) in DCM (2.0 mL, 0.10 M) under nitrogen atmosphere with blue LEDs irridiation for 24 hours. The d.r. value was determined through ^{1}H NMR of crude materials as 1.9:1. Afterwards, the crude product was transformed into the corresponding methyl ester, the crude was purified by FCC (Hex:EtOAc = 10:1 to 5:1) afford **18** (40 mg, 69% total yield) as a mixture of two diastereosiomers as a light yellow oil.

Note: The yield was reported as an average of two runs.

^{1}H NMR (500 MHz, CD_2Cl_2) δ 8.53 – 8.37 (m, 3H, mix), 8.30 (d, *J* = 5.0 Hz, 1H, minor), 7.97 (d, *J* = 15.0 Hz, 1H, minor), 7.61 (d, *J* = 6.5 Hz, 1H, minor), 7.47 – 7.41 (m, 2H, mix), 7.41 – 6.68 (m, 9H, mix), 6.45 – 6.40 (m, 1H, minor), 4.64 (s, 2H, mix), 4.18 (d, *J* = 6.3 Hz, 1H, major), 3.93 (d, *J* = 6.5 Hz, 1H, minor), 3.76 (dd, *J* = 5.9, 1.5 Hz, 6H, mix), 3.18 (dd, *J* = 10.7, 5.0 Hz, 1H, minor), 3.09 (dd, *J* = 9.5, 5.9 Hz, 1H, major), 2.27 – 2.17 (m, 2H, mix), 1.89 – 1.76 (m, 1H, major), 1.65 – 1.52 (m, 1H, minor).

The minor product: ^{13}C NMR (126 MHz, CD_2Cl_2) δ 165.4, 150.2, 148.1, 146.4, 144.4, 140.3, 139.3, 138.6, 135.1, 126.7, 126.4, 125.9, 123.4, 123.1, 52.0, 49.3, 41.4, 40.6, 34.8 ppm.

The major product: ^{13}C NMR (126 MHz, CD_2Cl_2) δ165.2, 150.1, 148.3, 143.6, 143.4, 143.1, 142.0, 140.2, 135.2, 126.3, 126.1, 124.0, 123.6, 123.4, 52.1, 48.7, 43.2, 41.0, 35.7 ppm.

HRMS (APCI, *m*/*z*): Calcd for $C_{19}H_{18}O_2N^+$[M+H]$^+$: 292.1332, found: 292.1335.

IR is not collected due to product is a mixture.

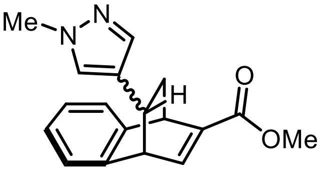

19

Methyl (10*S*)-10-(1-methyl-1*H*-pyrazol-3-yl)-1,4-dihydro-1,4-ethanonaphthalene-3-carboxylate (19): The title compound was prepared according to the general procedure using naphthalen-2-yl(1H-pyrazol-1-yl)methanone (44.4 mg, 0.200 mmol, 1.00 eq.), 1-methyl-3-vinyl-1*H*-pyrazole (216 mg, 2.00 mmol, 10.0 eq.) and *fac*-Ir(*p*-CF_3-ppy)$_3$ (1.7 mg, 2.0 mol%) in DCM (2.0 mL, 0.10 M) under nitrogen atmosphere with blue LEDs irridiation for 24 hours. The d.r. value was determined through ^{1}H NMR of crude materials as 1.2:1. Afterwards, the crude product was transformed into the corresponding methyl ester, the crude was purified by FCC (Hex:EtOAc = 10:1 to 5:1) afford **19** (47 mg, 80% total yield) as a mixture of two diastereosiomers as a light yellow oil.

Note: The yield was reported as an average of two runs.

^{1}H NMR (500 MHz, CD_2Cl_2) δ 7.57 (d, *J* = 6.4 Hz, 1H, major), 7.36 (d, *J* = 6.4 Hz, 2H, mix), 7.31 (d, *J* = 7.3 Hz, 1H, major), 7.28 – 7.24 (m, 2H, mix), 7.19 – 7.10 (m, 4H, mix), 7.07 (t, *J* = 7.4 Hz, 1H), 6.99 (d, *J* = 7.3 Hz, 1H), 6.69 (s, 1H), 6.51 (s, 1H),

5.32 (s, 1H, mix), 4.55 (s, 2H, mix), 4.18 (d, *J* = 6.3 Hz, 1H, minor), 3.95 (d, *J* = 6.5 Hz, 1H, major), 3.81 (s, 3H, minor), 3.74 (s, 6H, mix), 3.66 (s, 3H, major), 3.08 - 3.04 (m, 1H, major), 2.97 – 2.94 (m, 1H, minor), 2.23 – 2.15 (m, 1H, major), 2.14 – 2.07 (m, 1H, minor), 1.64 – 1.58 (m, 1H, minor), 1.38 – 1.32 (m, 1H, major).
^{13}C NMR (126 MHz, CD_2Cl_2) δ 165.6(major), 165.3(minor), 146.7(major), 144.9, 144.4, 143.21(minor), 143.16(minor), 140.9, 139.8, 139.7, 138.1, 138.0, 128.2(minor), 128.0(minor), 126.3(major), 126.1(minor), 125.9(minor), 125.6(major), 125.1(minor), 123.8, 123.5, 123.1(major), 51.94(minor), 51.88(major), 49.3(major), 49.0(minor), 40.9(minor), 40.6(major), 39.1(minor), 38.9(major), 36.4(minor), 36.2(major), 35.3(minor), 33.7(major) ppm.
HRMS (ESI, *m*/*z*): Calcd for $C_{18}H_{19}O_2N_2^+$[M+H]$^+$: 295.1441, found: 295.1442.
IR is not collected due to product is a mixture.

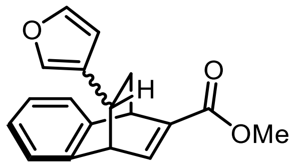

20

Methyl (10*S*)-10-(1-methyl-1*H*-pyrazol-3-yl)-1,4-dihydro-1,4-ethanonaphthalene-3-carboxylate (20): The title compound was prepared according to the general procedure using naphthalen-2-yl(1H-pyrazol-1-yl)methanone (44.4 mg, 0.200 mmol, 1.00 eq.), 3-vinylfuran (188 mg, 2.00 mmol, 10.0 eq.) and *fac*-Ir(*p*-CF_3-ppy)$_3$ (1.7 mg, 2.0 mol%) in DCM (2.0 mL, 0.10 M) under nitrogen atmosphere with blue LEDs irridiation for 24 hours. The d.r. value was determined through ^{1}H NMR of crude materials as 1.3:1. Afterwards, the crude product was transformed into the corresponding methyl ester, the crude was purified by FCC (Hex:EtOAc = 100:1 to 50:1) afford **20** (42 mg, 75% total yield) as a mixture of two diastereosiomers as a light yellow oil.
Note: The yield was reported as an average of two runs.
^{1}H NMR (500 MHz, $CDCl_3$) δ 7.62 (d, *J* = 6.4 Hz, 1H), 7.41 (d, *J* = 6.3 Hz, 1H), 7.36 (d, *J* = 1.3 Hz, 1H), 7.35 – 7.24 (m, 3H, mix), 7.22 – 7.13 (m, 4H, mix), 7.12 – 7.07 (m, 1H), 7.03 (d, *J* = 7.4 Hz, 1H), 6.88 (s, 1H), 6.26 (s, 1H), 5.45 (s, 1H, major), 5.32 (d, *J* = 1.5 Hz, 1H, minor), 4.61 (s, 2H, mix), 4.20 (d, *J* = 6.3 Hz, 1H, minor), 4.00 (d, *J* = 6.6 Hz, 1H, major), 3.79 (s, 6H, mix), 3.09 – 3.02 (m, 1H, major), 3.00 – 2.94 (m, 1H, minor), 2.21 – 2.06 (m, 2H, mix), 1.67 – 1.58 (m, 1H, major), 1.44 – 1.37 (m, 1H, minor).
^{13}C NMR (126 MHz, $CDCl_3$) δ 165.5(major), 165.3(minor), 146.6(major), 144.6, 143.8(minor), 143.2, 142.8(minor), 142.7(minor), 142.5(major), 140.5(minor), 139.5(minor), 139.2(minor), 139.1(major), 138.9, 129.1(minor), 128.1, 126.2, 126.0, 125.9, 125.8, 125.5, 123.7, 123.2, 123.0, 110.4(minor), 110.0(major), 51.89(minor), 51.85(major), 48.5(major), 48.2(minor), 40.4(minor), 40.1(major), 35.6(minor), 34.9(minor), 34.5(major), 33.9(major) ppm.
HRMS (APCI, *m*/*z*): Calcd for $C_{18}H_{17}O_4^+$[M+OH]$^+$: 297.1121, found: 297.1123.
IR is not collected due to product is a mixture.

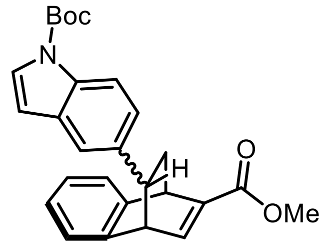

21

Tert-butyl 5-((1R,4S)-2-(methoxycarbonyl)-1,4-dihydro-1,4-ethanonaphthalen-9-yl)-1H-indole-1-carboxylate (21): The title compound was prepared according to the general procedure using naphthalen-2-yl(1H-pyrazol-1-yl)methanone (44.4 mg, 0.200 mmol, 1.00 eq.), tert-butyl 5-vinyl-1H-indole-1-carboxylate (486 mg, 2.00 mmol, 10.0 eq.) and *fac*-Ir(*p*-CF_3-ppy)$_3$ (1.7 mg, 2.0 mol%) in DCM (2.0 mL, 0.10 M) under nitrogen atmosphere with blue LEDs irridiation for 24 hours. The d.r. value was determined through ^{1}H NMR of crude materials as 1.4:1. Afterwards, the crude product was transformed into the corresponding methyl ester, the crude was purified by FCC (Hex:EtOAc = 10:1 to 3:1) to afford **21** (72.1 mg, 84% total yield) as a mixture of two diastereosiomers as a colorless oil.
Note: The yield was reported as an average of two runs.
^{1}H NMR (500 MHz, Chloroform-*d*) δ 8.04 (d, *J* = 8.5 Hz, 1H, minor), 7.84 (d, *J* = 8.6 Hz, 1H, major), 7.67 (d, *J* = 6.5 Hz, 1H, major), 7.60 (s, 1H, minor), 7.51 (s, 1H, major), 7.45 (d, *J* = 6.4 Hz, 1H, minor), 7.40 (d, *J* = 7.3 Hz, 1H, major), 7.33 (s, 2H, mix), 7.27 – 7.21 (m, 2H, mix), 7.17 – 7.15 (m, 2H, mix), 7.11 (d, *J* = 8.7 Hz, 1H, minor), 7.04 (t, *J* = 7.4 Hz, 1H, major), 6.81 (d, *J* = 7.3 Hz, 1H, major), 6.61 (s, 1H, major), 6.54 (s, 1H, minor), 6.42 (d, *J* = 8.7 Hz, 1H, major), 6.35 (s, 1H, major), 4.67 (s, 2H, mix), 4.22 (d, *J* = 6.1 Hz, 1H, minor), 3.96 (d, *J* = 6.2 Hz, 1H, major), 3.81 (s, 3H, minor), 3.79 (s, 3H, major), 3.28 – 3.25 (m, 1H, major), 3.21 – 3.18 (m, 1H, minor), 2.28 – 2.21 (m, 2H, mix), 1.92 – 1.87 (m, 1H, minor), 1.68 (s,10H, mix), 1.65 (s, 9H, major).
^{13}C NMR (126 MHz, Chloroform-*d*) δ 165.6(major), 165.4(minor), 149.8(minor), 147.2(major), 144.5, 144.1, 143.6, 142.9, 140.9, 139.4, 139.1, 138.9, 137.9, 134.0(minor), 130.9(minor), 130.4(minor), 126.4(minor), 126.3(major), 126.2(major), 126.0, 125.9, 125.8, 125.5(major), 124.6(major), 124.4(minor), 123.8, 123.0(minor), 122.9(major), 120.1(major), 119.8(minor), 115.2(minor), 114.6(major), 107.4(minor), 107.3(major), 83.8(minor), 83.6(minor), 51.9(minor), 51.8(major), 50.0(major), 49.4(minor), 45.4(minor), 43.7(major), 40.9(minor), 40.4(major), 35.9(minor), 35.0(major), 28.30(minor), 28.29(major) ppm.
HRMS (ESI, *m*/*z*): Calcd for $C_{27}H_{27}O_4NNa^+$ $[M+Na]^+$: 452.1832 found: 452.1830.
IR is not collected due to product is a mixture.

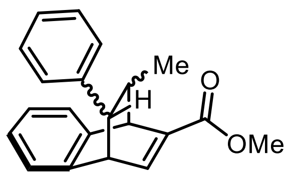

22

Methyl (1R,4S)-10-methyl-9-phenyl-1,4-dihydro-1,4-ethanonaphthalene-2-carboxylate (22): The title compound was prepared according to the general procedure using naphthalen-2-yl(1H-pyrazol-1-yl)methanone (44.4 mg, 0.200 mmol, 1.00 eq.), (*E*)-prop-1-en-1-ylbenzene or (*Z*)-prop-1-en-1-ylbenzene (236 mg, 2.00 mmol, 10.0 eq.) and *fac*-Ir(*p*-CF_3-ppy)$_3$ (1.7 mg, 2.0 mol%) in DCM (2.0 mL, 0.10 M) under nitrogen atmosphere with blue LEDs irridiation for 24 hours. The d.r. value was determined through ^{1}H NMR of crude materials as 1.3:1. Afterwards, the crude product was transformed into the corresponding methyl ester, the crude was purified by FCC (Hex:EtOAc = 40:1 to 20:1) to afford **22** (33.4 mg, 55% total yield from *trans-β*-methylstyrene; 23.7 mg, 39% total yield from *cis-β*-methylstyrene) as a mixture of two diastereosiomers as a colorless oil.

Note: The yield was reported as an average of two runs.

^{1}H NMR (500 MHz, Chloroform-*d*) δ 7.67 (d, *J* = 6.4 Hz, 1H, minor), 7.45 (d, *J* = 6.2 Hz, 1H, major), 7.33 (d, *J* = 7.4 Hz, 1H, minor), 7.34 – 7.03 (m, 15H, mix), 6.88 (d, *J* = 7.3 Hz, 1H, minor), 6.45 (d, *J* = 7.1 Hz, 1H, major), 4.36 (s, 1H, minor), 4.27 (s, 1H, major), 4.04 (d, *J* = 6.2 Hz, 1H, major), 3.82 (d, *J* = 6.7 Hz, 1H, minor), 3.78 (s, 3H, major), 3.76 (s, 3H, minor), 2.43 (d, *J* = 5.5 Hz, 1H, minor), 2.28 (d, *J* = 6.0 Hz, 1H, major), 2.05 (t, *J* = 6.7 Hz, 1H, major), 1.88 (t, *J* = 6.6 Hz, 1H, minor), 1.02 (d, *J* = 6.7 Hz, 3H, minor), 0.83 (d, *J* = 6.7 Hz, 3H, major).

^{13}C NMR (126 MHz, Chloroform-*d*) δ 166.2(minor), 165.4(major), 146.8(major), 145.0(minor), 144.3, 144.1(major), 143.5, 143.1(minor), 142.0, 140.3, 138.4(minor), 137.6(minor), 128.6(major), 128.3(minor), 128.1(minor), 127.9(major), 126.7(major), 126.6(minor), 126.3(minor), 126.2(minor), 126.1(major), 126.0(major), 125.7(major), 125.5(minor), 123.0(minor), 122.6(major), 54.9(major), 52.9(minor), 51.92(major), 51.87(minor), 49.6(minor), 49.2(major), 47.6(major), 47.1(minor), 42.6(major), 42.4(minor), 21.2(minor), 20.4(major) ppm.

HRMS (APCI, *m/z*): Calcd for $C_{21}H_{21}O_2^+$[M+H]$^+$: 305.1536, found: 305.1537.

IR is not collected due to product is a mixture.

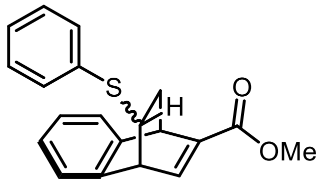

23

Methyl (1R,4R)-9-(phenylthio)-1,4-dihydro-1,4-ethanonaphthalene-2-carboxylate (23): The title compound was prepared according to the general procedure using naphthalen-2-yl(1H-pyrazol-1-yl)methanone (44.4 mg, 0.200 mmol, 1.00 eq.), phenyl(vinyl)sulfane (272 mg, 2.00 mmol, 10.0 eq.) and *fac*-Ir(*p*-CF_3-ppy)$_3$ (1.7 mg, 2.0 mol%) in DCM (2.0 mL, 0.10 M) under nitrogen atmosphere with blue LEDs irridiation for 24 hours. The d.r. value was determined through ^{1}H NMR of crude materials as 1.5:1. Afterwards, the crude product was transformed into the corresponding methyl ester, the crude was purified by FCC (Hex:EtOAc = 30:1 to 20:1) to afford **23** (46.4 mg, 72% total yield) as a mixture of two diastereosiomers as a colorless oil.

Note: The yield was reported as an average of two runs.
^{1}H NMR (500 MHz, Chloroform-*d*) δ 7.50 – 7.12 (m, 20H, mix), 4.59 (s, 1H, minor), 4.56 (s, 1H, major), 4.23 (d, *J* = 6.2 Hz, 1H, minor), 4.06 (d, *J* = 6.5 Hz, 1H, major), 3.80 (s, 3H, minor), 3.77 (s, 3H, major), 3.52 – 3.48 (m, 1H, major), 3.44 – 3.41 (m, 1H, minor), 2.33 – 2.28 (m, 1H, major), 2.24 – 2.19 (m, 1H, minor), 1.55 – 1.51 (m, 1H, minor), 1.33 – 1.28 (m, 1H, major).
^{13}C NMR (126 MHz, Chloroform-*d*) δ 165.2(mix), 145.3(major), 144.3, 142.7(minor), 142.6, 141.1(minor), 140.4, 139.7(minor), 138.5, 135.64(minor), 135.59, 131.7(major), 131.3, 129.3(minor), 129.2(major), 127.2(major), 127.1(minor), 126.6(major), 126.5(minor), 126.2(major), 126.1(minor), 125.8(major), 123.8(minor), 123.6(minor), 123.2(major), 52.0(minor), 51.9(major), 46.0(minor), 45.8(minor), 45.6(major), 44.8(major), 40.2(minor), 40.0(major), 34.6(minor), 34.2(major) ppm.
HRMS (APCI, *m*/*z*): Calcd for $C_{20}H_{19}O_2S^+$ [M+H]$^+$: 323.1100, found: 323.1102.
IR is not collected due to product is a mixture.

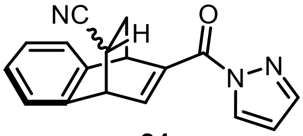

24

The title compound was prepared according to the general procedure using naphthalen-2-yl(1H-pyrazol-1-yl)methanone (44.4 mg, 0.200 mmol, 1.00 eq.), acrylonitrile (106 mg, 2.00 mmol, 10.0 eq.) and *fac*-Ir(*p*-CF_3-ppy)$_3$ (1.7 mg, 2.0 mol%) in DCM (2.0 mL, 0.10 M) under nitrogen atmosphere with blue LEDs irridiation for 24 hours. The d.r. value was determined through ^{1}H NMR of crude materials as 1.4:1, the crude was purified by FCC (Hex:EtOAc = 10:1 to 5:1) to afford **24** (46.2 mg, 84.0% total yield; minor: 18.5 mg, 40.0% yield, major: 27.7 mg, 44.0% yield) as two separable diastereoisomers as a colorless oil.
Note: The yield was reported as an average of two runs.
The minor diastereoisomer:

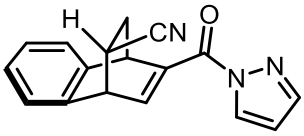

24a

***(1S,4R,10S)*-3-(1H-pyrazole-1-carbonyl)-1,4-dihydro-1,4-ethanonaphthalene-10-carbonitrile (24a):**
^{1}H NMR (500 MHz, Chloroform-*d*) δ 8.26 (s, 1H), 8.07 (d, *J* = 6.4 Hz, 1H), 7.64 (s, 1H), 7.21 – 7.19 (m, 2H), 7.11 – 7.09 (m, 2H), 6.39 (s, 1H), 4.82 (s, 1H), 4.49 (d, *J* = 6.4 Hz, 1H), 2.74 – 2.71 (m, 1H), 2.12 – 2.00 (m, 2H).
^{13}C NMR (126 MHz, Chloroform-*d*) δ 162.7, 148.2, 144.5, 142.0, 141.8, 138.8, 130.2, 127.3, 126.6, 124.1, 124.0, 122.0, 109.5, 44.2, 40.8, 32.9, 28.4 ppm.
HRMS (ESI, *m*/*z*): Calcd for $C_{17}H_{13}ON_3Na^+$ [M+Na]$^+$: 298.0951, found: 298.0950.
IR: 3150 (s), 2948 (s), 2238 (s), 1686 (s), 1475 (s).
The major diastereoisomer:

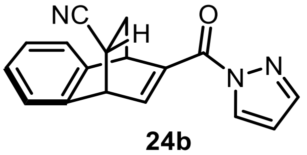

24b

***(1S,4R,10R)*-3-(1H-pyrazole-1-carbonyl)-1,4-dihydro-1,4-ethanonaphthalene-10-carbonitrile (24b):**

^{1}H NMR (500 MHz, Chloroform-*d*) δ 8.23 (s, 1H), 7.96 (d, *J* = 6.6 Hz, 1H), 7.63 (s, 1H), 7.33 – 7.31 (m, 1H), 7.25 – 7.24 (m, 1H), 7.18 – 7.15 (m, 2H), 6.38 (s, 1H), 4.78 (s, 1H), 4.38 (d, *J* = 6.5 Hz, 1H), 2.85 – 2.83 (m, 1H), 2.30 – 2.25 (m, 1H), 1.77 – 1.73 (m, 1H).

^{13}C NMR (126 MHz, Chloroform-*d*) δ 162.6, 148.8, 144.4, 142.2, 141.5, 136.9, 130.2, 127.4, 126.8, 125.5, 123.7, 121.1, 109.5, 44.2, 40.6, 32.3, 28.1 ppm.

HRMS (ESI, *m/z*): Calcd for $C_{17}H_{13}ON_3Na^+$ $[M+Na]^+$: 298.0951, found: 298.0951.

IR: 3131 (m), 2238 (s), 1686 (s), 1475 (s), 1357 (s).

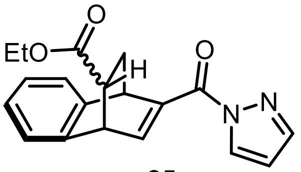

25

The title compound was prepared according to the general procedure using naphthalen-2-yl(1H-pyrazol-1-yl)methanone (44.4 mg, 0.200 mmol, 1.00 eq.), ethyl acrylate (200 mg, 2.00 mmol, 10.0 eq.) and *fac*-Ir(*p*-CF_3-ppy)$_3$ (1.7 mg, 2.0 mol%) in DCM (2.0 mL, 0.10 M) under nitrogen atmosphere with blue LEDs irridiation for 24 hours. The d.r. value was determined through ^{1}H NMR of crude materials as 1.1:1, the crude was purified by FCC (Hex:EtOAc = 20:1 to 5:1) to afford **25** (16.7 mg, 26.0% total yield; minor: 5.60 mg, 8.70% yield, major: 11.1 mg, 17.3% yield) as two separable diastereoisomers as a colorless oil.

Note: The yield was reported as an average of two runs.

The minor diastereoisomer:

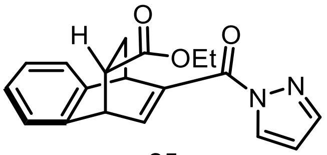

25a

Ethyl *(1S,4R,10S)*-3-(1H-pyrazole-1-carbonyl)-1,4-dihydro-1,4-ethanonaphthalene-10-carboxylate (25a):

^{1}H NMR (500 MHz, Chloroform-*d*) δ 8.29 (s, 1H), 7.66 (s, 1H), 7.29 (d, *J* = 6.7 Hz, 2H), 7.20 – 7.15 (m, 2H), 7.11 (d, *J* = 6.7 Hz, 1H), 6.39 (s, 1H), 4.56 (d, *J* = 5.4 Hz, 1H), 4.12 (t, *J* = 7.6 Hz, 2H), 3.72 (d, *J* = 8.8 Hz, 1H), 2.72 (td, *J* = 11.3, 5.3 Hz, 1H), 2.46 (dd, *J* = 11.1, 6.2 Hz, 1H), 2.13 (dd, *J* = 11.7, 6.2 Hz, 1H), 1.21 (t, *J* = 7.1 Hz, 3H).

^{13}C NMR (126 MHz, Chloroform-*d*) δ 174.2, 170.7, 146.2, 143.9, 139.3, 129.5, 127.1, 124.1, 123.4, 108.6, 50.1, 47.9, 46.4, 45.2, 42.4, 14.3 ppm.

HRMS (ESI, *m/z*): Calcd for $C_{19}H_{18}O_3Na^+$ $[M+Na]^+$:345.1210, found: 345.1210.

IR: 2981 (s), 1729 (s), 1685 (s), 1380 (s), 912 (s).

The major diastereoisomer:

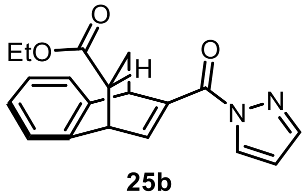

25b

Ethyl *(1S,4R,10R)*-3-(1H-pyrazole-1-carbonyl)-1,4-dihydro-1,4-ethanonaphthalene-10-carboxylate (25b):

^{1}H NMR (500 MHz, Chloroform-*d*) δ 8.23 (s, 1H), 8.00 (d, *J* = 6.4 Hz, 1H), 7.61 (s, 1H), 7.21 – 7.18 (m, 2H), 7.08 – 7.05 (m, 2H), 6.34 (s, 1H), 4.74 (s, 1H), 4.50 (d, *J* = 6.4 Hz, 1H), 4.09 – 4.04 (m, 2H), 2.72 – 2.69 (m, 1H), 2.06 – 2.02 (m, 1H), 1.95 – 1.89 (m, 1H), 1.18 (t, *J* = 7.3 Hz, 3H).

^{13}C NMR (126 MHz, Chloroform-*d*) δ 174.2, 163.0, 150.6, 144.1, 143.2, 140.8, 140.6, 130.2, 126.4, 126.1, 123.8, 109.0, 61.1, 44.8, 44.4, 41.4, 31.6, 14.3 ppm.

HRMS (ESI, *m*/*z*): Calcd for $C_{19}H_{18}O_3Na^+$ $[M+Na]^+$:345.1210, found: 345.1212.

IR: 2981 (s), 1729 (s), 1685 (s), 1380 (s), 912 (s).

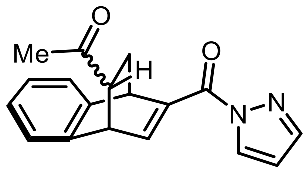

26

The title compound was prepared according to the general procedure using naphthalen-2-yl(1H-pyrazol-1-yl)methanone (44.4 mg, 0.200 mmol, 1.00 eq.), but-3-en-2-one (140 mg, 2.00 mmol, 10.0 eq.) and *fac*-Ir(*p*-CF_3-ppy)$_3$ (1.7 mg, 2.0 mol%) in DCM (2.0 mL, 0.10 M) under nitrogen atmosphere with blue LEDs irridiation for 24 hours. The d.r. value was determined through ^{1}H NMR of crude materials as 3:1, the crude was purified by FCC (Hex:EtOAc = 10:1 to 5:1) to afford **26** (25.1 mg, 43.0% total yield; minor: 5.00 mg, 8.60% yield, major: 20.1 mg, 34.4% yield) as a major diastereosiomer as a colorless oil.

Note: The yield was reported as an average of two runs.

The minor diastereoisomer:

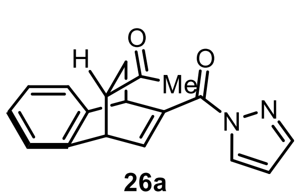

26a

1-(*(1S,4R,10S)*-3-(1H-pyrazole-1-carbonyl)-1,4-dihydro-1,4-ethanonaphthalen-10-yl)ethan-1-one (26a):

^{1}H NMR (500 MHz, Chloroform-*d*) δ 8.21 (s, 1H), 8.04 (d, *J* = 6.5 Hz, 1H), 7.61 (s, 1H), 7.22 – 7.17 (m, 2H), 7.07 – 7.06 (m, 2H), 6.34 (s, 1H), 4.74 (s, 1H), 4.40 (d, *J* =

6.2 Hz, 1H), 2.82 – 2.79 (m, 1H), 2.08 (s, 3H), 2.03 – 1.96 (m, 1H), 1.88 – 1.82 (m, 1H).
^{13}C NMR (126 MHz, Chloroform-*d*) δ 206.9, 163.0, 151.3, 144.1, 143.0, 141.1, 139.8, 130.2, 126.3, 126.2, 123.9, 123.6, 109.1, 54.2, 43.6, 41.3, 30.9, 29.1 ppm.
HRMS (ESI, *m*/*z*): Calcd for $C_{18}H_{16}O_2N_2Na^+$ $[M+Na]^+$: 315.1104, found: 315.1105.
IR: 2973 (m), 1706 (s), 1379 (s), 1306 (s), 740 (s).
The major diastereoisomer:

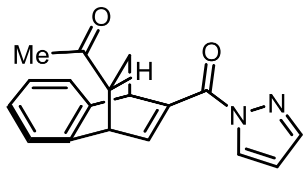

26b

1-(*(1S,4R,10R)*-3-(1H-pyrazole-1-carbonyl)-1,4-dihydro-1,4-ethanonaphthalen-10-yl)ethan-1-one (26b):
^{1}H NMR (500 MHz, Chloroform-*d*) δ 8.31 (s, 1H), 8.19 (d, *J* = 6.6 Hz, 1H), 7.71 (s, 1H), 7.29 – 7.21 (m, 2H), 7.15 – 7.11 (m, 2H), 6.45 (s, 1H), 4.81 (s, 1H), 4.53 (d, *J* = 6.9 Hz, 1H), 2.91 – 2.88 (m, 1H), 2.06 (s, 4H), 1.95 – 1.92 (m, 1H).
^{13}C NMR (126 MHz, Chloroform-*d*) δ 206.9, 163.0, 151.7, 144.2, 143.3, 141.3, 137.8, 130.2, 126.5, 126.1, 125.1, 123.4, 109.2, 51.3, 44.2, 41.3, 29.1, 28.8 ppm.
HRMS (ESI, *m*/*z*): Calcd for $C_{18}H_{16}O_2N_2Na^+$ $[M+Na]^+$: 315.1104, found: 315.1104.
IR: 2973 (m), 1706 (s), 1379 (s), 1306 (s), 740 (s).

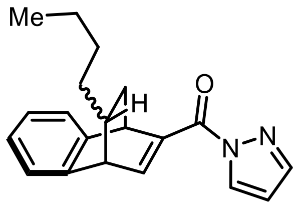

27

((1R,4R)-9-butyl-1,4-dihydro-1,4-ethanonaphthalen-2-yl)(1H-pyrazol-1-yl)methanone (27): The title compound was prepared according to the general procedure using naphthalen-2-yl(1H-pyrazol-1-yl)methanone (44.4 mg, 0.200 mmol, 1.00 eq.), 1-hexene (200 mg, 2.00 mmol, 10.0 eq.) and *fac*-Ir(*p*-CF_3-ppy)$_3$ (1.7 mg, 2.0 mol%) in DCM (2.0 mL, 0.10 M) under nitrogen atmosphere with blue LEDs irridiation for 24 hours. The d.r. value was determined through ^{1}H NMR of crude materials as 1.2:1, the crude was purified by FCC (Hex:EtOAc = 50:1 to 20:1) to afford **27** (8.6 mg, 14% total yield) as a mixture of two diastereosiomers as a colorless oil.
Note: The yield was reported as an average of two runs.
^{1}H NMR (500 MHz, Chloroform-*d*) δ 8.23 (d, *J* = 8.3 Hz, 2H, mix), 8.13 (d, *J* = 6.7 Hz, 1H, minor), 7.96 (d, *J* = 6.5 Hz, 1H, major), 7.62 (s, 2H, mix), 7.19 – 7.02 (m, 8H, mix), 6.35 (s, 2H, mix), 4.63 (s, 1H, major), 4.60 (s, 1H, minor), 4.05 (d, *J* = 6.5 Hz, 1H, major), 3.95 (d, *J* = 6.8 Hz, 1H, minor), 1.99 – 1.94 (m, 1H, major), 1.84 – 1.73 (m, 3H, mix), 1.38 – 1.32 (m, 2H, major), 1.27 – 1.14 (m, 10H, mix), 0.96 – 0.87 (m, 2H, major), 0.81 (t, *J* = 6.9 Hz, 3H, major), 0.76 (t, *J* = 6.8 Hz, 3H, minor).

^{13}C NMR (126 MHz, Chloroform-*d*) δ 163.3(minor), 163.1(major), 154.0(minor), 151.7(major), 144.0(major), 143.9, 143.5(minor), 142.9, 142.88, 140.0, 139.3(minor), 139.1(minor), 130.3, 130.2(minor), 125.9, 125.8, 125.7, 125.6, 125.4, 123.6, 123.2, 123.0, 108.90(major), 108.86(minor), 47.5(major), 47.0(minor), 41.8(major), 41.4(minor), 40.6(major), 37.4(minor), 36.9(major), 35.6(minor), 34.1(major), 33.7(minor), 30.3(major), 30.1(minor), 22.9(major), 22.8(minor), 14.24(major), 14.21(minor) ppm.
HRMS (ESI, *m*/*z*): Calcd for $C_{20}H_{22}ON_2Na^+$ [M+Na]$^+$: 329.1624, found: 329.1625.
IR is not collected due to product is a mixture.

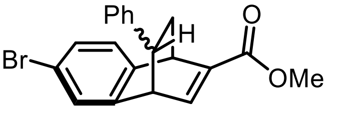

28

Methyl (1R,4S)-6-bromo-9-phenyl-1,4-dihydro-1,4-ethanonaphthalene-2-carboxylate (28): The title compound was prepared according to the general procedure using (6-bromonaphthalen-2-yl)(1H-pyrazol-1-yl)methanone (60 mg, 0.20 mmol, 1.0 eq.), styrene (0.23 mL, 2.0 mmol, 10 eq.) and *fac*-Ir(*p*-CF_3-ppy)$_3$ (1.7 mg, 2.0 mol%) in DCM (2.0 mL, 0.10 M) under nitrogen atmosphere with blue LEDs irridiation for 24 hours. The d.r. value was determined through ^{1}H NMR of crude materials as 1.3:1. Afterwards, the crude product was transformed into the corresponding methyl ester, the crude was purified by FCC (Hex:EtOAc = 20:1 to 10:1) to afford **27** (58.9 mg, 80% total yield) as a mixture of two diastereosiomers as a colorless oil.
Note: The yield was reported as an average of two runs.
^{1}H NMR (500 MHz, Chloroform-*d*) δ 7.52 (d, *J* = 6.4 Hz, 1H, major), 7.31 – 7.03 (m, 14H, mix), 6.92 (s, 1H, major), 6.42 – 6.40 (m, 2H), 4.54 (s, 2H, mix), 4.05 (d, *J* = 6.1 Hz, 1H, minor), 3.82 (d, *J* = 6.7 Hz, 1H, major), 3.72 (s, 3H, minor), 3.70 (s, 3H, major), 3.09 – 3.05 (m, 1H, major), 3.00 – 2.97 (m, 1H, minor), 2.14 – 2.05 (m, 2H, mix), 1.75 – 1.71 (m, 1H, minor), 1.54 – 1.50 (m, 1H, major).
^{13}C NMR (126 MHz, Chloroform-*d*) δ 165.3(major), 165.1(minor), 146.3(major), 145.7(minor), 144.2, 143.6, 143.2, 143.0, 141.9, 141.2, 141.1, 139.5, 129.12, 129.08, 128.8(minor), 128.7, 128.27, 128.25, 127.9(major), 126.8(minor), 126.7(major), 126.2, 125.3, 124.4, 119.4(minor), 119.2(major), 52.0(minor), 51.9(major), 49.3(major), 48.6(minor), 45.2(minor), 43.6(major), 40.4(minor), 39.9(major), 35.3(minor), 34.2(major) ppm.
HRMS (APCI, *m*/*z*): Calcd for $C_{19}H_{12}BrO_2^+$ [M-CH_5+H]$^+$: 351.0015, found: 351.0016.
IR is not collected due to product is a mixture.

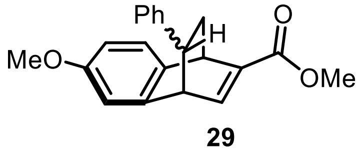

29

Methyl (10*S*)-7-methoxy-10-phenyl-1,4-dihydro-1,4-ethanonaphthalene-3-carboxylate (29): The title compound was prepared according to the general procedure using (6-methoxynaphthalen-2-yl)(1*H*-pyrazol-1-yl)methanone (50.5 mg, 0.200 mmol,

1.00 eq.), styrene (208 mg, 2.00 mmol, 10.0 eq.) and *fac*-Ir(*p*-CF_3-ppy)$_3$ (1.7 mg, 2.0 mol%) in DCM (2.0 mL, 0.10 M) under nitrogen atmosphere with blue LEDs irridiation for 24 hours. The d.r. value was determined through ^{1}H NMR of crude materials as 1.4:1. Afterwards, the crude product was transformed into the corresponding methyl ester, the crude was purified by FCC (Hex:EtOAc = 100:1) afford **29** (57 mg, 88% total yield) as a mixture of two diastereosiomers as a light yellow oil.
Note: The yield was reported as an average of two runs.
^{1}H NMR (500 MHz, $CDCl_3$) δ 7.62 (dd, *J* = 6.5, 1.8 Hz, 2H), 7.39 (dd, *J* = 6.3, 1.8 Hz, 1H, minor), 7.30 – 7.08 (m, 8H, mix), 6.85 (d, *J* = 2.5 Hz, 1H, minor), 6.73 (dd, *J* = 8.1, 2.5 Hz, 2H), 6.66 (dd, *J* = 8.0, 2.5 Hz, 1H, minor), 6.53 – 6.48 (m, 2H), 6.45 (d, *J* = 2.5 Hz, 1H, major), 4.58 (d, *J* = 2.4 Hz, 2H, mix), 4.12 (dd, *J* = 6.3, 2.1 Hz, 1H, minor), 3.88 (dd, *J* = 6.5, 2.3 Hz, 1H, major), 3.80 (d, *J* = 6.5, 2.3 Hz, 6H, mix), 3.77 (s, 3H), 3.69 (s, 3H), 3.16 – 3.12 (m, 1H, major), 3.10 – 3.07 (m, 1H, minor), 2.21 – 2.16 (m, 2H, mix), 1.81 – 1.77 (m, 1H, minor), 1.62 – 1.58 (m, 1H, major).
^{13}C NMR (126 MHz, $CDCl_3$) δ 165.6(major), 165.3(minor), 158.1(minor), 157.9(major), 146.8(major), 145.0(minor), 144.8(minor), 143.9(minor), 143.5, 141.7(minor), 140.2, 140.2, 136.6, 135.1(minor), 128.6, 128.1, 128.1(major), 127.9, 126.6(minor), 126.5, 124.3(minor), 123.4, 112.9(major), 111.1(major), 110.2(minor), 109.9(minor), 55.64(major), 55.61(minor), 51.9(minor), 51.8(major), 49.8(major), 49.1(minor), 45.5(minor), 43.8, 40.0(minor), 39.6(major), 36.1(minor), 34.9(major) ppm.
HRMS (APCI, *m/z*): Calcd for $C_{13}H_{13}O_3^+$ $[M+H]^+$: 321.1485, found: 321.1486.
IR is not collected due to product is a mixture.

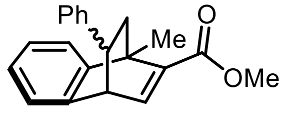

30

The title compound was prepared according to the general procedure using (1-methylnaphthalen-2-yl)(1H-pyrazol-1-yl)methanone (47.2 mg, 0.200 mmol, 1.00 eq.), styrene (208 mg, 2.00 mmol, 10.0 eq.) and *fac*-Ir(*p*-CF_3-ppy)$_3$ (1.7 mg, 2.0 mol%) in DCM (2.0 mL, 0.10 M) under nitrogen atmosphere with blue LEDs irridiation for 24 hours. The d.r. value was determined through ^{1}H NMR of crude materials as 1:1. Afterwards, the crude product was transformed into the corresponding methyl ester, the crude was purified by FCC (Hex:EtOAc = 40:1) afford **30** (25 mg, 41% total yield) as a mixture of two diastereosiomers as a light yellow oil.
Note: The yield was reported as an average of two runs.
^{1}H NMR (500 MHz, Chloroform-*d*) δ 7.64 (d, *J* = 6.6 Hz, 1H, major), 7.56 (d, *J* = 6.6 Hz, 1H, minor), 7.37 (d, *J* = 7.5 Hz, 1H, major), 7.32 (d, *J* = 7.1 Hz, 1H, minor), 7.24 – 6.98 (m, 13H, mix), 6.84 (d, *J* = 7.3 Hz, 1H, major), 6.47 (d, *J* = 6.9 Hz, 2H, major), 4.13 – 4.11 (m, 1H, minor), 3.87 (d, *J* = 6.8 Hz, 1H, major), 3.71 (s, 6H, mix), 3.25 – 3.22 (m, 1H, major), 2.99 – 2.96 (m, 1H, minor), 2.39 – 2.34 (m, 1H, minor), 2.21 – 2.16 (m, 1H, major), 2.02 (s, 3H, major), 1.69 – 1.65 (m, 1H, minor), 1.55 (s, 3H), 1.42 – 1.39 (m, 1H, major).

^{13}C NMR (126 MHz, Chloroform-*d*) δ 166.2(minor), 166.1(major), 148.2(major), 147.6, 147.1, 143.7, 143.5, 142.9, 142.5, 142.4, 141.0, 139.0, 128.1(major), 128.0(major), 127.7, 126.4, 126.1, 126.0(major), 125.6(minor), 125.3, 124.0(minor), 122.6(minor), 120.2(major), 51.51(minor), 51.49(major), 50.8(minor), 49.1(major), 47.3(minor), 45.1(major), 43.8, 43.6(minor), 41.2(major), 37.8(minor), 19.0, (major) 17.1(minor) ppm.
HRMS (APCI, *m*/*z*): Calcd for $C_{21}H_{21}O_2^+$ $[M+Na]^+$: 305.1536, found: 305.1537.
IR is not collected due to product is a mixture.

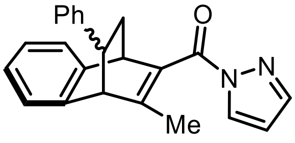

31

The title compound was prepared according to the general procedure using (3-methylnaphthalen-2-yl)(1*H*-pyrazol-1-yl)methanone (47.2 mg, 0.200 mmol, 1.00 eq.), styrene (208 mg, 2.00 mmol, 10.0 eq.) and *fac*-Ir(*p*-CF_3-ppy)$_3$ (1.7 mg, 2.0 mol%) in DCM (2.0 mL, 0.10 M) under nitrogen atmosphere with blue LEDs irridiation for 24 hours. The d.r. value was determined through ^{1}H NMR of crude materials as 1.1:1. Afterwards, the crude product was transformed into the corresponding methyl ester, the crude was purified by FCC (Hex:EtOAc = 10:1) afford **31** (44 mg, 64% total yield) as a mixture of two diastereosiomers as a light yellow oil.
Note: The yield was reported as an average of two runs.
The minor diastereoisomer:

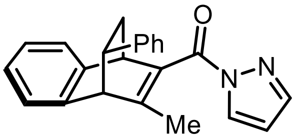

31a

***((1R,4R,10S)*-2-methyl-10-phenyl-1,4-dihydro-1,4-ethanonaphthalen-3-yl)(1H-pyrazol-1-yl)methanone (31a):**
^{1}H NMR (500 MHz, Chloroform-*d*) δ 8.22 (d, J = 2.9 Hz, 1H), 7.71 (d, J = 1.5 Hz, 1H), 7.28 – 7.15 (m, 7H), 7.15 – 7.11 (m, 1H), 7.08 (dd, J = 5.4, 3.2 Hz, 2H), 6.42 (dd, J = 2.9, 1.5 Hz, 1H), 4.45 (t, J = 2.7 Hz, 1H), 3.78 (d, J = 2.5 Hz, 1H), 3.11 – 3.08 (m, 1H), 2.55 – 2.51 (m, 1H), 2.01 – 1.96 (m, 1H).
^{13}C NMR (126 MHz, Chloroform-*d*) δ 166.1, 156.4, 144.4, 143.9, 143.9, 142.8, 134.8, 129.5, 128.6, 128.2, 126.6, 126.1, 125.7, 123.2, 123.1, 109.3, 57.0, 45.4, 44.2, 34.4, 21.1 ppm.
HRMS (ESI, *m*/*z*): Calcd for $C_{23}H_{20}ON_2Na^+$ $[M+Na]^+$: 363.1468, found: 363.1469.
IR: 2940 (s), 1695 (s), 1378 (s), 1331 (s), 756 (s).
The major diastereoisomer:

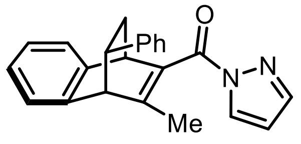

31a
minor

and

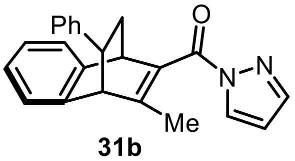

31b
major

(*(1R,4R,10R)*-2-methyl-10-phenyl-1,4-dihydro-1,4-ethanonaphthalen-3-yl)(1H-pyrazol-1-yl)methanone (31b):

^{1}H NMR (500 MHz, Chloroform-*d*) δ 8.26 (d, *J* = 2.2 Hz, 1H), 7.75 (d, *J* = 0.6 Hz, 1H), 7.32 – 7.30 (m, 1H), 7.24 – 7.20 (m, 2H), 7.11 – 7.09 (m, 3H), 6.90 (d, *J* = 6.2 Hz, 1H), 6.53 – 6.51 (m, 2H), 6.47 – 6.45 (m, 1H), 4.54 (t, *J* = 2.7 Hz, 1H), 3.73 (d, *J* = 2.2 Hz, 1H), 3.28 – 3.25 (m, 1H), 2.77 – 2.72 (m, 1H), 2.19 (s, 3H), 1.62 – 1.58 (m, 1H).
^{13}C NMR (126 MHz, $CDCl_3$) δ 166.0, 159.4, 144.8, 144.0, 143.9, 138.4, 133.6, 129.6, 128.1, 128.1, 126.4, 126.3, 126.2, 125.5, 122.6, 109.2, 56.6, 44.2, 43.7, 35.9, 19.0 ppm.
HRMS (ESI, *m*/*z*): Calcd for $C_{23}H_{20}ON_2Na^+$ $[M+Na]^+$: 363.1468, found: 363.1468.
IR is not collected due to product is a mixture.

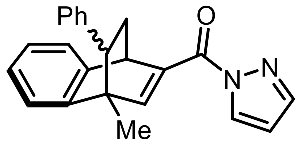

32

The title compound was prepared according to the general procedure using (3-methylnaphthalen-2-yl)(1*H*-pyrazol-1-yl)methanone (47.2 mg, 0.200 mmol, 1.00 eq.), styrene (208 mg, 2.00 mmol, 10.0 eq.) and *fac*-Ir(*p*-CF_3-ppy)$_3$ (1.7 mg, 2.0 mol%) in DCM (2.0 mL, 0.10 M) under nitrogen atmosphere with blue LEDs irridiation for 24 hours. The d.r. value was determined through ^{1}H NMR of crude materials as 1.5:1. Afterwards, the crude product was transformed into the corresponding methyl ester, the crude was purified by FCC (Hex:EtOAc = 10:1) afford **32** (27 mg, 40% total yield) as a mixture of two diastereosiomers as a light yellow oil.
Note: The yield was reported as an average of two runs.

The minor diastereoisomer:

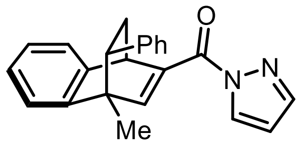

32a

((1S,4R,10R)-1-methyl-10-phenyl-1,4-dihydro-1,4-ethanonaphthalen-3-yl)(1H-pyrazol-1-yl)methanone (32a):

^{1}H NMR (500 MHz, Chloroform-*d*) δ 8.37 (d, *J* = 2.9 Hz, 1H), 7.73 (d, *J* = 1.5 Hz, 1H), 7.69 (d, *J* = 1.9 Hz, 1H), 7.34 (d, *J* = 7.1 Hz, 1H), 7.30 (d, *J* = 7.3 Hz, 1H), 7.25 – 7.14 (m, 7H), 6.48 (dd, *J* = 2.9, 1.5 Hz, 1H), 4.83 – 4.82 (m, 1H), 2.74 – 2.71 (m, 1H), 2.39 – 2.34 (m, 1H), 2.12 – 2.08 (m, 1H), 1.51 (s, 3H) ppm.

^{13}C NMR (126 MHz, Chloroform-*d*) δ 163.4, 155.2, 146.0, 144.2, 143.5, 143.4, 141.5, 130.2, 129.0, 128.4, 126.8, 125.83, 125.76, 123.7, 120.4, 109.2, 51.8, 48.2, 42.5, 39.3, 18.5 ppm.
HRMS (ESI, *m*/*z*): Calcd for $C_{23}H_{20}ON_2Na^+$ [M+Na]$^+$: 363.1468, found: 363.1469.
IR: 3566 (s), 2363 (s), 2157 (s), 1969 (s), 1684 (s), 1380 (s).
The major diastereoisomer:

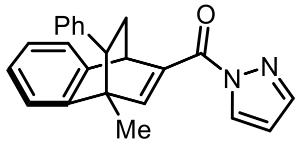

32b

((1S,4R,10S)-1-methyl-10-phenyl-1,4-dihydro-1,4-ethanonaphthalen-3-yl)(1H-pyrazol-1-yl)methanone (32b):
^{1}H NMR (500 MHz, Chloroform-*d*) δ 8.33 (d, *J* = 2.8 Hz, 1H), 7.98 (d, *J* = 1.9 Hz, 1H), 7.73 (d, *J* = 2.0 Hz, 1H), 7.41 (d, *J* = 8.5 Hz, 1H), 7.26 – 7.23 (m, 1H), 7.17 – 7.14 (m, 1H), 7.12 – 7.04 (m, 3H), 6.95 (d, *J* = 7.5 Hz, 1H), 6.45 – 6.38 (m, 1H), 6.30 (s, 2H), 4.85 – 4.83 (m, 1H), 2.99 – 2.96 (m, 1H), 2.59 – 2.43 (m, 1H), 1.72 – 1.63 (m, 1H), 1.47 (s, 3H) ppm.
^{13}C NMR (126 MHz, Chloroform-*d*) δ 163.2, 158.3, 144.4, 144.1, 141.7, 141.1, 140.2, 130.3, 129.0, 127.8, 126.6, 126.1, 125.8, 123.8, 122.9, 109.0, 49.6, 48.4, 41.9, 38.0, 18.6 ppm.
HRMS (ESI, *m*/*z*): Calcd for $C_{23}H_{20}ON_2Na^+$ [M+Na]$^+$: 363.1468, found: 363.1468.
IR: 2923 (s), 2362 (s), 2162 (s), 19776 (s), 1684 (s), 1380 (s).

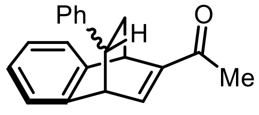

33

The title compound was prepared according to the general procedure using 1-(naphthalen-2-yl)ethan-1-one (34 mg, 0.20 mmol, 1.0 eq.), styrene (0.23 mL, 2.0 mmol, 10.0 eq.) and *fac*-Ir(*p*-CF_3-ppy)$_3$ (1.7 mg, 2.0 mol%) in DCM (2.0 mL, 0.10 M) under nitrogen atmosphere with blue LEDs irridiation for 24 hours. The d.r. value was determined through ^{1}H NMR of crude materials as 1.5:1. Afterwards, the crude product was transformed into the corresponding methyl ester, the crude was purified by FCC (Hex:EtOAc = 20:1 to 8:1) to afford **33** (46.6 mg, 85.0% total yield; minor: 18.6 mg, 34.0% yield, major: 28.0 mg, 51.0% yield) as two separable diastereoisomers as a colorless oil.
Note: The yield was reported as an average of two runs.
The minor diastereoisomer:

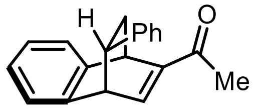

33a

1-(*(1S,4R,10S)*-10-phenyl-1,4-dihydro-1,4-ethanonaphthalen-3-yl)ethan-1-one (33a):

^{1}H NMR (500 MHz, Chloroform-*d*) δ 7.25 – 7.18 (m, 6H), 7.09 – 7.04 (m, 4H), 4.73 (s, 1H), 4.12 (d, *J* = 6.2 Hz, 1H), 3.06 – 3.03 (m, 1H), 2.24 (s, 3H), 2.09 – 2.04 (m, 1H), 1.71 – 1.67 (m, 1H).
^{13}C NMR (126 MHz, Chloroform-*d*) δ 194.9, 149.7, 144.9, 144.7, 143.4, 143.0, 128.6, 127.9, 126.7, 126.2, 125.8, 124.1, 123.0, 49.2, 45.6, 38.8, 34.9, 25.4 ppm.
HRMS (APCI, *m/z*): Calcd for $C_{20}H_{19}O^+$ [M+H]$^+$: 275.1430, found: 275.1429.
IR: 2955 (s), 1664 (s), 1372 (s), 756 (s), 700 (s).
The major diastereoisomer:

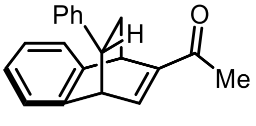

33b

1-(*(1S,4R,10R)*-10-phenyl-1,4-dihydro-1,4-ethanonaphthalen-3-yl)ethan-1-one (33b):
^{1}H NMR (500 MHz, Chloroform-*d*) δ 7.58 (d, *J* = 6.2 Hz, 1H), 7.37 (d, *J* = 7.3 Hz, 1H), 7.21 (t, *J* = 7.4 Hz, 1H), 7.10 – 7.04 (m, 4H), 6.87 (d, *J* = 7.3 Hz, 1H), 6.46 (d, *J* = 6.1 Hz, 2H), 4.80 (s, 1H), 3.96 (d, *J* = 6.4 Hz, 1H), 3.19 – 3.16 (m, 1H), 2.31 (s, 3H), 2.14 – 2.08 (m, 1H), 1.61 – 1.58 (m, 1H).
^{13}C NMR (126 MHz, Chloroform-*d*) δ 195.1, 148.3, 147.4, 144.2, 143.5, 138.8, 128.09, 128.06, 126.5, 126.4, 126.2, 125.5, 123.2, 49.6, 44.0, 38.3, 34.4, 25.3 ppm.
HRMS (APCI, *m/z*): Calcd for $C_{20}H_{17}O^+$ [M-H]$^+$: 273.1274, found: 273.1274.
IR: 2956 (s), 1662 (s), 1371 (s), 753 (s), 701 (s).

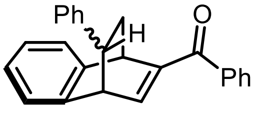

34

The title compound was prepared according to the general procedure using naphthalen-2-yl(phenyl)methanone (46.4 mg, 0.200 mmol, 1.00 eq.), styrene (0.23 mL, 2.0 mmol, 10 eq.) and *fac*-Ir(*p*-CF_3-ppy)$_3$ (1.7 mg, 2.0 mol%) in DCM (2.0 mL, 0.10 M) under nitrogen atmosphere with blue LEDs irridiation for 24 hours. The d.r. value was determined through ^{1}H NMR of crude materials as 1.3:1. Afterwards, the crude product was transformed into the corresponding methyl ester, the crude was purified by FCC (Hex:EtOAc = 10:1 to 5:1) to afford **34** (49.0 mg, 73.0% total yield; minor: 21.3 mg, 31.7% yield, major: 27.7 mg, 41.3% yield) as two separable diastereoisomers as a colorless oil.
Note: The yield was reported as an average of two runs.
The minor diastereoisomer:

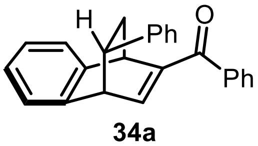

34a

Phenyl(*(1S,4R,10S)*-10-phenyl-1,4-dihydro-1,4-ethanonaphthalen-3-yl)methanone (34a):

^{1}H NMR (500 MHz, Chloroform-*d*) δ 7.58 (d, *J* = 7.6 Hz, 2H), 7.42 (t, *J* = 7.4 Hz, 1H), 7.32-7.28 (m, 3H), 7.23 – 7.17 (m, 3H), 7.12-7.09 (m, 3H), 7.06 (d, *J* = 7.6 Hz, 2H), 6.97 (d, *J* = 6.3 Hz, 1H), 4.80 (s, 1H), 4.15 (d, *J* = 6.1 Hz, 1H), 3.09 – 3.07 (m, 1H), 2.17 – 2.12 (m, 1H), 1.90 – 1.85 (m, 1H).
^{13}C NMR (126 MHz, Chloroform-*d*) δ 193.2, 148.3, 147.3, 144.6, 143.3, 143.1, 137.8, 132.1, 129.4, 128.6, 128.3, 127.9, 126.6, 126.2, 125.9, 124.1, 123.1, 49.5, 45.9, 40.9, 35.0 ppm.
HRMS (APCI, *m*/*z*): Calcd for $C_{25}H_{19}O^+$ [M-H]$^+$: 335.1430, found: 335.1431.
IR: 2363 (m), 1639 (s), 1474 (m), 1285 (s), 757 (s).
The major diastereoisomer:

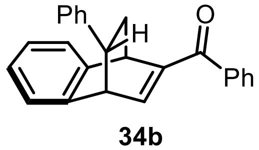

34b

Phenyl*((1S,4R,10R)*-10-phenyl-1,4-dihydro-1,4-ethanonaphthalen-3-yl)methanone (34b):
^{1}H NMR (500 MHz, Chloroform-*d*) δ 7.68 (d, *J* = 7.6 Hz, 2H), 7.54 (t, *J* = 7.4 Hz, 1H), 7.45 – 7.42 (m, 3H), 7.35 (d, *J* = 6.5 Hz, 1H), 7.28 - 7.27 (m, 1H), 7.13 – 7.10 (m, 4H), 6.91 (d, *J* = 7.4 Hz, 1H), 6.48 (d, *J* = 6.1 Hz, 2H), 4.89 (s, 1H), 4.00 (d, *J* = 6.3 Hz, 1H), 3.24 – 3.20 (m, 1H), 2.33 – 2.27 (m, 1H), 1.71 – 1.67 (m, 1H).
^{13}C NMR (126 MHz, Chloroform-*d*) δ 193.6, 150.0, 146.9, 144.2, 143.5, 138.7, 137.9, 132.1, 129.4, 128.3, 128.09, 128.08, 126.5, 126.4, 126.2, 125.6, 123.2, 49.9, 44.3, 40.4, 34.7 ppm.
HRMS (APCI, *m*/*z*): Calcd for $C_{25}H_{21}O^+$ [M+H]$^+$: 337.1587, found: 337.1588.
IR: 2366 (m), 1639 (s), 1458 (m), 1319 (s), 701 (s).

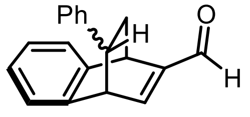

35

The title compound was prepared according to the general procedure using 2-naphthaldehyde (31.2 mg, 0.200 mmol, 1.00 eq.), styrene (0.23 mL, 2.0 mmol, 10 eq.) and *fac*-Ir(*p*-CF_3-ppy)$_3$ (1.7 mg, 2.0 mol%) in DCM (2.0 mL, 0.10 M) under nitrogen atmosphere with blue LEDs irridiation for 24 hours. The d.r. value was determined through ^{1}H NMR of crude materials as 1.1:1. Afterwards, the crude product was transformed into the corresponding methyl ester, the crude was purified by FCC (Hex:EtOAc = 50:1 to 20:1) to afford **35** (26 mg, 50% total yield; minor: 12.4 mg, 23.8% yield, major: 13.6 mg, 26.2% yield) as two separable diastereoisomers as a colorless oil.

Note: The yield was reported as an average of two runs.

The minor diastereoisomer:

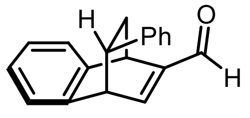

35a

***(1S,4R,10S)*-10-phenyl-1,4-dihydro-1,4-ethanonaphthalene-3-carbaldehyde (35a):**
^{1}H NMR (500 MHz, Chloroform-*d*) δ 9.60 (s, 1H), 7.38 – 7.27 (m, 5H), 7.22 – 7.12 (m, 5H), 4.71 (s, 1H), 4.30 (d, *J* = 6.3 Hz, 1H), 3.19 – 3.16 (m, 1H), 2.22 – 2.16 (m, 1H), 1.80 – 1.75 (m, 1H).
^{13}C NMR (126 MHz, Chloroform-*d*) δ 188.4, 153.6, 151.0, 144.3, 143.1, 142.3, 128.7, 127.8, 126.8, 126.4, 125.9, 124.1, 123.2, 49.5, 45.9, 37.5, 34.9 ppm.
HRMS (APCI, *m*/*z*): Calcd for $C_{19}H_{15}O^+$ $[M-H]^+$: 259.1117, found: 259.1119.
IR: 2945 (s), 1677 (s), 1155 (s), 757 (s), 700 (s).
The major diastereoisomer:

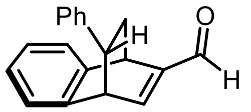

35b

***(1S,4R,10R)*-10-phenyl-1,4-dihydro-1,4-ethanonaphthalene-3-carbaldehyde (35b):**
^{1}H NMR (500 MHz, Chloroform-*d*) δ 9.47 (s, 1H), 7.51 (d, *J* = 6.4 Hz, 1H), 7.27 (d, *J* = 7.4 Hz, 1H), 7.12 (t, *J* = 7.5 Hz, 1H), 7.00 – 6.95 (m, 4H), 6.78 (d, *J* = 7.3 Hz, 1H), 6.36 (d, *J* = 6.1 Hz, 2H), 4.61 (s, 1H), 3.94 (d, *J* = 6.4 Hz, 1H), 3.11 – 3.08 (m, 1H), 2.05 – 1.99 (m, 1H), 1.55 – 1.51 (m, 1H).
^{13}C NMR (126 MHz, Chloroform-*d*) δ 188.5, 155.8, 149.7, 143.5, 143.1, 138.5, 128.2, 128.1, 126.6, 126.4, 125.7, 123.3, 49.9, 44.0, 37.1, 34.3 ppm.
HRMS (APCI, *m*/*z*): Calcd for $C_{19}H_{15}O^+$ $[M-H]^+$: 259.1117, found: 259.1121.
IR: 2960 (s), 1675 (s), 1162 (s), 754 (s), 720 (s).

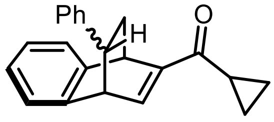

36

The title compound was prepared according to the general procedure using cyclopropyl(naphthalen-2-yl)methanone (39.2 mg, 0.200 mmol, 1.00 eq.), styrene (0.23 mL, 2.0 mmol, 10 eq.) and *fac*-Ir(*p*-CF_3-ppy)$_3$ (1.7 mg, 2.0 mol%) in DCM (2.0 mL, 0.10 M) under nitrogen atmosphere with blue LEDs irridiation for 24 hours. The d.r. value was determined through ^{1}H NMR of crude materials as 1.4:1. Afterwards, the crude product was transformed into the corresponding methyl ester, the crude was purified by FCC (Hex:EtOAc = 20:1 to 10:1) to afford **36** (46.2 mg, 77.0% total yield; minor: 19.3 mg, 32.0% yield, major: 26.9 mg, 45.0% yield) as two separable diastereoisomers as a colorless oil.
Note: The yield was reported as an average of two runs.
The minor diastereoisomer:

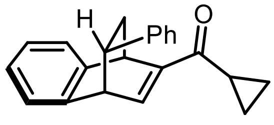

36a

Cyclopropyl(*(1S,4R,10S)*-10-phenyl-1,4-dihydro-1,4-ethanonaphthalen-3-yl)methanone (36a):

^{1}H NMR (500 MHz, Chloroform-*d*) δ 7.35 (d, *J* = 6.3 Hz, 1H), 7.22-7.17 (m, 4H), 7.12 (t, *J* = 7.3 Hz, 1H), 7.06 – 7.05 (m, 4H), 4.70 (s, 1H), 4.12 (d, *J* = 5.9 Hz, 1H), 3.05 – 3.03 (m, 1H), 2.27 – 2.23 (m, 1H), 2.08 – 2.02 (m, 1H), 1.71 – 1.66 (m, 1H), 1.10 – 1.05 (m, 1H), 0.97 – 0.92 (m, 1H), 0.85 – 0.80 (m, 1H), 0.77 – 0.72 (m, 1H).
^{13}C NMR (126 MHz, Chloroform-*d*) δ 196.9, 150.1, 144.8, 143.6, 143.4, 143.1, 128.6, 128.0, 126.6, 126.1, 125.7, 124.1, 122.9, 49.3, 45.7, 39.3, 35.0, 16.1, 11.1, 11.0 ppm.
HRMS (APCI, *m*/*z*): Calcd for $C_{22}H_{21}O^{+}$ $[M+H]^{+}$: 301.1587, found: 301.1587.
IR: 2363 (s), 1650 (s), 1607 (s), 1397 (s), 757 (s).
The major diastereoisomer:

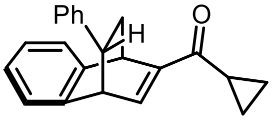

36b

Cyclopropyl(*(1S,4R,10R)*-10-phenyl-1,4-dihydro-1,4-ethanonaphthalen-3-yl)methanone (36b):
^{1}H NMR (500 MHz, Chloroform-*d*) δ 7.72 (d, *J* = 6.5 Hz, 1H), 7.37 (d, *J* = 7.3 Hz, 1H), 7.22 (t, *J* = 7.4 Hz, 1H), 7.10 – 7.05 (m, 4H), 6.89 (d, *J* = 7.3 Hz, 1H), 6.49 – 6.47 (m, 2H), 4.80 (s, 1H), 4.00 (d, *J* = 6.5 Hz, 1H), 3.24 – 3.20 (m, 1H), 2.39 – 2.34 (m, 1H), 2.17 – 2.12 (m, 1H), 1.62 – 1.59 (m, 1H), 1.17 – 1.12 (m, 1H), 1.08 – 1.03 (m, 1H), 0.95 – 0.90 (m, 1H), 0.88 – 0.83 (m, 1H).
^{13}C NMR (126 MHz, Chloroform-*d*) δ 197.1, 148.6, 146.1, 144.3, 143.6, 139.0, 128.1, 126.4, 126.3, 126.1, 125.4, 123.2, 49.6, 44.1, 38.8, 34.4, 16.0, 11.11, 11.07 ppm.
HRMS (APCI, *m*/*z*): Calcd for $C_{22}H_{21}O^{+}$ $[M+H]^{+}$: 301.1587, found: 301.1588.
IR: 2956 (s), 1649 (s), 1396 (s), 957 (s), 701 (s).

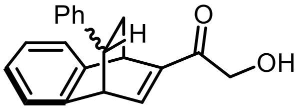

37

The title compound was prepared according to the general procedure using 2-hydroxy-1-(naphthalen-2-yl)ethan-1-one (37.2 mg, 0.200 mmol, 1.00 eq.), styrene (0.23 mL, 2.0 mmol, 10 eq.) and *fac*-Ir(*p*-CF_3-ppy)$_3$ (1.7 mg, 2.0 mol%) in DCM (2.0 mL, 0.10 M) under nitrogen atmosphere with blue LEDs irridiation for 24 hours. The d.r. value was determined through ^{1}H NMR of crude materials as 1.3:1. Afterwards, the crude product was transformed into the corresponding methyl ester, the crude was purified by FCC (Hex:EtOAc = 10:1 to 5:1) to afford **37** (43.5 mg, 75% total yield; minor: 18.9 mg, 32.6% yield, major: 24.6 mg, 42.4% yield) as two separable diastereoisomers as a colorless oil.
Note: The yield was reported as an average of two runs.
The minor diastereoisomer:

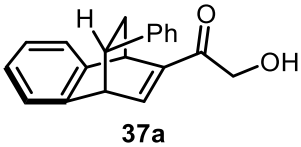

37a

2-Hydroxy-1-((1S,4R,10S)-10-phenyl-1,4-dihydro-1,4-ethanonaphthalen-3-

yl)ethan-1-one (37a):

^{1}H NMR (500 MHz, Chloroform-*d*) δ 7.27 – 7.20 (m, 4H), 7.16 – 7.07 (m, 3H), 7.03 – 6.97 (m, 3H), 4.73 (s, 1H), 4.58 (dd, *J* = 18.0, 4.8 Hz, 1H), 4.38 (dd, *J* = 18.0, 4.2 Hz, 1H), 4.15 (d, *J* = 6.4 Hz, 1H), 3.23 (t, *J* = 5.0 Hz, 1H), 3.09 – 3.06 (m, 1H), 2.13 – 2.09 (m, 1H), 1.76 – 1.72 (m, 1H).

^{13}C NMR (126 MHz, Chloroform-*d*) δ 195.0, 145.9, 145.5, 144.3, 142.8, 142.3, 128.8, 127.8, 126.9, 126.4, 126.0, 124.1, 123.2, 64.5, 49.2, 45.6, 39.1, 34.9 ppm.

HRMS (ESI, *m*/*z*): Calcd for $C_{20}H_{18}O_2Na^+$ [M+Na]$^+$: 313.1199, found:313.1199.

IR: 3024 (s), 1667 (s), 1085 (s), 756 (s), 700 (s).

The major diastereoisomer:

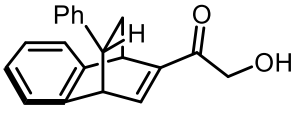

37b

2-Hydroxy-1-((1S,4R,10R)-10-phenyl-1,4-dihydro-1,4-ethanonaphthalen-3-yl)ethan-1-one (37b):

^{1}H NMR (500 MHz, Chloroform-*d*) δ 7.61 (d, *J* = 6.5 Hz, 1H), 7.38 (d, *J* = 7.3 Hz, 1H), 7.23 (t, *J* = 7.4 Hz, 1H), 7.13 – 7.06 (m, 4H), 6.87 (d, *J* = 7.3 Hz, 1H), 6.45 (d, *J* = 6.6 Hz, 2H), 4.80 (s, 1H), 4.66 (dd, *J* = 17.8, 4.2 Hz, 1H), 4.46 (dd, *J* = 17.9, 3.8 Hz, 1H), 3.99 (d, *J* = 6.2 Hz, 1H), 3.29 (s, 1H), 3.20 – 3.16 (m, 1H), 2.18 – 2.12 (m, 1H), 1.66 – 1.61 (m, 1H).

^{13}C NMR (126 MHz, Chloroform-*d*) δ 195.3, 147.7, 144.5, 143.5, 143.1, 138.3, 128.2, 128.0, 126.64, 126.62, 126.3, 125.8, 123.2, 64.4, 49.5, 43.8, 38.7, 34.3 ppm.

HRMS (ESI, *m*/*z*): Calcd for $C_{20}H_{18}O_2Na^+$ [M+Na]$^+$: 313.1199, found: 313.1199.

IR: 3024 (s), 1666 (s), 1353 (s), 1081 (s), 701 (s).

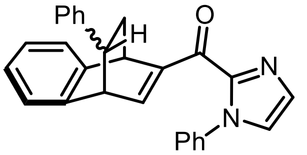

38

The title compound was prepared according to the general procedure using naphthalen-2-yl(1-phenyl-1H-imidazol-2-yl)methanone (59.6 mg, 0.200 mmol, 1.00 eq.), styrene (0.23 mL, 2.0 mmol, 10 eq.) and *fac*-Ir(*p*-CF_3-ppy)$_3$ (1.7 mg, 2.0 mol%) in DCM (2.0 mL, 0.1 M) under nitrogen atmosphere with blue LEDs irridiation for 24 hours. The d.r. value was determined through ^{1}H NMR of crude materials as 1:1. Afterwards, the crude product was transformed into the corresponding methyl ester, the crude was purified by FCC (Hex:EtOAc = 10:1 to 3:1) to afford **38** (66 mg, 82% total yield; minor: 33 mg, 41% yield, major: 33 mg, 41% yield) as two separable diastereoisomers as a colorless oil.

Note: The yield was reported as an average of two runs.

The minor diastereoisomer:

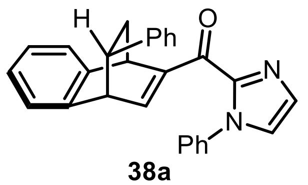

38a

(*(1S,4R,10S)*-10-phenyl-1,4-dihydro-1,4-ethanonaphthalen-3-yl)(1-phenyl-1H-imidazol-2-yl)methanone (38a):

^{1}H NMR (500 MHz, Chloroform-*d*) δ 8.07 (d, *J* = 6.3 Hz, 1H), 7.39 - 7.34 (m, 3H), 7.22 – 7.02 (m, 13H), 4.72 (s, 1H), 4.21 (d, *J* = 6.4 Hz, 1H), 3.05 – 3.02 (m, 1H), 2.11 – 2.05 (m, 1H), 1.78 – 1.74 (m, 1H).

^{13}C NMR (126 MHz, Chloroform-*d*) δ 179.3, 150.2, 147.7, 144.9, 143.4, 143.2, 143.0, 138.9, 129.5, 129.2, 128.7, 128.6, 128.1, 126.5, 126.3, 125.9, 125.9, 125.7, 123.9, 123.2, 49.6, 46.1, 40.0, 35.6 ppm.

HRMS (ESI, *m*/*z*): Calcd for $C_{28}H_{23}ON_2^+$ [M+H]$^+$: 403.1805, found: 403.1806.

IR: 1635 (s), 1493 (s), 1406 (s), 1129 (s), 756 (s).

The major diastereoisomer:

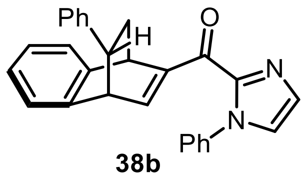

38b

(*(1S,4R,10R)*-10-phenyl-1,4-dihydro-1,4-ethanonaphthalen-3-yl)(1-phenyl-1H-imidazol-2-yl)methanone (38b):

^{1}H NMR (500 MHz, Chloroform-*d*) δ 8.42 (d, *J* = 6.6 Hz, 1H), 7.40 – 7.37 (m, 3H), 7.25 – 7.19 (m, 4H), 7.12 – 7.09 (m, 2H), 7.02 - 6.95 (m, 4H), 6.79 (d, *J* = 7.3 Hz, 1H), 6.40 – 6.38 (m, 2H), 4.74 (s, 1H), 3.98 (d, *J* = 6.6 Hz, 1H), 3.25 – 3.21 (m, 1H), 2.13 (t, *J* = 11.4 Hz, 1H), 1.55 – 1.49 (m, 1H).

^{13}C NMR (126 MHz, Chloroform-*d*) δ 179.1, 153.1, 146.0, 144.2, 143.8, 143.2, 138.8, 138.7, 129.3, 129.2, 128.7, 128.1, 128.0, 126.4, 126.3, 126.24, 126.17, 125.9, 125.5, 123.0, 50.1, 43.8, 39.2, 34.5 ppm.

HRMS (ESI, *m*/*z*): Calcd for $C_{28}H_{23}ON_2^+$ [M+H]$^+$: 403.1805, found: 403.1807.

IR: 1639 (s), 1493 (s), 1407 (s), 1129 (s), 760 (s).

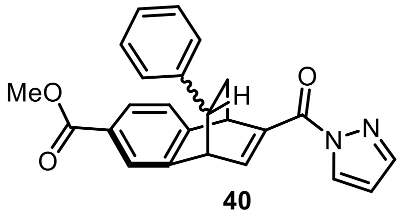

40

The title compound was prepared according to the general procedure using methyl 6-(1H-pyrazole-1-carbonyl)-2-naphthoate (56 mg, 0.20 mmol, 1.0 eq.), styrene (0.23 mL, 2.0 mmol, 10 eq.) and *fac*-Ir(*p*-CF_3-ppy)$_3$ (1.7 mg, 2.0 mol%) in DCM (2.0 mL, 0.10 M) under nitrogen atmosphere with blue LEDs irridiation for 24 hours. The d.r. value was determined through ^{1}H NMR of crude materials as 1.5:1. Afterwards, the

crude product was transformed into the corresponding methyl ester, the crude was purified by FCC (Hex:EtOAc = 10:1 to 3:1) to afford **40** (62.2 mg, 81.0% total yield; minor: 20.7 mg, 27.0% yield, 41.5 mg, 54.0% yield) as two separable diastereoisomers as a colorless oil.

Note: The yield was reported as an average of two runs.

The minor diastereoisomer:

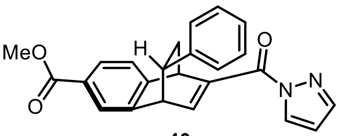

40a

Methyl *(1S,4R,10S)*-10-phenyl-3-(1H-pyrazole-1-carbonyl)-1,4-dihydro-1,4-ethanonaphthalene-7-carboxylate (40a):

^{1}H NMR (500 MHz, Chloroform-*d*) δ 8.27 (s, 1H), 7.88 (s, 2H), 7.81 (d, *J* = 7.5 Hz, 1H), 7.63 (s, 1H), 7.31 (d, *J* = 7.7 Hz, 1H), 7.21 – 7.18 (m, 2H), 7.14 (s, 3H), 6.39 (s, 1H), 4.85 (s, 1H), 4.29 (d, *J* = 6.3 Hz, 1H), 3.84 (s, 3H), 3.08 – 3.05 (m, 1H), 2.17 – 2.12 (m, 1H), 2.06 – 2.02 (m, 1H).

^{13}C NMR (126 MHz, Chloroform-*d*) δ 167.3, 163.2, 150.1, 148.1, 144.3, 144.1, 143.3, 140.7, 130.1, 128.7, 128.1, 128.0, 126.8, 124.2, 123.9, 109.3, 52.2, 49.5, 45.7, 42.6, 35.2 ppm.

HRMS (ESI, *m*/*z*): Calcd for $C_{24}H_{20}O_3N_2Na^+$ [M+Na]$^+$: 407.1366, found: 407.1365.

IR: 2955 (s), 1700 (s), 1385 (s), 1270(s), 700 (s).

The major diastereoisomer:

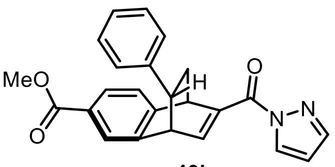

40b

Methyl *(1S,4R,10R)*-10-phenyl-3-(1H-pyrazole-1-carbonyl)-1,4-dihydro-1,4-ethanonaphthalene-7-carboxylate (40b):

^{1}H NMR (500 MHz, Chloroform-*d*) δ 8.34 – 8.31 (m, 2H), 7.96 (d, *J* = 7.5 Hz, 1H), 7.73 (s, 1H), 7.58 (s, 1H), 7.47 (d, *J* = 7.7 Hz, 1H), 7.11 – 7.09 (m, 3H), 6.50 (d, *J* = 7.0 Hz, 2H), 6.46 (s, 1H), 4.95 (s, 1H), 4.16 (d, *J* = 6.6 Hz, 1H), 3.85 (s, 3H), 3.31 – 3.27 (m, 1H), 2.45 – 2.39 (m, 1H), 1.70 – 1.66 (m, 1H).

^{13}C NMR (126 MHz, Chloroform-*d*) δ 167.3, 163.0, 153.2, 149.2, 144.2, 142.9, 138.9, 138.7, 130.2, 128.3, 127.9, 127.7, 127.3, 126.7, 123.1, 109.2, 52.1, 50.0, 43.5, 41.9, 34.2 ppm.

HRMS (ESI, *m*/*z*): Calcd for $C_{24}H_{20}O_3N_2Na^+$ [M+Na]$^+$: 407.1366, found: 407.1366.

IR: 2951 (s), 1718 (s), 1380 (s), 1269 (s), 1196 (s).

6. Relative Reactivities

6.1 Internal competition experiment

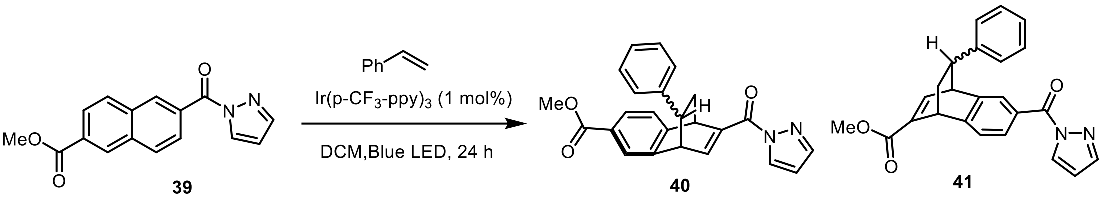

81% yield, dr=2:1

~8% NMR yield

An oven-dried 4 mL screw-cap vial equipped with a stir bar and a septum was cooled under vacuum. After evacuated/backfilled with N_2 (x 3), the vial was transferred into glovebox and charged with Ir(*p*-CF_3-ppy)$_3$ (1.0 mg, 1.0 mol%) and acyl pyrazole **39** (28 mg, 0.10 mmol, 1.0 eq.). After moved out, styrene (115 μL, 1.00 mmol, 10.0 eq.) and dry DCM (1 mL) were sequentially added to the vial. Then the septum was replaced with a cap. The vial was capped and the reaction was stirred under blue LED irradiation with a cooling fan to keep room temperature. After 24 h, the solution was concentrated by rotary evaporation. 1,1,2,2-Tetrachlorethane (10.5 μL, 0.200 mmol) was added to the residue for crude ^{1}H-NMR to determine d.r..

6.2 External competition experiment

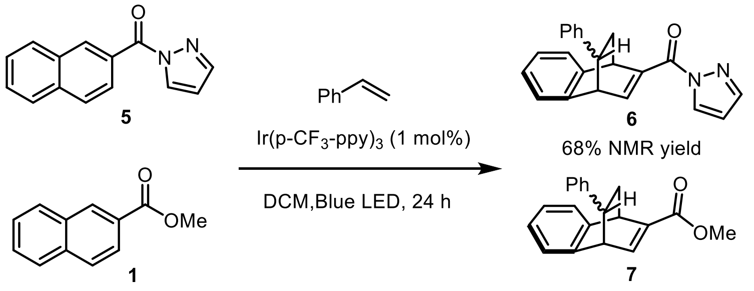

68% NMR yield

10% NMR yield

An oven-dried 4 mL screw-cap vial equipped with a stir bar and a septum was cooled under vacuum. After evacuated/backfilled with N_2 (x 3), the vial was transferred into glovebox and charged with Ir(*p*-CF_3-ppy)$_3$ (1.0 mg, 1.0 mol%) and acyl pyrazole **5** (22 mg, 0.10 mmol, 1.0 eq.) and ester 1 (18.6 mg, 0.100 mmol, 1.0 eq.). After moved out, styrene (115 μL, 1.00 mmol, 10.0 eq.) and dry DCM (1 mL) were sequentially added to the vial. Then the septum was replaced with a cap. The vial was capped and the reaction was stirred under blue LED irradiation with a cooling fan to keep room temperature. After 24 h, the solution was concentrated by rotary evaporation. 1,1,2,2-Tetrachlorethane (10.5 μL, 0.200 mmol) was added to the residue for crude ^{1}H-NMR.

6.3 Comparison of light sources

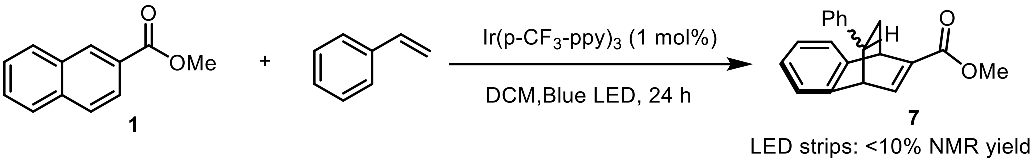

LED strips: <10% NMR yield

IPR: 90% NMR yield

An oven-dried 4 mL screw-cap vial equipped with a stir bar and a septum was cooled under vacuum. After evacuated/backfilled with N_2 (x 3), the vial was transferred into glovebox and charged with Ir(*p*-CF_3-ppy)$_3$ (1.0 mg, 1.0 mol%) and ester **1** (18.6

mg, 0.10 mmol, 1.0 eq.). After moved out, styrene (115 μL, 1.0 mmol, 10 eq.) and dry DCM (2 mL) were sequentially added to the vial. Then the septum was replaced with a cap. The vial was capped and the reaction was stirred under blue LED irradiation (LED strips or IPR see following pictures for set up) with a cooling fan to keep room temperature. After 24 h, the solution was concentrated by rotary evaporation. 1,1,2,2-Tetrachlorethane (10.5 μL, 0.10 mmol) was added to the residue for crude ^{1}H-NMR.

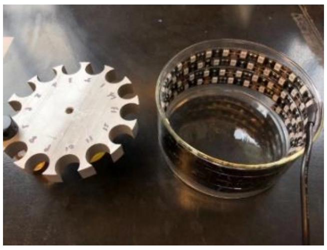

LED strips

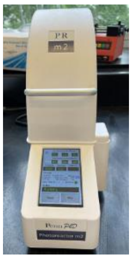

IPR

7. Gram scale synthesis

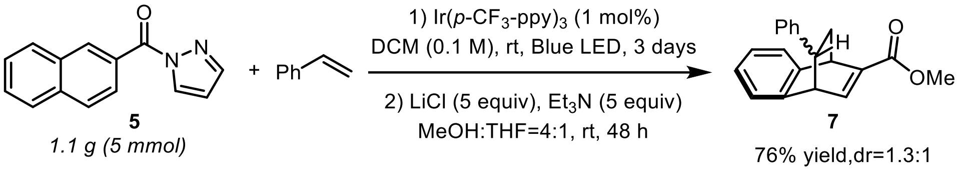

An oven-dried 100 mL flask equipped with a stir bar was cooled under vacuum. After evacuated/backfilled with N_2 (x 3), the vial was transferred into the glovebox and charged with Ir(*p*-CF_3-ppy)$_3$ (50 mg, 1.0 mol%) and acyl pyrazole (1.1 g, 5.0 mmol, 1.0 eq.) After moved out, styrene (5.7 mL, 50 mmol, 10 eq.) and dry DCM (50 mL) were sequentially added to the flask. Then the septum was replaced with a cap. The vial was capped and the reaction was stirred under blue LED irradiation with a cooling fan to keep room temperature (see following pictures for set up). After 3 days, the solution was concentrated by rotary evaporation, the residue for crude ^{1}H-NMR to determine rr and dr. Afterward, the crude product was transformed into the corresponding methyl ester through the following procedure: The crude mixture was transferred into a 10 mL round-bottom flask and dissolved in anhydrous methanol (100 mL) and THF (25 mL). To this solution was added LiCl (1.1 g, 25 mmol, 5.0 eq.) and Et_3N (3.5 mL, 25 mmol, 5.0 eq.) at room temperature. After 48 hours of stirring, the reaction mixture was diluted with CH_2Cl_2. The combined organic solution were concentrated under reduced pressure and the crude was purified by FCC (Hex: EtOAc = 30:1 to 10:1) to afford to give corresponding cycloadducts (1.10 g, 76.0% total yield; minor: 478 mg, 33.0% yield, major: 622 mg, 43.0% yield), single crystals of products were obtained by slowly diffusion of DCM solution.

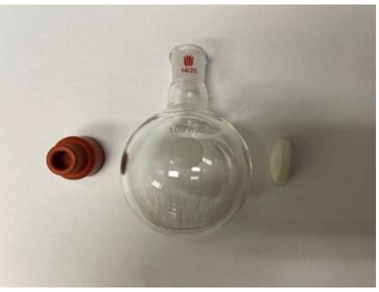

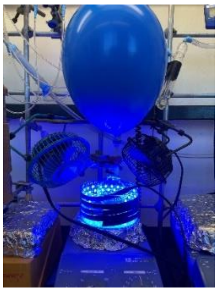

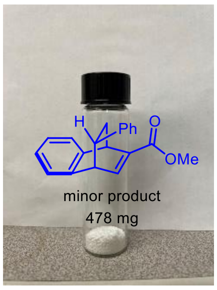

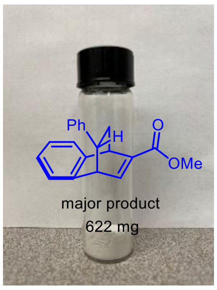

Alternatively, the reaction was scaled up with Vapourtec E-series Flow Chemistry System. Blue LED λ= 450 nm, flow rate = 200 µL/min, retention time = 50 min, total reaction time =240 min, 1.06 g, 73.0% total yield; minor: 461 mg, 31.7% yield, major: 599 mg, 41.3% yield.

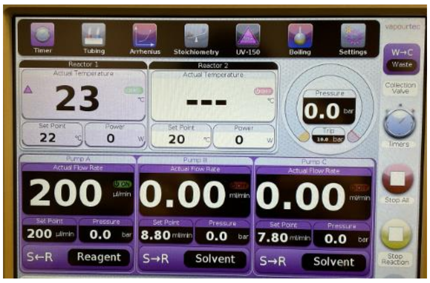

8. Further Derivatizations

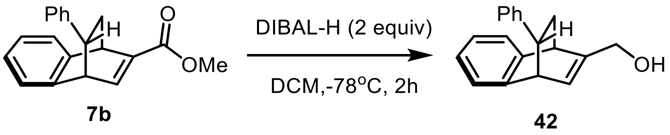

((1S,4R,10R)-10-phenyl-1,4-dihydro-1,4-ethanonaphthalen-3-yl)methanol (41): An oven-dried 50 mL flask equipped with a stir bar was cooled under vacuum. **7b** (58 mg, 0.20 mmol, 1.0 eq.) was added to the flask, then anhydrous DCM (2 mL) was added. After that diisobutyl aluminium hydride (1.0 M in DCM, 0.44 mL, 0.44 mmol, 2.0 eq.) was added at -78 °C in a dry ice/acetone bath. The reaction mixture was stirred at same temperature for 2 hours beforequenched with sat. NH_4Cl (aq.) (10 mL) The aqueous layer was extracted with DCM (3 × 10 mL). The combined organic layers were washed with brine (15 mL), dried over with anhydrous Na_2SO_4, filtered and concentrated by rotary evaporation. The residue was purified by FCC (Hex: EtOAc = 20:1 to 10:1) to afford alcohol **42** (46 mg, 88% yield) as a colorless oil [28].

^{1}H NMR (500 MHz, Chloroform-*d*) δ 7.32 (d, *J* = 7.3 Hz, 1H), 7.18 (t, *J* = 7.5 Hz, 1H), 7.11 – 7.03 (m, 4H), 6.84 (d, *J* = 7.2 Hz, 1H), 6.51 – 6.47 (m, 3H), 4.31 (s, 2H), 4.02 (s, 1H), 3.80 (d, *J* = 6.4 Hz, 1H), 3.22 – 3.18 (m, 1H), 2.23 – 2.18 (m, 1H), 1.63 – 1.59 (m, 1H), 1.55 (s, 1H).

^{13}C NMR (126 MHz, Chloroform-*d*) δ 148.0, 144.7, 144.6, 141.2, 130.2, 128.0, 127.9, 126.1, 125.8, 125.6, 125.2, 122.4, 63.8, 48.5, 45.2, 42.4, 35.1 ppm.

HRMS (APCI, *m/z*): Calcd for $C_{19}H_{19}O^+$ $[M+H]^+$: 263.1430, found:263.1430.

IR: 2946 (s), 1600 (s), 1458 (s), 752 (s), 700 (s).

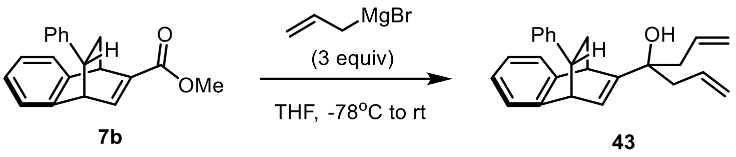

4-((1S,4R,10R)-10-phenyl-1,4-dihydro-1,4-ethanonaphthalen-3-yl) hepta-1,6-dien-4-ol (42): An oven-dried 50 mL flask equipped with a stir bar was cooled under vacuum. **7b** (58 mg, 0.20 mmol, 1.0 eq.) and THF (2 mL) were added.After that, 2.0 M allylmagnesium bromide in THF solution (0.3 mL, 0.6 mmol, 3.0 equiv) was added at 0 °C in a dry ice/acetone bath under argon atmosphere. Then the reaction at room temperature for 2 h, quenched with sat. NH_4Cl (aq.) (10 mL), extracted with DCM (3 × 10 mL). The combined organic layers were washed with brine (10 mL), dried over with anhydrous Na_2SO_4, filtered and concentrated by rotary evaporation. The residue was purified by FCC (Hex: EtOAc = 10:1 to 5:1) to afford tertiary alcohol **43** (56 mg, 83% yield)as a colorless oil[29].

^{1}H NMR (500 MHz, Chloroform-*d*) δ 7.19 (t, *J* = 7.1 Hz, 1H), 7.07 (t, *J* = 7.4 Hz, 1H), 7.01 – 6.99 (m, 3H), 6.94 (t, *J* = 7.4 Hz, 1H), 6.73 (d, *J* = 7.2 Hz, 1H), 6.41 – 6.38 (m, 3H), 5.72 – 5.63 (m, 1H), 5.29 – 5.20 (m, 1H), 5.06 – 5.03 (m, 2H), 4.98 – 4.90 (m, 2H), 4.00 (s, 1H), 3.71 (d, *J* = 6.5 Hz, 1H), 3.09 – 3.05 (m, 1H), 2.52 – 2.43 (m, 2H), 2.40 – 2.35 (m, 1H), 2.28 – 2.24 (m, 1H), 2.14 – 2.09 (m, 1H), 1.80 (s, 1H), 1.49 – 1.45 (m, 1H).

^{13}C NMR (126 MHz, Chloroform-*d*) δ 151.2, 145.1, 144.7, 141.1, 133.7, 133.5, 130.0, 128.0, 127.9, 126.1, 125.7, 125.5, 125.1, 122.0, 119.5, 118.8, 75.1, 48.5, 45.1, 43.5, 43.4, 42.1, 35.0 ppm.

HRMS (APCI, *m/z*): Calcd for $C_{25}H_{25}^+$ $[M-H_2O]^+$: 325.1951, found: 325.1952.

IR: 3071 (s), 3022 (s), 2948 (s), 1638 (s), 970 (s).

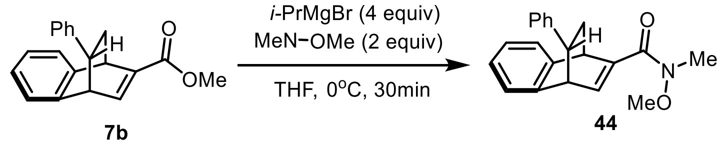

(1S,4R,10R)-N-methoxy-N-methyl-10-phenyl-1,4-dihydro-1,4 ethanonaphthalene-3-carboxamide (43): An oven-dried 50 mL flask equipped with a stir bar was cooled under vacuum. **7b** (58 mg, 0.20 mmol, 1.0 eq.) and *N*,*O*-dimethylhydroxyamine hydrochloride (39 mg, 0.40 mmol, 2.0 eq.) and dry THF (2 mL) were added.Then a solution of *i*-PrMgCl (0.4 mL, 2.0 M in THF, 0.8 mmol, 4 eq.) was slowly added at 0 °C in an ice/water bath. The mixture was stirred for 30 min. After completion, the reaction was quenched with sat. NH_4Cl (aq.) (10 mL) andextracted with DCM (3 × 10 mL). The combined organic layers were washed with brine (10 mL), dried over with anhydrous Na_2SO_4, filtered and concentrated by rotary evaporation. The residue was purified by FCC (Hex: EtOAc = 20:1 to 10:1) to afford amide **44** (51 mg, 80% yield)[30] as a colorless oil.

^{1}H NMR (500 MHz, Chloroform-*d*) δ 7.34 (d, *J* = 7.0 Hz, 2H), 7.19 (t, *J* = 7.4 Hz, 1H), 7.19 – 7.03 (m, 4H), 6.85 (d, *J* = 7.3 Hz, 1H), 6.47 – 6.45 (m, 2H), 4.54 (s, 1H), 3.91 (d, *J* = 6.6 Hz, 1H), 3.45 (s, 3H), 3.27 (s, 3H), 3.23 – 3.19 (m, 1H), 2.32 – 2.26 (m, 1H), 1.61 – 1.57 (m, 1H).

^{13}C NMR (126 MHz, Chloroform-*d*) δ 167.8, 144.5, 143.9, 141.9, 141.8, 139.3, 128.0, 128.0, 126.3, 126.0, 125.9, 125.3, 122.6, 61.0, 49.1, 44.1, 42.5, 34.9, 33.4 ppm.
HRMS (ESI, *m*/*z*): Calcd for $C_{21}H_{21}O_2NNa^+$ [M+Na]$^+$: 342.1465, found: 342.1465.
IR: 2959 (m), 1635 (s), 1474 (s), 1378 (s), 973 (s).

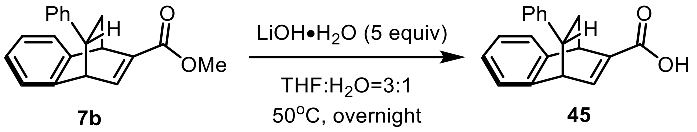

(1R,4S)-9-phenyl-1,4-dihydro-1,4-ethanonaphthalene-2-carboxylic acid (44): mL round bottom flask was charged with methyl ester **7b** (58 mg, 0.20 mmol, 1.0 eq.), LiOH·H2O (42 mg, 1 mmol, 5 eq.), and THF/H2O (3:1, 0.5 M). The reaction was stirred at 50 °C in oil bath overnight. After cooled down to room temperature and the reaction was acidified with 3 N HCl (10 mL). The aqueous layer was xtracted with DCM (3 × 10 mL). The combined organic layers were washed with brine (10 mL), dried over with anhydrous Na_2SO_4, filtered and concentrated by rotary evaporation. The residue was purified by FCC (Hex: EtOAc = 8:1 to 4:1) to afford unsaturated acid **45** (46 mg, 84% yield)[31] as colorless solid.
^{1}H NMR (500 MHz, Chloroform-*d*) δ 11.98 (s, 1H), 7.83 (d, *J* = 6.5 Hz, 1H), 7.39 (d, *J* = 7.3 Hz, 1H), 7.23 (t, *J* = 7.5 Hz, 1H), 7.11 – 7.06 (m, 4H), 6.87 (d, *J* = 7.3 Hz, 1H), 6.47 (d, *J* = 6.1 Hz, 2H), 4.65 (s, 1H), 3.99 (d, *J* = 6.2 Hz, 1H), 3.20 – 3.17 (m, 1H), 2.25 – 2.21 (m, 1H), 1.65 – 1.61 (m, 1H).
^{13}C NMR (126 MHz, Chloroform-*d*) δ 170.4, 150.0, 144.0, 143.4, 139.0, 138.4, 128.10, 128.07, 126.5, 126.4, 125.6, 122.9, 49.8, 43.7, 40.1, 34.6 ppm.
HRMS (APCI, *m*/*z*): Calcd for $C_{19}H_{15}O_2^+$ [M-H]$^+$: 275.1078, found: 275.1078.
IR: 3024 (s), 1619 (s), 1424 (s), 1284 (s), 757 (s).

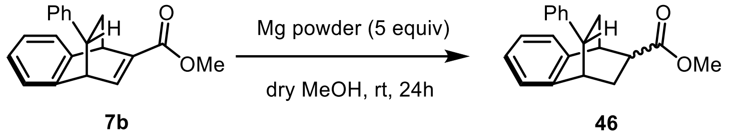

An oven-dried 50 mL flask equipped with a stir bar was cooled under vacuum. **7b** (58 mg, 0.20 mmol, 1.0 eq.), magnesium powder (48.6 mg, 2.00 mmol, 5.00 eq.) and 5 mL anhydrous MeOH were added.The reaction was stirred at room temperature for 24 h under an argon atmosphere. Then 3 N HCl (xx mL) was added carefully at room temperature until the excess magnesium was dissolved The reaction was extracted with DCM (3 × 10 mL). The combined organic layerw were washed with brine (10 mL), dried over with anhydrous Na_2SO_4, filtered and concentrated by rotary evaporation. The residue was purified by FCC (Hex: EtOAc = 20:1 to 10:1) to afford the title compound **46** as separable mixture of diastereomers (50.0 mg, 86.0% yield, dr=1.70:1; minor: 18.5 mg, 32.0% yield, major: 31.5 mg, 54.0% yield)[32] as a colorless oil.
The minor diastereoisomer:

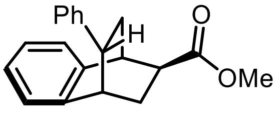

46a

Methyl (1S,3S,4S,10R)-10-phenyl-1,2,3,4-tetrahydro-1,4-ethanonaphthalene-3-carboxylate (46a):

^{1}H NMR (500 MHz, Chloroform-*d*) δ 7.19 (d, *J* = 9.3 Hz, 2H), 7.11 – 7.07 (m, 1H), 7.02 – 7.00 (m, 3H), 6.83 (d, *J* = 7.3 Hz, 1H), 6.48 – 6.47 (m, 2H), 3.73 (s, 3H), 3.41 (s, 1H), 3.23 – 3.20 (m, 1H), 2.95 (s, 1H), 2.58 – 2.54 (m, 1H), 2.43 – 2.39 (m, 1H), 2.30 – 2.25 (m, 1H), 1.67 – 1.61 (m, 1H), 1.40 – 1.35 (m, 1H).

^{13}C NMR (126 MHz, Chloroform-*d*) δ 175.9, 146.7, 143.0, 140.8, 128.0, 127.8, 126.6, 126.6, 126.4, 126.0, 123.6, 52.2, 43.3, 41.73, 41.69, 37.8, 32.3, 30.4 ppm.

HRMS (APCI, *m*/*z*): Calcd for $C_{20}H_{18}O_2^+$ $[M+H]^+$: 293.1536, found: 293.1536.

IR: 2946 (s), 1736 (s), 1492 (s), 1200 (m), 701 (s).

The major diastereoisomer:

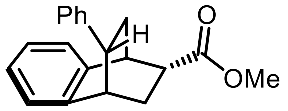

46b

Methyl (1S,4S,10R)-10-phenyl-1,2,3,4-tetrahydro-1,4-ethanonaphthalene-3-carboxylate (46b):

^{1}H NMR (500 MHz, Chloroform-*d*) δ 7.25-7.21 (m, 2H), 7.17 – 7.09 (m, 4H), 6.91 (d, *J* = 7.3 Hz, 1H), 6.60 – 6.58 (m, 2H), 3.58 (s, 3H), 3.54 (s, 1H), 3.20 – 3.16 (m, 1H), 3.04 (s, 1H), 2.98 – 2.95 (m, 1H), 2.43 – 2.37 (m, 1H), 2.25 – 2.20 (m, 1H), 1.91 – 1.86 (m, 1H), 1.60 – 1.58 (m, 1H).

^{13}C NMR (126 MHz, Chloroform-*d*) δ 175.2, 146.6, 140.6, 140.5, 128.1, 127.7, 126.57, 126.55, 126.0, 124.6, 51.9, 43.2, 41.9, 41.6, 37.7, 36.2, 31.5 ppm.

HRMS (APCI, *m*/*z*): Calcd for $C_{20}H_{18}O_2^+$ $[M+H]^+$: 293.1536, found: 293.1535.

IR: 2946 (s), 1736 (s), 1492 (s), 1200 (m), 701 (s).

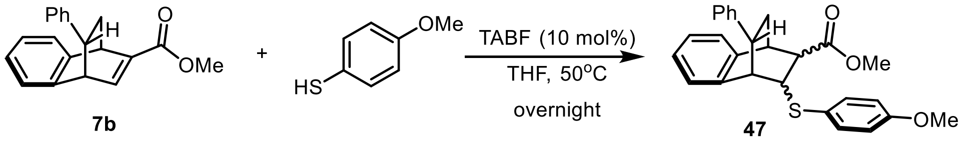

Methyl (1S,4S,10R)-2-((4-methoxyphenyl)thio)-10-phenyl-1,2,3,4-tetrahydro-1,4-ethanonaphthalene-3-carboxylate (47): An oven-dried 50 mL flask equipped with a stir bar was cooled under vacuum. **7b** (64 mg, 0.22 mmol, 1.1 eq.) was added to the flask.After evacuated/backfilled with N_2 (x 3), 4-methoxybenzenethiol (24.6 µL, 0.20 mmol, 1.00 eq.), and TBAF (20 µL, 0.020 mmol, 1.0 M in THF) were added. The reaction mixture was stirred at 50 °C in oil bath for 12 h. After completion, the reaction was quenched with sat. NH_4Cl (aq.) (10 mL), extracted with DCM (3 × 10 mL). The combined organic layers were washed with brine (10 mL), dried over with anhydrous Na_2SO_4, filtered and concentrated by rotary evaporation. The residue was purified by FCC (Hex: EtOAc = 20:1 to 10:1) to afford product **47** as a colorless oil (60 mg, 70% yield, dr=1:1)[33].

^{1}H NMR (500 MHz, Chloroform-*d*) δ 7.42 (d, *J* = 7.2 Hz, 2H, major), 7.32 – 6.97 (m, 14H, mix), 6.89 (d, *J* = 7.3 Hz, 1H, minor), 6.76 (t, *J* = 8.5 Hz, 4H, mix), 6.70 (d, *J* = 7.3 Hz, 1H, minor), 6.55 (d, *J* = 6.2 Hz, 2H, major), 6.41 – 6.39 (m, 2H, major), 4.07 –

4.05 (m, 1H, major), 3.94 – 3.91 (m, 1H, minor), 3.72 (s, 6H, mix), 3.69 (s, 3H, mix), 3.54 (s, 3H, mix), 3.48 (d, J = 6.9 Hz, 1H, minor), 3.42 (s, 2H), 3.16 – 3.13 (m, 1H, major), 2.96 (s, 1H, major), 2.83 (s, 1H, minor), 2.61 (d, J = 6.7 Hz, 1H, minor), 2.40 – 2.35 (m, 2H, mix), 2.22 – 2.17 (m, 1H, major), 1.56 – 1.52 (m, 1H, minor), 1.39 – 1.34 (m, 1H, major).

^{13}C NMR (126 MHz, Chloroform-*d*) δ 174.1(major), 173.5(minor), 159.9(minor), 159.7(major), 146.0(minor), 145.7(major), 141.6, 140.1, 139.7, 137.3, 135.7(minor), 135.6(major), 128.7, 128.2, 128.07, 128.05, 127.9, 127.3, 127.1, 126.9, 126.8, 126.3, 126.2, 126.1, 125.0, 124.9, 124.5, 123.5, 114.8(minor), 114.7(major), 55.47(minor), 55.46(major), 52.5(major), 52.3(minor), 50.8(minor), 50.6(major), 50.4(major), 49.5(minor), 46.5(major), 45.7(minor), 43.6(major), 39.3(minor), 38.7(major), 36.4(minor), 36.2(major), 31.4(minor) ppm.

HRMS (APCI, *m*/*z*): Calcd for $C_{27}H_{27}O_3S$ $[M+H]^+$: 431.1675, found: 431.1678.

IR is not collected due to product is a mixture.

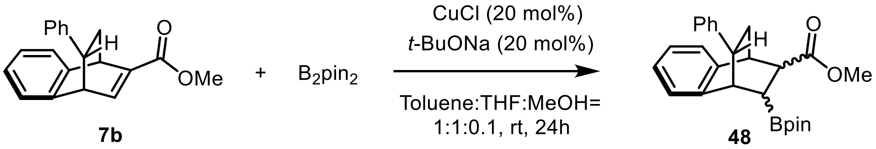

Methyl (1S,4S,10R)-10-phenyl-2-(4,4,5,5-tetramethyl-1,3,2-dioxaborolan-2-yl)-1,2,3,4-tetrahydro-1,4-ethanonaphthalene-3-carboxylate (48): An oven-dried 10 mL flask equipped with a stir bar was cooled under vacuum. CuCl (4.2 mg, 0.040 mmol, 0.20 eq.), *t*-BuONa (4.1 mg, 0.040 mmol, 0.20 eq.), bis(pinacolato)diboron (B_2pin_2, 106 mg, 0.400 mmol, 2.00 eq.) and **7b** (58 mg, 0.20 mmol, 1.0 eq.) were added into the flaskAfter evacuated/backfilled with N_2 (x 3), anhydrous toluene (1.5 mL), THF (1.5 mL) and MeOH (0.15 mL) were added successively by syringe. The reaction mixture was stirred for 24 hours. After completion, the reaction was quenched with sat. NH_4Cl (aq.) (10 mL), extracted with DCM (3 × 10 mL). The combined organic layers were washed with brine (10 mL), dried over with anhydrous Na_2SO_4, filtered and concentrated by rotary evaporation. The residue was purified by FCC (Hex: EtOAc = 30:1 to 8:1) to afford one diastereoisomer **48** (51 mg, 61% yield, dr=1:1)[34] as colorless oil.

^{1}H NMR (500 MHz, Chloroform-*d*) δ 7.19 – 7.14 (m, 2H), 7.09 – 6.96 (m, 4H), 6.81 (d, J = 7.3 Hz, 1H), 6.59 (d, J = 6.4 Hz, 2H), 3.70 (s, 3H), 3.69-3.67 (m, 1H), 3.36 (s, 1H), 2.98 (s, 1H), 2.84 (d, J = 11.7 Hz, 1H), 2.25 – 2.21 (m, 1H), 1.29 (s, 6H), 1.25 (s, 6H), 1.15 (d, J = 11.7 Hz, 1H).

^{13}C NMR (126 MHz, Chloroform-*d*) δ 176.6, 147.4, 143.2, 142.6, 128.0, 127.9, 126.4, 126.2, 125.7, 125.3, 123.6, 83.3, 52.1, 43.5, 43.1, 39.5, 38.3, 32.5, 25.2 ppm.

HRMS (ESI, *m*/*z*): Calcd for $C_{26}H_{31}O_4BNa^+$ $[M+Na]^+$: 441.2208, found: 441.2213.

IR: 3023 (s), 2945 (s), 1680 (s), 1427 (s), 1193 (s).

9. Synthesis of drug molecules

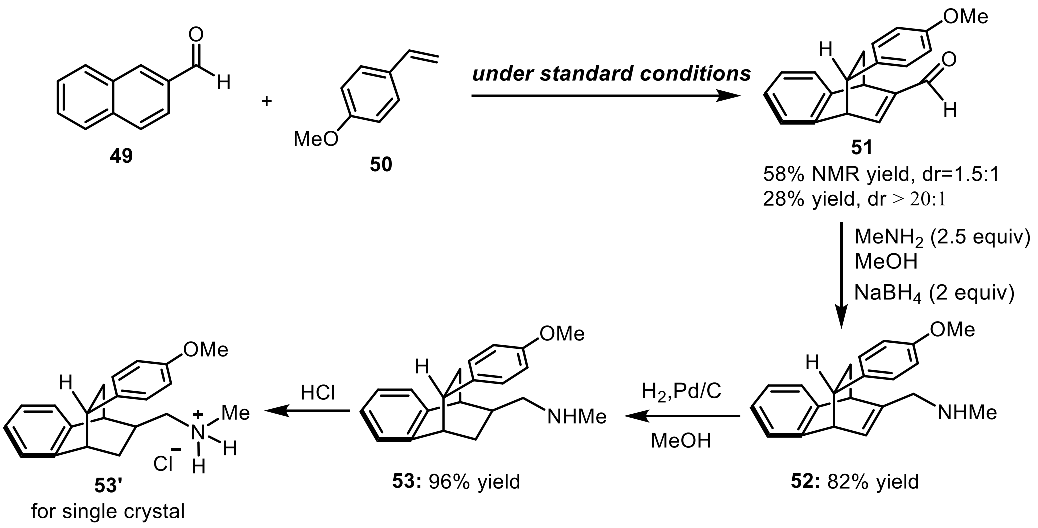

An oven-dried 20 mL flask with a stir bar and a septum was cooled under vacuum. After evacuated/backfilled with N_2 (x3), the flask was transferred into glovebox and charged with Ir(*p*-CF_3-ppy)$_3$ (10 mg, 1.0 mol%) and 2-naphthaldehyde (156 mg, 1.00 mmol, 1.00 eq.). After moved out, 1-methoxy-4-vinylbenzene (1.3 g, 10 mmol, 10 eq.) and dry DCM (10 mL) were sequentially added to the vial. Then the septum was replaced with a cap. The vial was capped and the reaction was stirred under blue LED irradiation with a cooling fan to keep room temperature. After completion, quenched with sat. NH_4Cl (aq.) (10 mL), extracted with DCM (3 × 10 mL). The combined organic layer was washed with brine (10 mL), dried over with anhydrous Na_2SO_4, filtered and concentrated by rotary evaporation. The residue was purified by FCC (Hex: EtOAc = 20:1 to 10:1) to obtain corresponding cycloadducts **51** as colorless oil (81 mg, 28% yield, dr >20:1).

^{1}H NMR (500 MHz, Chloroform-*d*) δ 9.63 (s, 1H), 7.39 (dd, *J* = 6.2, 1.8 Hz, 1H), 7.34 – 7.29 (m, 2H), 7.21 – 7.18 (m, 2H), 7.07 (d, *J* = 8.7 Hz, 2H), 6.86 (d, *J* = 8.7 Hz, 2H), 4.72 (d, *J* = 2.2 Hz, 1H), 4.28 (dd, *J* = 6.1, 2.2 Hz, 1H), 3.82 (s, 3H), 3.17 – 3.13 (m, 1H), 2.22 – 2.17 (m, 1H), 1.78 – 1.74 (m, 1H).

^{13}C NMR (126 MHz, Chloroform-*d*) δ 188.4, 158.5, 153.8, 151.0, 143.2, 142.2, 136.3, 128.7, 126.3, 125.9, 124.1, 123.2, 114.0, 55.4, 49.9, 45.0, 37.5, 34.9 ppm.

HRMS (APCI, *m*/*z*): Calcd for $C_{20}H_{17}O_2^+$ [M-H]$^+$: 289.1223, found: 289.1221.

IR: 2954 (m), 2834 (s), 1675 (s), 1510 (s), 1473 (s).

An oven-dried 10 mL flask equipped with a stir bar was cooled under vacuum. Aldehyde **51** (58 mg, 0.20 mmol, 1.0 equiv.) was added into the flask.After evacuated/backfilled with N_2 (x 3), dry MeOH (2 mL) and $MeNH_2$ (80 μL, 0.50 mmol, 2.5 eq., 40% in MeOH) were added. After 1 h of stirring at room temperature, imine formation was complete (monitored by NMR). $NaBH_4$ (15.1 mg, 0.400 mmol, 2.00 eq.) was added and the reaction mixture and the mixture was stirred under N_2 atmosphere for 2 h at room temperature. The reaction was quenched by addition of aqueous NaOH (2.0 M, xx mL), extracted with DCM (3 × 10 mL). The combined organic layers were washed with brine (10 mL), dried over with anhydrous Na_2SO_4, filtered and concentrated by rotary evaporation. The residue was purified by FCC (Hex: EtOAc =

5:1 to 1:1) to afford corresponding amine **52** as a light yellow oil (50 mg, 82% yield).
^{1}H NMR (500 MHz, Chloroform-*d*) δ 7.15 – 6.98 (m, 6H), 6.73 (d, *J* = 8.3 Hz, 2H), 6.05 (d, *J* = 6.0 Hz, 1H), 5.15 (s, 1H), 3.87 (s, 1H), 3.81 (d, *J* = 4.0 Hz, 1H), 3.68 (s, 3H), 3.33 (q, *J* = 15.0 Hz, 2H), 2.87 – 2.84 (m, 1H), 2.27 (s, 3H), 2.03 – 1.96 (m, 1H), 1.62 – 1.58 (m, 1H).
^{13}C NMR (126 MHz, Chloroform-*d*) δ 158.0, 148.3, 146.1, 143.7, 137.8, 128.9, 126.6, 125.2, 125.1, 123.1, 122.2, 113.7, 55.3, 54.3, 47.9, 44.7, 43.9, 36.3, 35.6 ppm.
HRMS (ESI, *m*/*z*): Calcd for $C_{21}H_{24}ON^+$ $[M+H]^+$: 306.1852, found: 306.1856.
IR: 2946 (s), 2833 (s), 1581 (s), 1302 (s), 1036 (s).

An oven-dried 10 mL flask equipped with a stir bar was cooled under vacuum. Amine **52** (61mg, 0.20 mmol, 1.00 eq.) and Pd/C 10% (20 mg) were added into the flask. After evacuated/backfilled with N_2 (x 3), dry MeOH (1 mL) was added. After evacuated/backfilled with H_2, the reaction mixture was stirred at room temperature for 24 h with H_2 balloon attached. The reaction mixture was filtered throught a pad of celite. The filtrate concentrated by rotary evaporation to afford the product **53** as a light yellow oil (59 mg, 96% yield, >20:1 dr).
^{1}H NMR (500 MHz, Chloroform-*d*) δ 7.24 – 7.16 (m, 6H), 6.88 (d, *J* = 8.7 Hz, 2H), 5.28 (s, 1H), 3.79 (s, 3H), 3.18 (s, 1H), 3.07 (s, 1H), 3.03 – 2.99 (m, 1H), 2.85 – 2.78 (m, 2H), 2.53 (s, 3H), 2.07 – 2.03 (m, 1H), 1.89 – 1.82 (m, 2H), 1.60 – 1.54 (m, 2H), 1.43 – 1.39 (m, 1H).
^{13}C NMR (126 MHz, Chloroform-*d*) δ 158.0, 144.3, 143.9, 135.2, 129.0, 126.5, 126.4, 123.9, 123.8, 114.0, 55.5, 53.4, 42.4, 40.7, 36.8, 35.0, 29.9, 25.0, 24.8 ppm.
HRMS (ESI, *m*/*z*): Calcd for $C_{21}H_{26}ON^+$ $[M+H]^+$: 308.2009, found: 308.2011.
IR: 2928 (s), 2789 (s), 1609 (s), 1460 (s), 1179 (s).

The product **53** was dissolved in Et_2O. An excess of a solution of HCl was added to form a precipitate, and then filtered, filtrate concentrated by rotary evaporation to afford the product **53’** as a white solid.
^{1}H NMR (500 MHz, Chloroform-*d*) δ 9.41 (s, 2H), 7.19 – 7.06 (m, 6H), 6.82 (d, *J* = 8.6 Hz, 2H), 3.72 (s, 3H), 3.29 (s, 1H), 3.13 – 3.09 (m, 1H), 3.04 (s, 1H), 2.90 – 2.85 (m, 1H), 2.75 – 2.71 (m, 1H), 2.54 (s, 3H), 1.99 – 1.94 (m, 2H), 1.85 – 1.79 (m, 1H), 1.61 – 1.55 (m, 1H), 1.39 – 1.34 (m, 1H).
^{13}C NMR (126 MHz, Chloroform-*d*) δ 158.0, 143.8, 143.1, 134.8, 128.9, 126.60, 126.56, 124.1, 123.8, 114.1, 55.4, 52.0, 42.1, 40.2, 36.3, 35.6, 33.5, 25.0, 24.7 ppm.
HRMS (ESI, *m*/*z*): Calcd for $C_{21}H_{26}ON^+$ $[M-Cl]^+$: 308.2009, found: 308.2011.
IR: 2923 (s), 2852 (s), 1740 (s), 1512 (s), 1248 (s).

10. X-ray structure

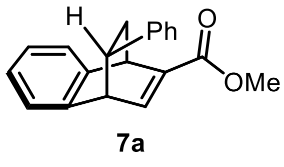

7a

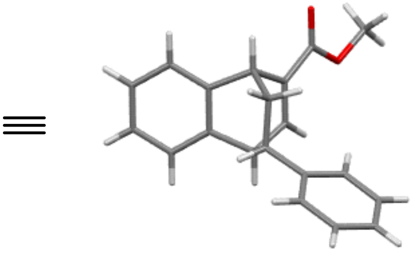

Single crystal was obtained by slow evaporation from DCM solution. A colorless crystal (approximate dimensions 0.257 × 0.193 × 0.084 mm^3) was placed onto the tip of a MiTeGen loop and mounted on a Bruker Venture D8 diffractometer equipped with a PhotonIII detector at 153(2) K.

Data collection

The data collection was carried out using Mo Kα radiation (graphite monochromator) with a frame time of 1 and 3 seconds and a detector distance of 4.00 cm. A collection strategy was calculated and complete data to a resolution of 0.80 Å with a redundancy of 18 were collected. Ten major sections of frames were collected with 1° ω and Φ scans. A total of 1470 frames were collected. The total exposure time was 0.82 hours. The frames were integrated with the Bruker SAINT software package[35] using a narrow-frame algorithm. The integration of the data using a monoclinic unit cell yielded a total of 57415 reflections to a maximum θ angle of 26.39° (0.80 Å resolution), of which 3047 were independent (average redundancy 18.843, completeness = 99.9%, Rint = 9.52%, Rsig = 2.57%) and 2445 (80.24%) were greater than $2\sigma(F^2)$. The final cell constants of a = 9.6874(7) Å, b = 14.9785(12) Å, c = 11.1842(9) Å, β = 113.606(2)°, volume = 1487.1(2) $Å^3$, are based upon the refinement of the XYZ-centroids of 9942 reflections above 20 σ(I) with 4.716° < 2θ < 52.64°. Data were corrected for absorption effects using the Multi-Scan method (SADABS[36]). The ratio of minimum to maximum apparent transmission was 0.891. The calculated minimum and maximum transmission coefficients (based on crystal size) are 0.9790 and 0.9930. Please refer to Table 1 for additional crystal and refinement information.

Structure solution and refinement

The space group $P2_1/n$ was determined based on intensity statistics and systematic absences. The structure was solved and refined using the SHELX suite of programs.[37,38] An intrinsic-methods solution was calculated, which provided most non-hydrogen atoms from the E-map. Full-matrix least squares / difference Fourier cycles were performed, which located the remaining non-hydrogen atoms. All non-hydrogen atoms were refined with anisotropic displacement parameters. The hydrogen atoms were placed in ideal positions and refined as riding atoms with relative isotropic displacement parameters. The final anisotropic full-matrix least-squares refinement on F^2 with 200 variables converged at R1 = 5.02%, for the observed data and wR2 = 13.22% for all data. The goodness-of-fit was 1.095. The largest peak in the final difference electron density synthesis was 0.276 e-/$Å^3$ and the largest hole was -0.235 e-/$Å^3$ with an RMS deviation of 0.048 e-/Å3. On the basis of the final model, the calculated density was 1.297 g/cm^3 and F(000), 616 e^-.

Crystal data and structure refinement

Empirical formula	$C_{20}H_{18}O_2$	
Formula weight	290.34	
Crystal color, shape, size	colourless block, 0.257 × 0.193 × 0.084 mm3	
Temperature	153(2) K	
Wavelength	0.71073 Å	
Crystal system, space group	Monoclinic, $P2_1/n$	
Unit cell dimensions	a = 9.6874(7) Å	α = 90°.

	b = 14.9785(12) Å β = 113.606(2)°.
	c = 11.1842(9) Å γ = 90°.
Volume	1487.1(2) $Å^3$
Z	4
Density (calculated)	1.297 Mg/m^3
Absorption coefficient	0.082 mm^{-1}
F(000)	616
**Data collection**	
Diffractometer	Venture D8, Bruker
Source	IµS 3.0, Incoatec
Detector	Photon III
Theta range for data collection	2.358 to 26.388°.
Index ranges	-11<=h<=12, -18<=k<=18, -13<=l<=13
Reflections collected	57415
Independent reflections	3047 [R_{int} = 0.0952]
Observed Reflections	2445
Completeness to theta = 25.242°	100.0 %
**Solution and Refinement**	
Absorption correction	Semi-empirical from equivalents
Max. and min. transmission	0.7454 and 0.6643
Solution	Intrinsic methods
Refinement method	Full-matrix least-squares on F^2
Weighting scheme	$w = [\sigma^2Fo^2 + AP^2 + BP]^{-1}$, with
	$P = (Fo^2 + 2 Fc^2)/3$, A = 0.0592, B = 0.7699
Data / restraints / parameters	3047 / 0 / 200
Goodness-of-fit on F^2	1.095
Final R indices [I>2σ(I)]	R1 = 0.0502, wR2 = 0.1244
R indices (all data)	R1 = 0.0619, wR2 = 0.1322
Largest diff. peak and hole	0.276 and -0.235 e.$Å^{-3}$

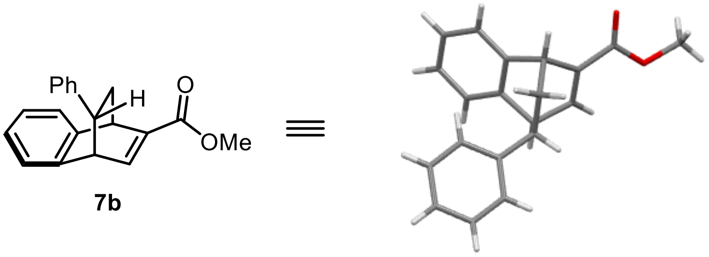

Single crystal was obtained by slow evaporation from DCM solution. A colorless crystal (approximate dimensions 0.296 × 0.266 × 0.228 mm^3) was placed onto the tip of a MiTeGen loop and mounted on a Bruker Venture D8 diffractometer equipped with a PhotonIII detector at 153(2) K.

Data collection

The data collection was carried out using Mo Kα radiation (graphite monochromator) with a frame time of 1 seconds and a detector distance of 4.00 cm. A collection strategy was calculated and complete data to a resolution of 0.75 Å with a

redundancy of 11 were collected. Six major sections of frames were collected with 1° ω and Φ scans. A total of 982 frames were collected. The total exposure time was 0.27 hours. The frames were integrated with the Bruker SAINT software package[35] using a narrow-frame algorithm. The integration of the data using a monoclinic unit cell yielded a total of 40558 reflections to a maximum θ angle of 27.53° (0.77 Å resolution), of which 3474 were independent (average redundancy 11.675, completeness = 99.9%, Rint = 6.51%, Rsig = 2.80%) and 2685 (77.29%) were greater than 2σ(F2). The final cell constants of a = 17.4160(7) Å, b = 7.3263(4) Å, c = 11.8242(6) Å, β = 93.273(2)°, volume = 1506.25(13) Å^3, are based upon the refinement of the XYZ-centroids of 9228 reflections above 20 σ(I) with 6.028° < 2θ < 54.61°. Data were corrected for absorption effects using the Multi-Scan method (SADABS[36]). The ratio of minimum to maximum apparent transmission was 0.956. The calculated minimum and maximum transmission coefficients (based on crystal size) are 0.9760 and 0.9820. Please refer to Table 1 for additional crystal and refinement information.

Structure solution and refinement

The space group $P2_1/c$ was determined based on intensity statistics and systematic absences. The structure was solved and refined using the SHELX suite of programs.[37,38] An intrinsic-methods solution was calculated, which provided most non-hydrogen atoms from the E-map. Full-matrix least squares / difference Fourier cycles were performed, which located the remaining non-hydrogen atoms. All non-hydrogen atoms were refined with anisotropic displacement parameters. The hydrogen atoms were placed in ideal positions and refined as riding atoms with relative isotropic displacement parameters. The final anisotropic full-matrix least-squares refinement on F2 with 200 variables converged at R1 = 4.37%, for the observed data and wR2 = 11.58% for all data. The goodness-of-fit was 1.020. The largest peak in the final difference electron density synthesis was 0.216 e^-/Å^3 and the largest hole was -0.212 e^-/Å^3 with an RMS deviation of 0.048 e^-/Å^3. On the basis of the final model, the calculated density was 1.280 g/cm^3 and F(000), 616 e^-.

Crystal data and structure refinement

Empirical formula	$C_{20}H_{18}O_2$	
Formula weight	290.34	
Crystal color, shape, size	colorless block, 0.296 × 0.266 × 0.228 mm3	
Temperature	153(2) K	
Wavelength	0.71073 Å	
Crystal system, space group	Monoclinic, $P2_1/c$	
Unit cell dimensions	a = 17.4160(7) Å	α = 90°.
	b = 7.3263(4) Å	β = 93.273(2)°.
	c = 11.8242(6) Å	γ = 90°.
Volume	1506.25(13) Å^3	
Z	4	
Density (calculated)	1.280 Mg/m^3	
Absorption coefficient	0.081 mm^{-1}	
F(000)	616	

Data collection

Diffractometer	Venture D8, Bruker
Source	IμS 3.0, Incoatec
Detector	Photon III
Theta range for data collection	2.343 to 27.529°.
Index ranges	-22<=h<=22, -9<=k<=9, -15<=l<=15
Reflections collected	40558
Independent reflections	3474 [R_{int} = 0.0651]
Observed Reflections	2685
Completeness to theta = 25.242°	100.0 %
**Solution and Refinement**	
Absorption correction	Semi-empirical from equivalents
Max. and min. transmission	0.7456 and 0.7128
Solution	Intrinsic methods
Refinement method	Full-matrix least-squares on F^2
Weighting scheme	$w = [\sigma^2 Fo^2 + AP^2 + BP]^{-1}$, with $P = (Fo^2 + 2\ Fc^2)/3$, A = 0.0524, B = 0.5674
Data / restraints / parameters	3474 / 0 / 200
Goodness-of-fit on F^2	1.020
Final R indices [I>2σ(I)]	R1 = 0.0437, wR2 = 0.1043
R indices (all data)	R1 = 0.0629, wR2 = 0.1158
Largest diff. peak and hole	0.216 and -0.212 e.$Å^{-3}$

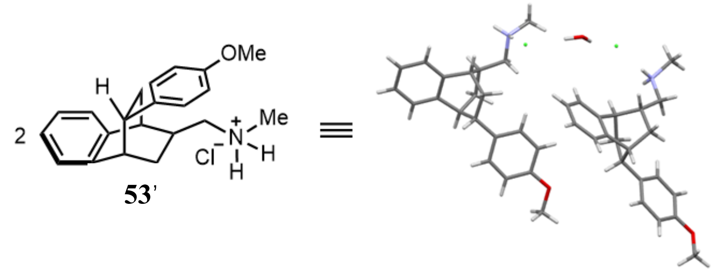

53'

Single crystal was obtained by slow evaporation from DCM/Hex solution. A colorless crystal (approximate dimensions 0.295 × 0.209 × 0.122 mm^3) was placed onto the tip of a MiTeGen loop and mounted on a Bruker Venture D8 diffractometer equipped with a PhotonIII detector at 153(2) K.

Data collection

The data collection was carried out using Mo Kα radiation (graphite monochromator) with a frame time of 2 and 3 seconds and a detector distance of 4.00 cm. A collection strategy was calculated and complete data to a resolution of 0.77 Å with a redundancy of 11 were collected. Six major sections of frames were collected with 1° ω and φ scans. A total of 1164 frames were collected. The total exposure time was 0.65 hours. The frames were integrated with the Bruker SAINT software package[35] using a narrow-frame algorithm. The integration of the data using a monoclinic unit cell yielded a total of 75469 reflections to a maximum θ angle of 27.53° (0.77 Å resolution), of which 8672 were independent (average redundancy 8.703, completeness = 99.8%, Rint = 9.57%, Rsig = 4.19%) and 7487 (86.34%) were greater than $2\sigma(F^2)$. The final cell constants of a = 16.0511(5) Å, b = 7.7600(3) Å, c = 17.0992(6) Å, β =

117.5120(10)°, volume = 1888.96(12) $Å^3$, are based upon the refinement of the XYZ-centroids of 9945 reflections above 20 σ(I) with 5.075° < 2θ < 54.95°. Data were corrected for absorption effects using the Multi-Scan method (SADABS[36]). The ratio of minimum to maximum apparent transmission was 0.849. The calculated minimum and maximum transmission coefficients (based on crystal size) are 0.9400 and 0.9750. Please refer to Table 1 for additional crystal and refinement information.

Structure solution and refinement

The space group $P2_1$ was determined based on intensity statistics and systematic absences. The structure was solved and refined using the SHELX suite of programs.[37,38] An intrinsic-methods solution was calculated, which provided most non-hydrogen atoms from the E-map. Full-matrix least squares / difference Fourier cycles were performed, which located the remaining non-hydrogen atoms. All non-hydrogen atoms were refined with anisotropic displacement parameters. The hydrogen atoms were placed in ideal positions and refined as riding atoms with relative isotropic displacement parameters. The structure is twinned with a domain ratio of 67:33. The final anisotropic fullmatrix least-squares refinement on F^2 with 460 variables converged at R1 = 4.13%, for the observed data and wR2 = 11.35% for all data. The goodness-of-fit was 1.031. The largest peak in the final difference electron density synthesis was 0.363 e-/$Å^3$ and the largest hole was -0.256 e-/$Å^3$ with an RMS deviation of 0.050 e-/$Å^3$. On the basis of the final model, the calculated density was 1.241 g/cm^3 and F(000), 756 e-.

Crystal data and structure refinement

Empirical formula	$C_{42} H_{54} Cl_2 N_2 O_3$
Formula weight	705.77
Crystal color, shape, size	colorless block, 0.295 × 0.209 × 0.122 mm^3
Temperature	153(2) K
Wavelength	0.71073 Å
Crystal system, space group	Monoclinic, $P2_1$
Unit cell dimensions	a = 16.0511(5) Å α = 90°.
	b = 7.7600(3) Å β = 117.5120(10)°.
	c = 17.0992(6) Å γ = 90°.
Volume	1888.96(12) Å3
Z	2
Density (calculated)	1.241 Mg/m^3
Absorption coefficient	0.213 mm^{-1}
F(000)	756

Data collection

Diffractometer	Venture D8, Bruker
Source	Iμ3.0, Incoatec
Detector	Photon III
Theta range for data collection	2.372 to 27.530°.
Index ranges	-20<=h<=20, -10<=k<=10, -22<=l<=22
Reflections collected	75469
Independent reflections	8672 [R_{int} = 0.0957]

Observed Reflections 7487

Completeness to theta = 25.242° 99.9 %

Solution and Refinement

Absorption correction Semi-empirical from equivalents

Max. and min. transmission 0.7456 and 0.6327

Solution Intrinsic methods

Refinement method Full-matrix least-squares on F^2

Weighting scheme $w = [\sigma^2 Fo^2 + AP^2]^{-1}$, with $P = (Fo^2 + 2 Fc^2)/3$, A = 0.075400

Data / restraints / parameters 8672 / 1 / 461

Goodness-of-fit on F^2 1.029

Final R indices [I>2σ(I)] R1 = 0.0412, wR2 = 0.1090

R indices (all data) R1 = 0.0497, wR2 = 0.1130

Major twin domain for inversion twin 0.67(6)

Largest diff. peak and hole 0.361 and -0.254 e.Å^{-3}

11. NMR Spectra

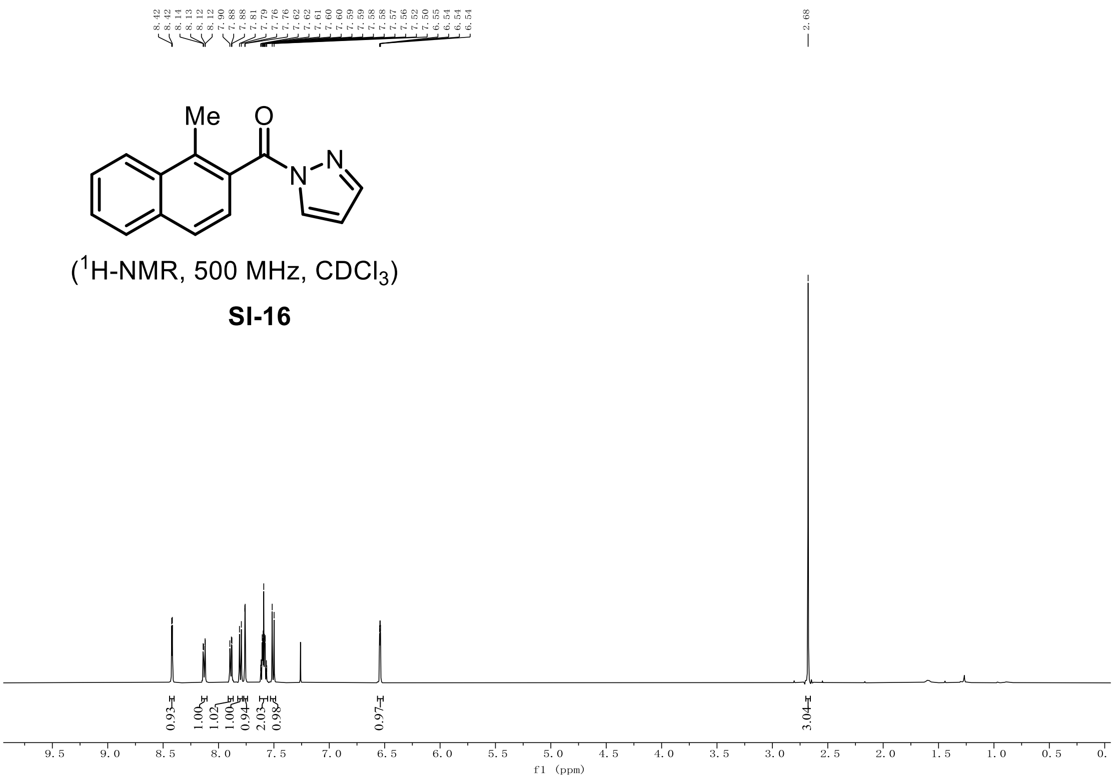

^{1}H-NMR Spectra of compound **SI-16.**

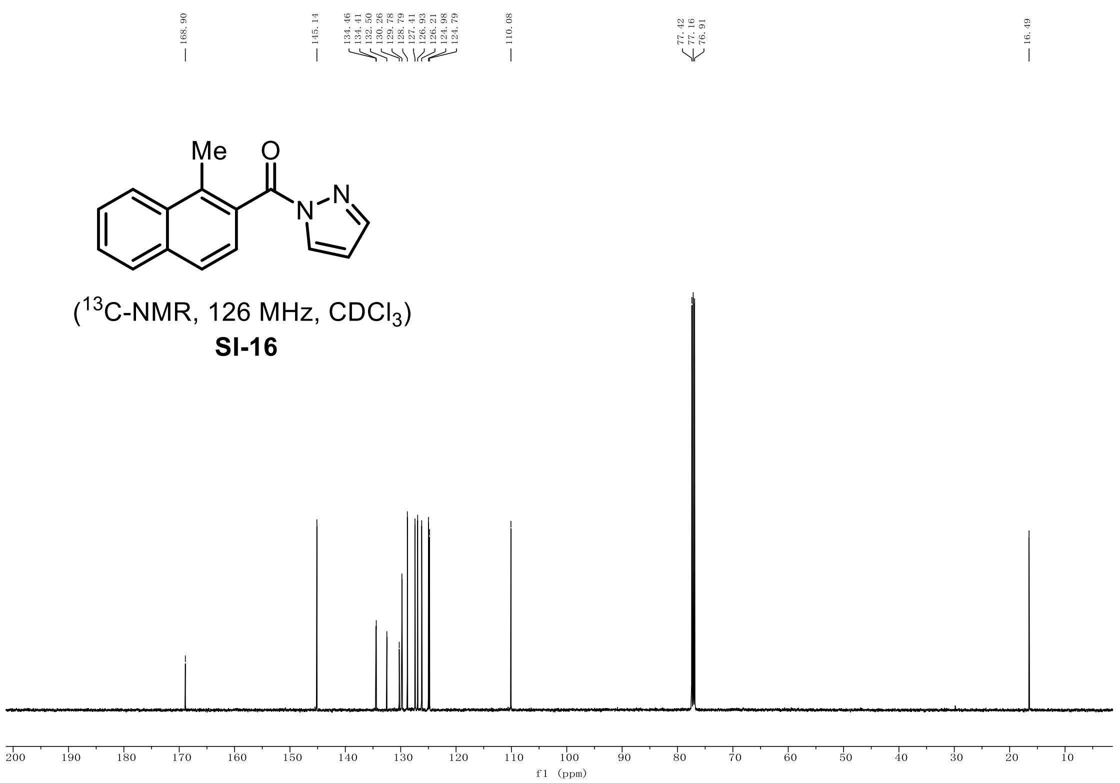

^{13}C-NMR Spectra of compound **SI-16.**

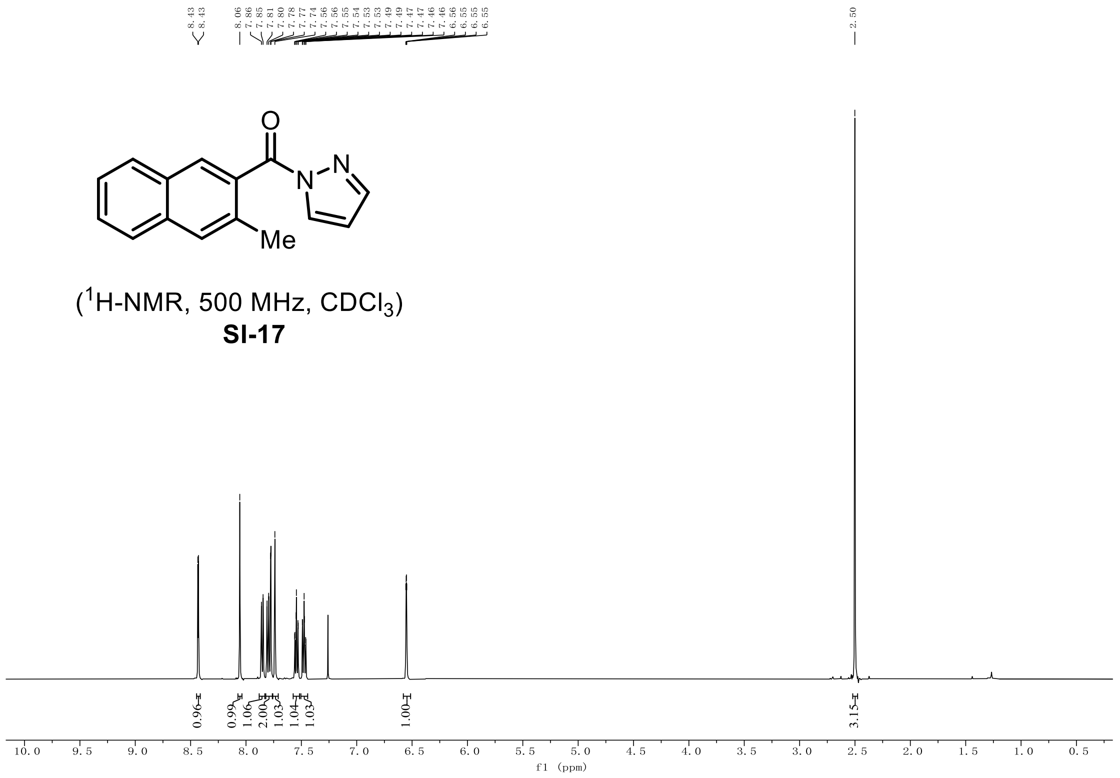

^{1}H-NMR Spectra of compound **SI-17.**

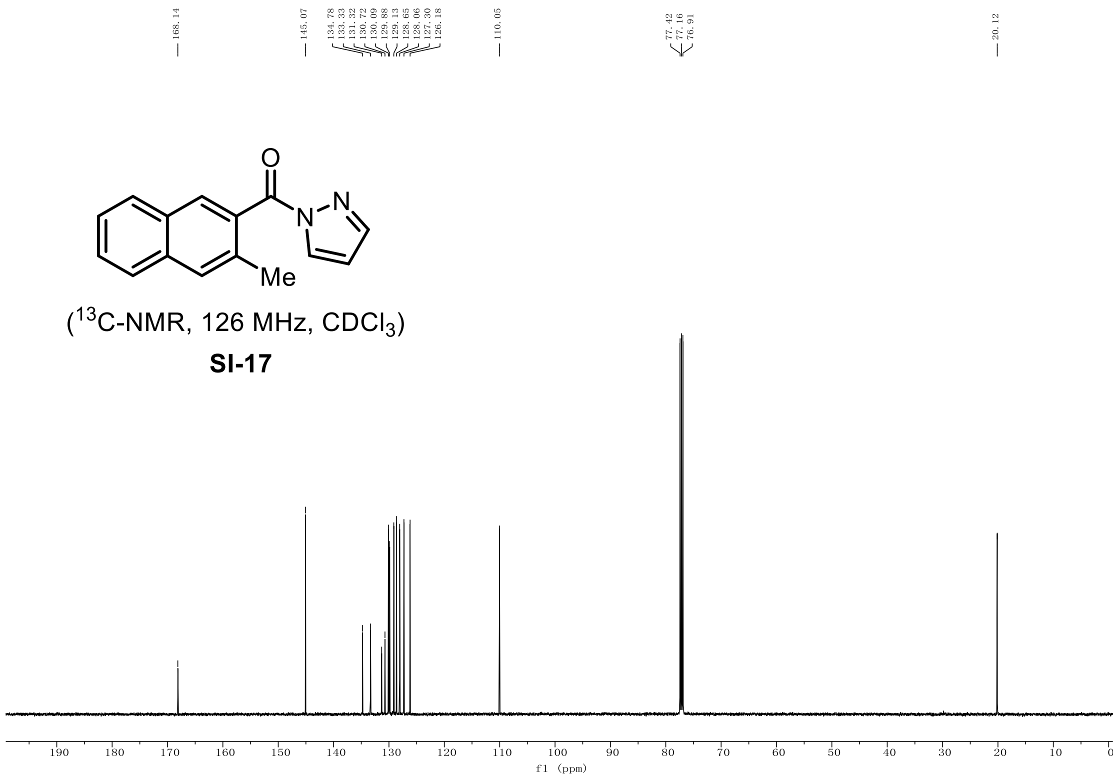

^{13}C-NMR Spectra of compound **SI-17.**

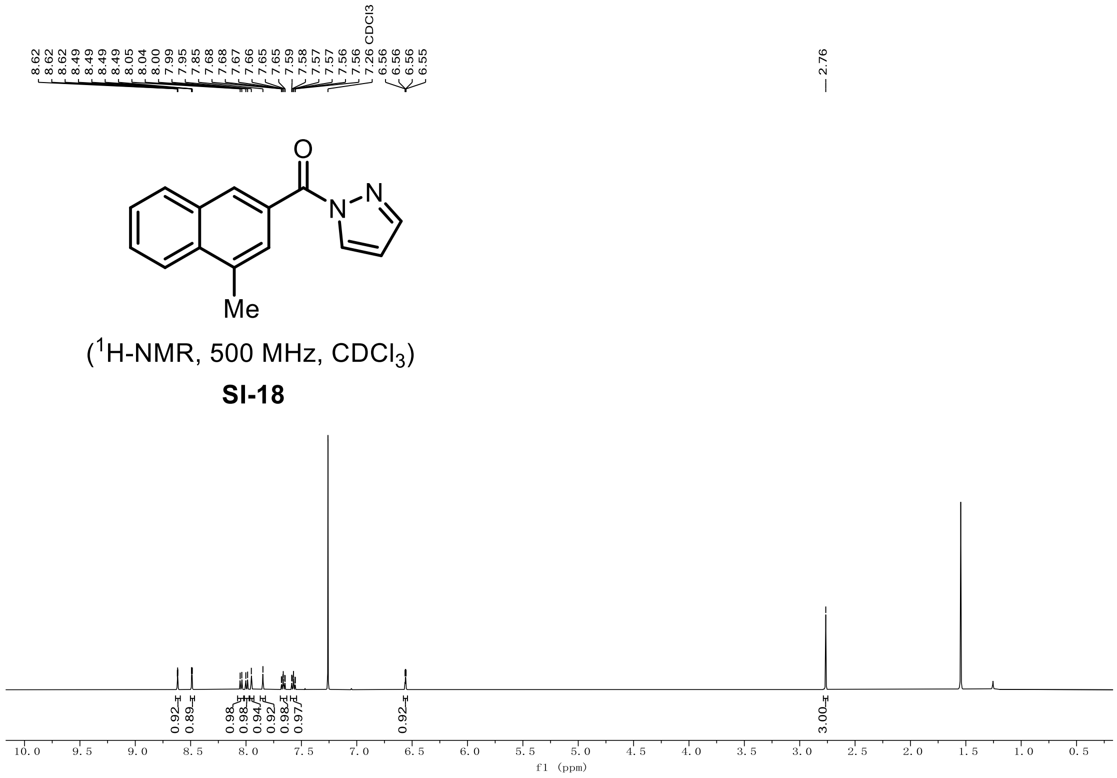

^{1}H-NMR Spectra of compound **SI-18.**

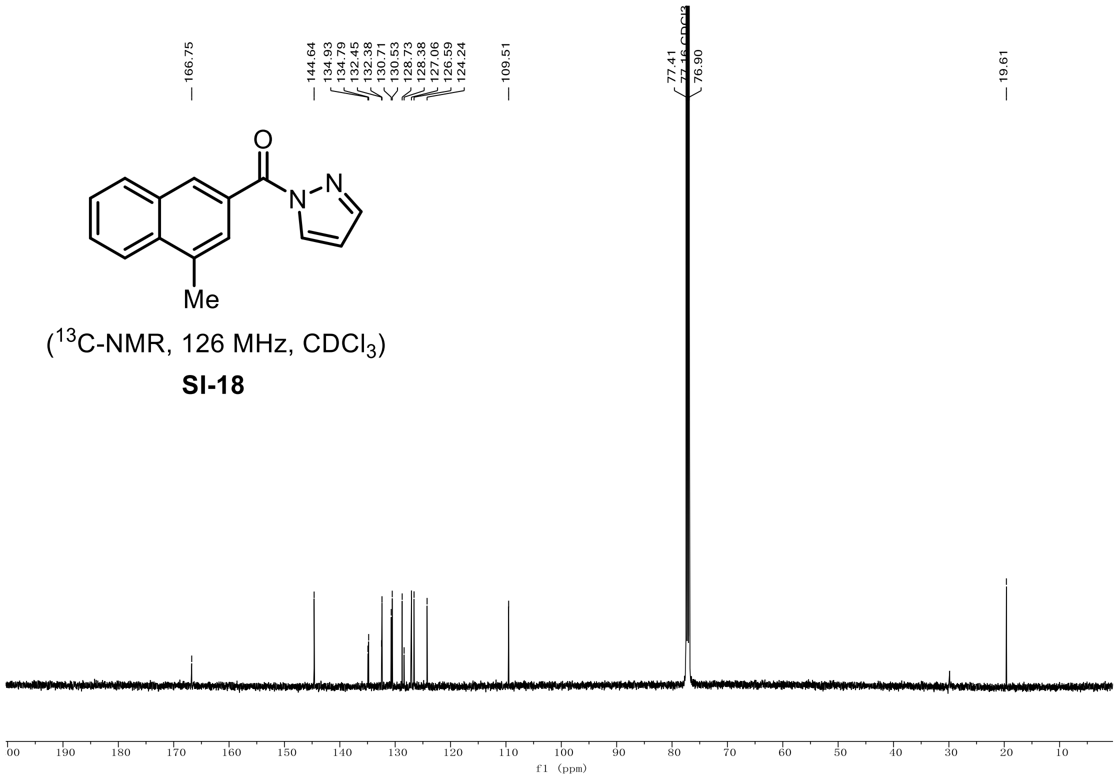

^{13}C-NMR Spectra of compound **SI-18.**

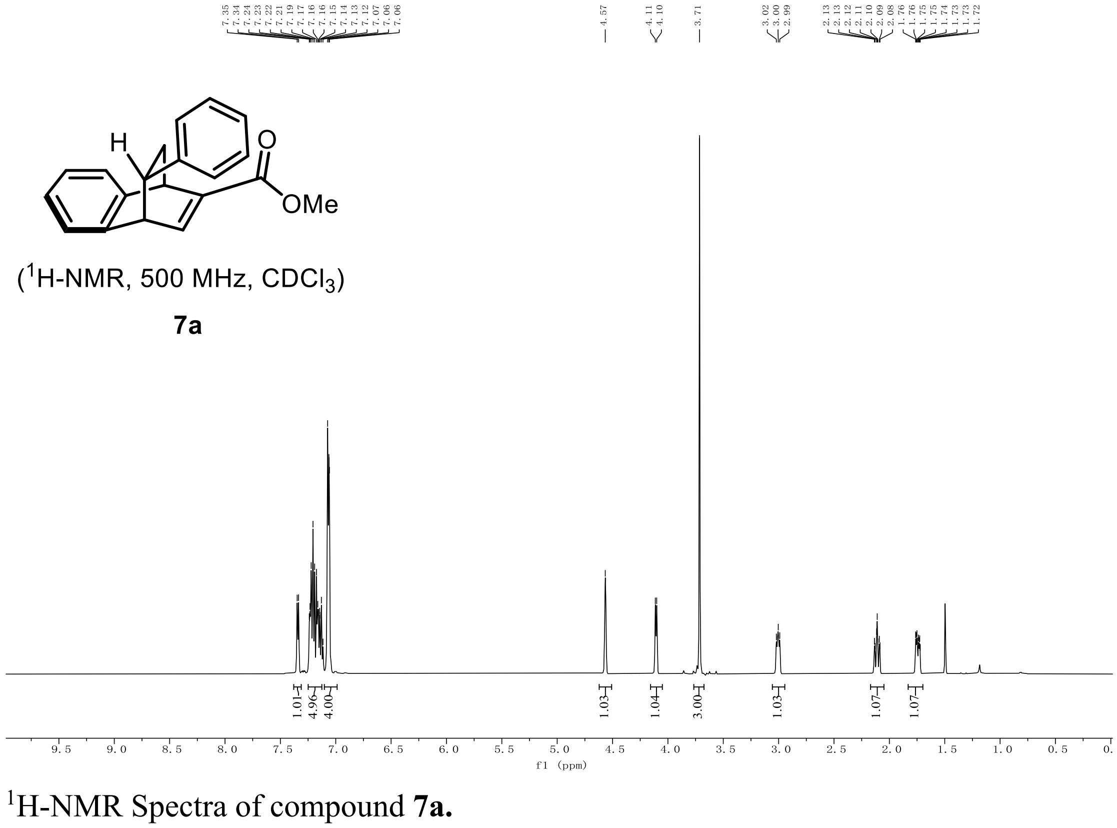

^{1}H-NMR Spectra of compound **7a.**

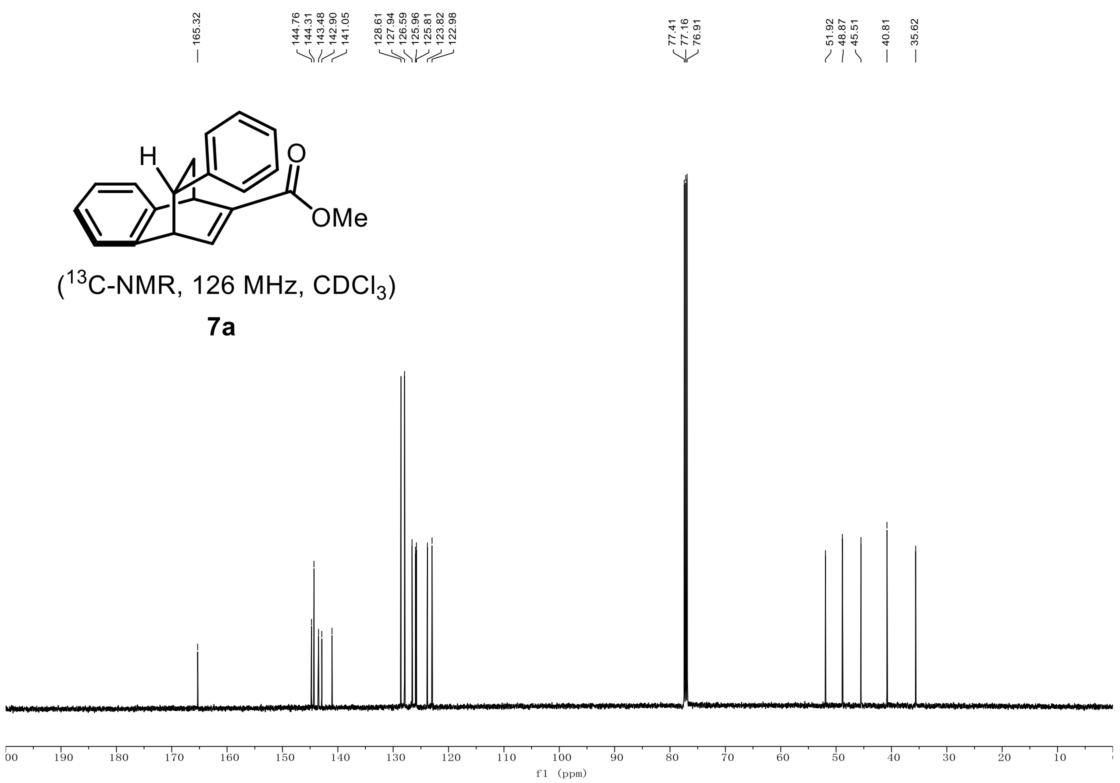

^{13}C-NMR Spectra of compound **7a.**

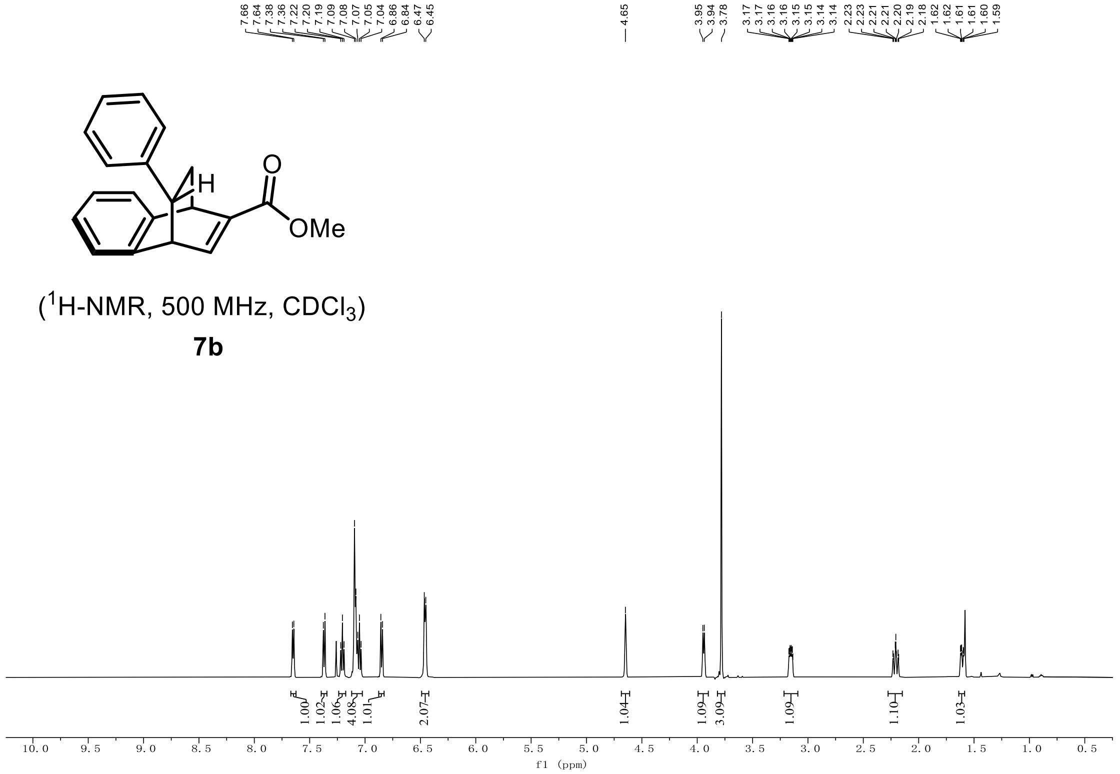

^{1}H-NMR Spectra of compound **7b.**

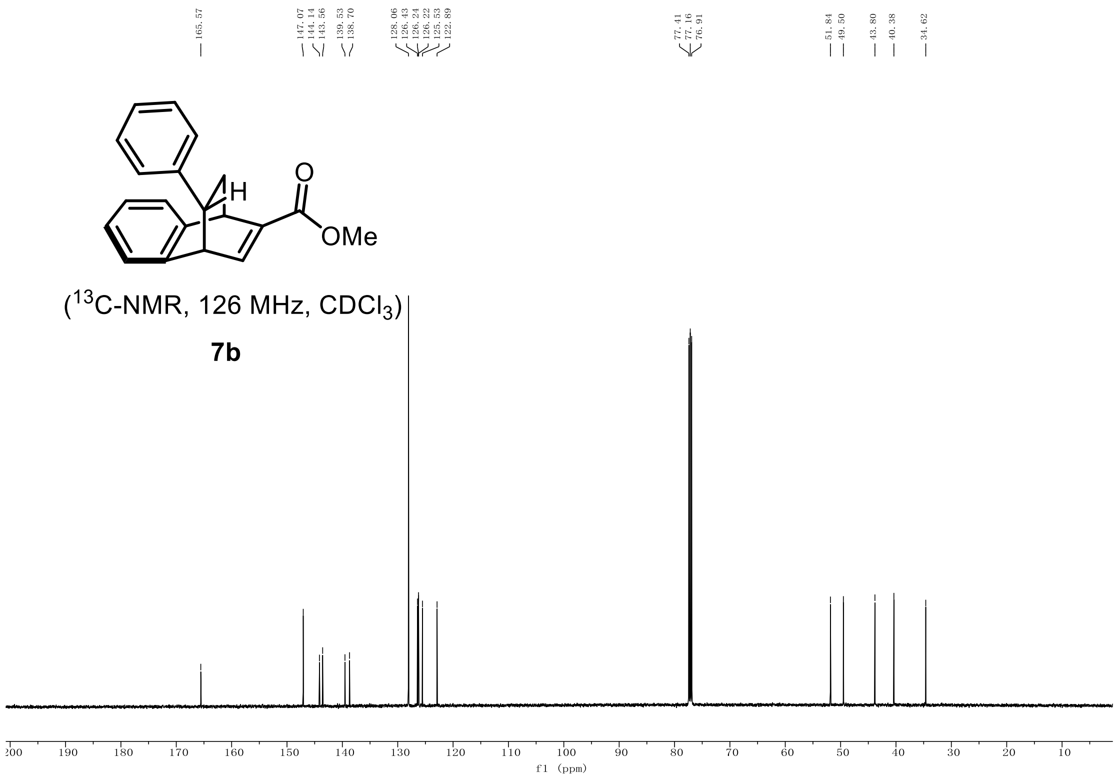

^{13}C-NMR Spectra of compound **7b.**

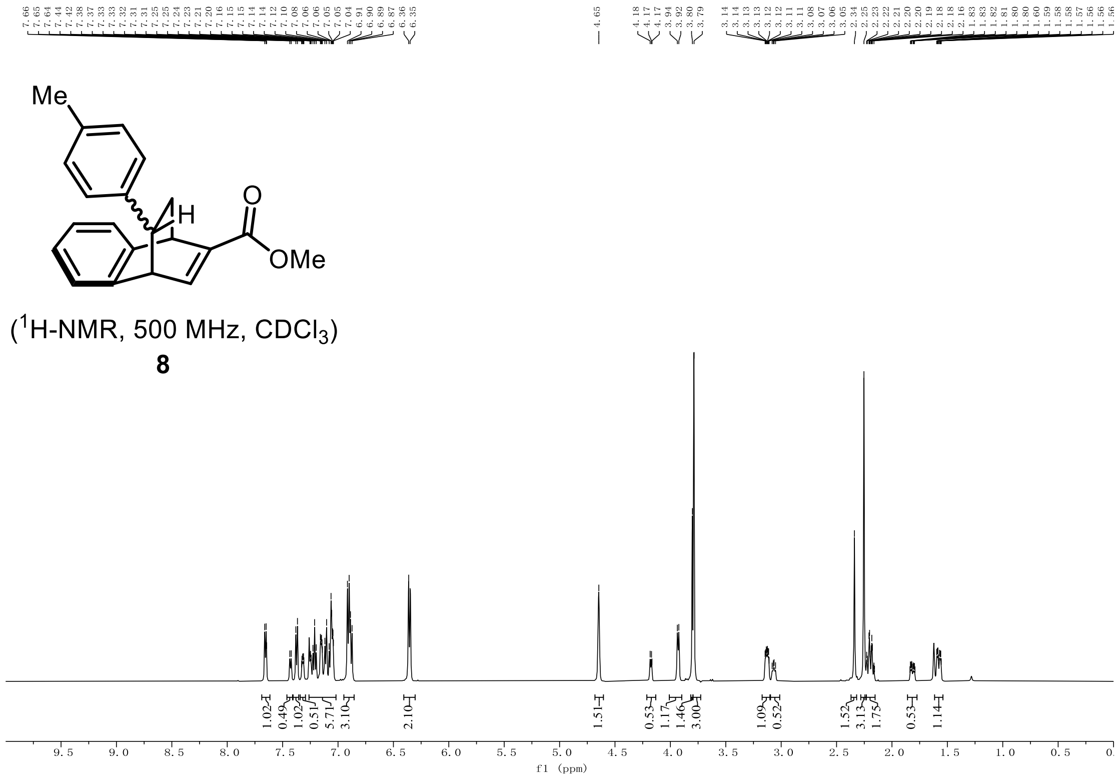

^{1}H-NMR Spectra of compound **8.**

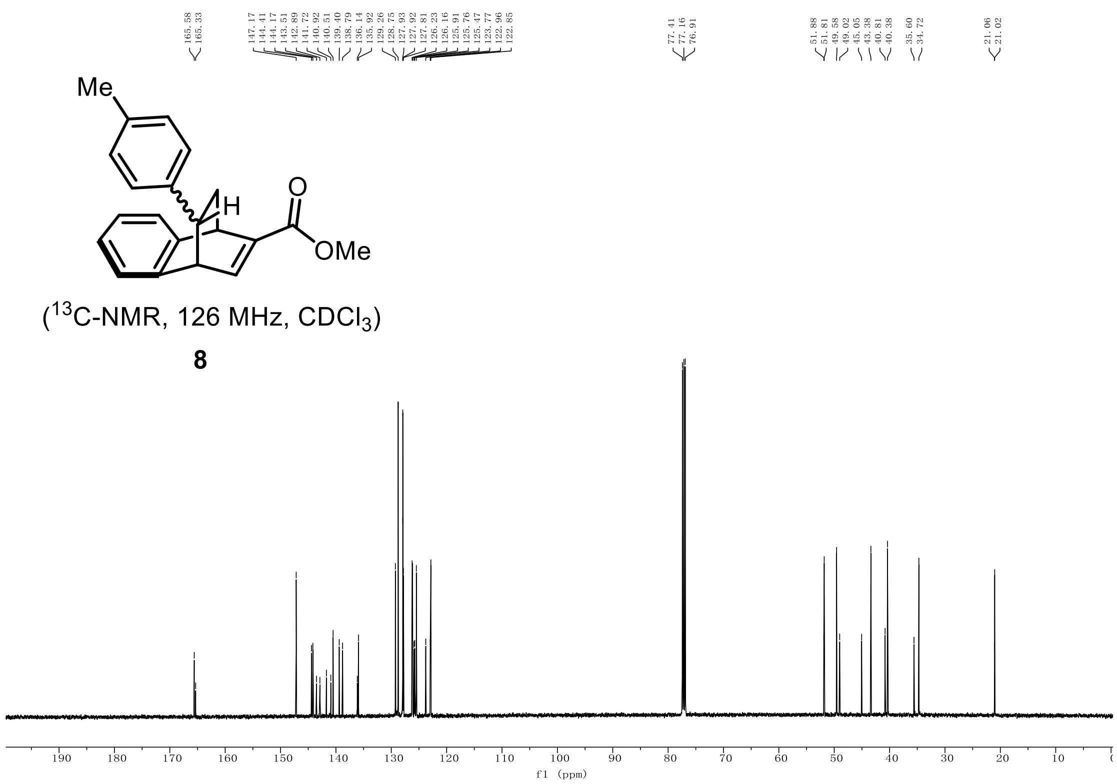

^{13}C-NMR Spectra of compound **8.**

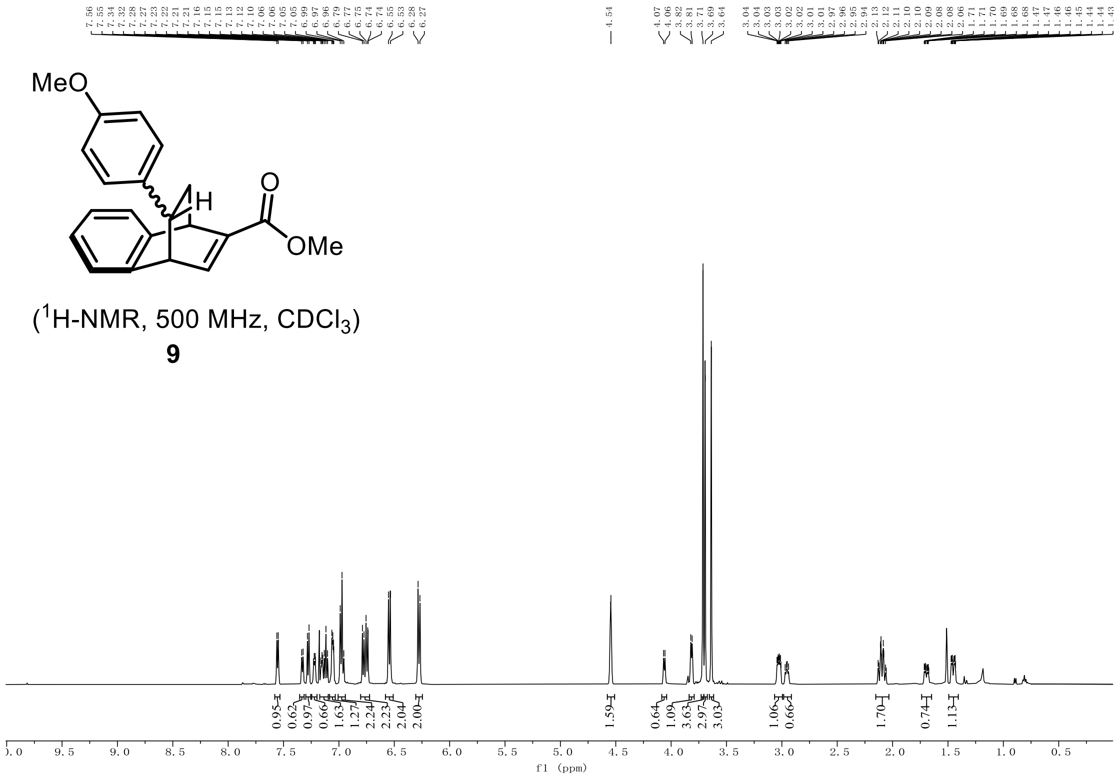

^{1}H-NMR Spectra of compound **9.**

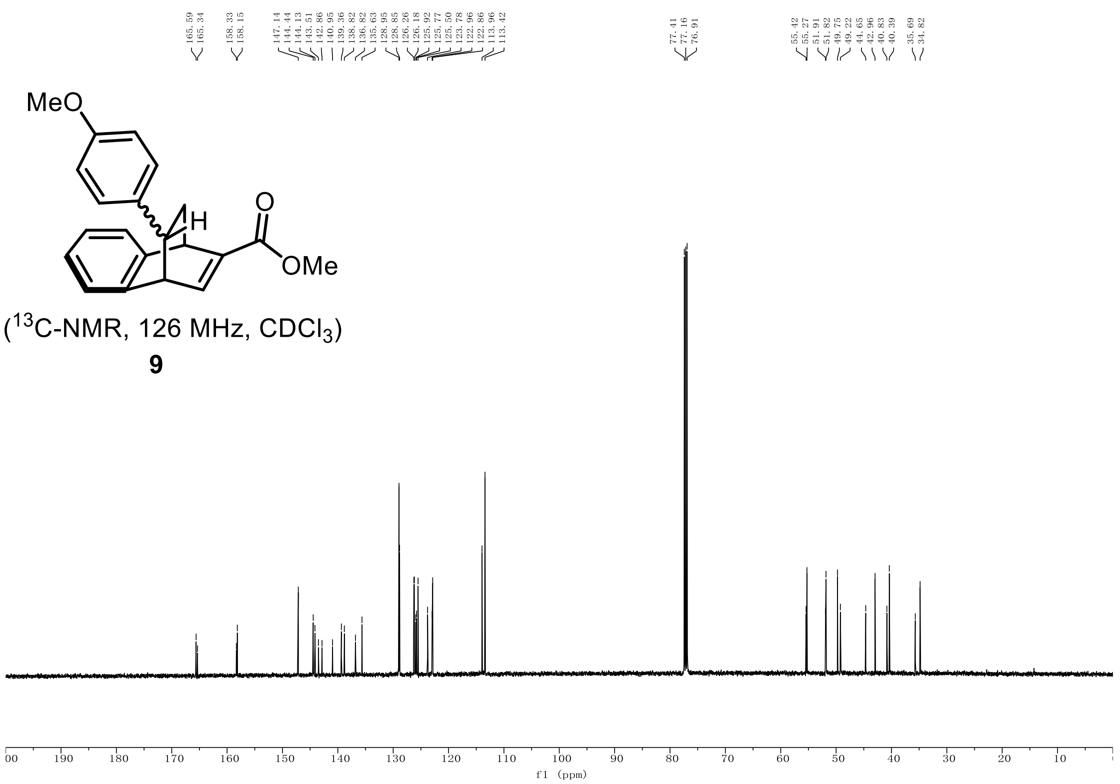

^{13}C-NMR Spectra of compound **9.**

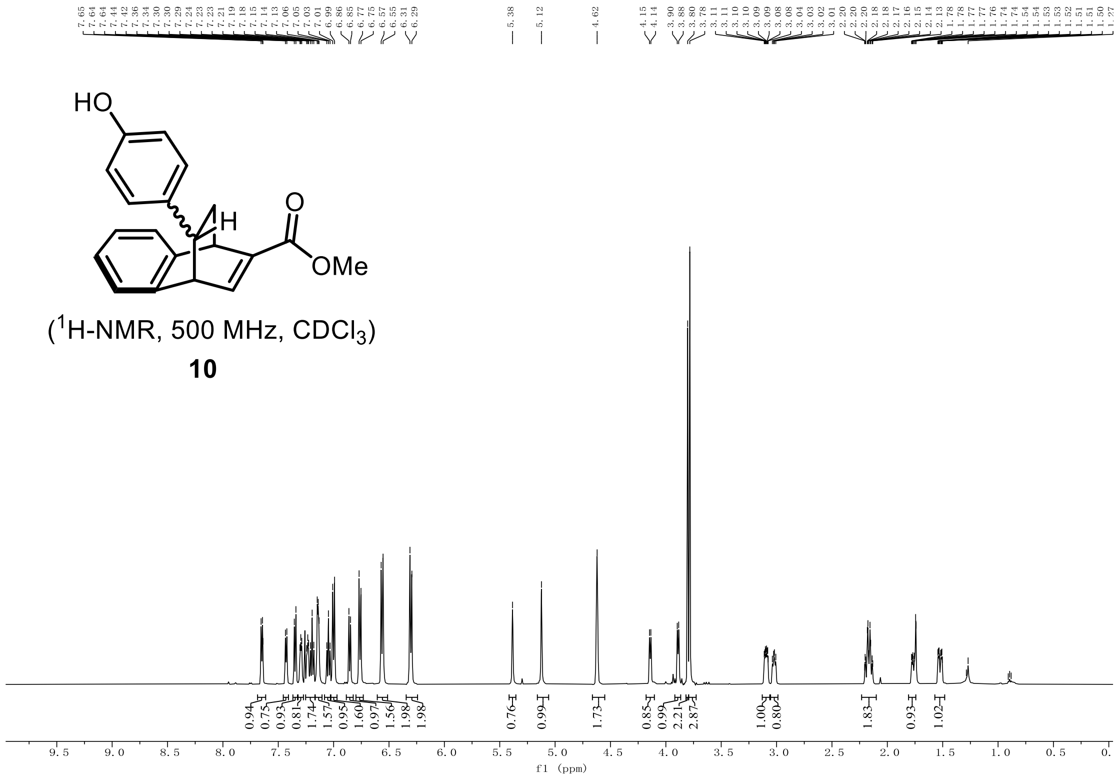

^{1}H-NMR Spectra of compound **10.**

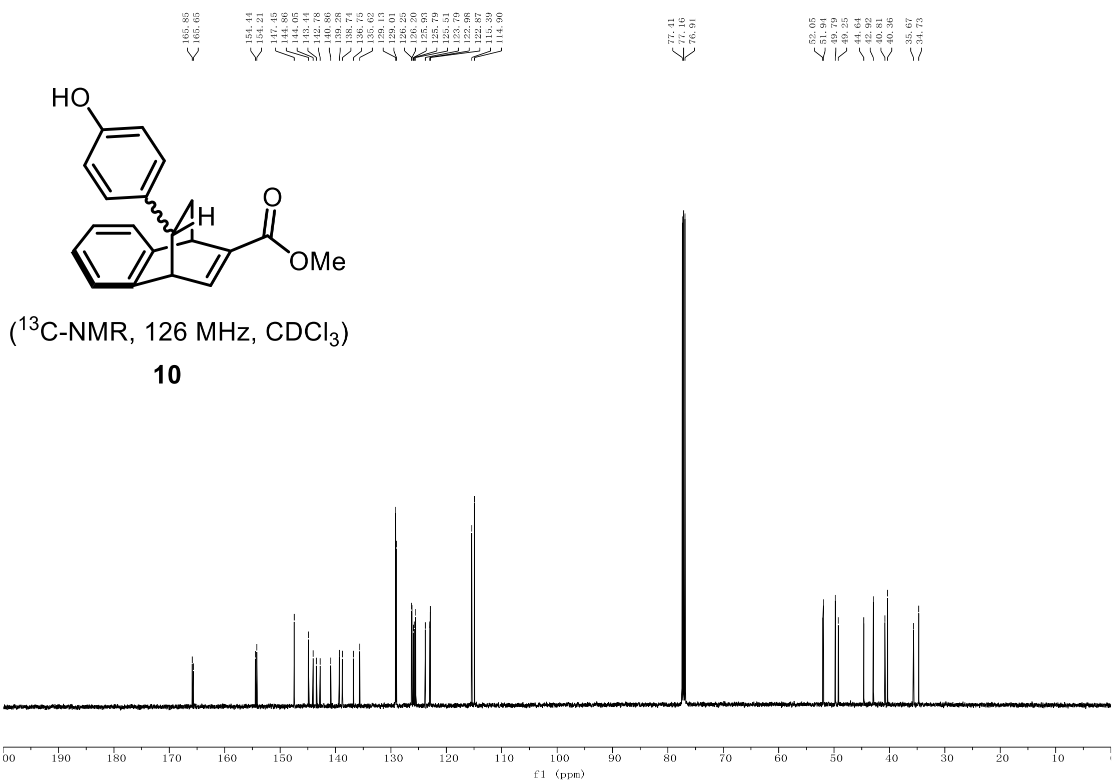

^{13}C-NMR Spectra of compound **10.**

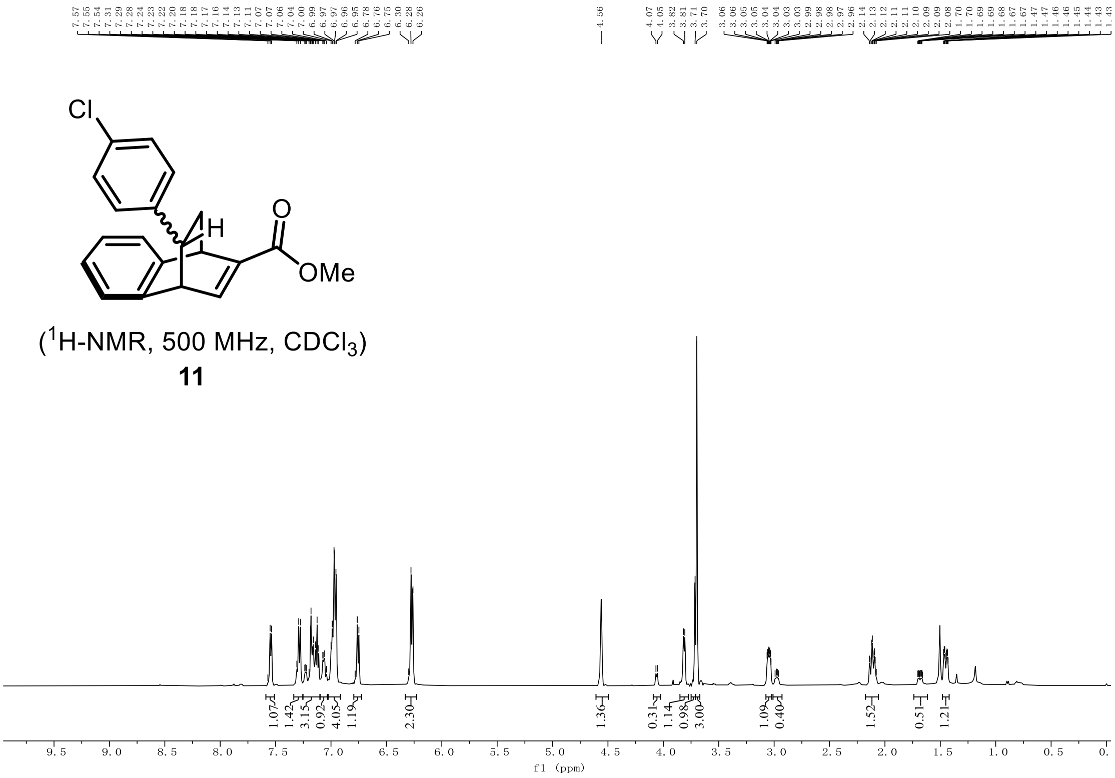

^{1}H-NMR Spectra of compound **11.**

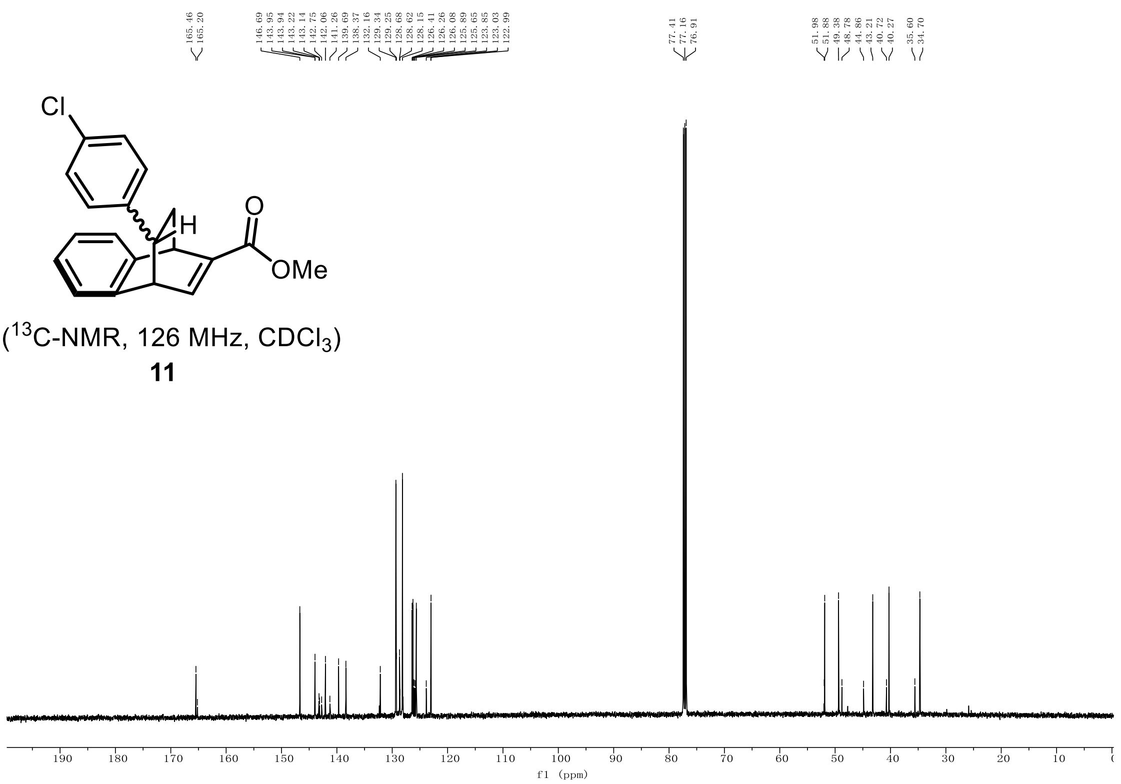

^{13}C-NMR Spectra of compound **11.**

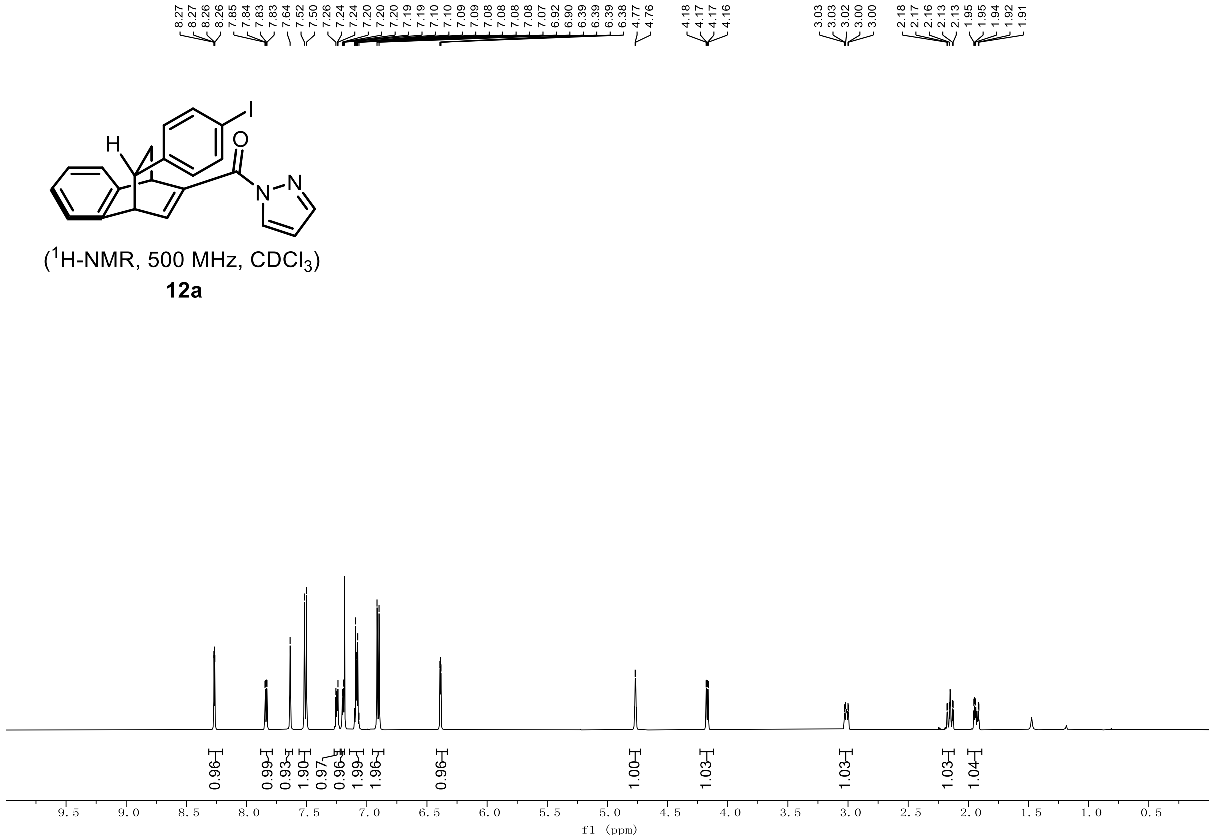

^{1}H-NMR Spectra of compound **12a.**

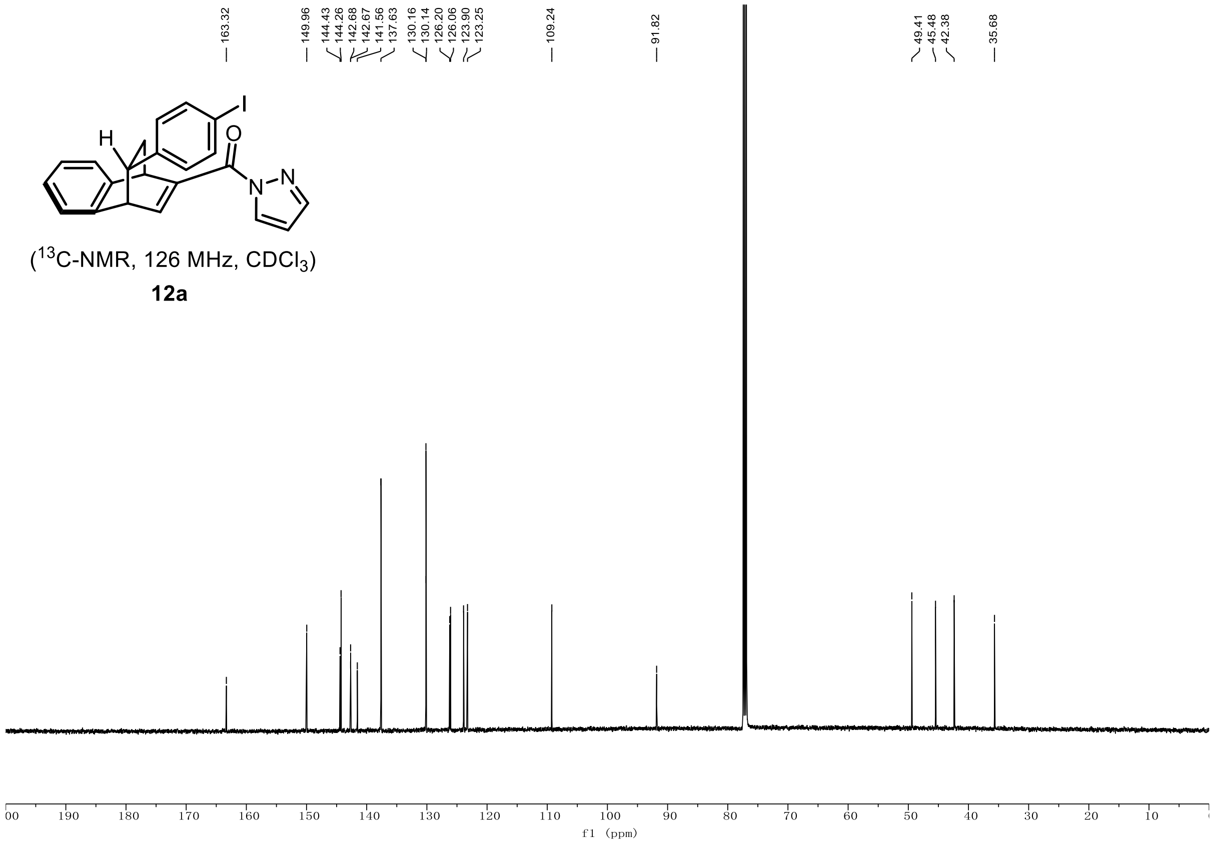

^{13}C-NMR Spectra of compound **12a.**

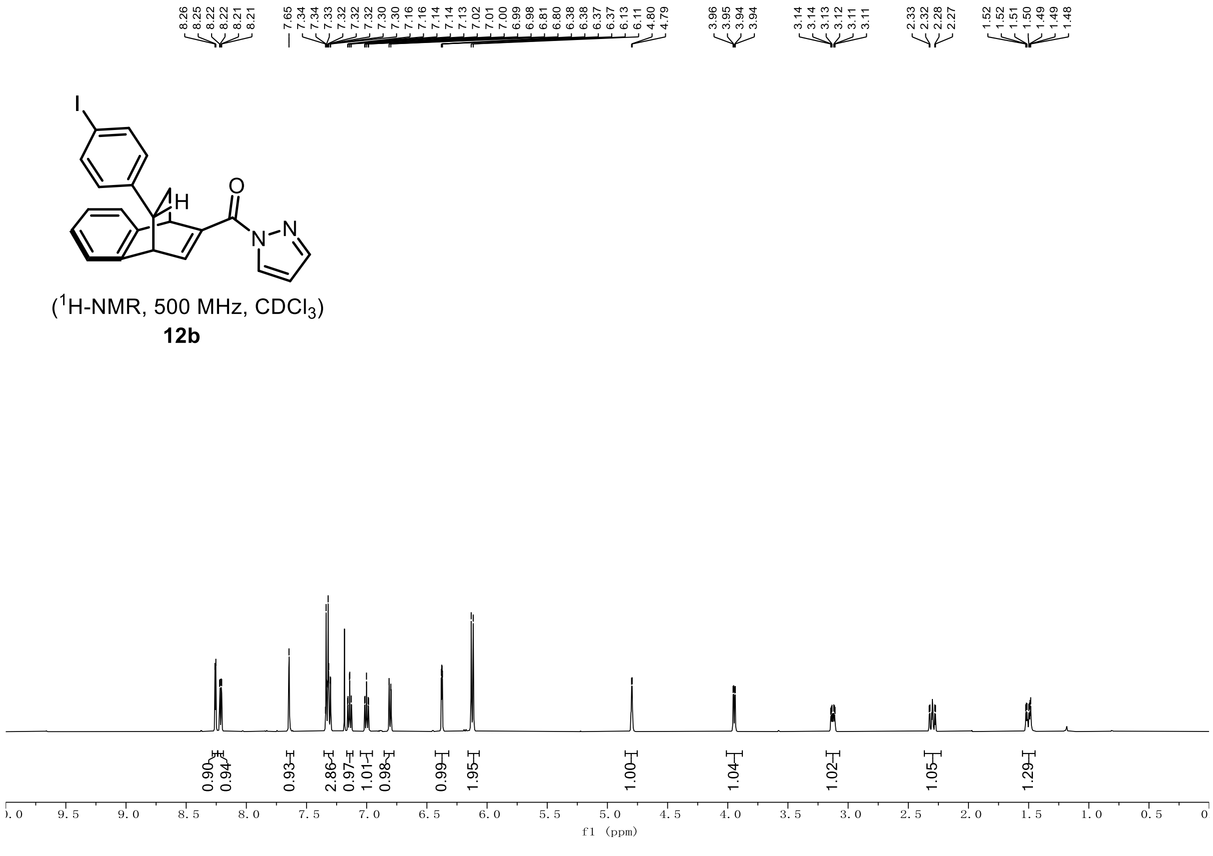

^{1}H-NMR Spectra of compound **12b.**

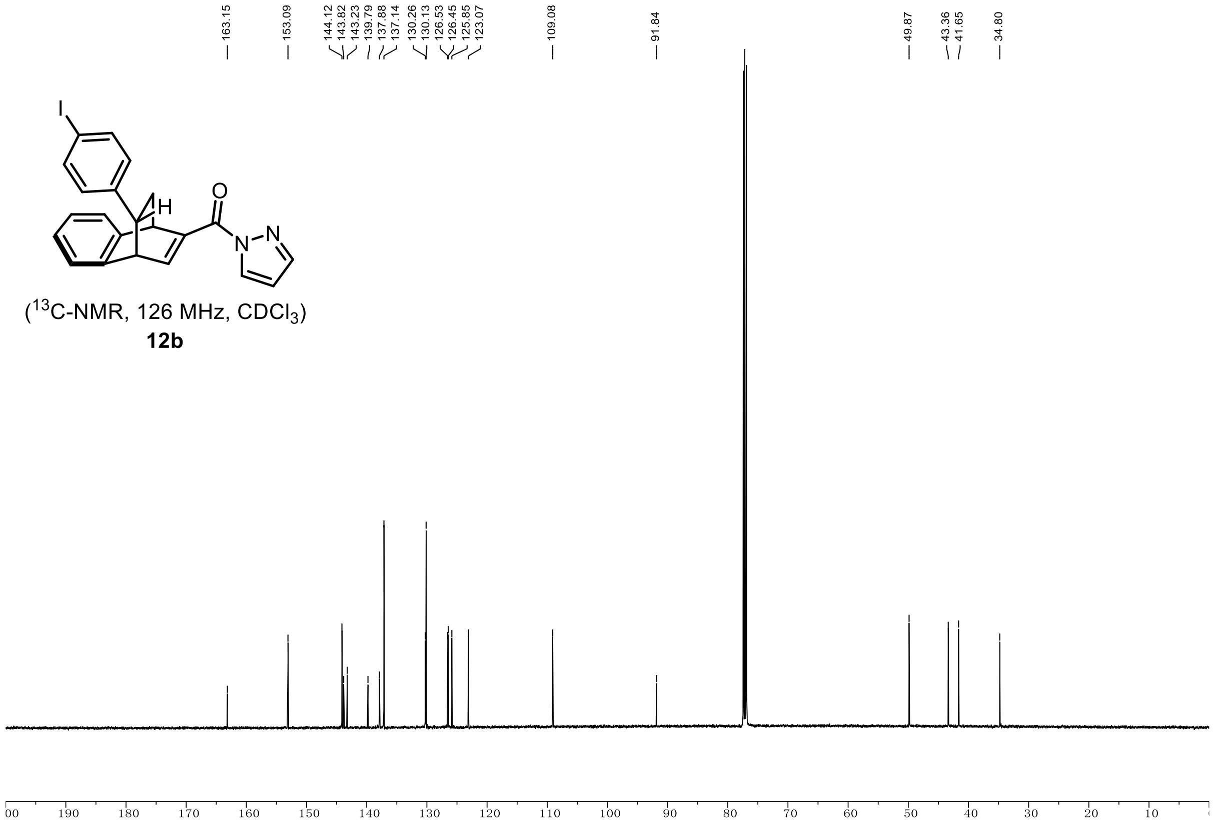

^{13}C-NMR Spectra of compound **12b.**

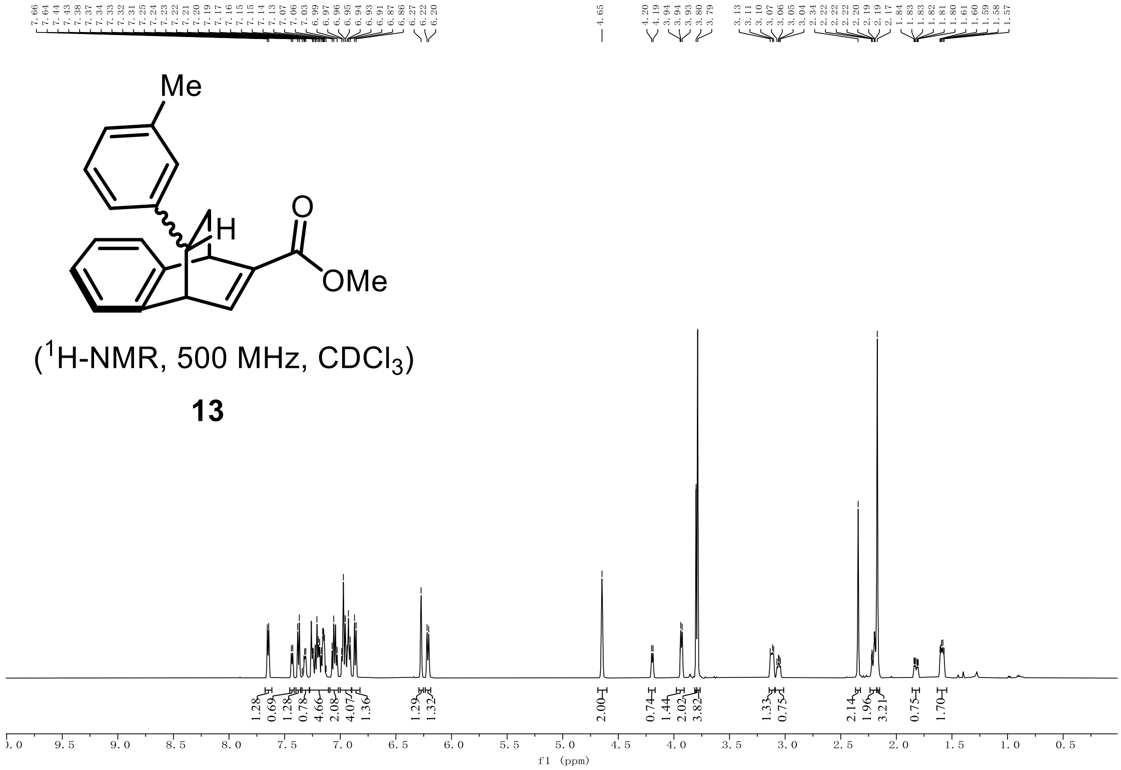

^{1}H-NMR Spectra of compound **13.**

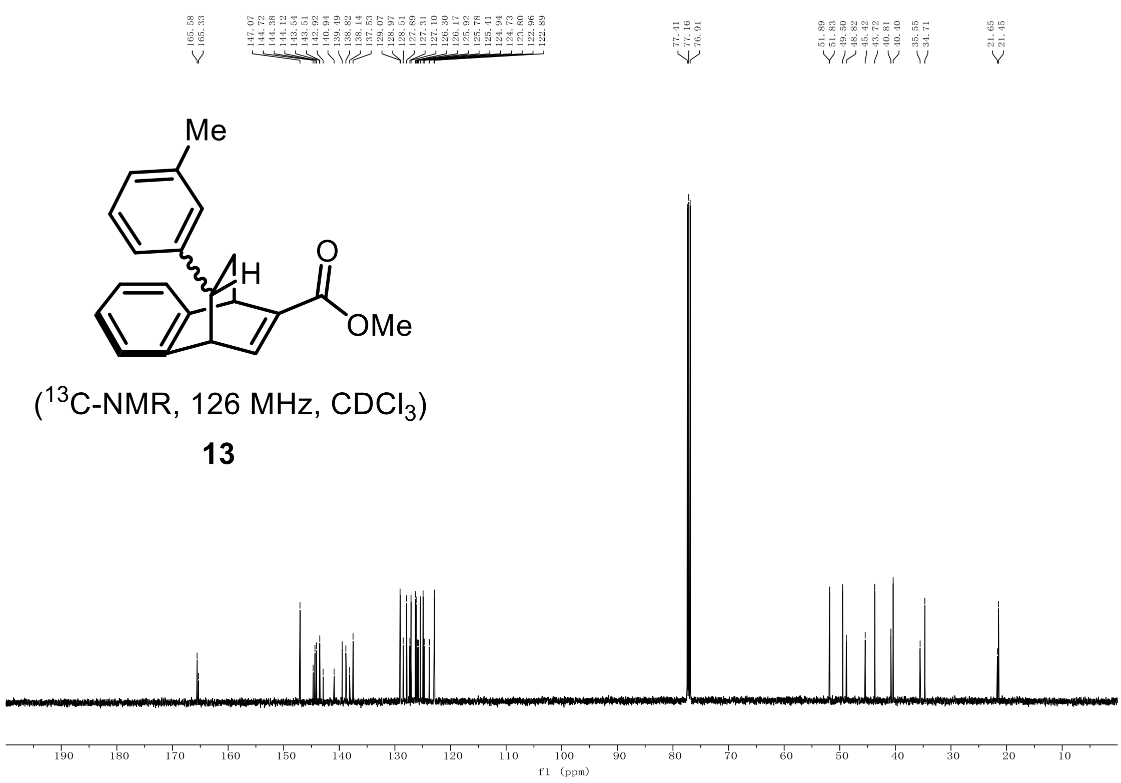

^{13}C-NMR Spectra of compound **13.**

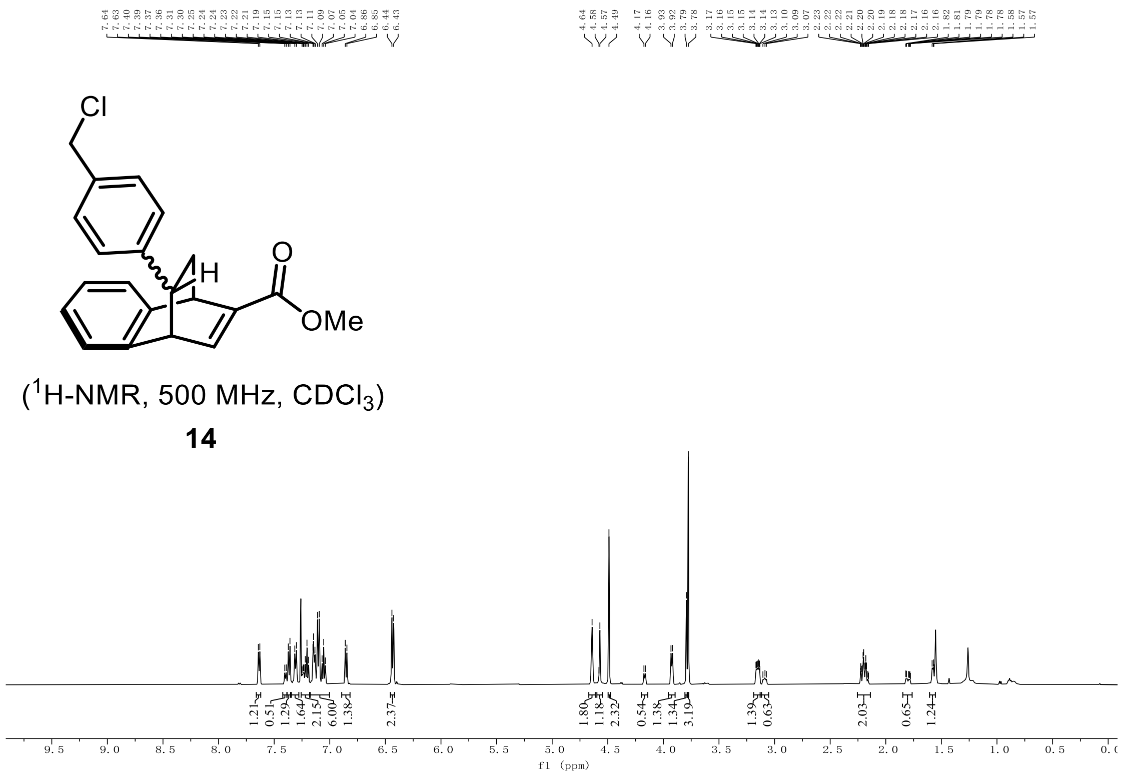

^{1}H-NMR Spectra of compound **14.**

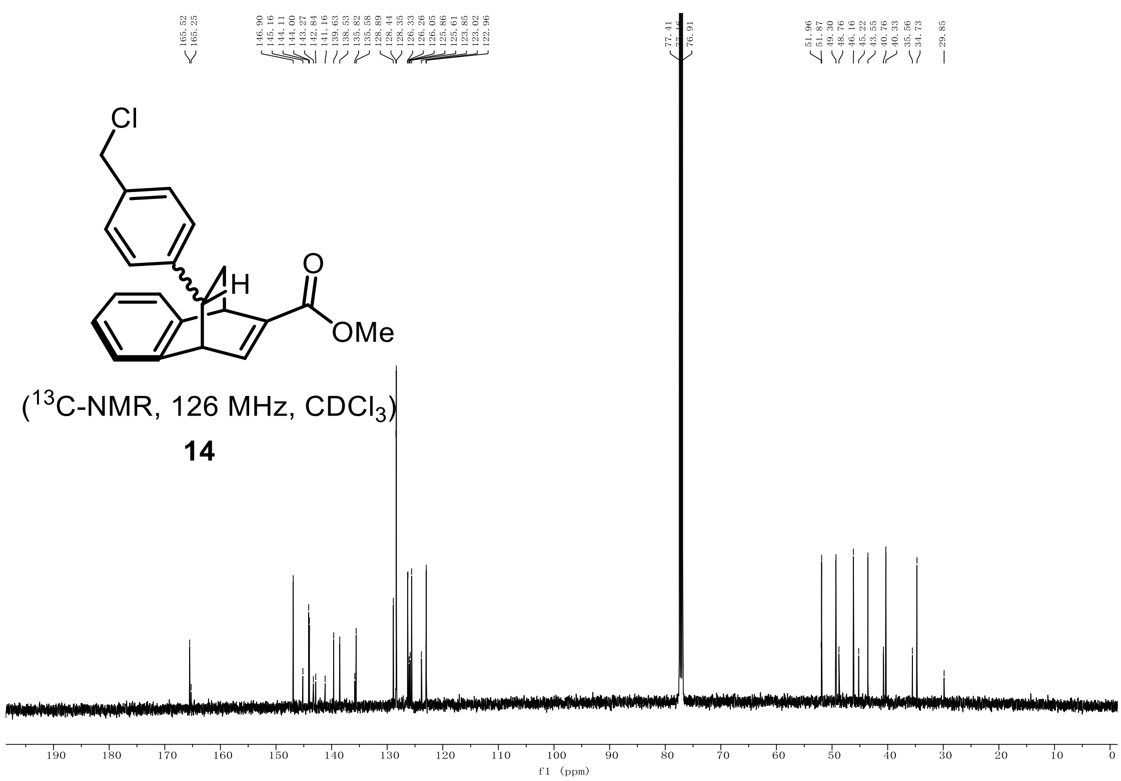

^{13}C-NMR Spectra of compound **14.**

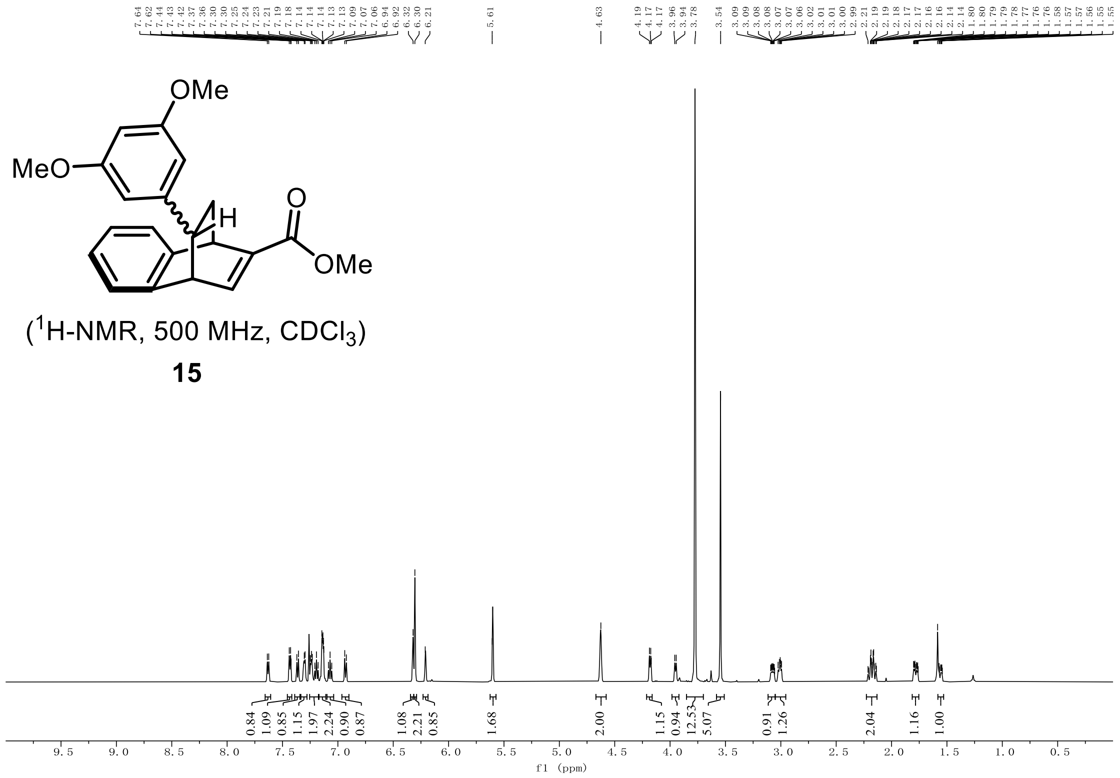

^{1}H-NMR Spectra of compound **15.**

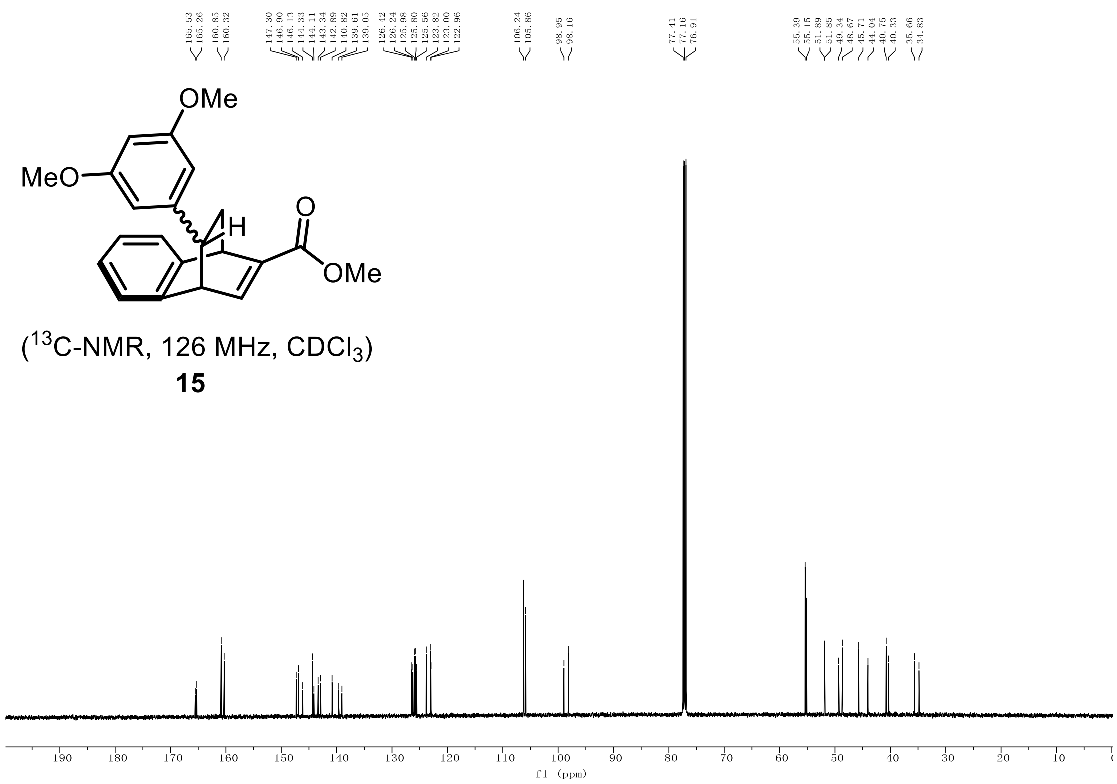

^{13}C-NMR Spectra of compound **15.**

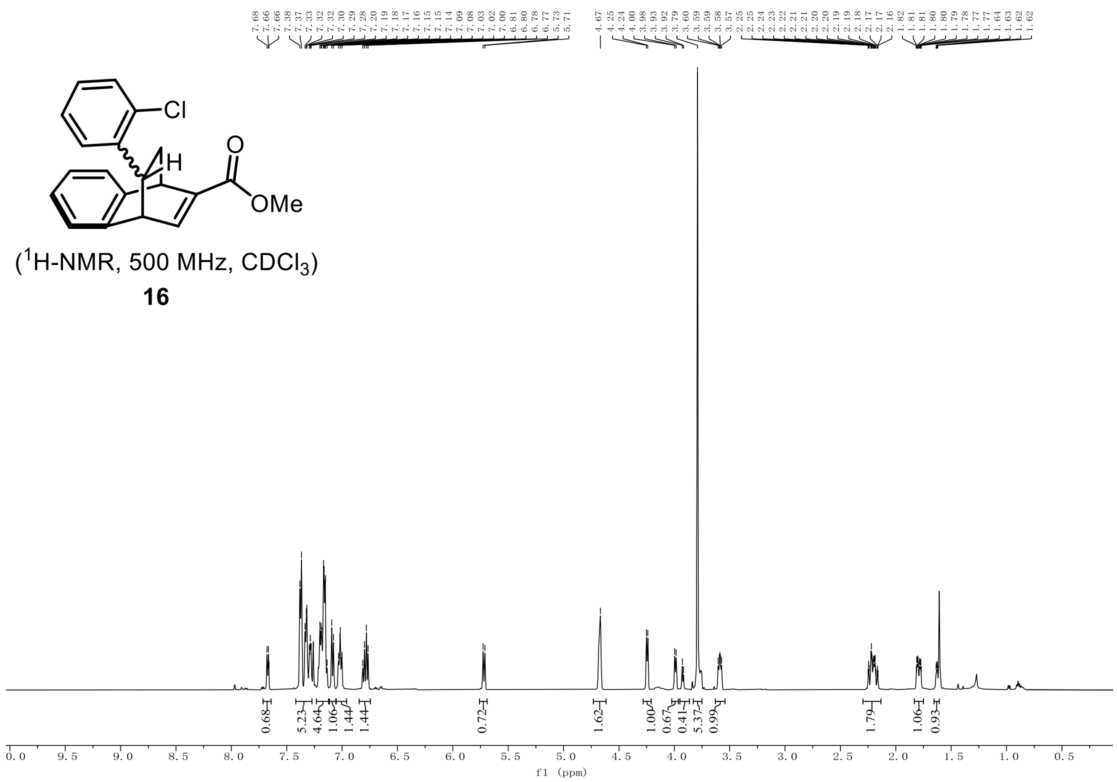

^{1}H-NMR Spectra of compound **16.**

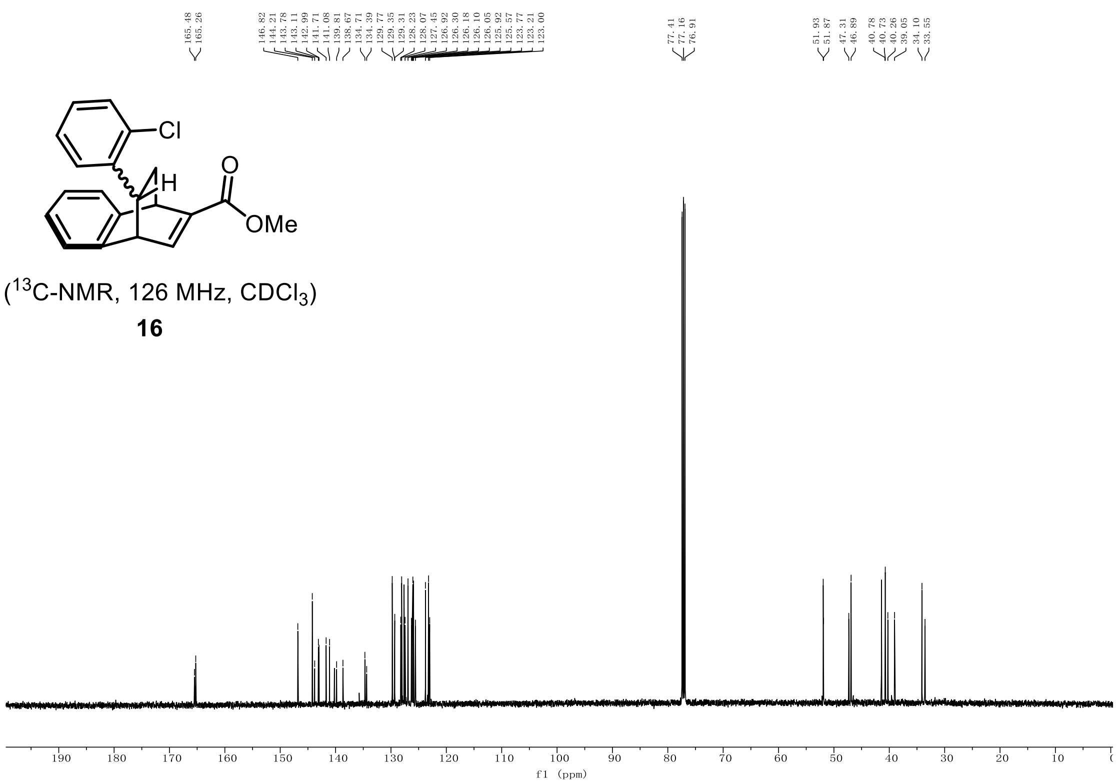

^{13}C-NMR Spectra of compound **16.**

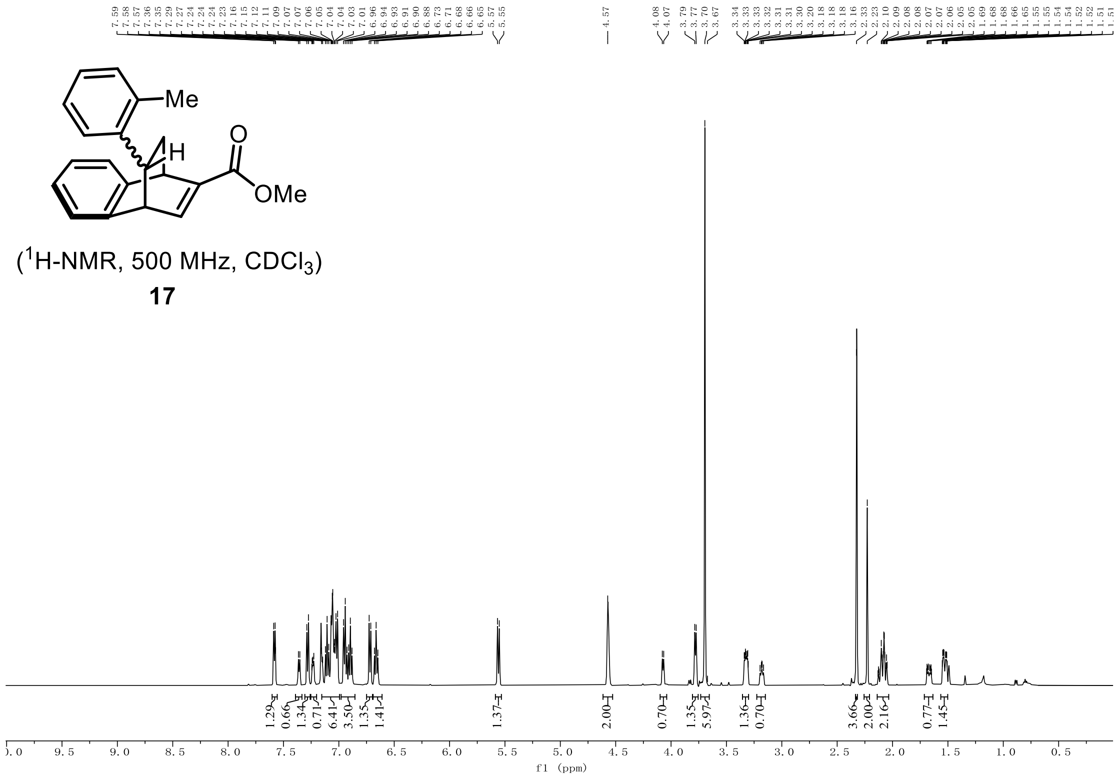

^{1}H-NMR Spectra of compound **17.**

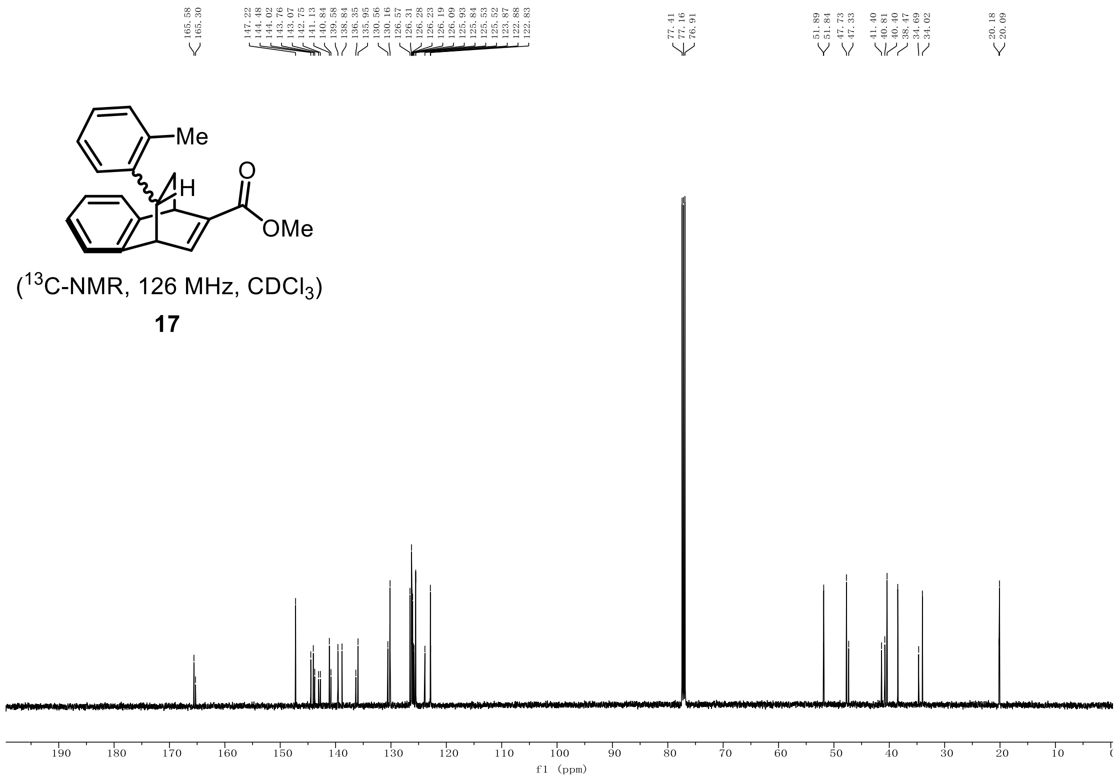

^{13}C-NMR Spectra of compound **17.**

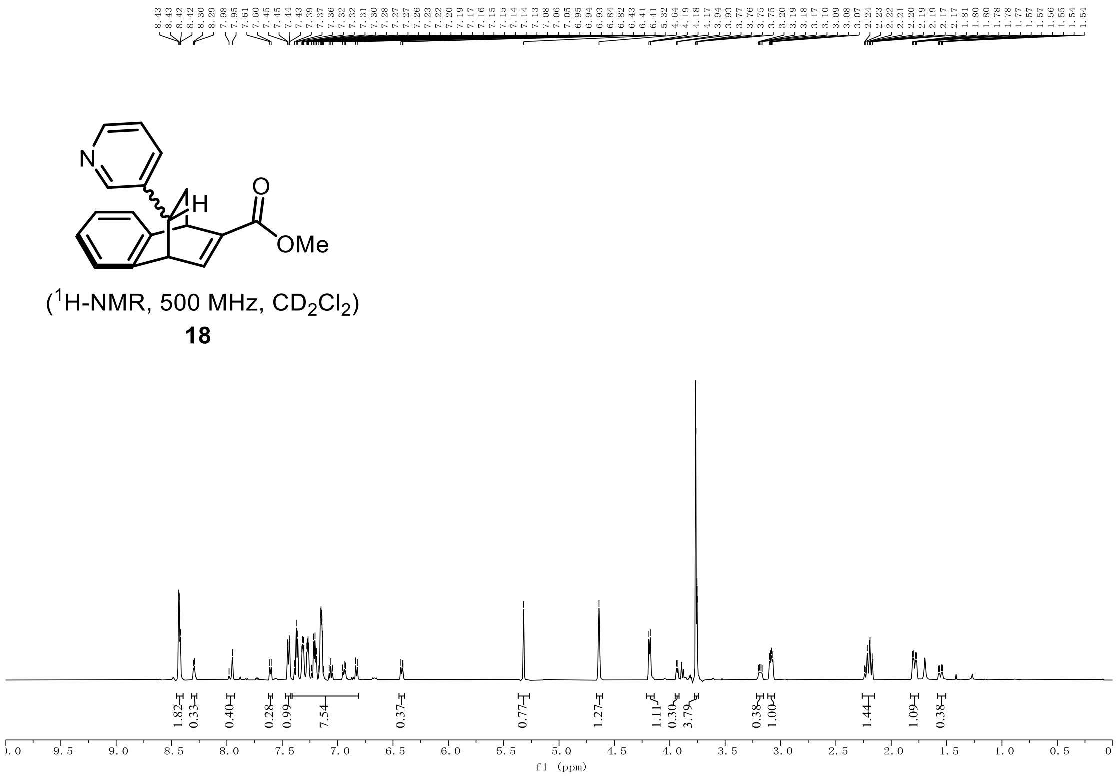

^{1}H-NMR Spectra of compound **18.**

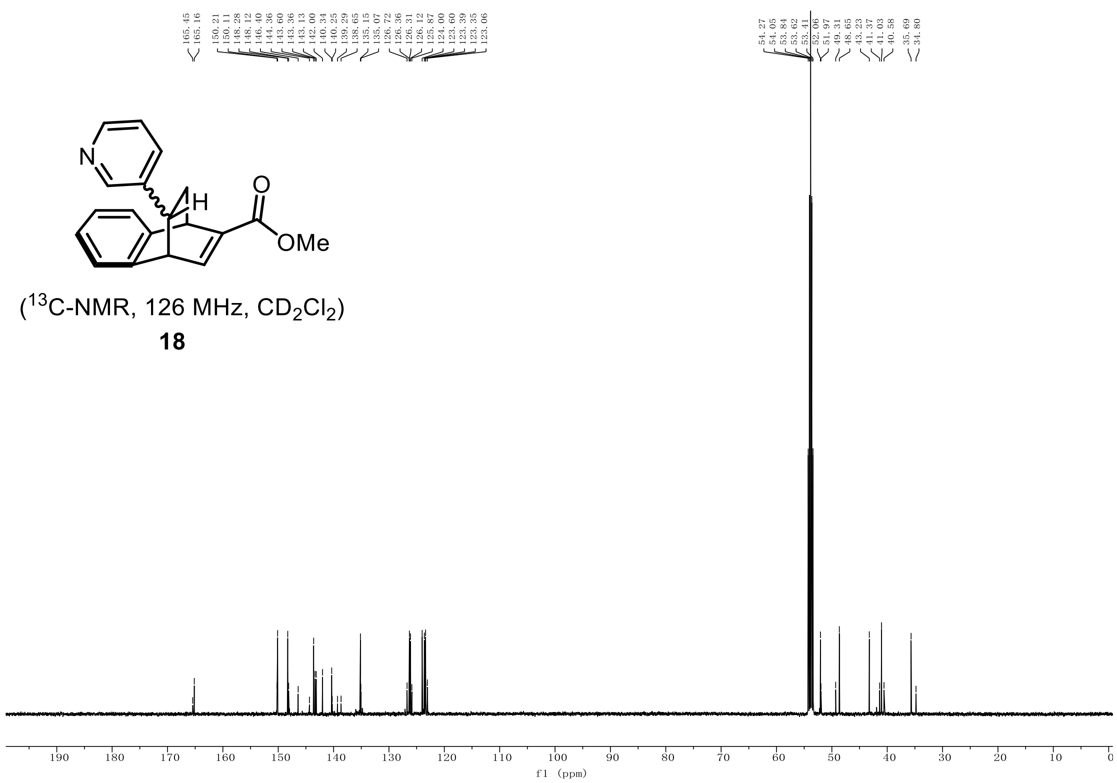

^{13}C-NMR Spectra of compound **18.**

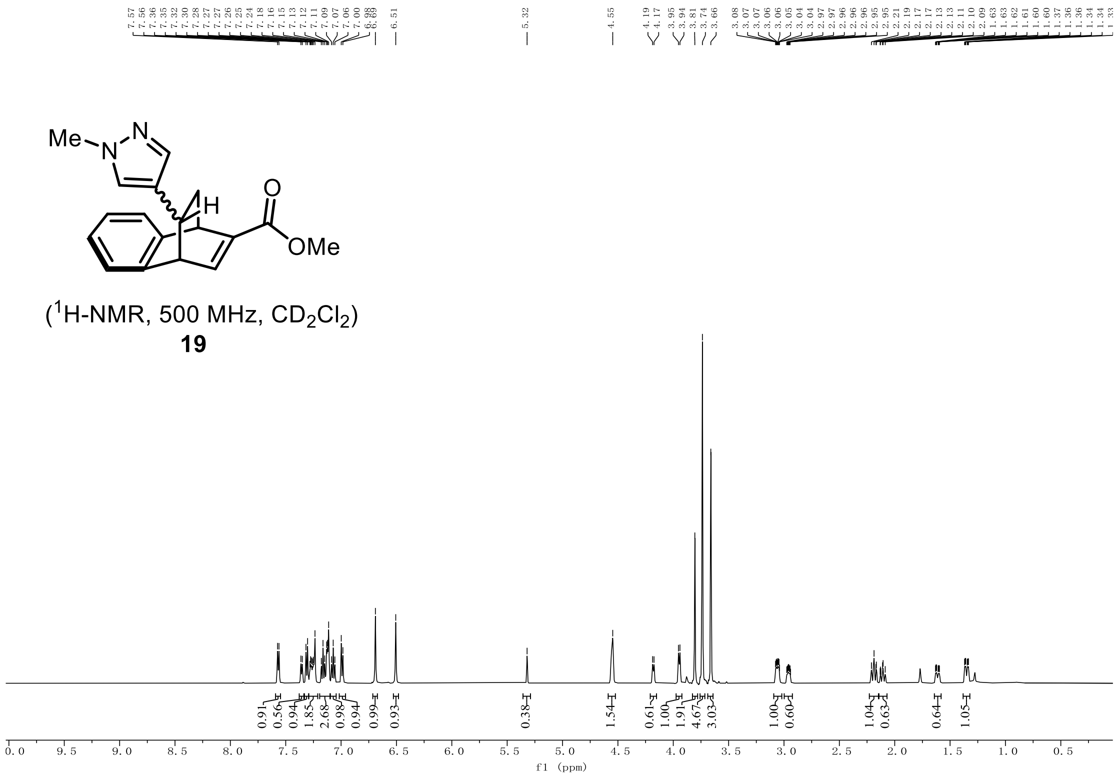

^{1}H-NMR Spectra of compound **19.**

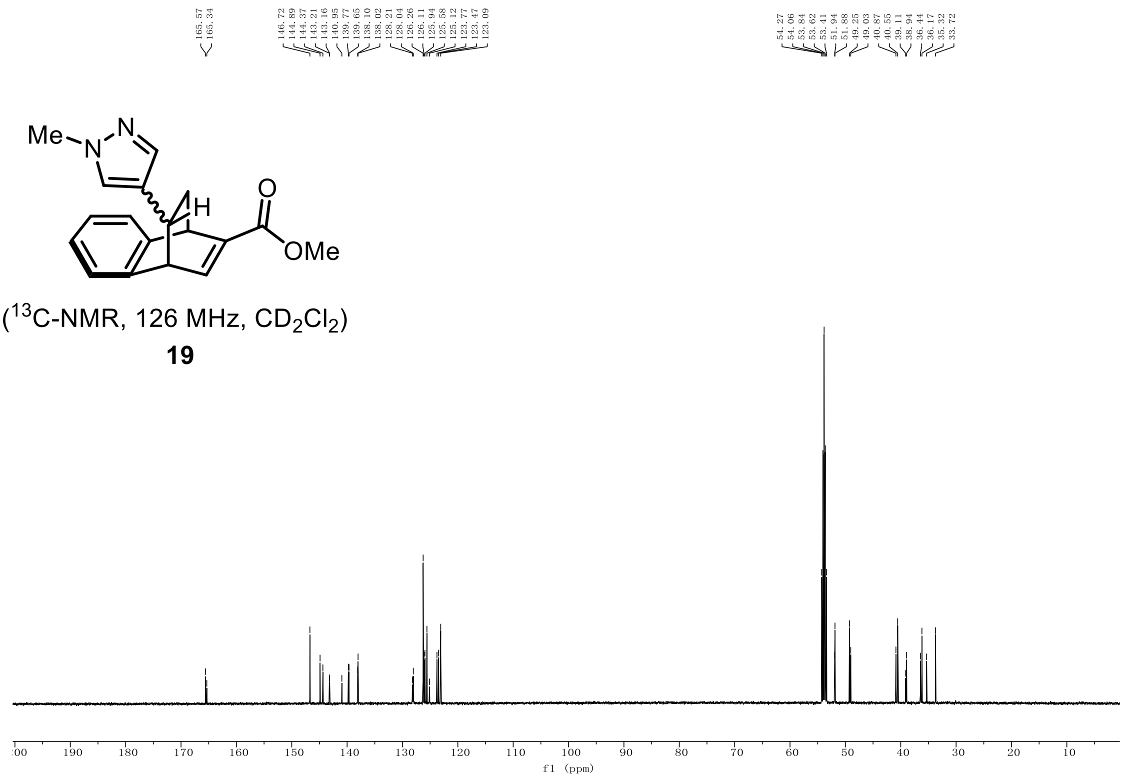

^{13}C-NMR Spectra of compound **19.**

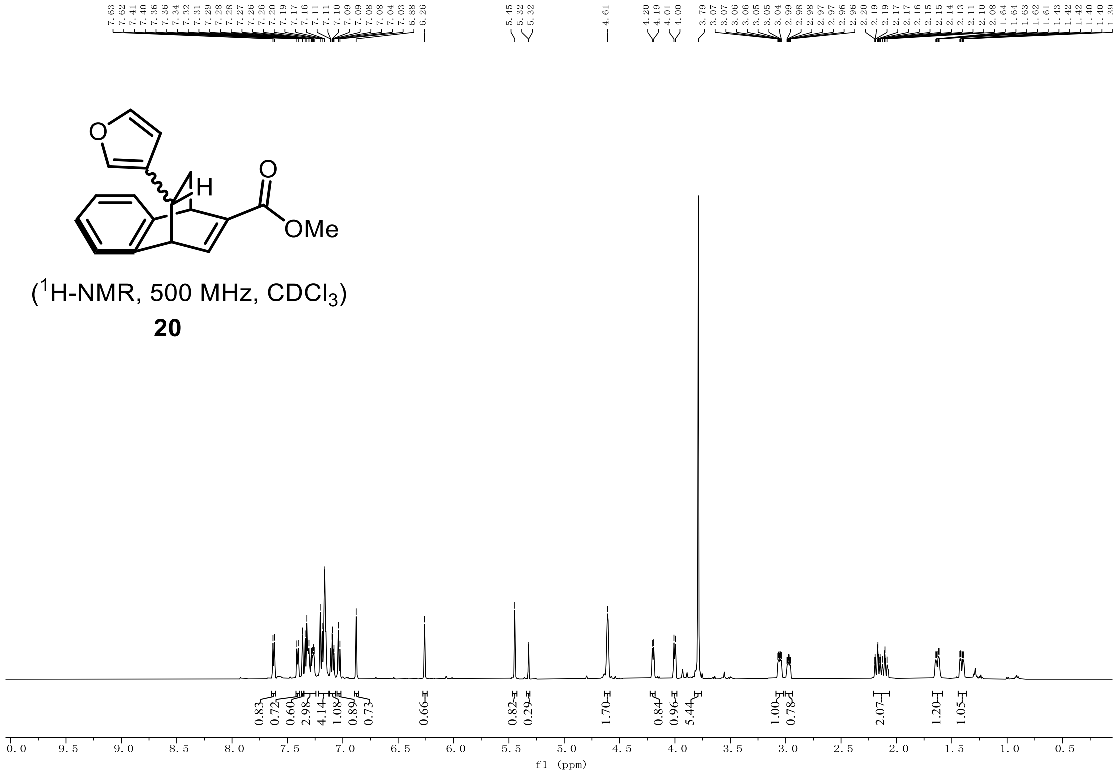

^{1}H-NMR Spectra of compound **20.**

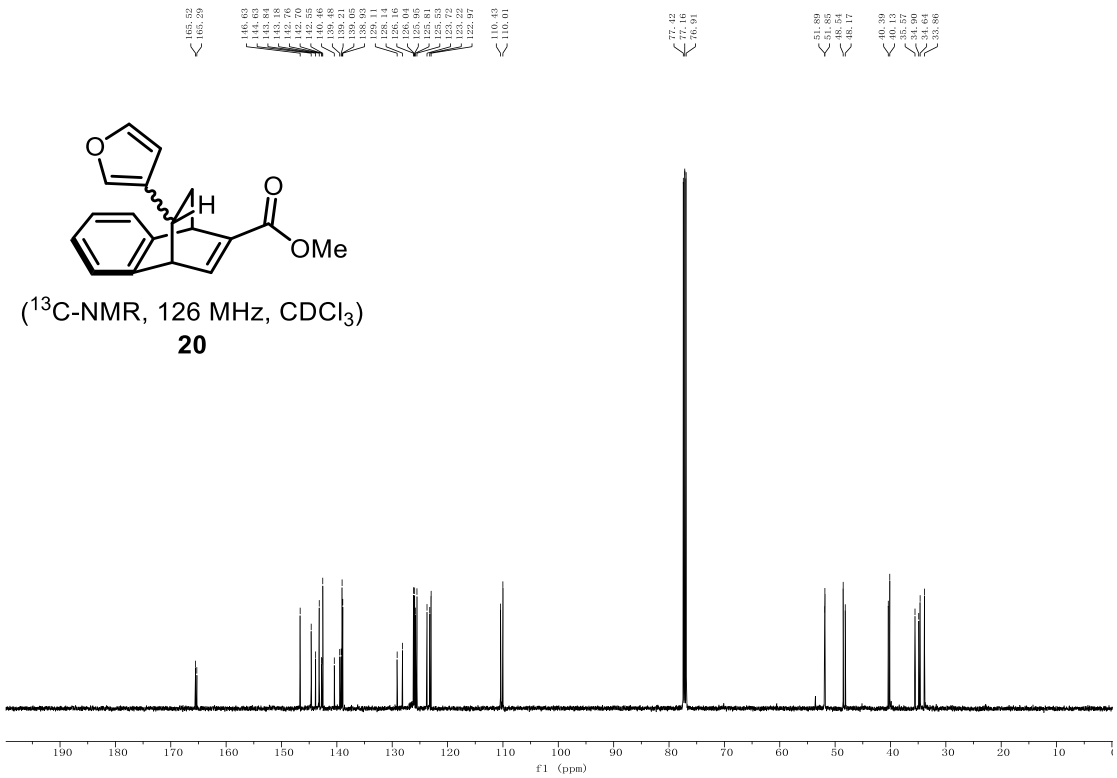

^{13}C-NMR Spectra of compound **20.**

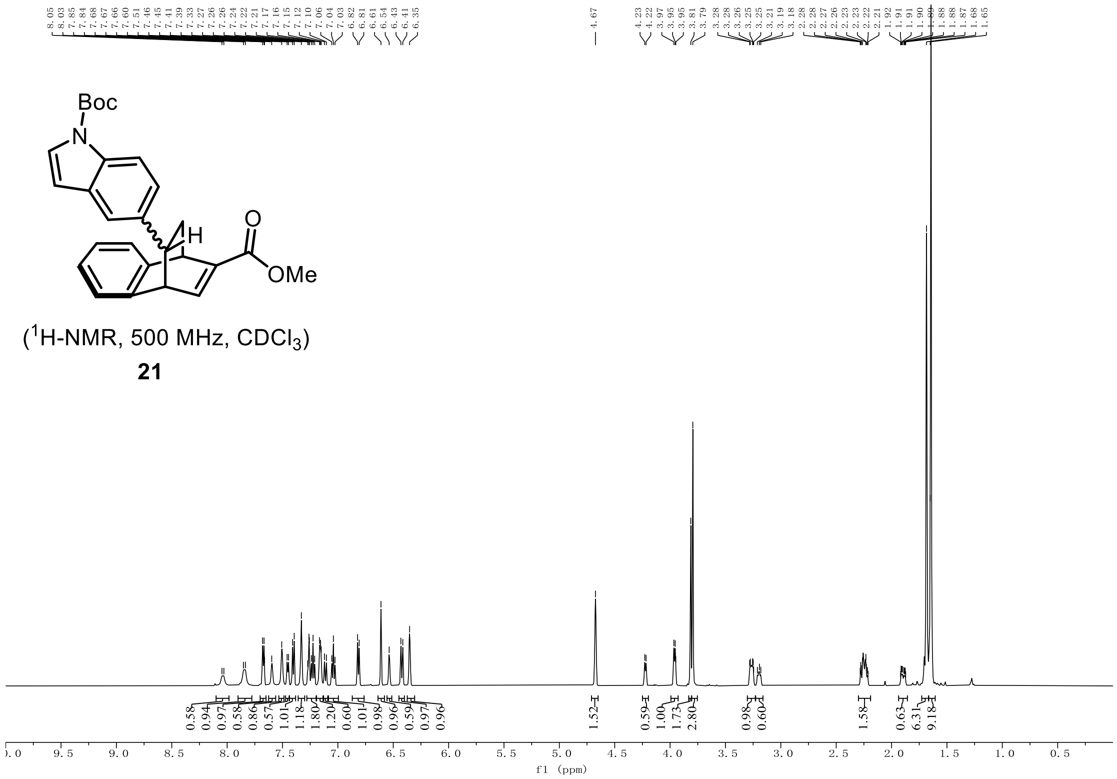

^{1}H-NMR Spectra of compound **21.**

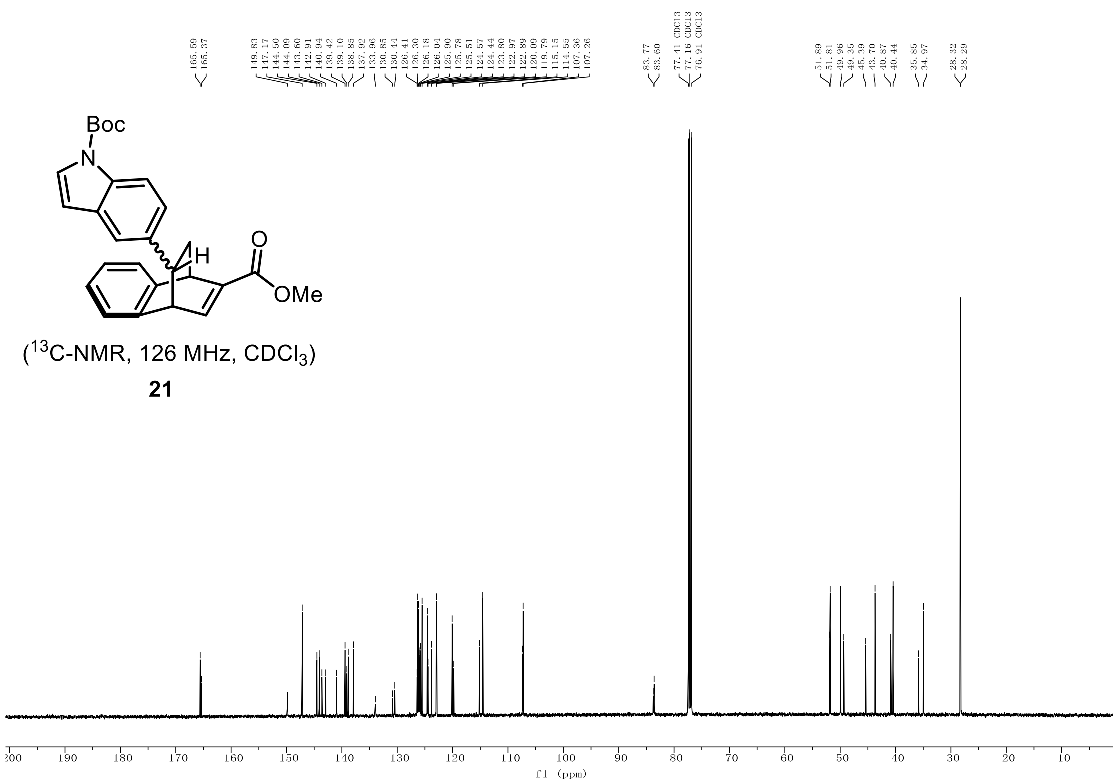

^{13}C-NMR Spectra of compound **21.**

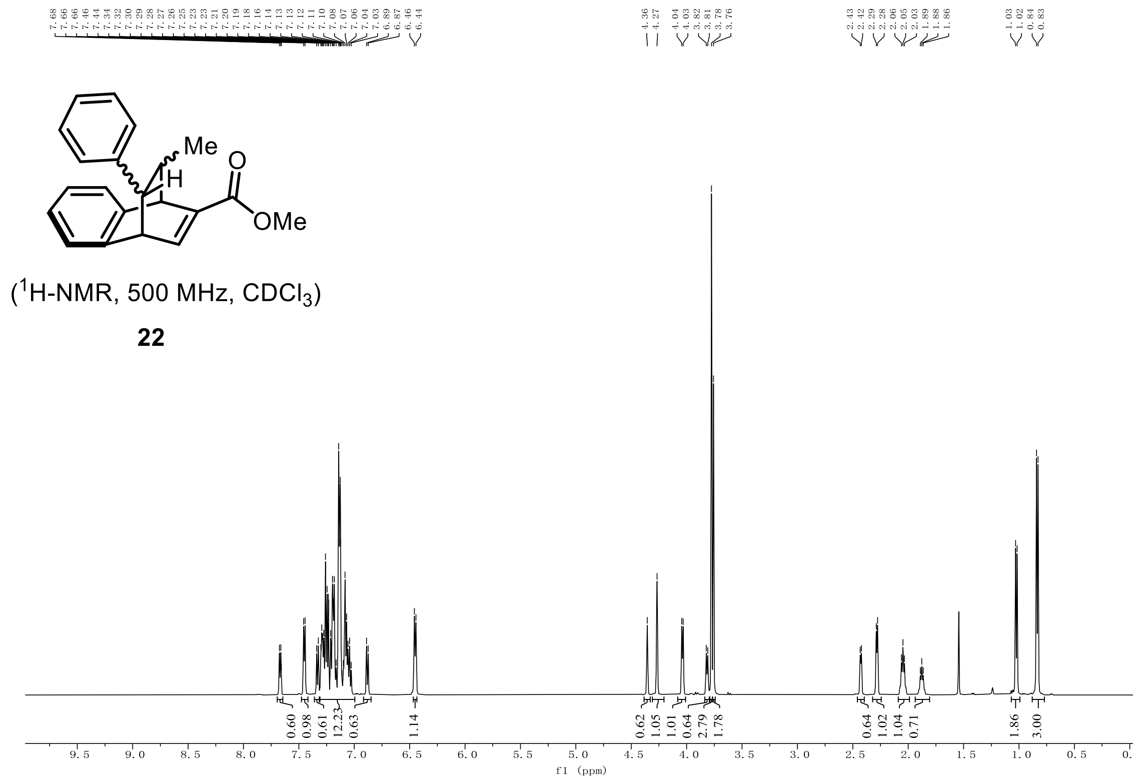

^{1}H-NMR Spectra of compound **22.**

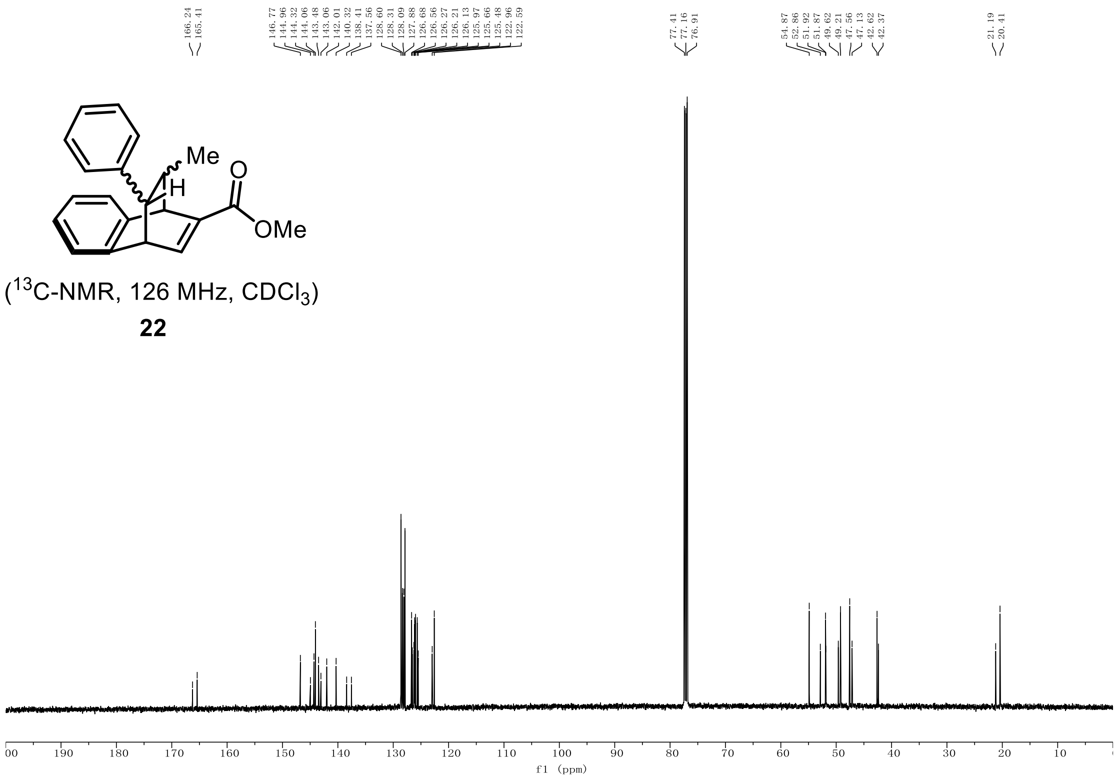

^{13}C-NMR Spectra of compound **22.**

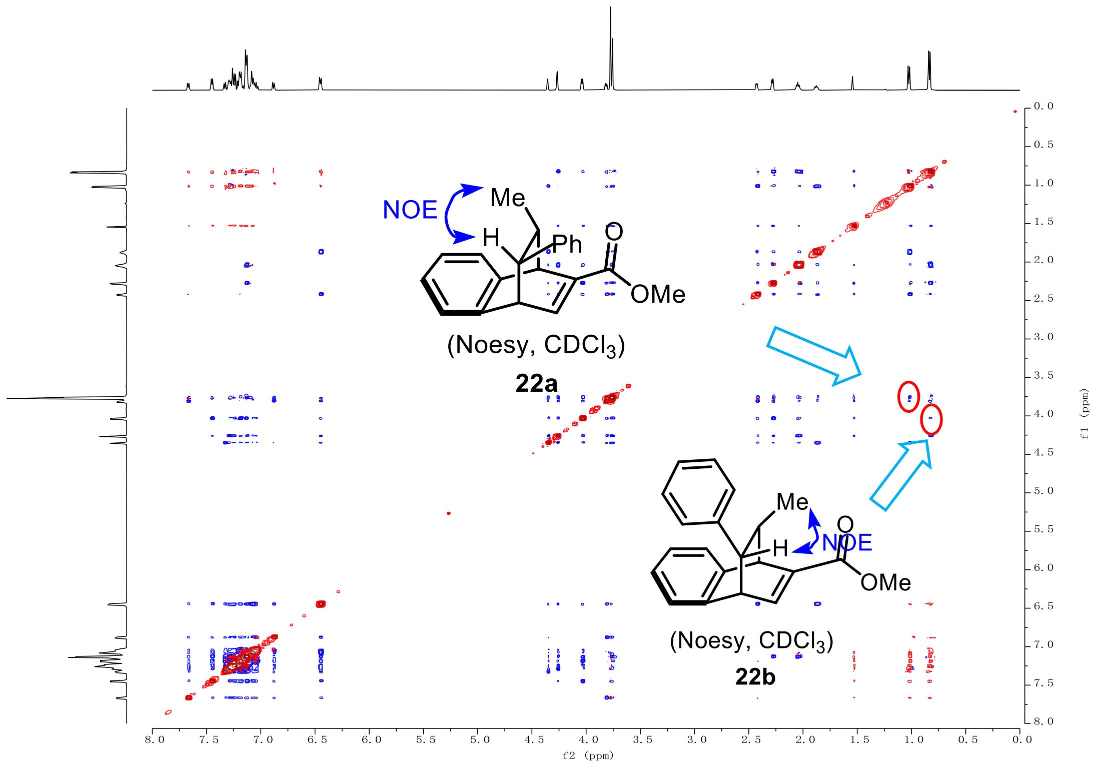

Noesy Spectra of compound **22a, 22b.**

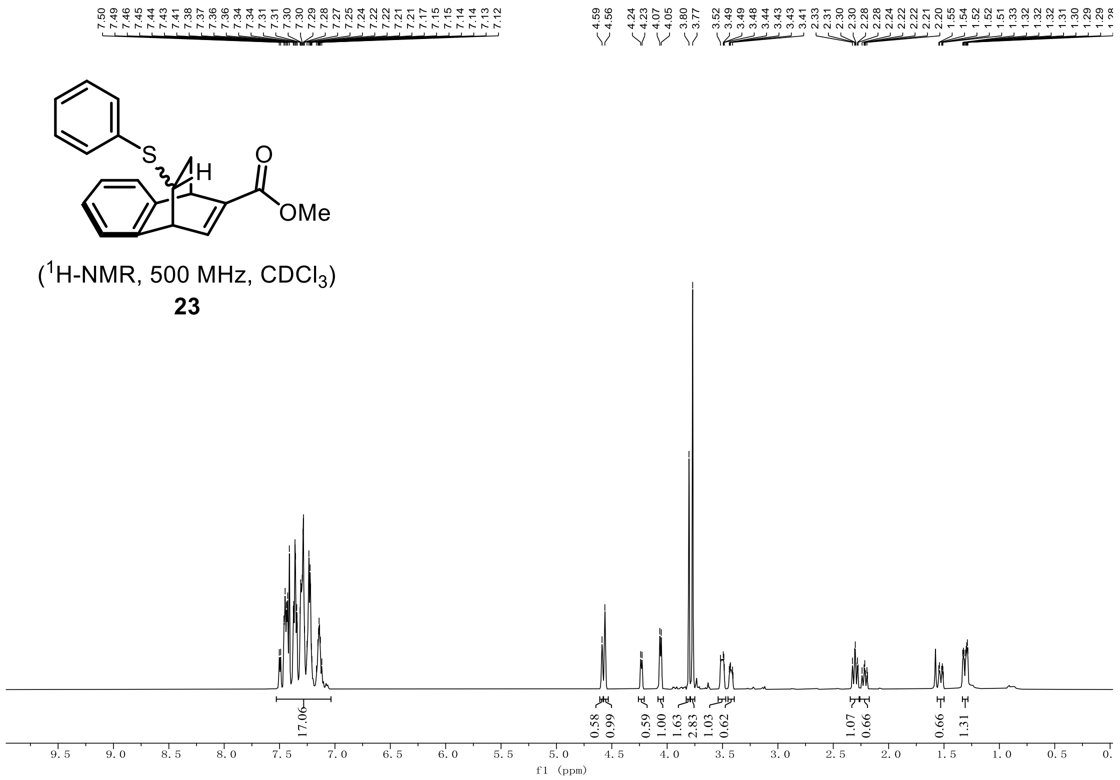

^{1}H-NMR Spectra of compound **23.**

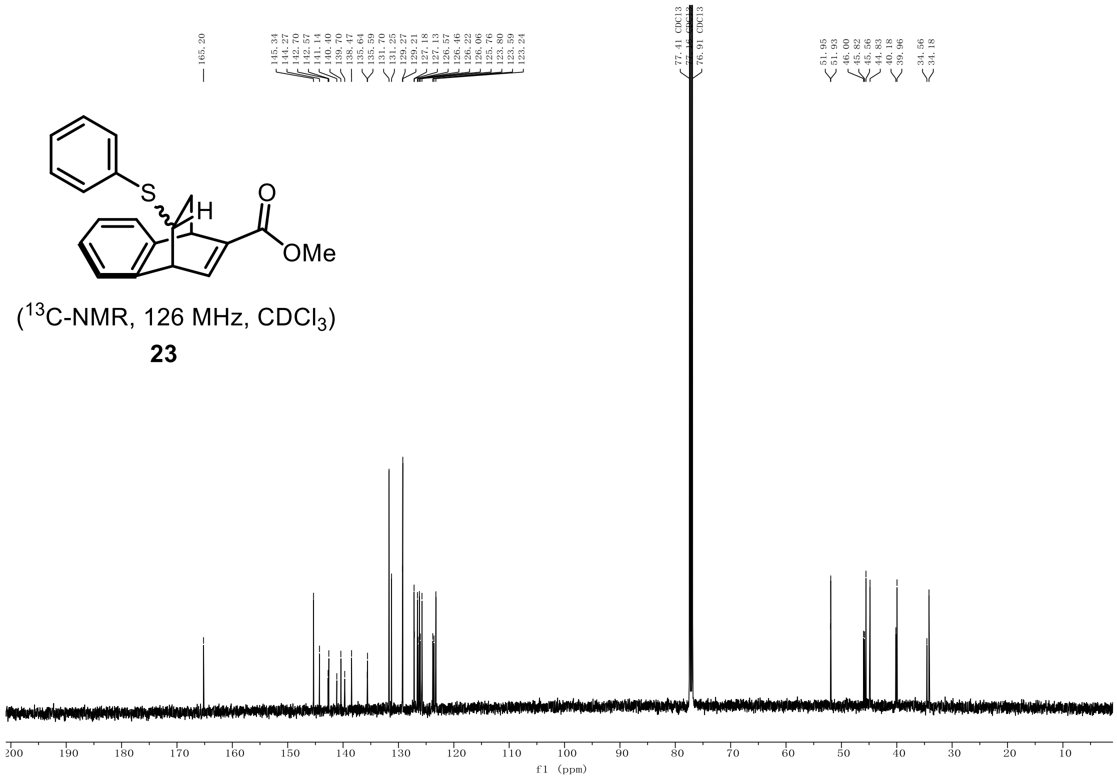

^{13}C-NMR Spectra of compound **23.**

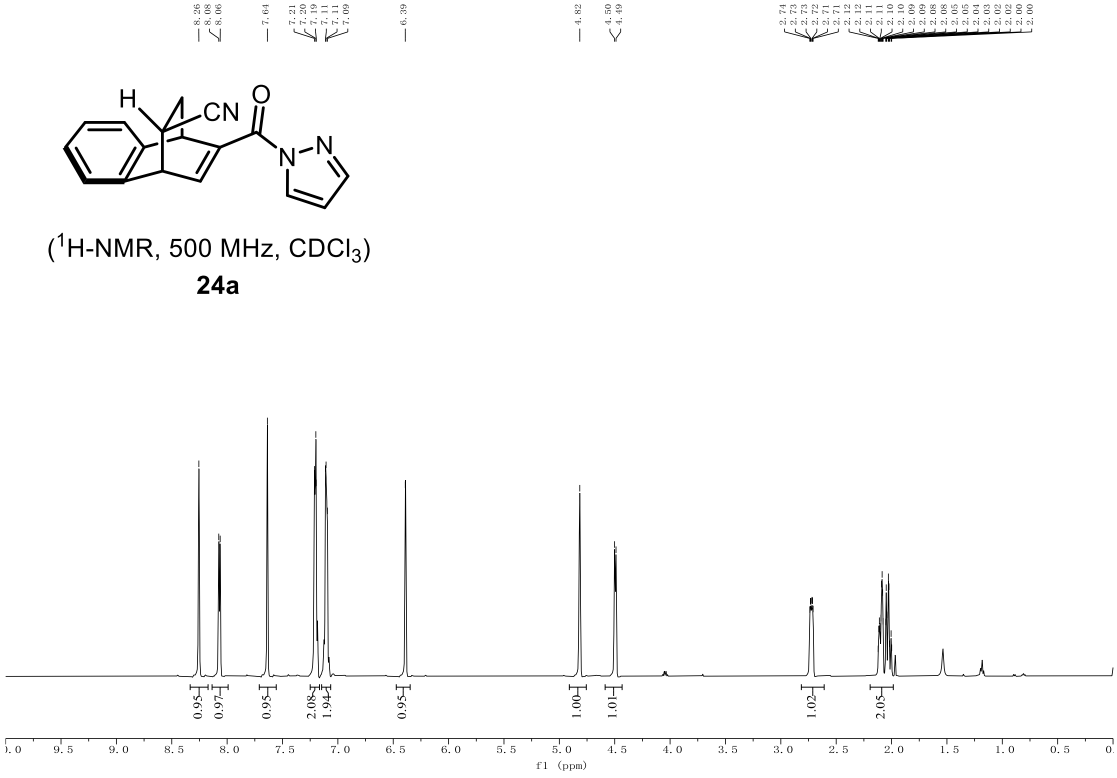

^{1}H-NMR Spectra of compound **24a.**

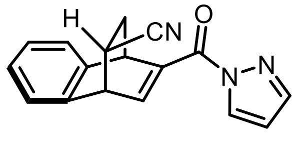

(^{13}C-NMR, 126 MHz, $CDCl_3$)
24a

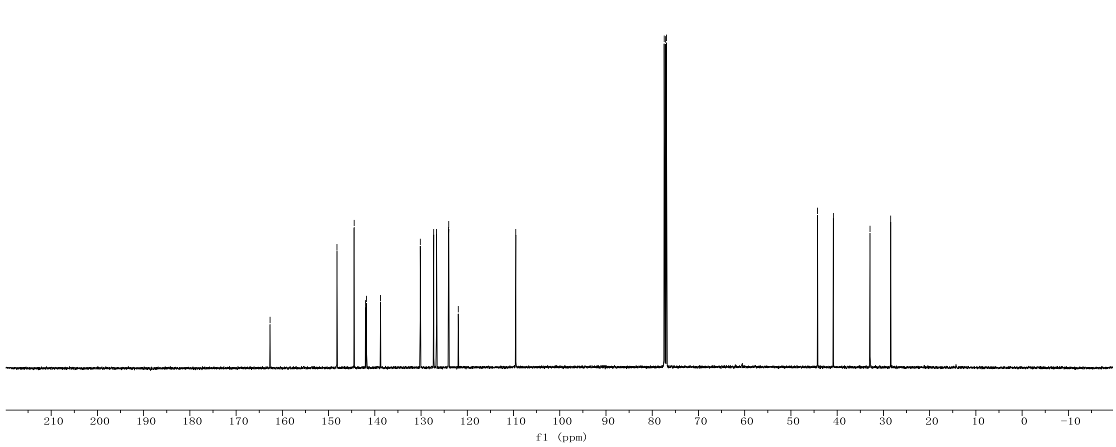

^{13}C-NMR Spectra of compound **24a.**

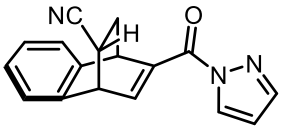

(^{1}H-NMR, 500 MHz, $CDCl_3$)
24b

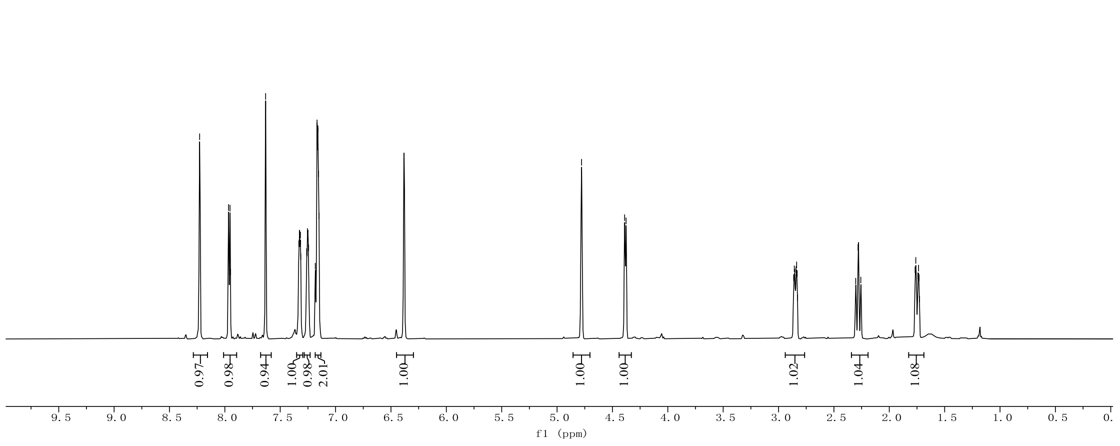

^{1}H-NMR Spectra of compound **24b.**

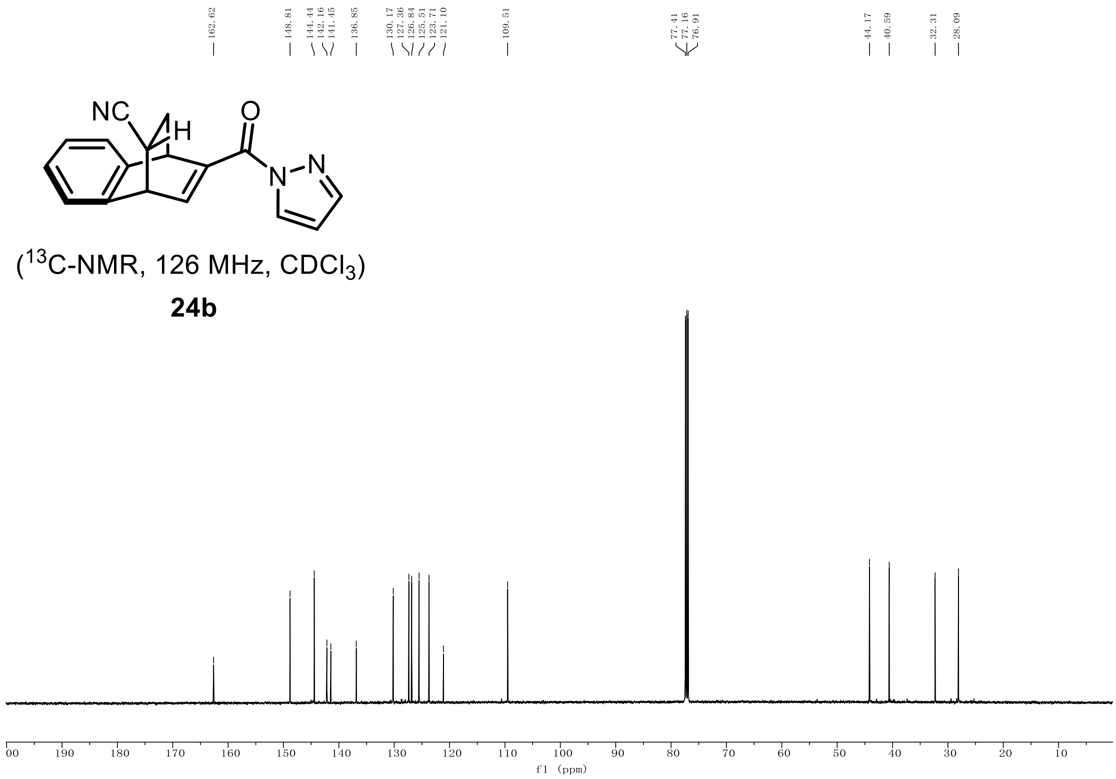

^{13}C-NMR Spectra of compound **24b.**

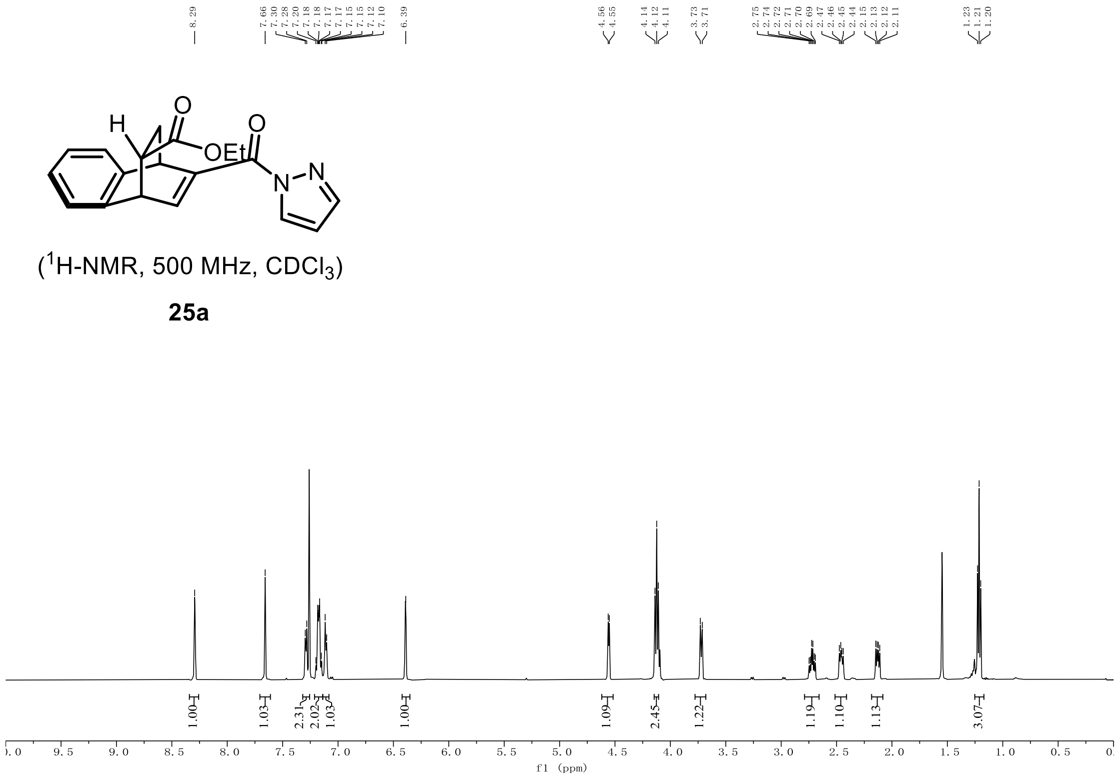

^{1}H-NMR Spectra of compound **25a.**

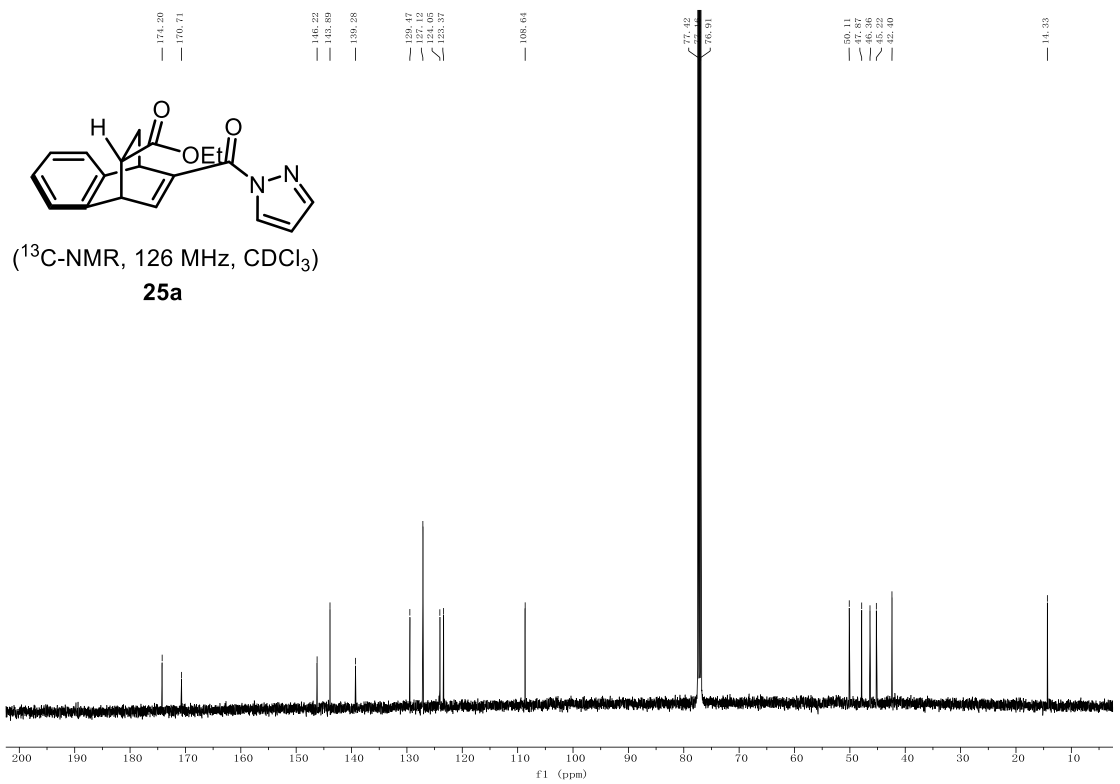

^{13}C-NMR Spectra of compound **25a.**

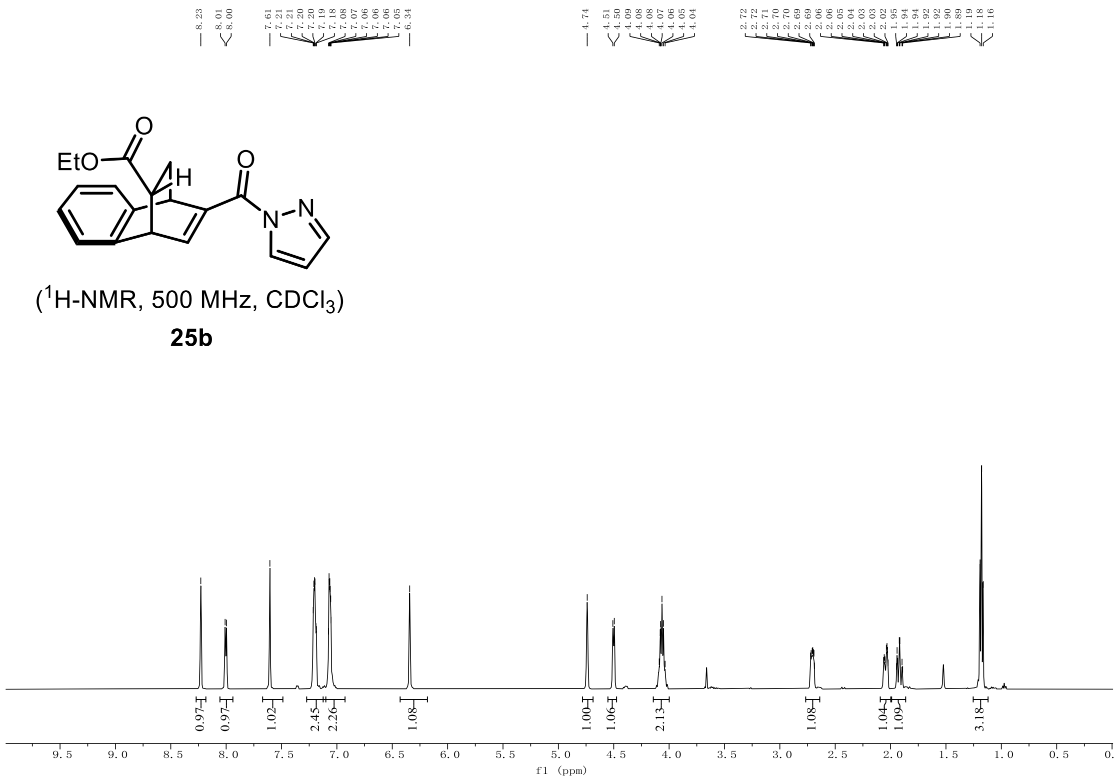

^{1}H-NMR Spectra of compound **25b.**

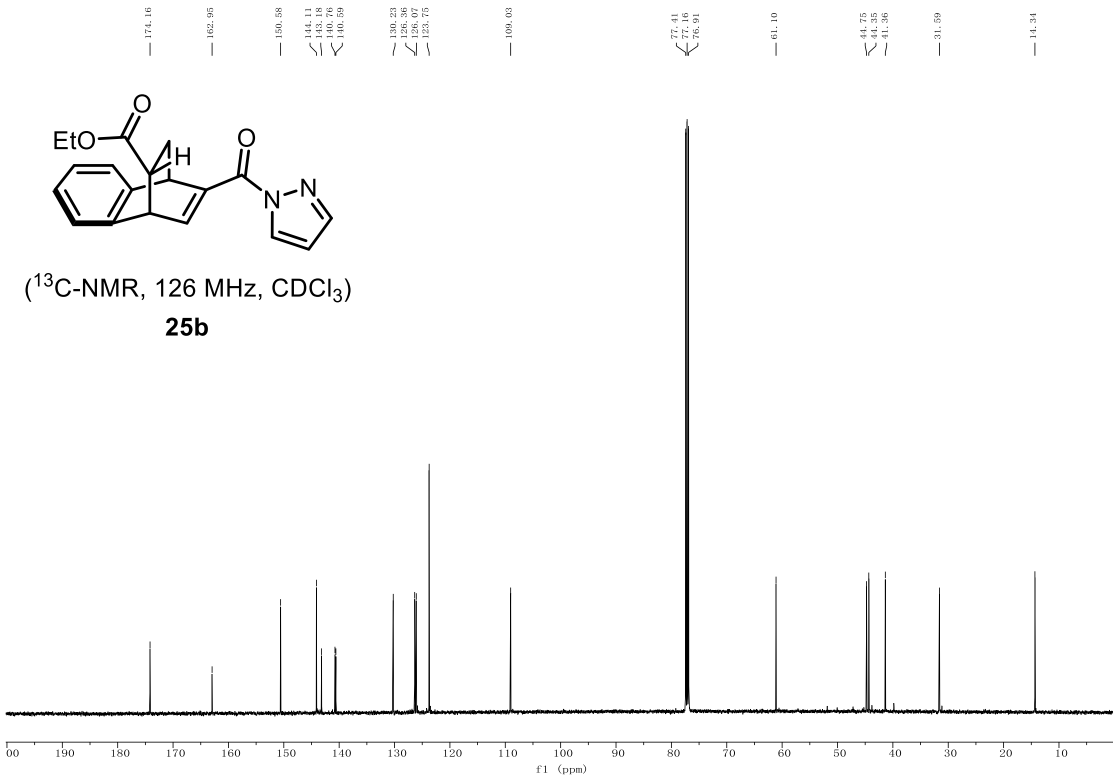

^{13}C-NMR Spectra of compound **25b.**

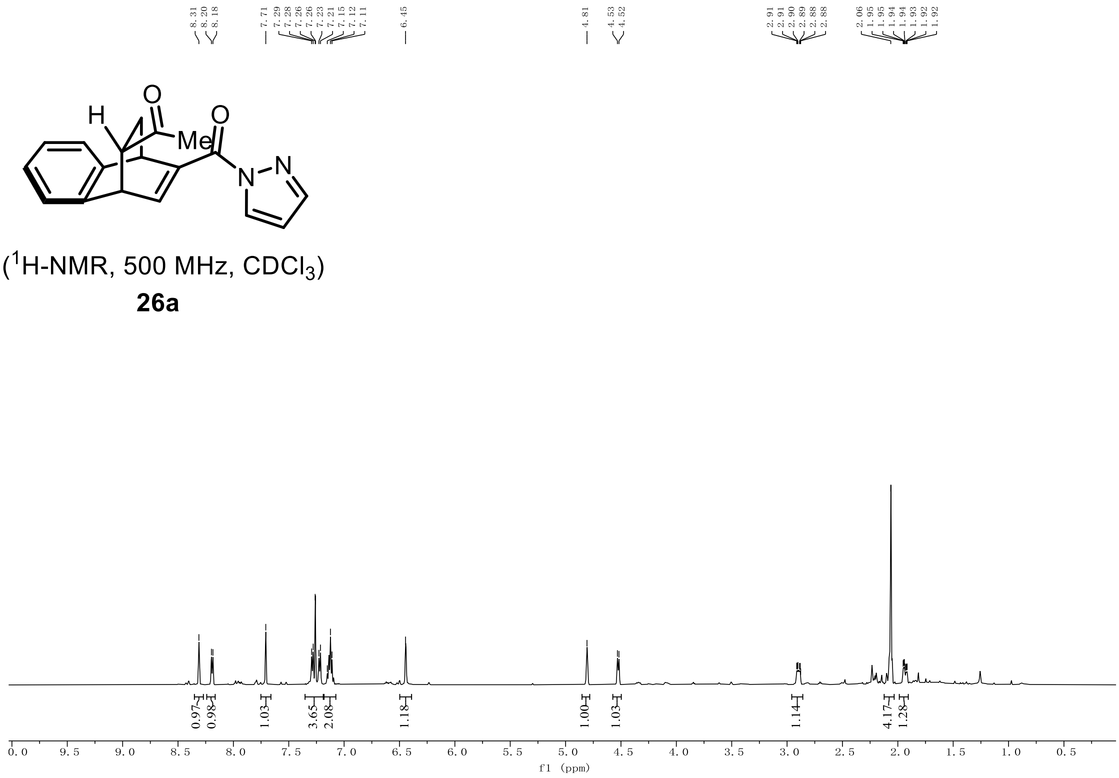

^{1}H-NMR Spectra of compound **26a.**

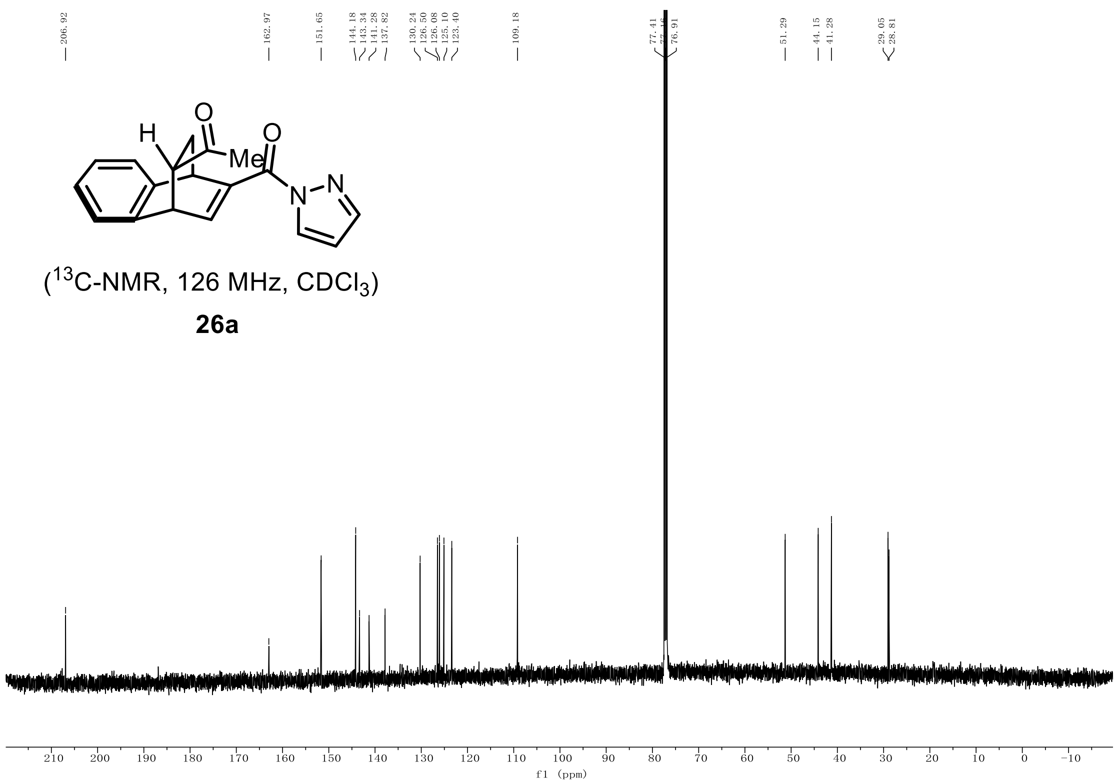

^{13}C-NMR Spectra of compound **26a.**

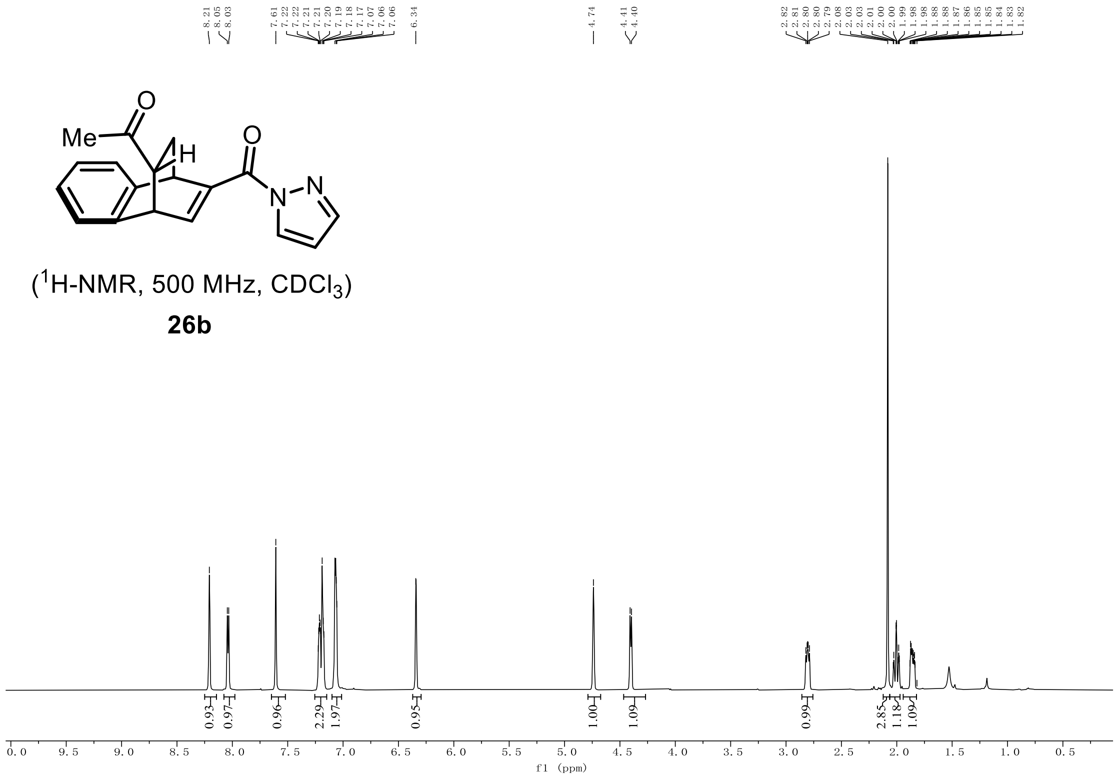

^{1}H-NMR Spectra of compound **26b.**

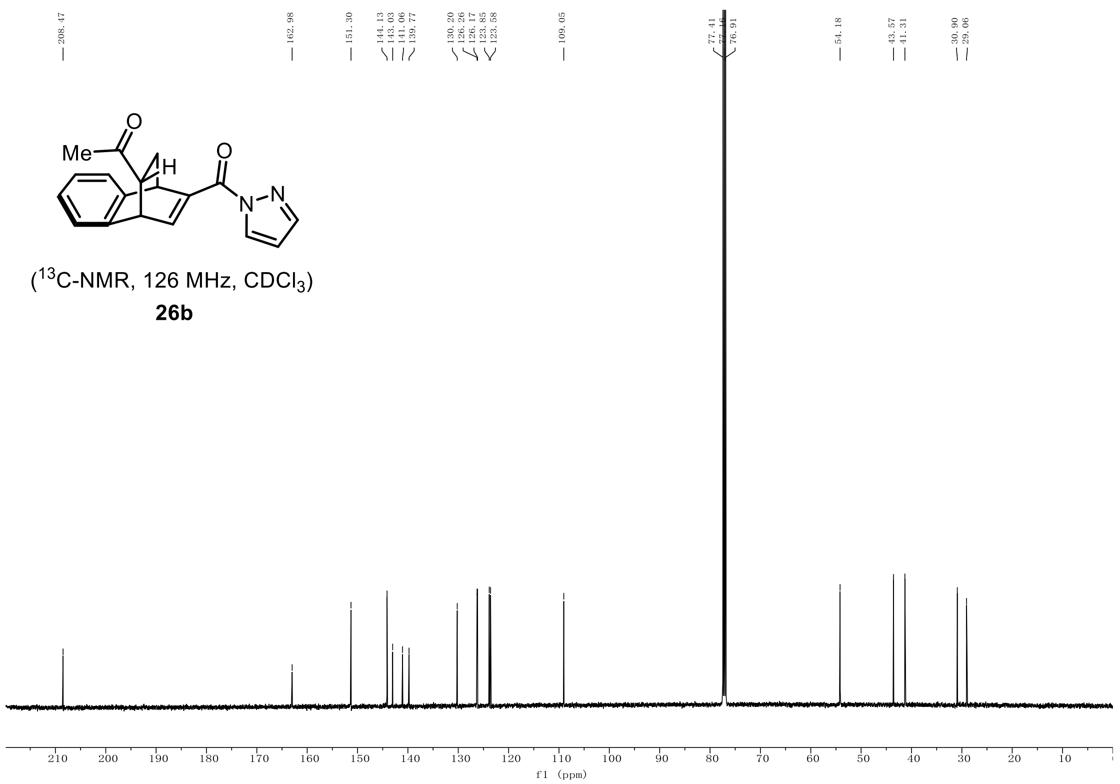

^{13}C-NMR Spectra of compound **26b.**

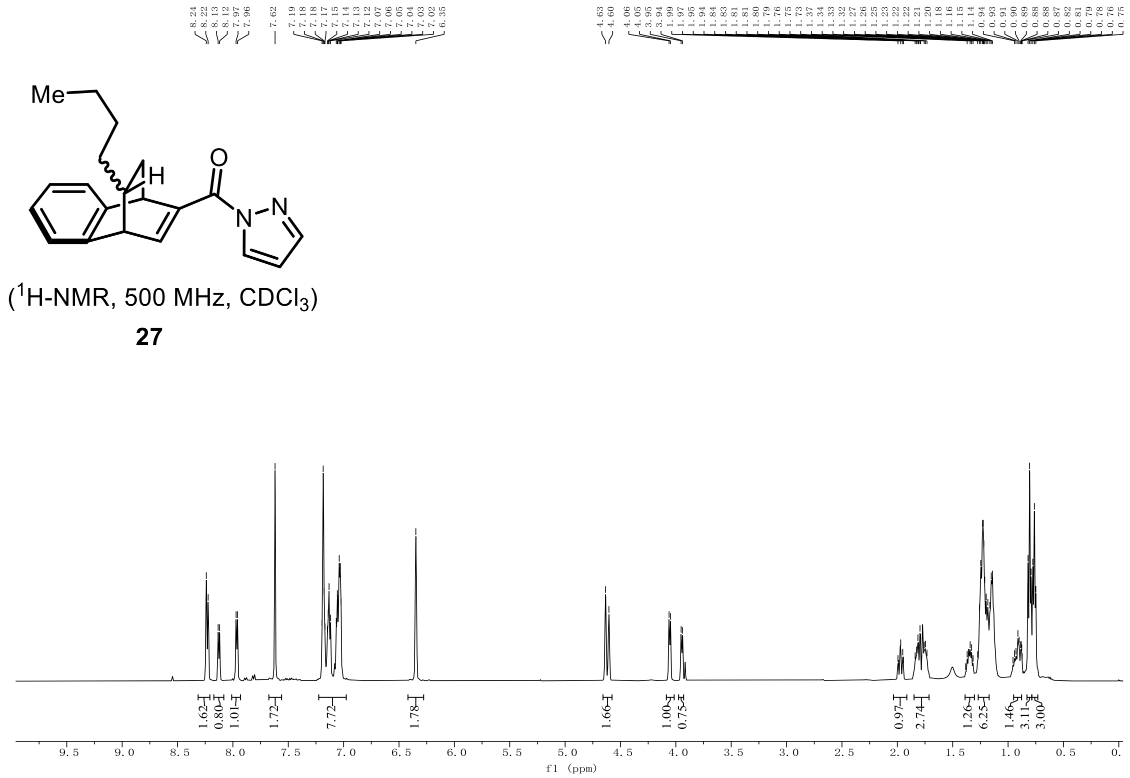

^{1}H-NMR Spectra of compound **27.**

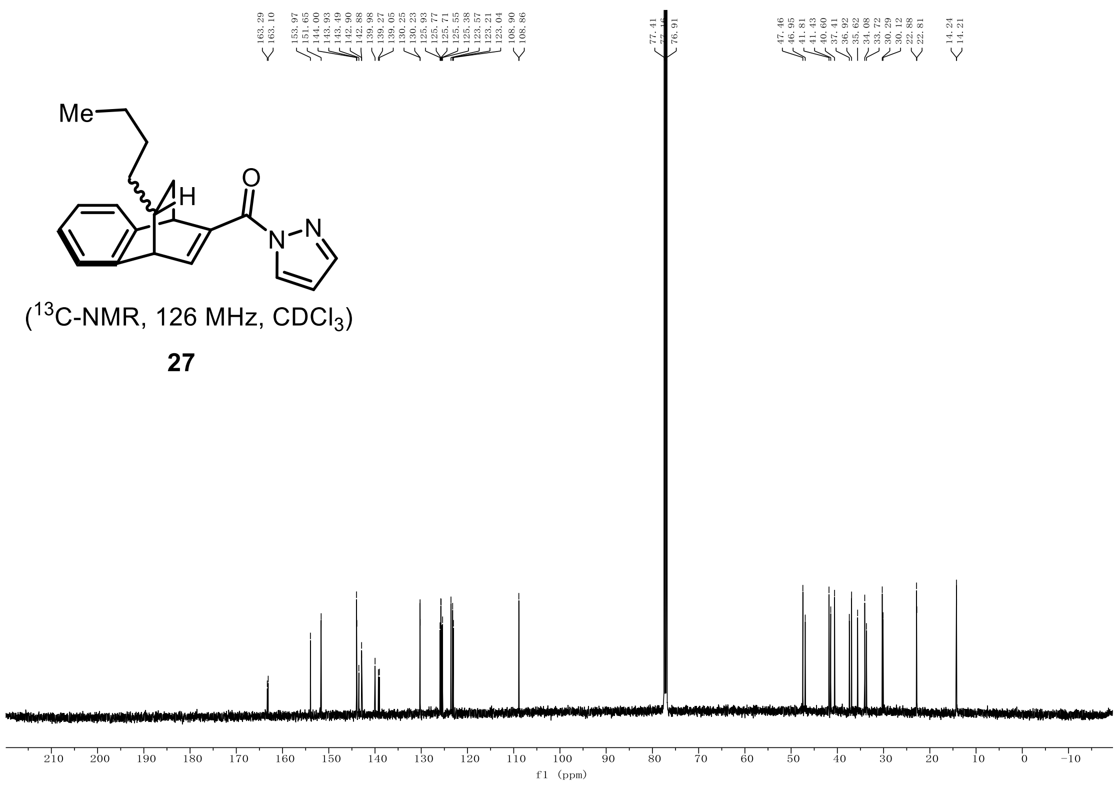

^{13}C-NMR Spectra of compound **27.**

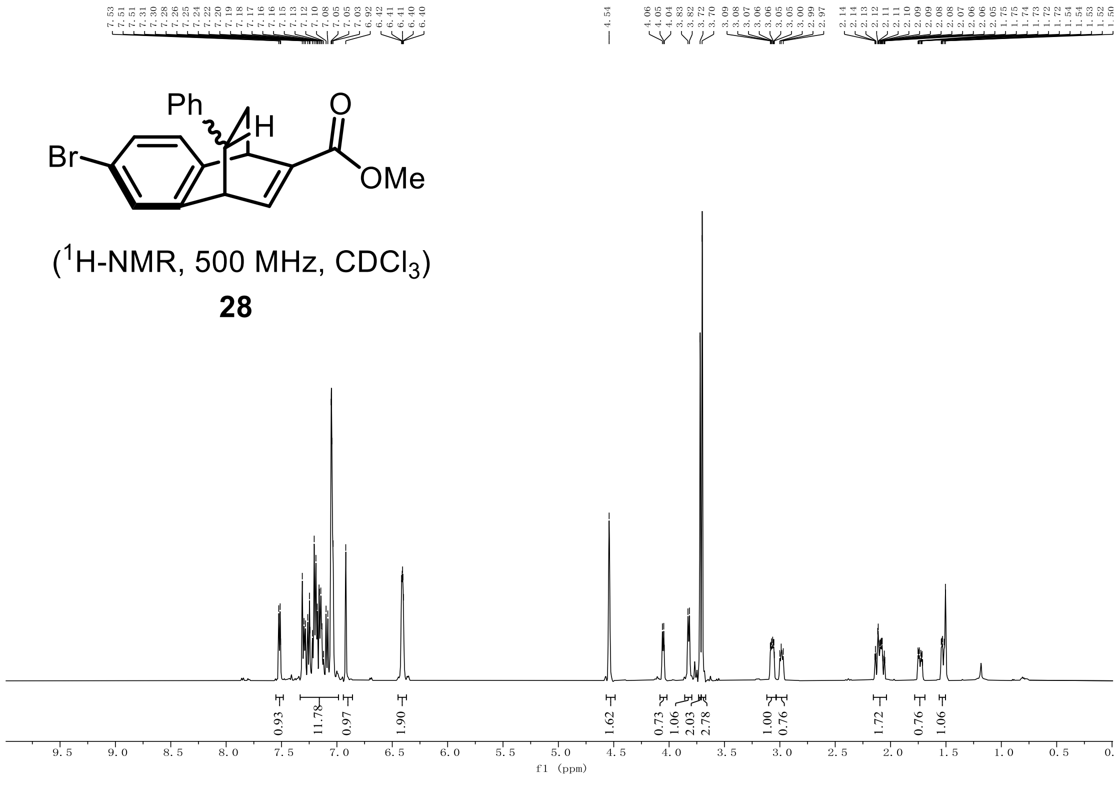

^{1}H-NMR Spectra of compound **28.**

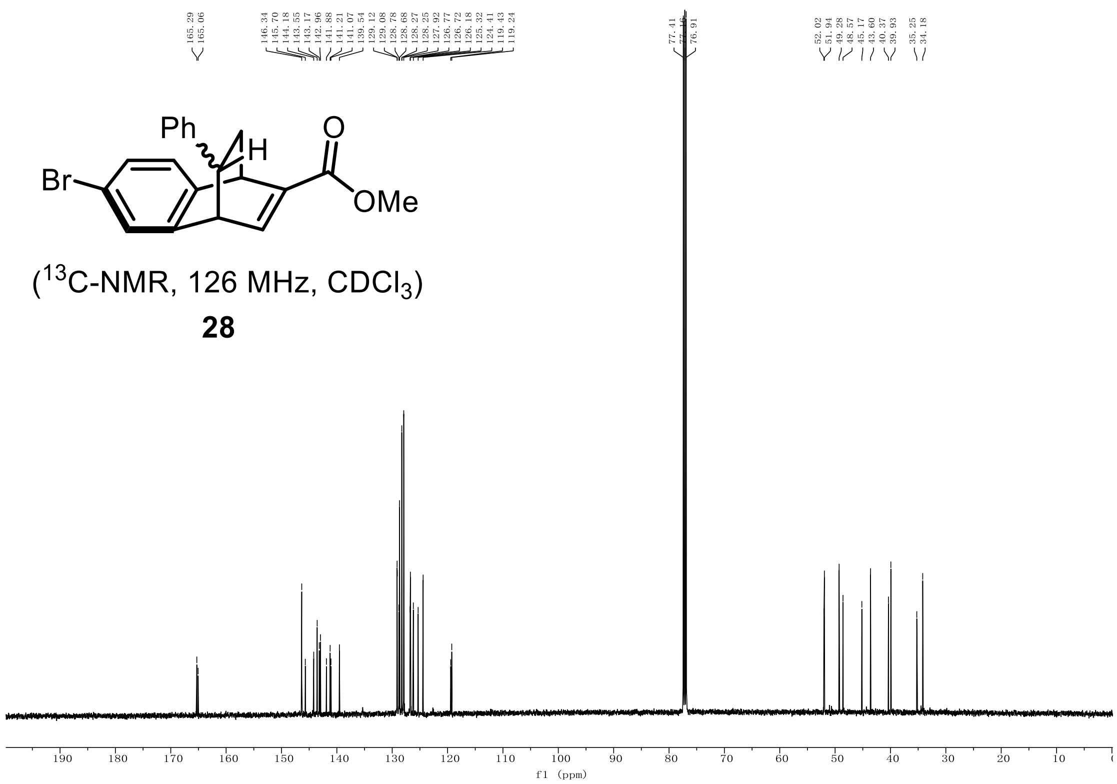

^{13}C-NMR Spectra of compound **28.**

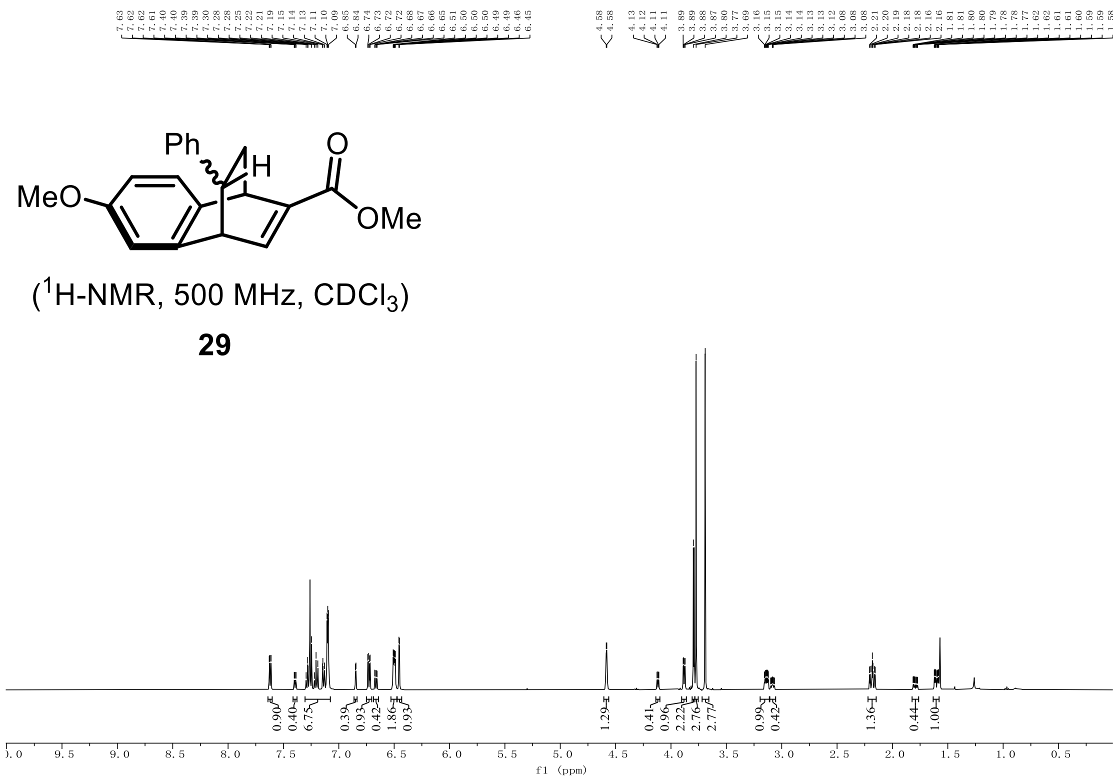

^{1}H-NMR Spectra of compound **29.**

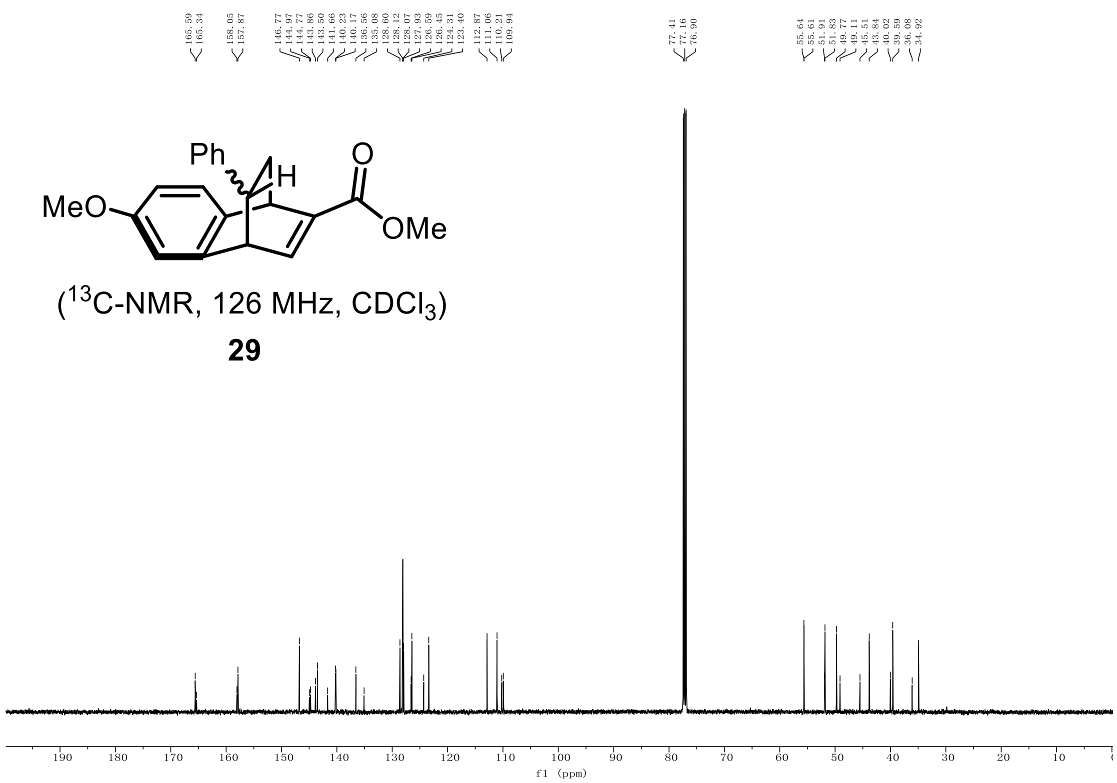

^{13}C-NMR Spectra of compound **29.**

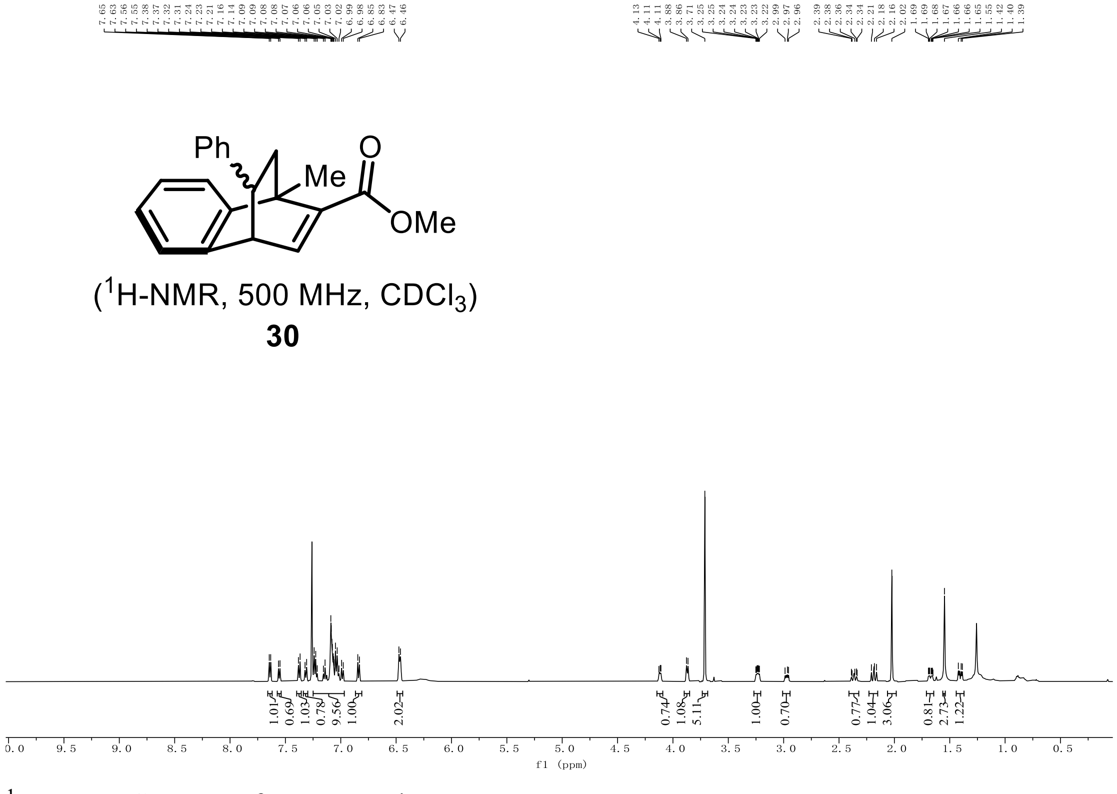

^{1}H-NMR Spectra of compound **30.**

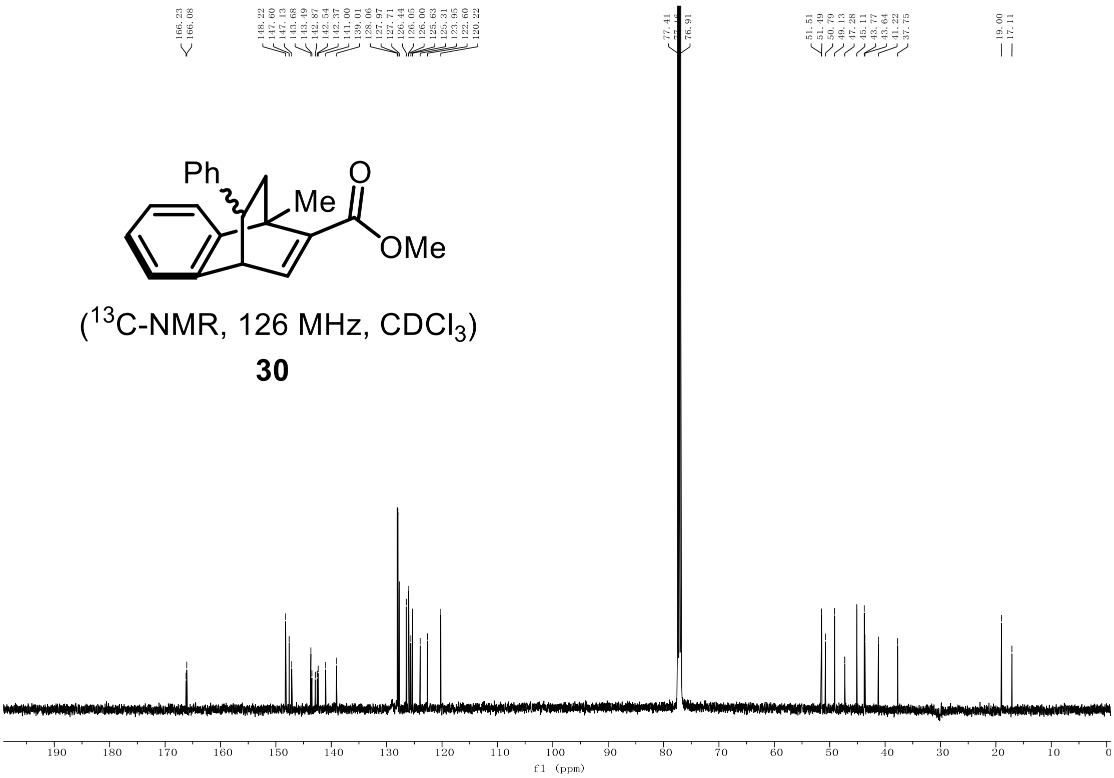

^{13}C-NMR Spectra of compound **30.**

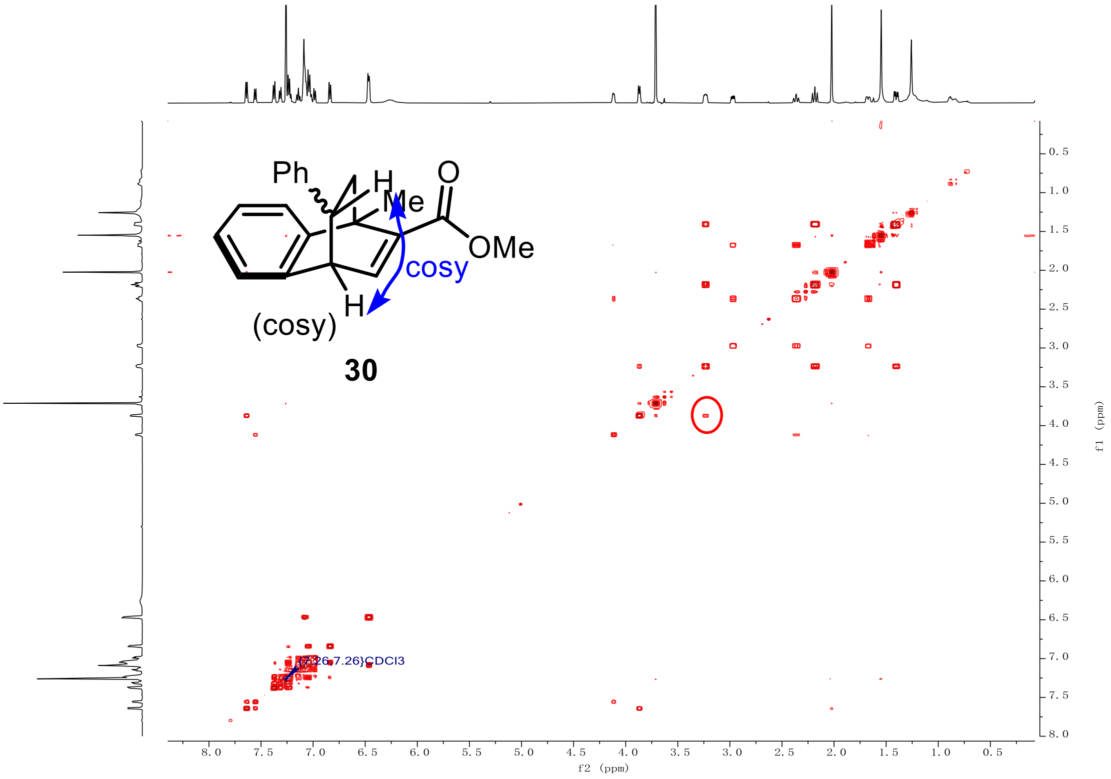

Cosy Spectra of compound **30.**

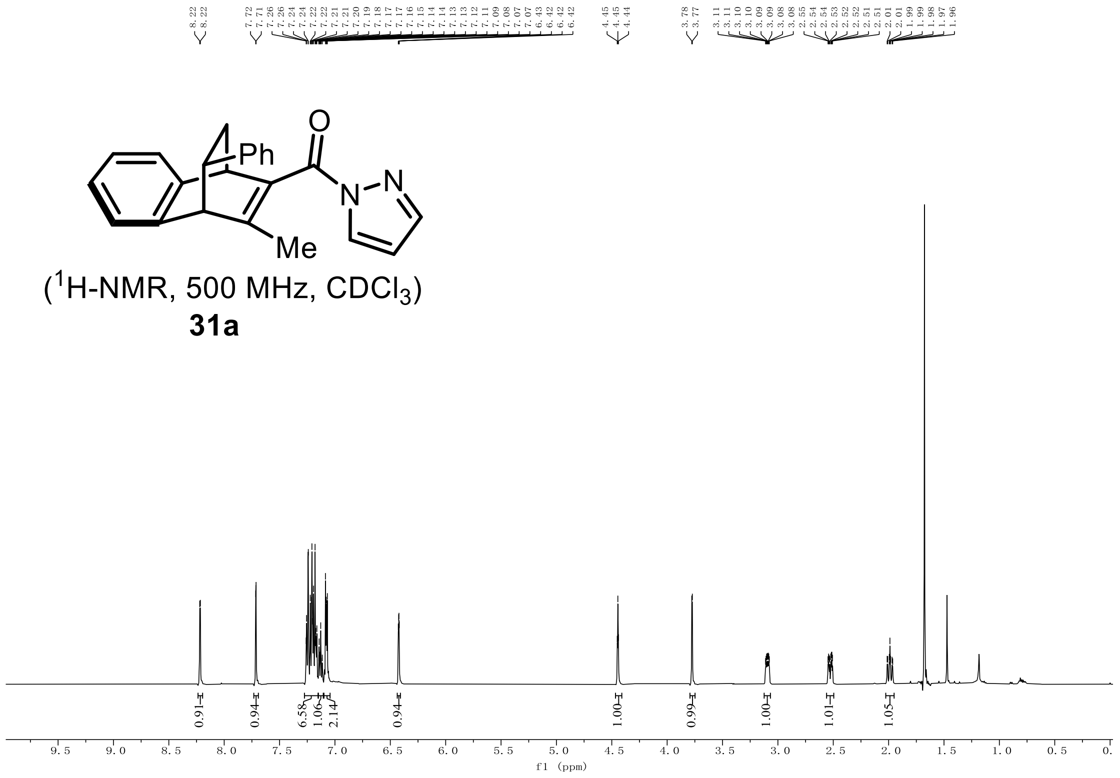

^{1}H-NMR Spectra of compound **31a.**

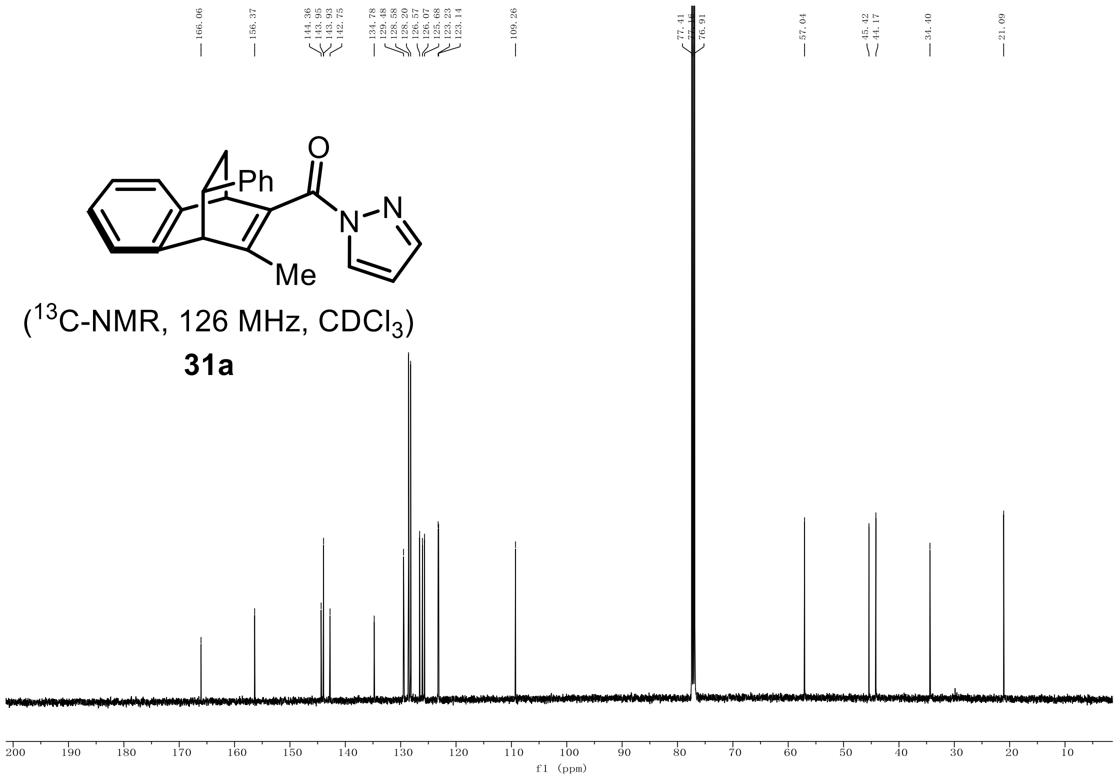

^{13}C-NMR Spectra of compound **31a.**

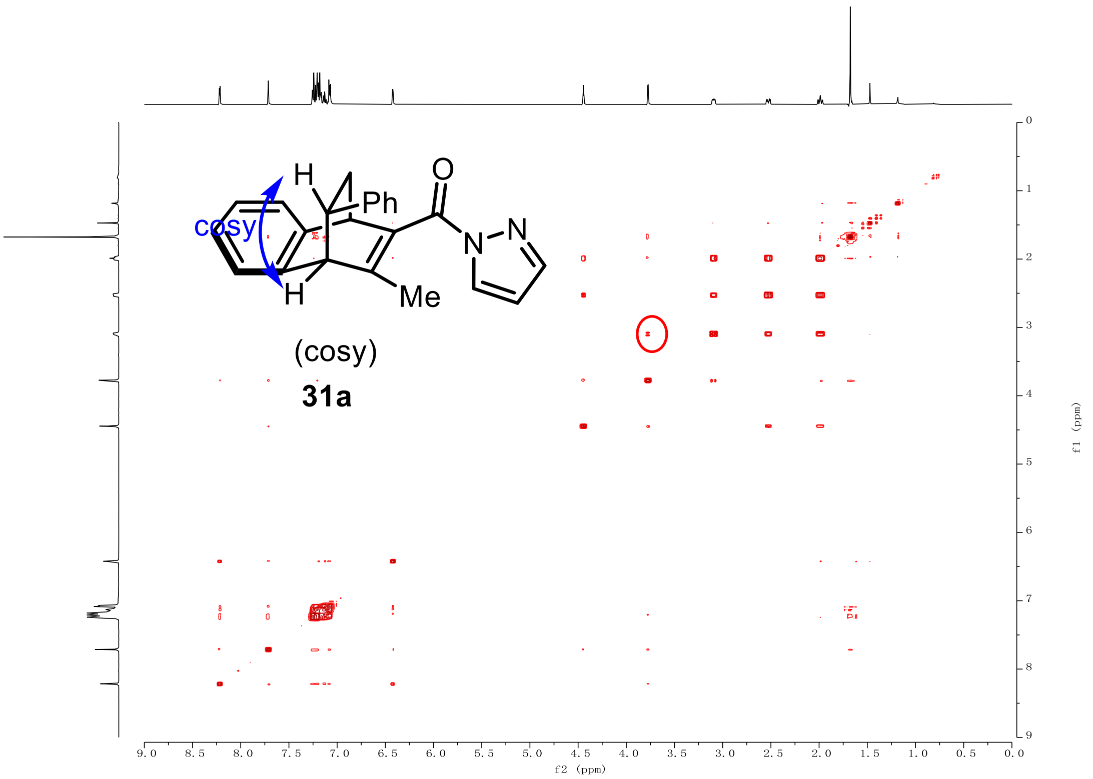

Cosy Spectra of compound **31a.**

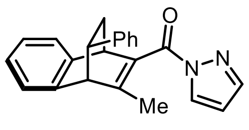

(^{1}H-NMR, 500 MHz, CDCl$_3$)
31a
minor

and

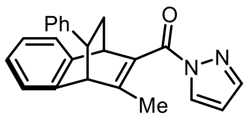

(^{1}H-NMR, 500 MHz, CDCl$_3$)
31b
major

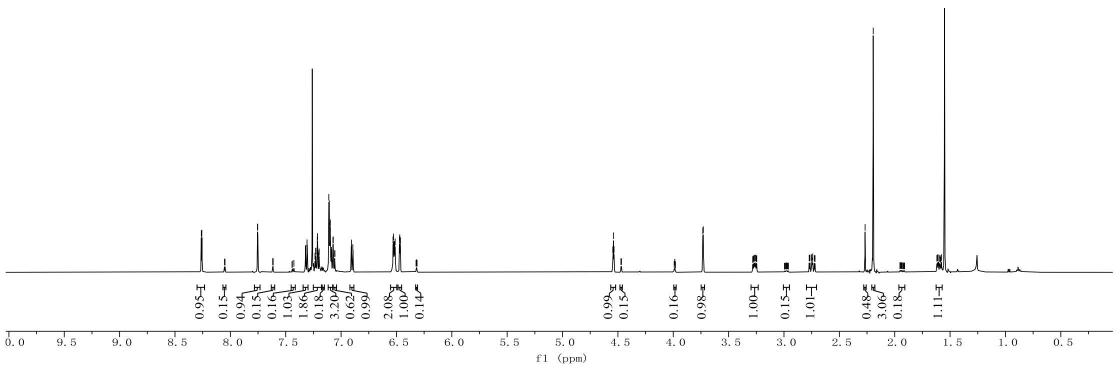

^{1}H-NMR Spectra of compound **31a, 31b.**

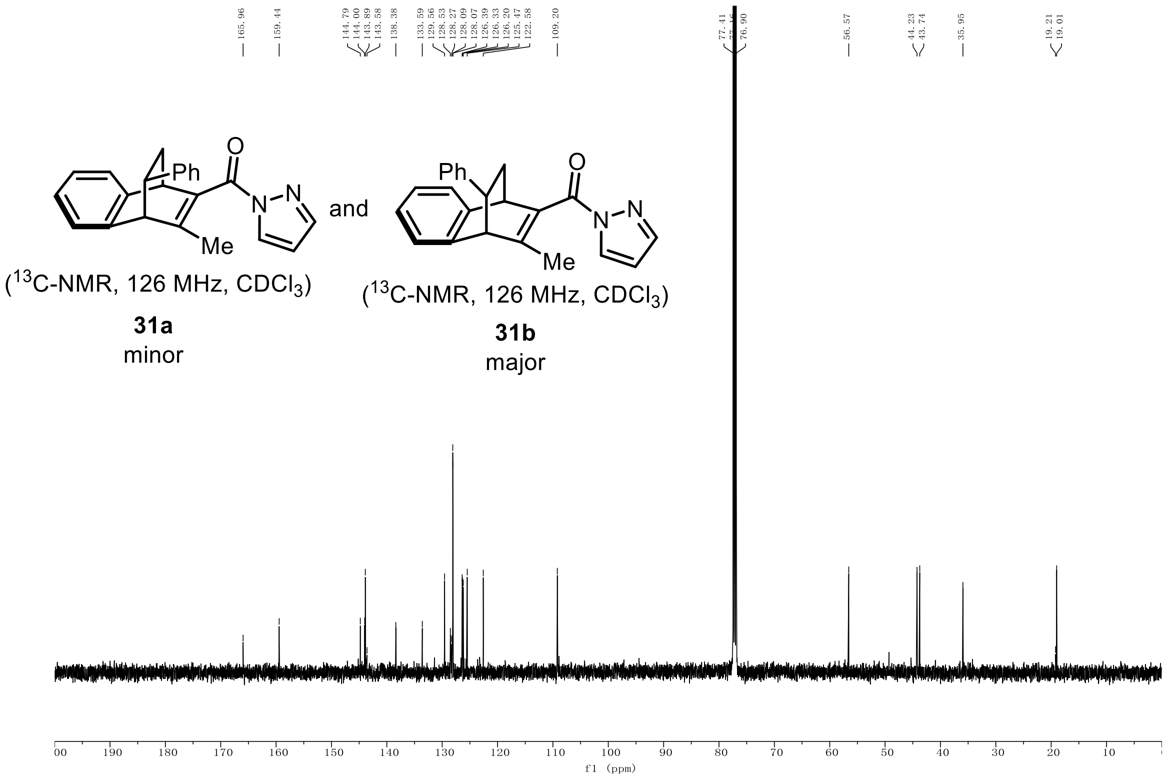

^{13}C-NMR Spectra of compound **31a, 31b.**

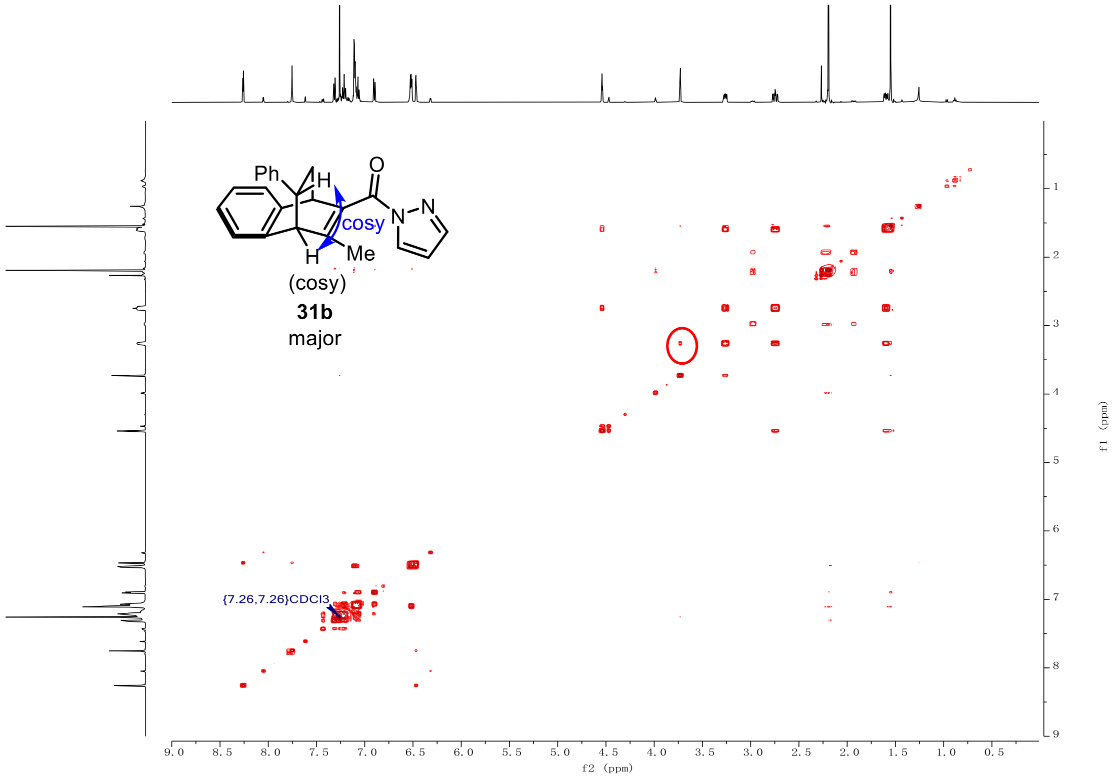

Cosy Spectra of compound **31b.**

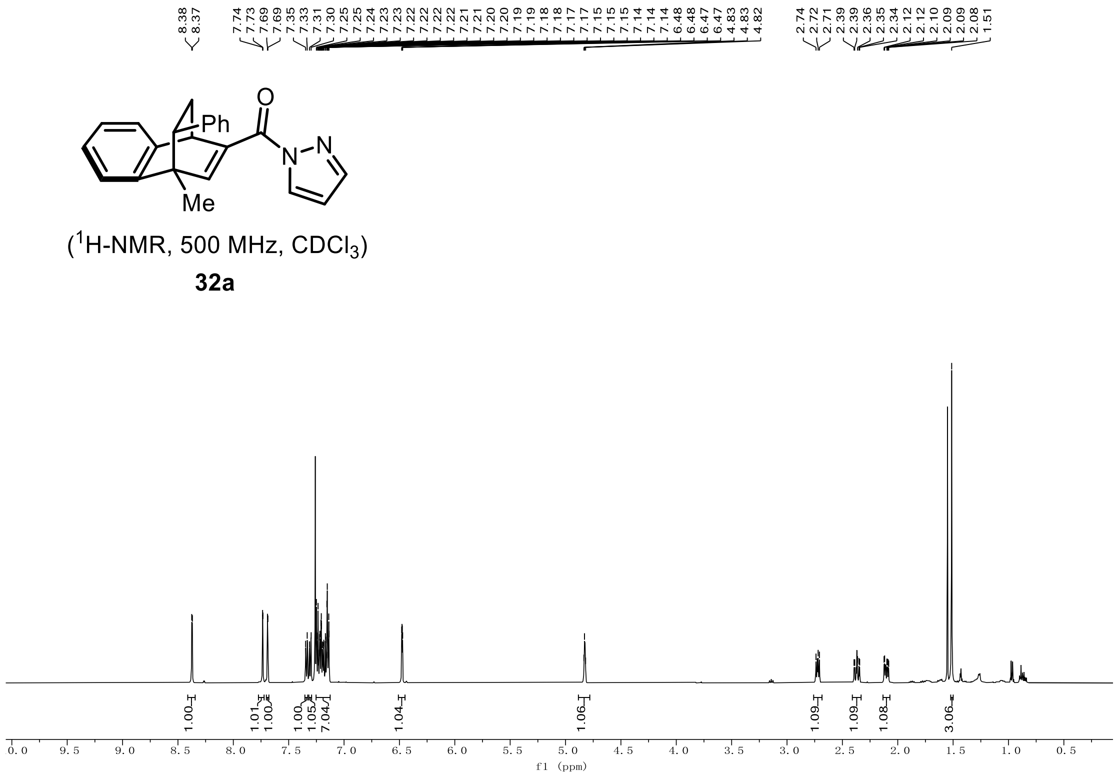

^{1}H-NMR Spectra of compound **32a.**

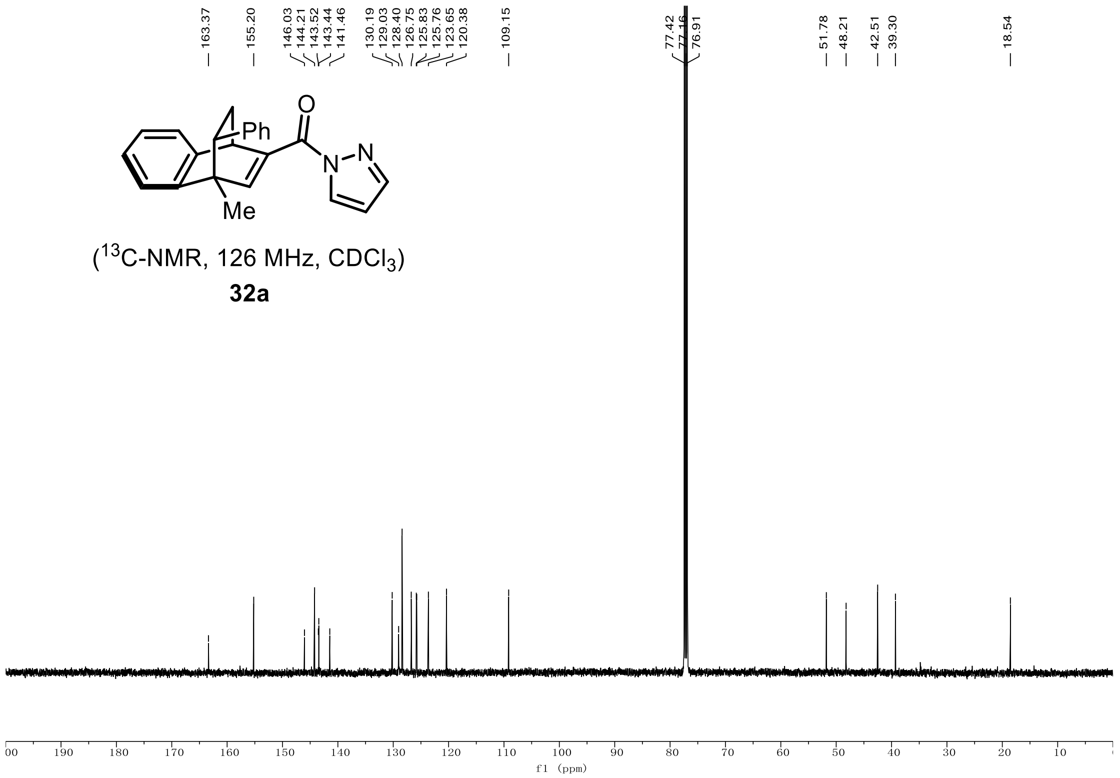

^{13}C-NMR Spectra of compound **32a.**

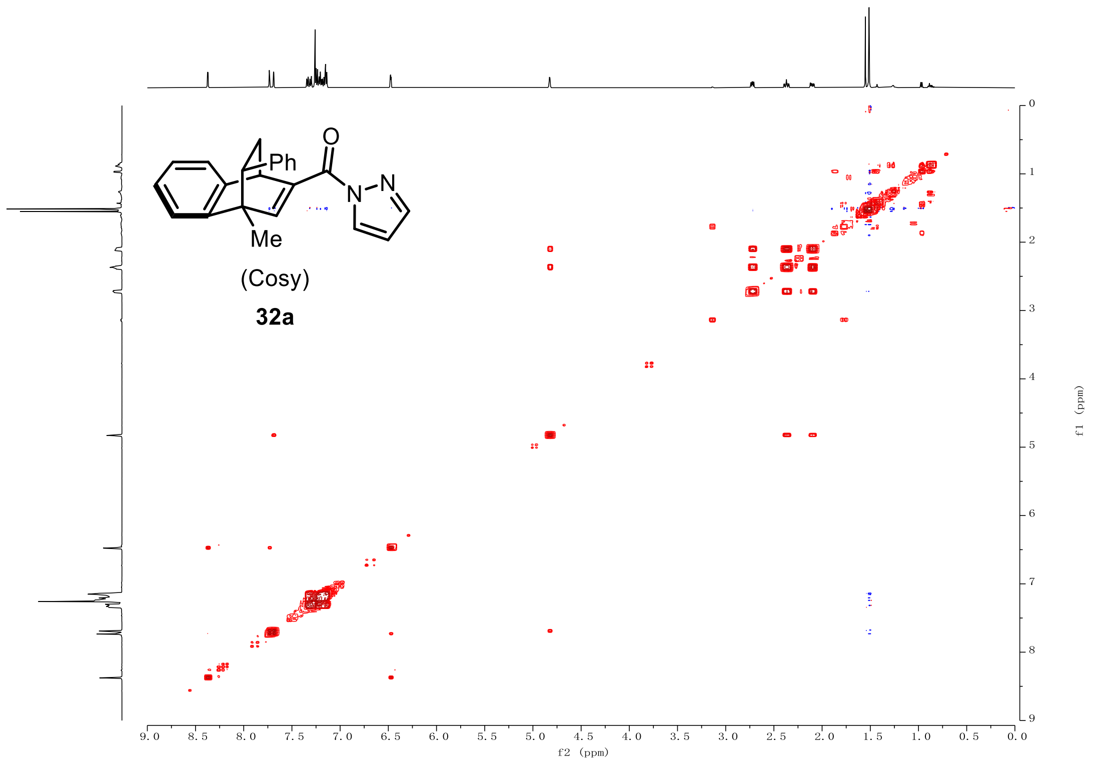

Cosy Spectra of compound **32a.**

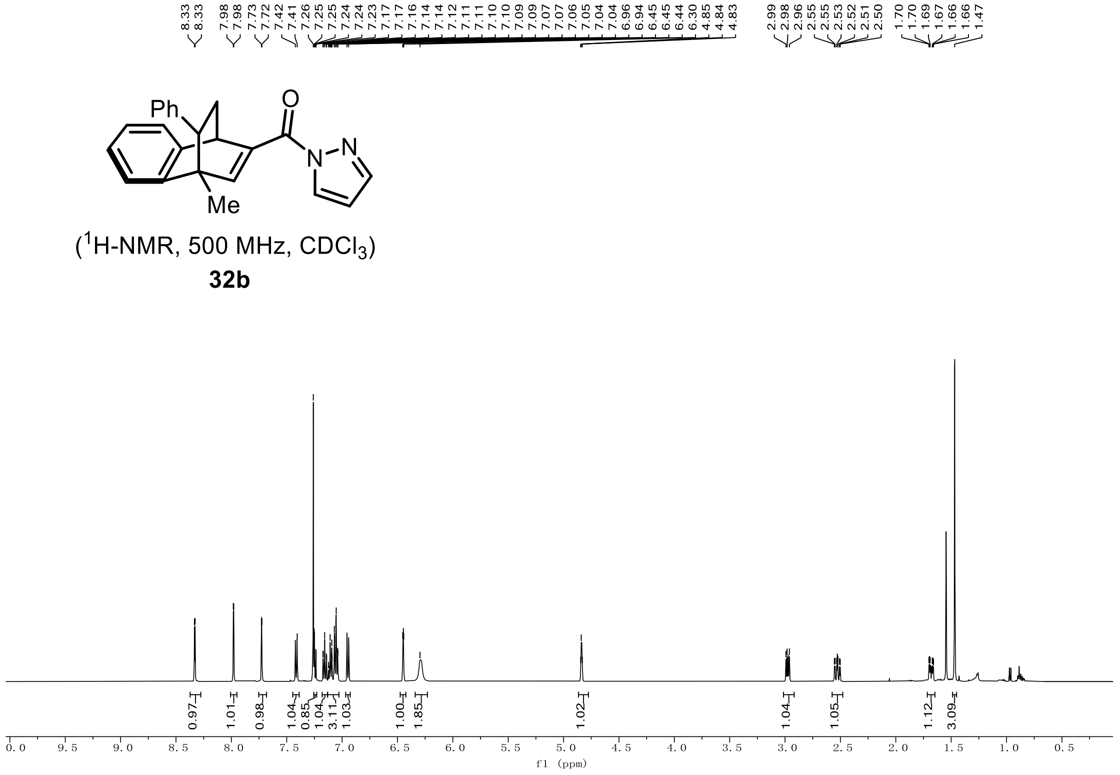

^{1}H-NMR Spectra of compound **32b.**

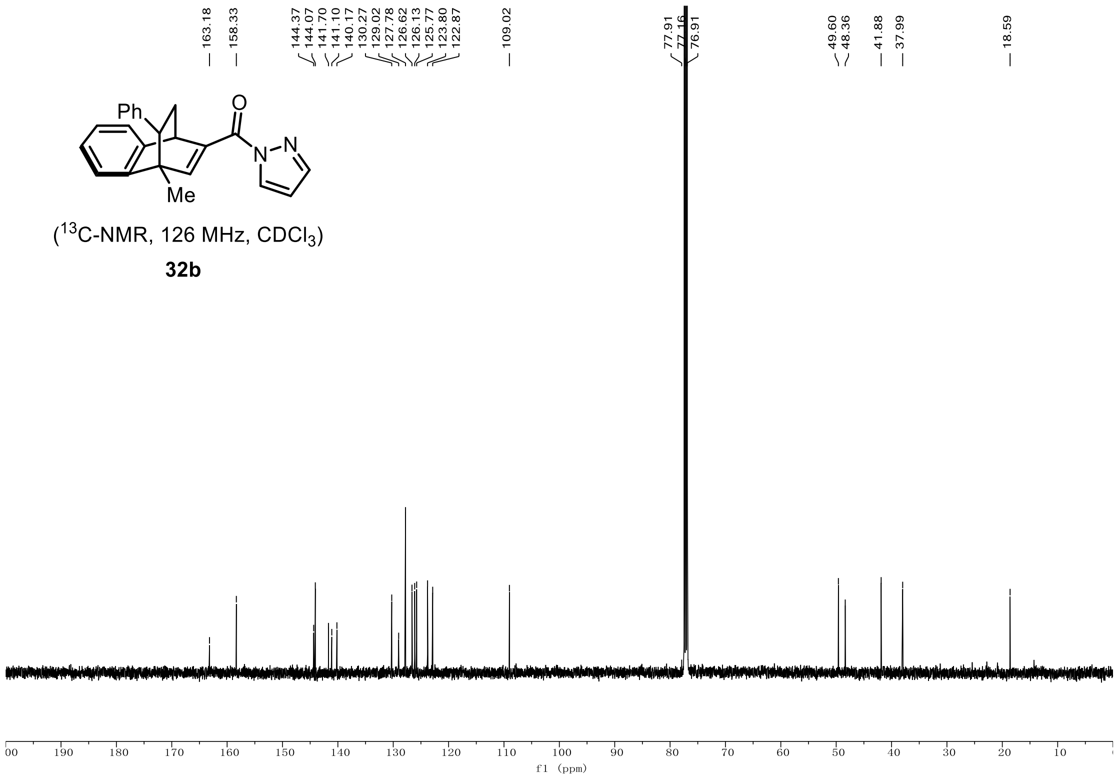

^{13}C-NMR Spectra of compound **32b.**

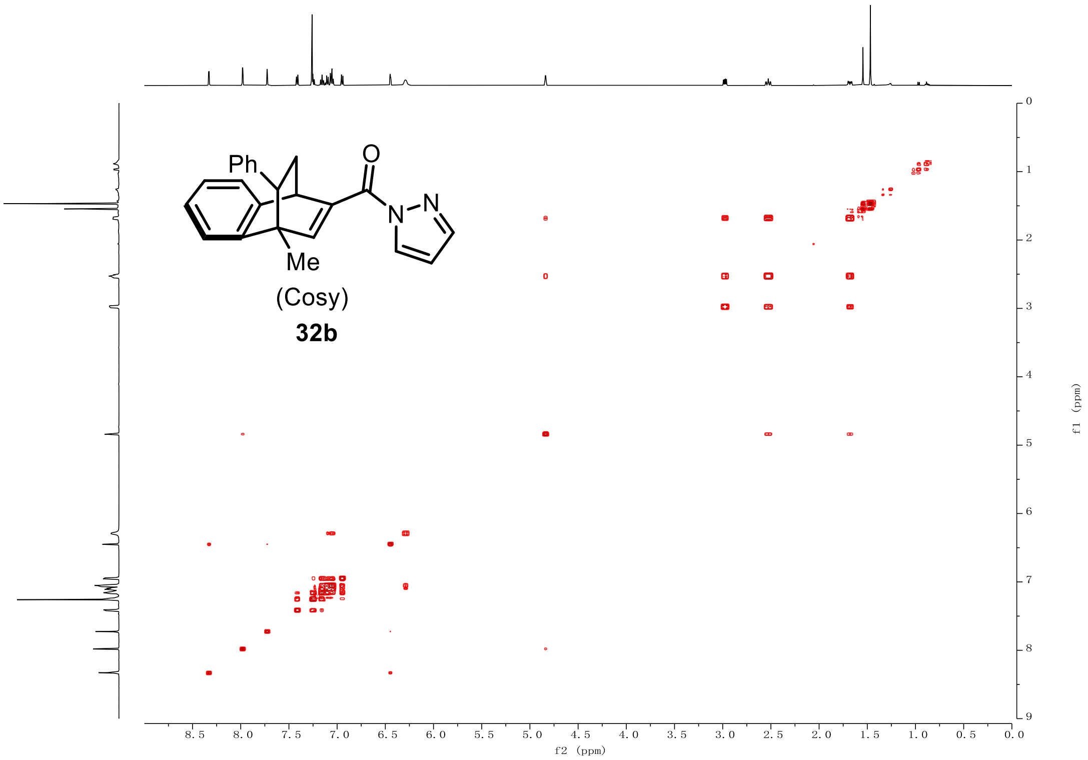

Cosy Spectra of compound **32b.**

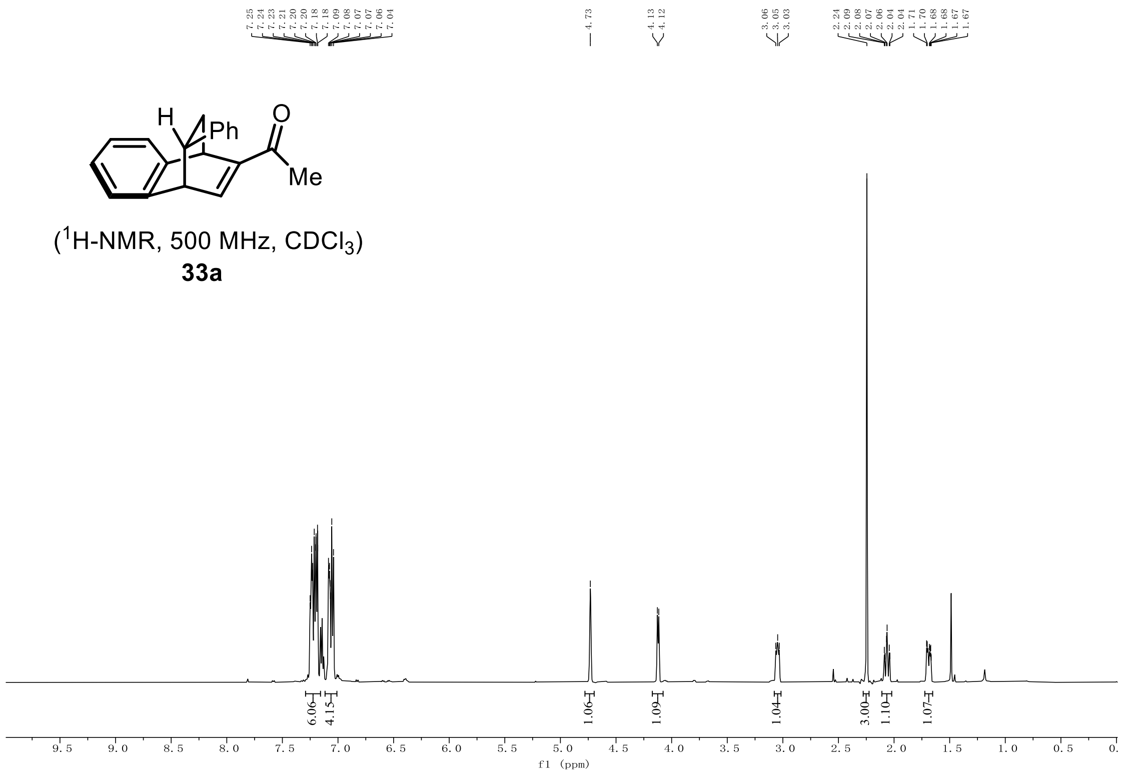

^{1}H-NMR Spectra of compound **33a.**

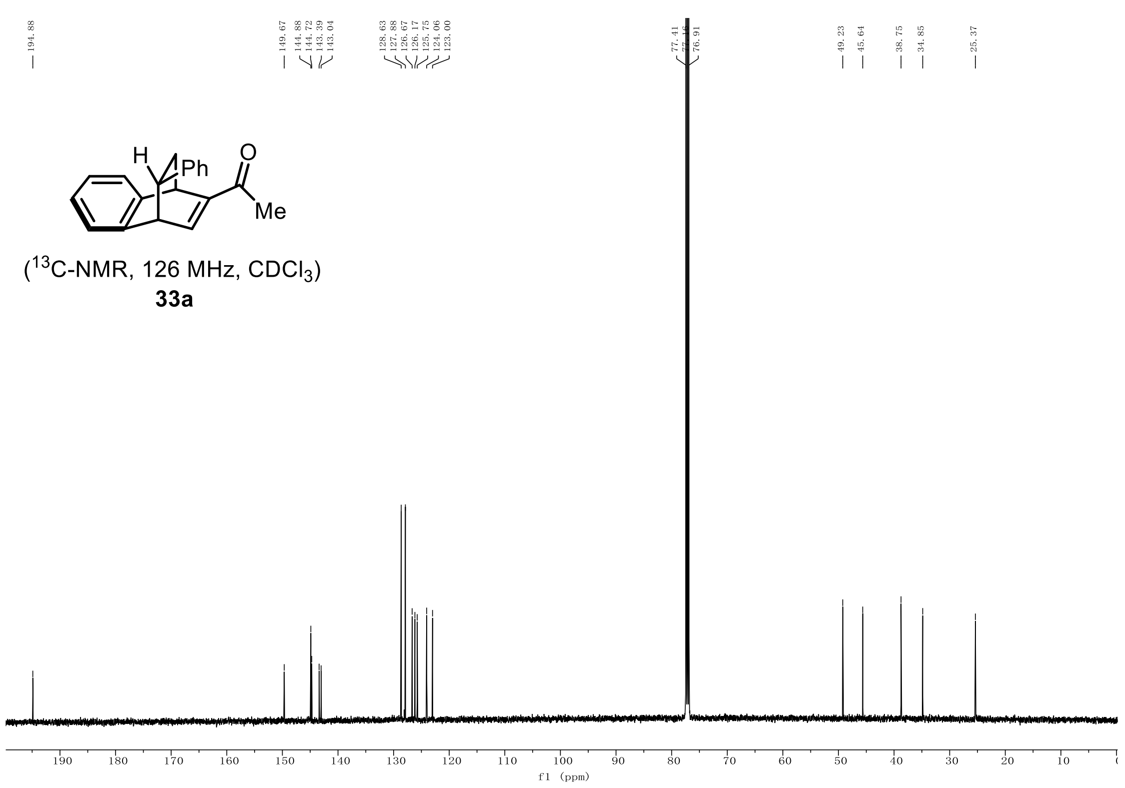

^{13}C-NMR Spectra of compound **33a.**

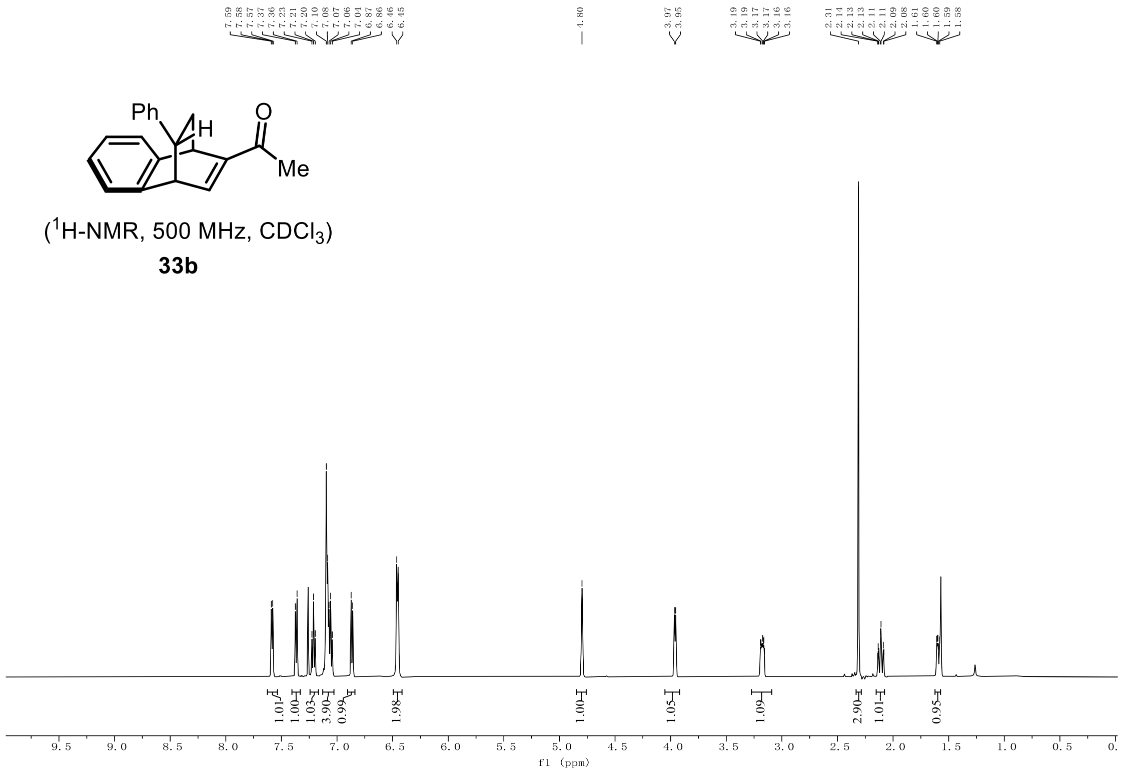

^{1}H-NMR Spectra of compound **33b.**

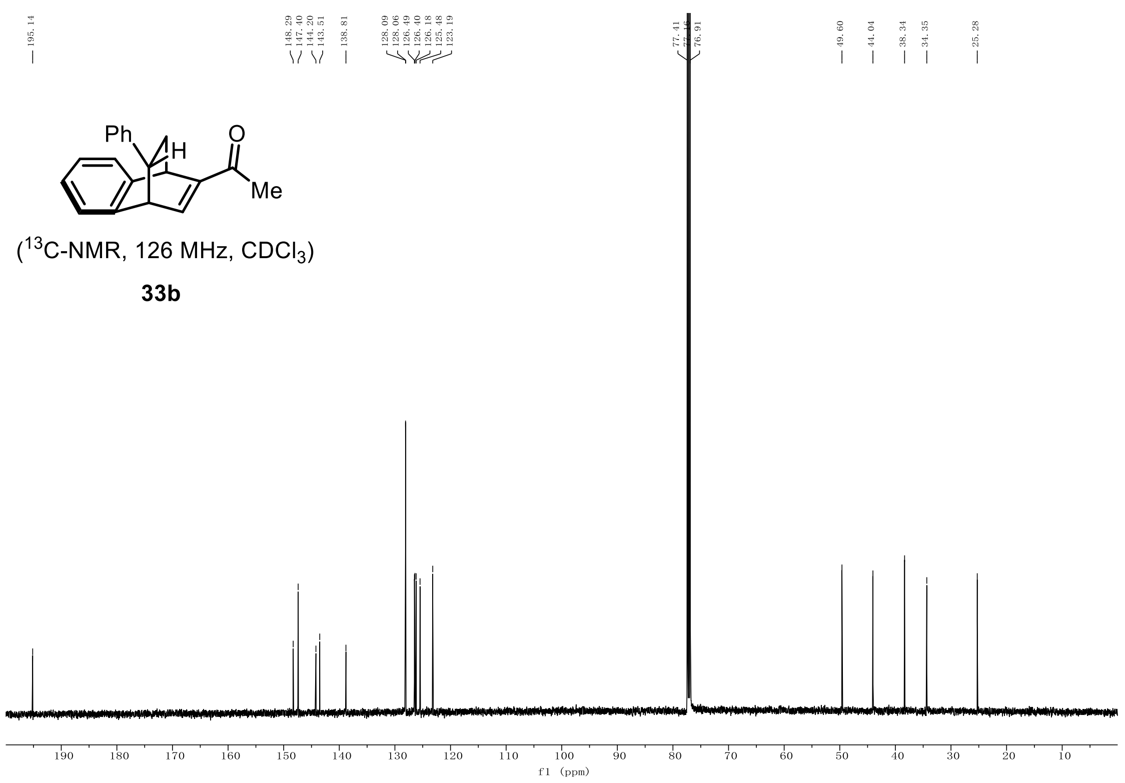

^{13}C-NMR Spectra of compound **33b.**

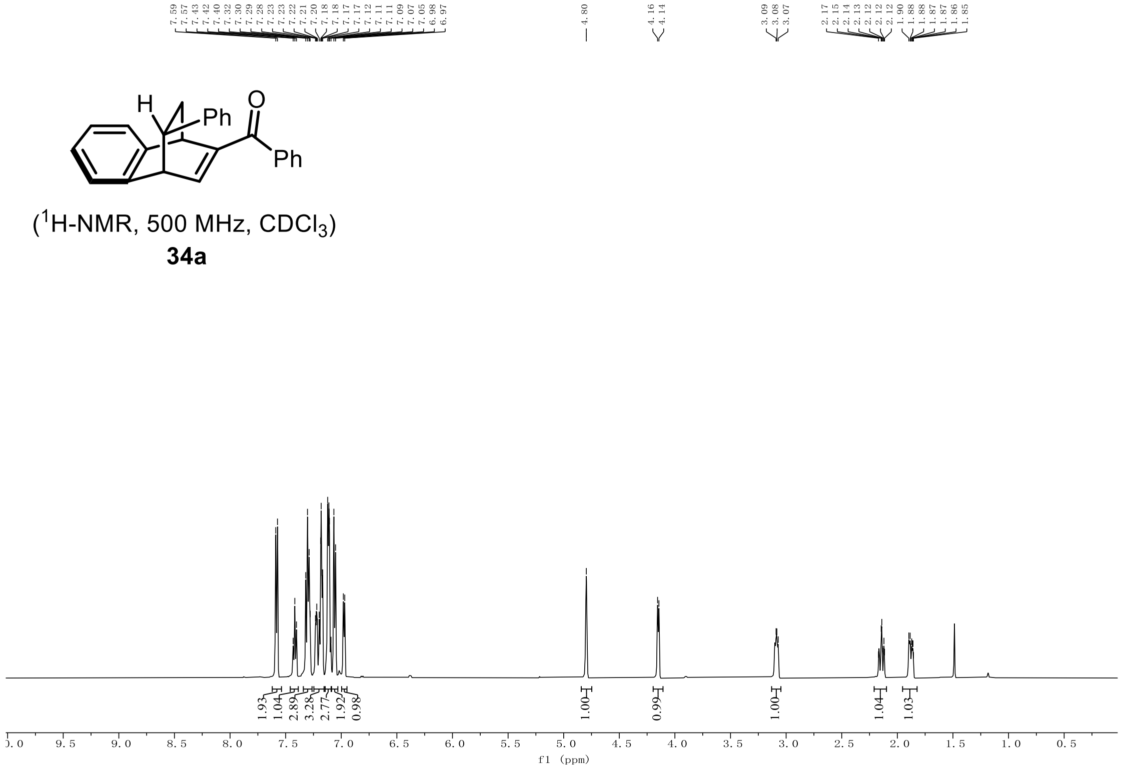

^{1}H-NMR Spectra of compound **34a.**

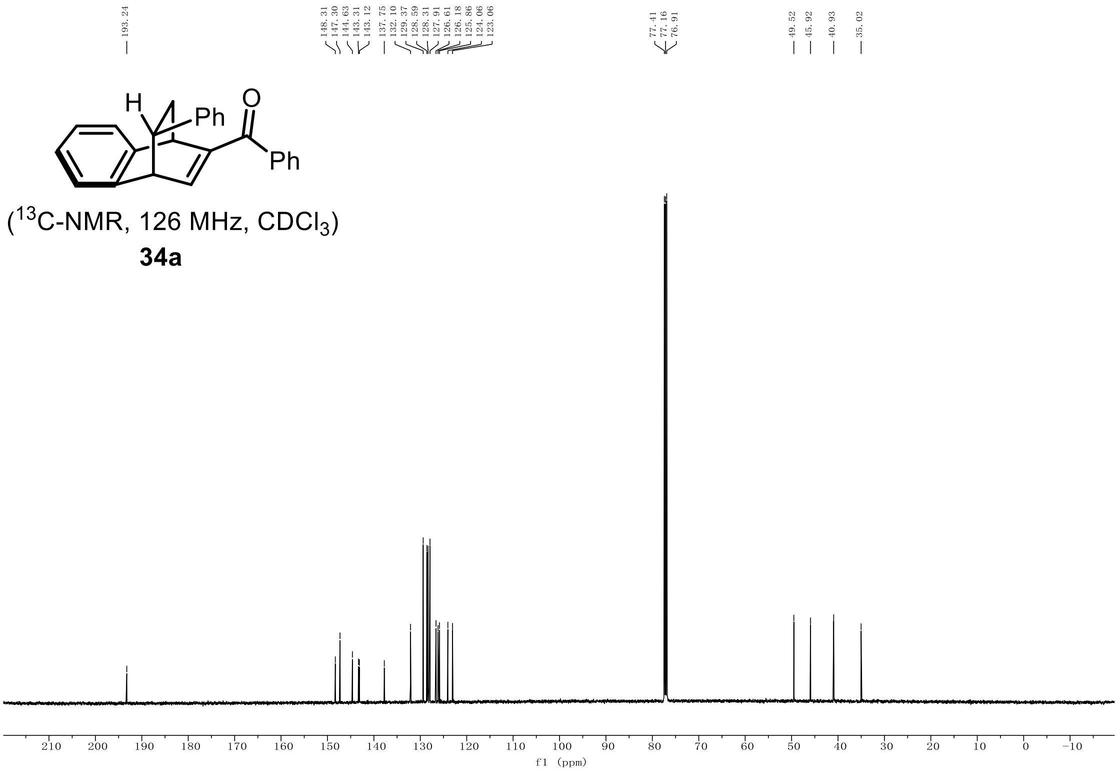

^{13}C-NMR Spectra of compound **34a.**

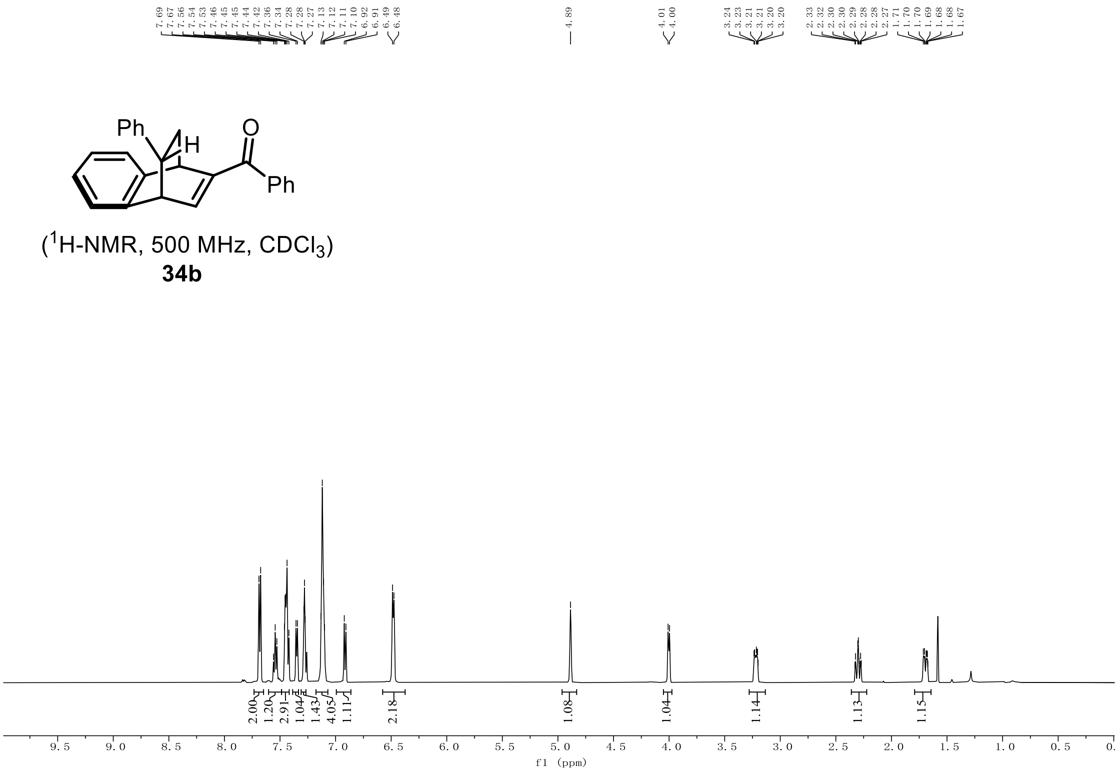

^{1}H-NMR Spectra of compound **34b.**

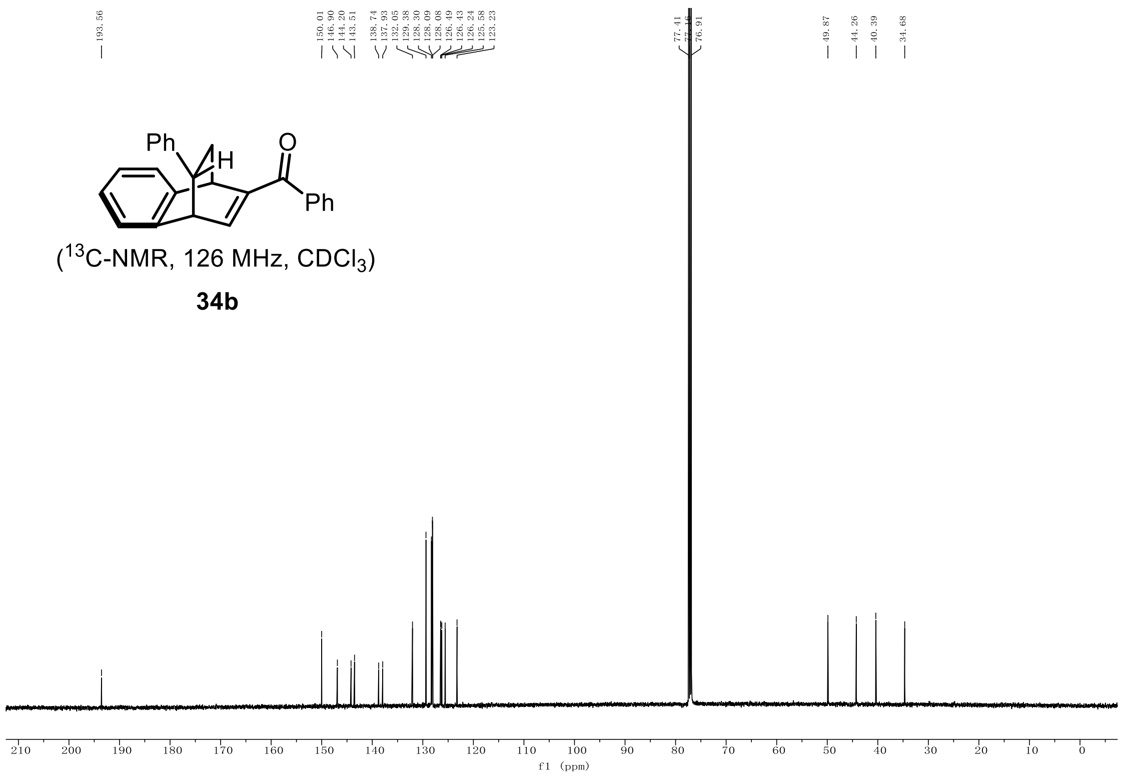

^{13}C-NMR Spectra of compound **34b.**

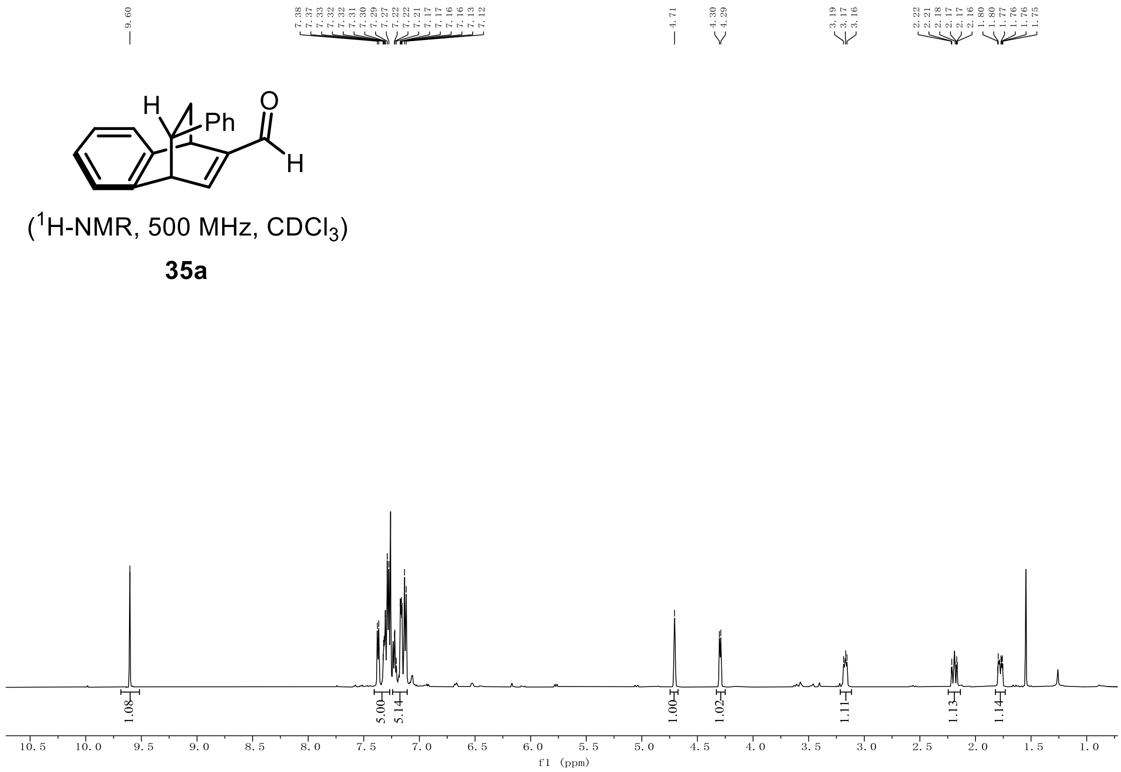

^{1}H-NMR Spectra of compound **35a.**

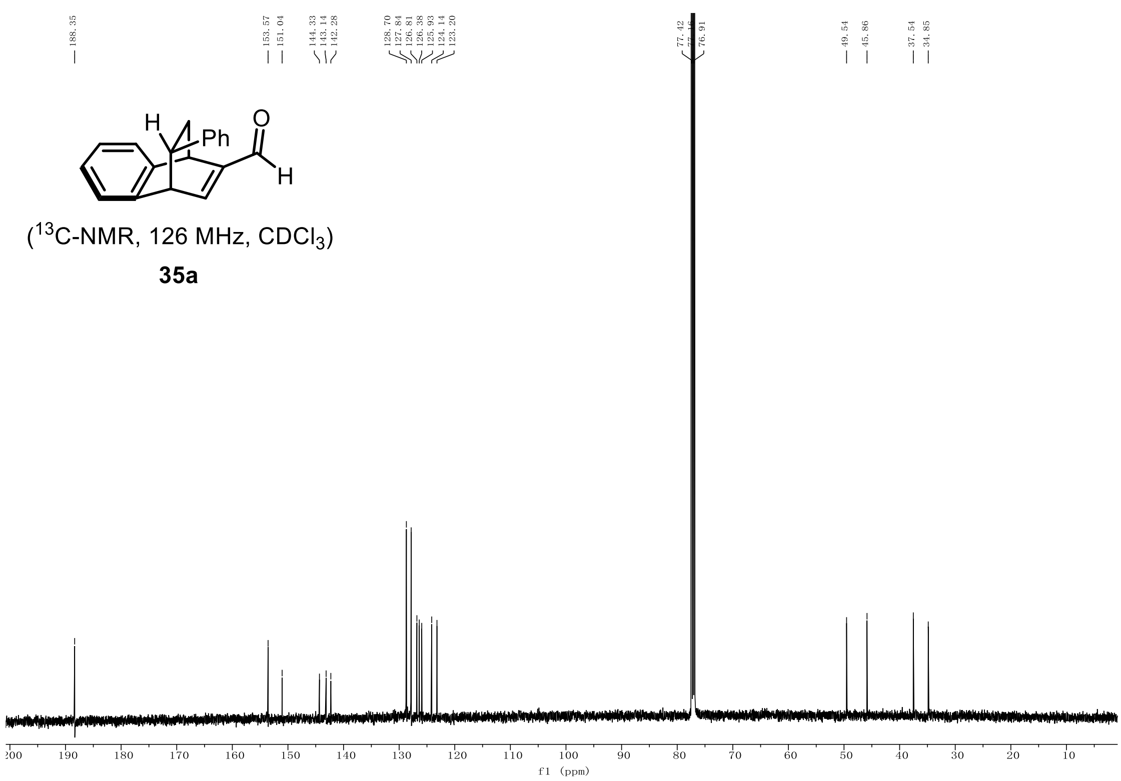

^{13}C-NMR Spectra of compound **35a.**

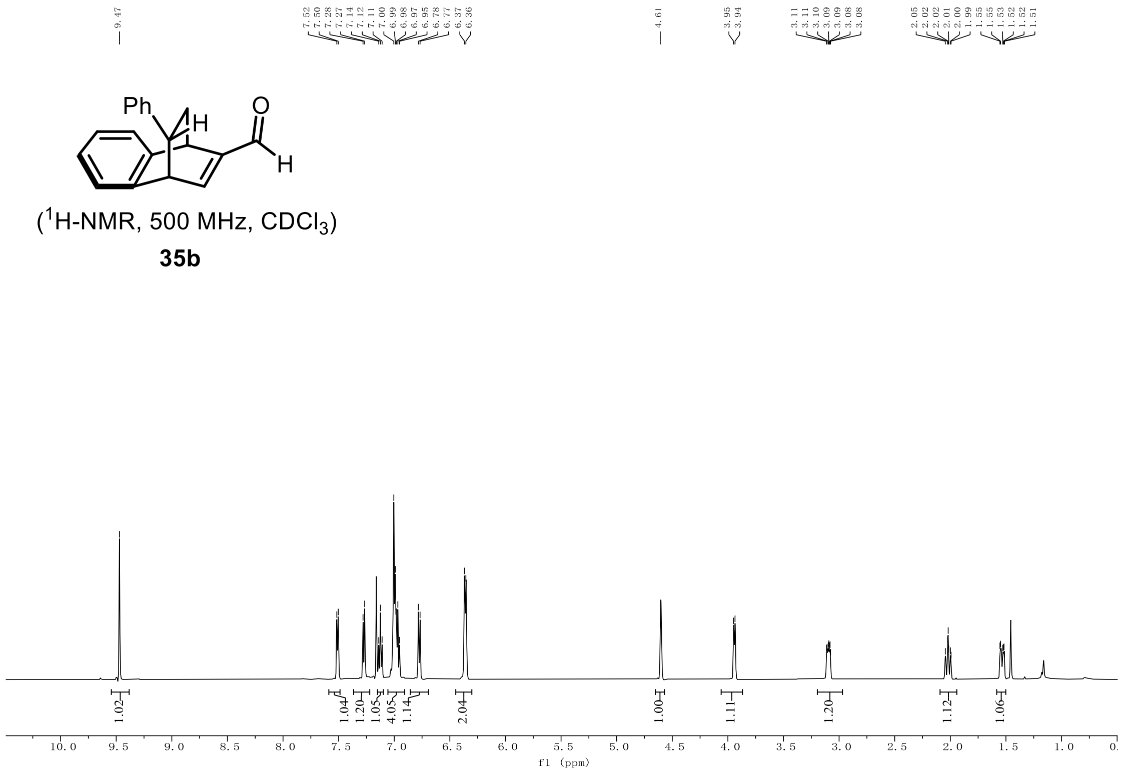

^{1}H-NMR Spectra of compound **35b.**

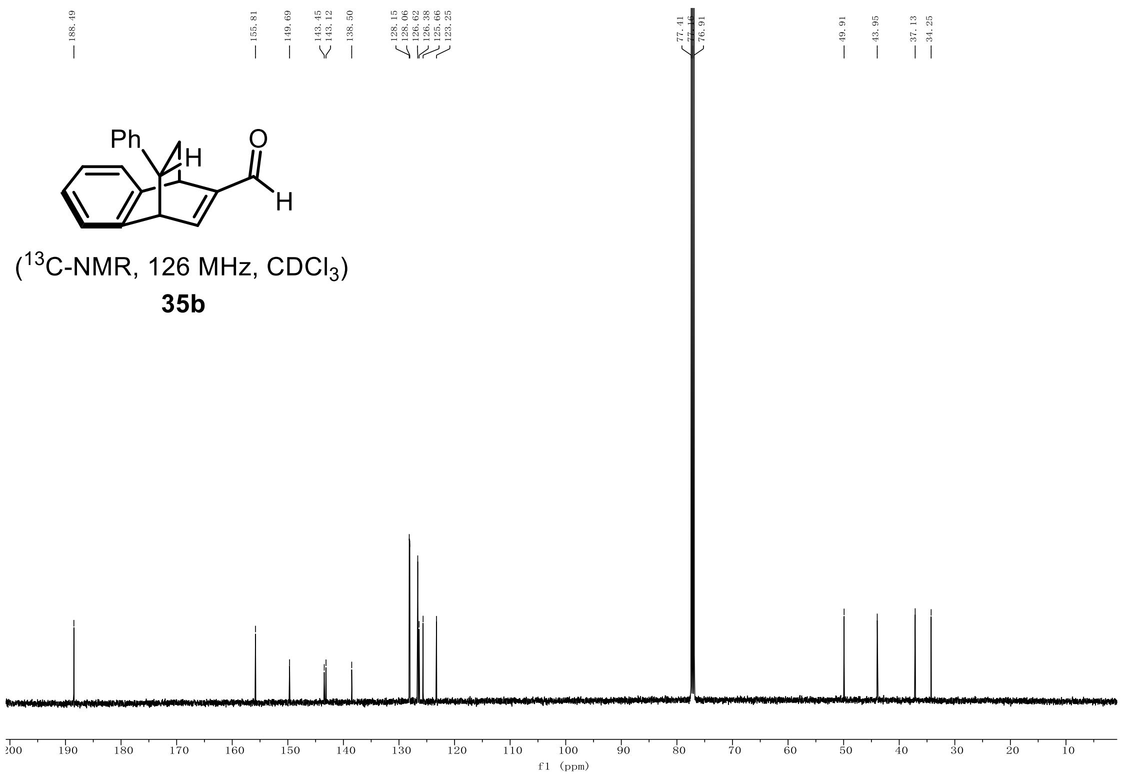

^{13}C-NMR Spectra of compound **35b.**

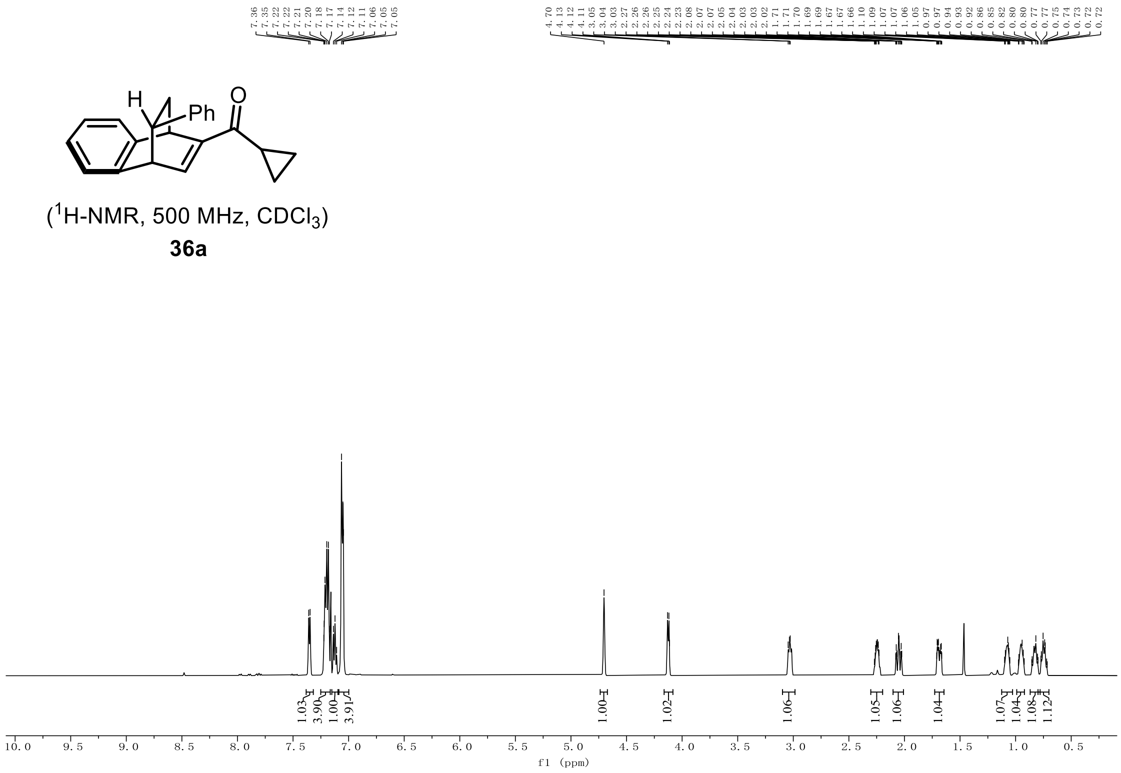

^{1}H-NMR Spectra of compound **36a.**

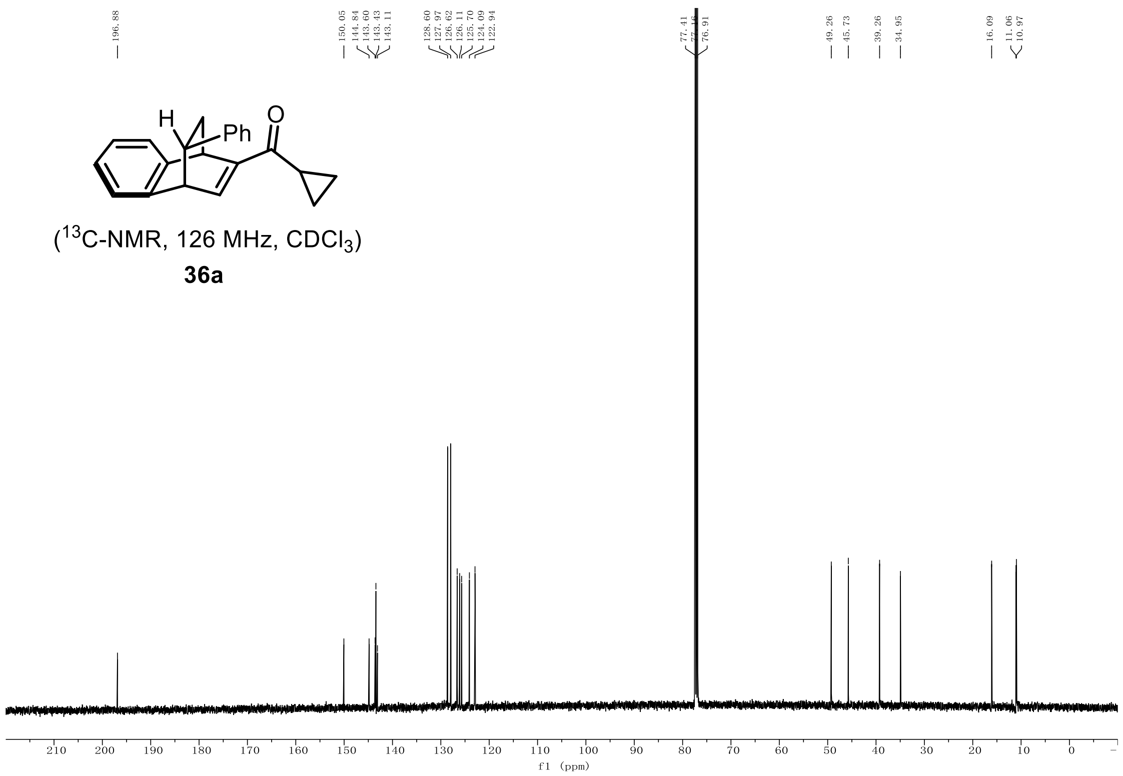

^{13}C-NMR Spectra of compound **36a.**

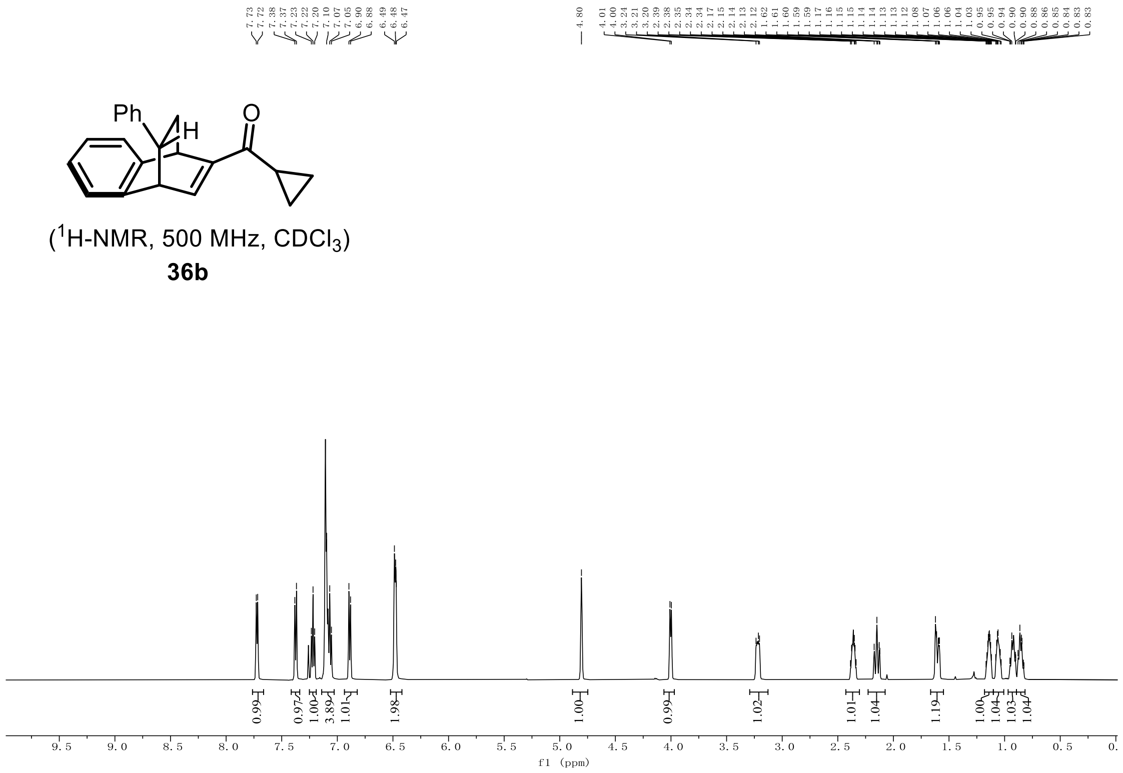

^{1}H-NMR Spectra of compound **36b.**

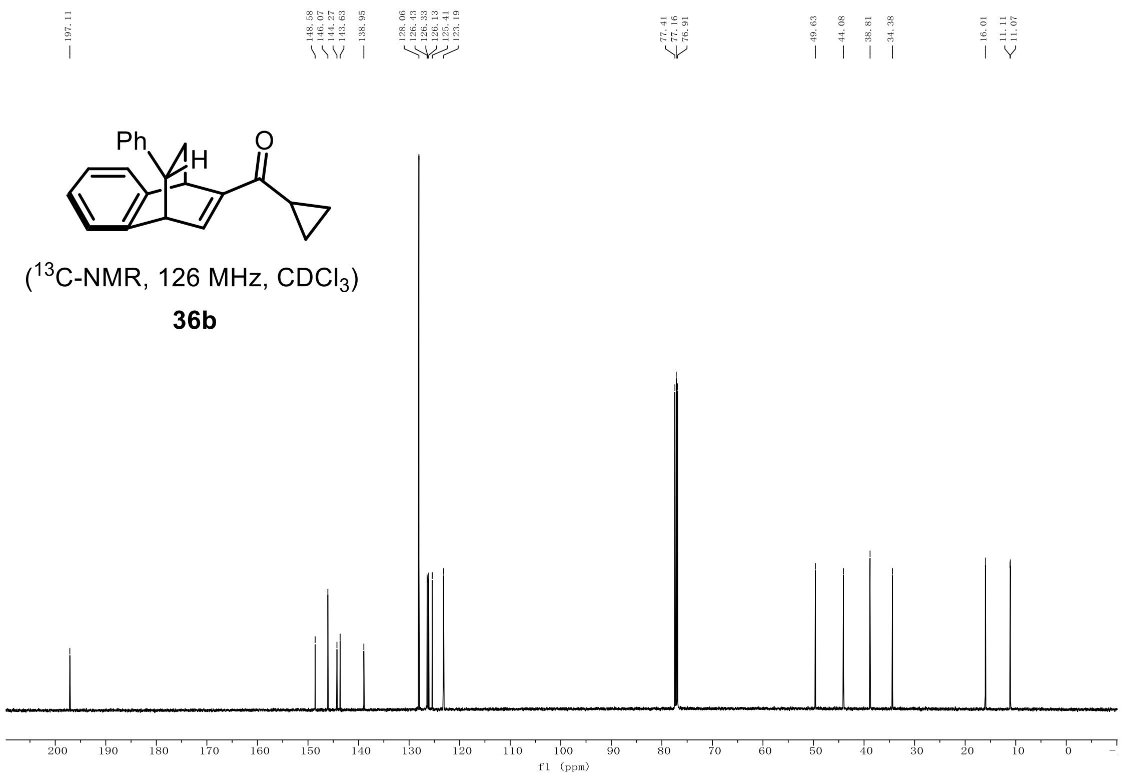

^{13}C-NMR Spectra of compound **36b.**

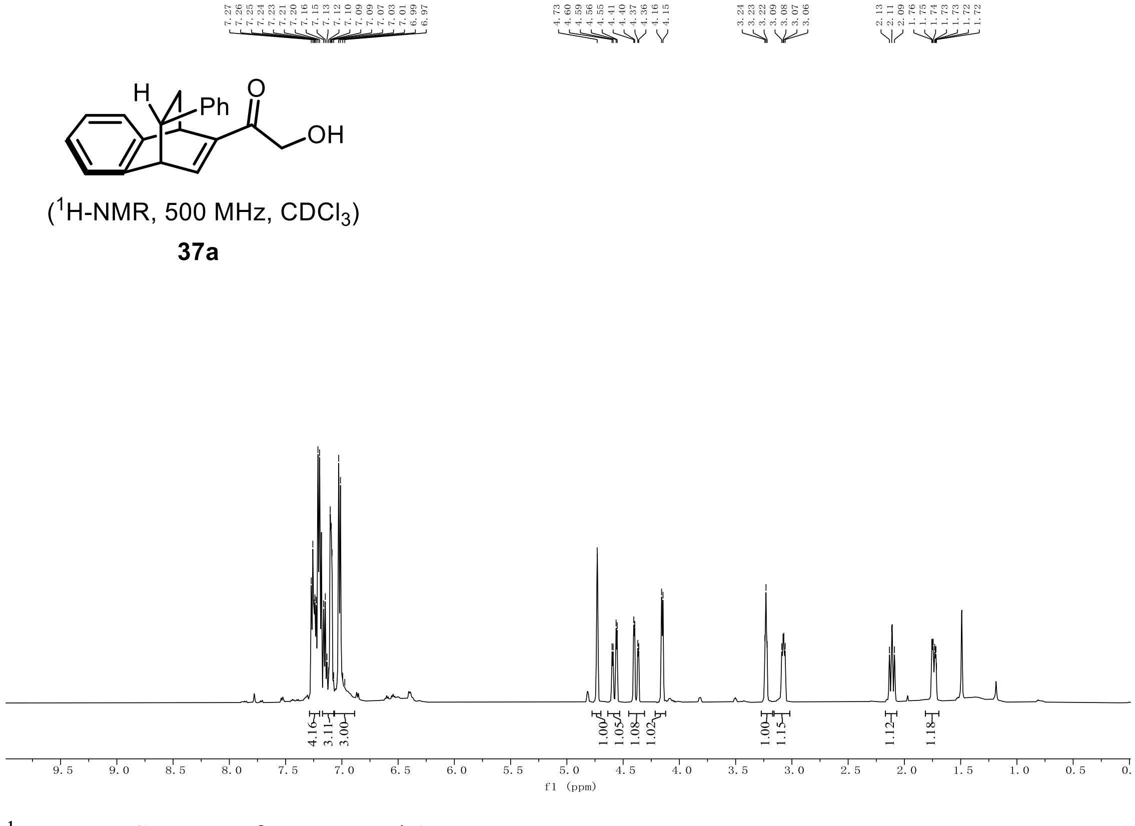

^{1}H-NMR Spectra of compound **37a.**

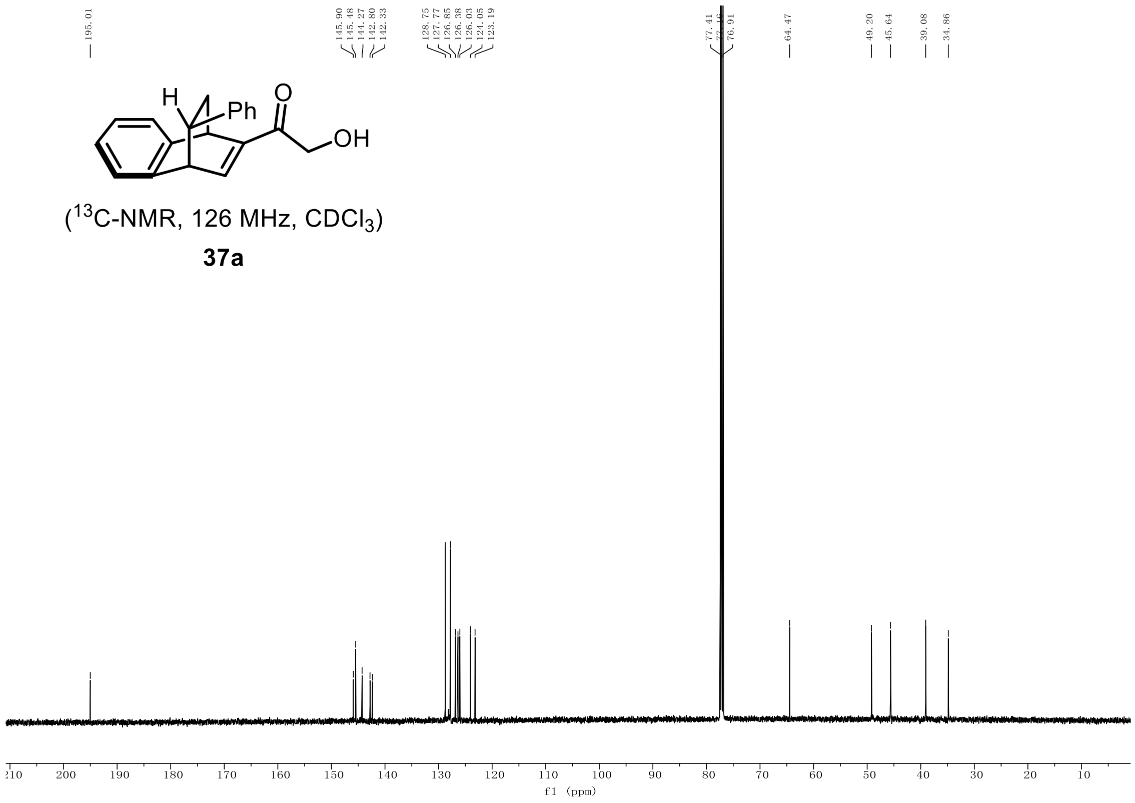

^{13}C-NMR Spectra of compound **37a.**

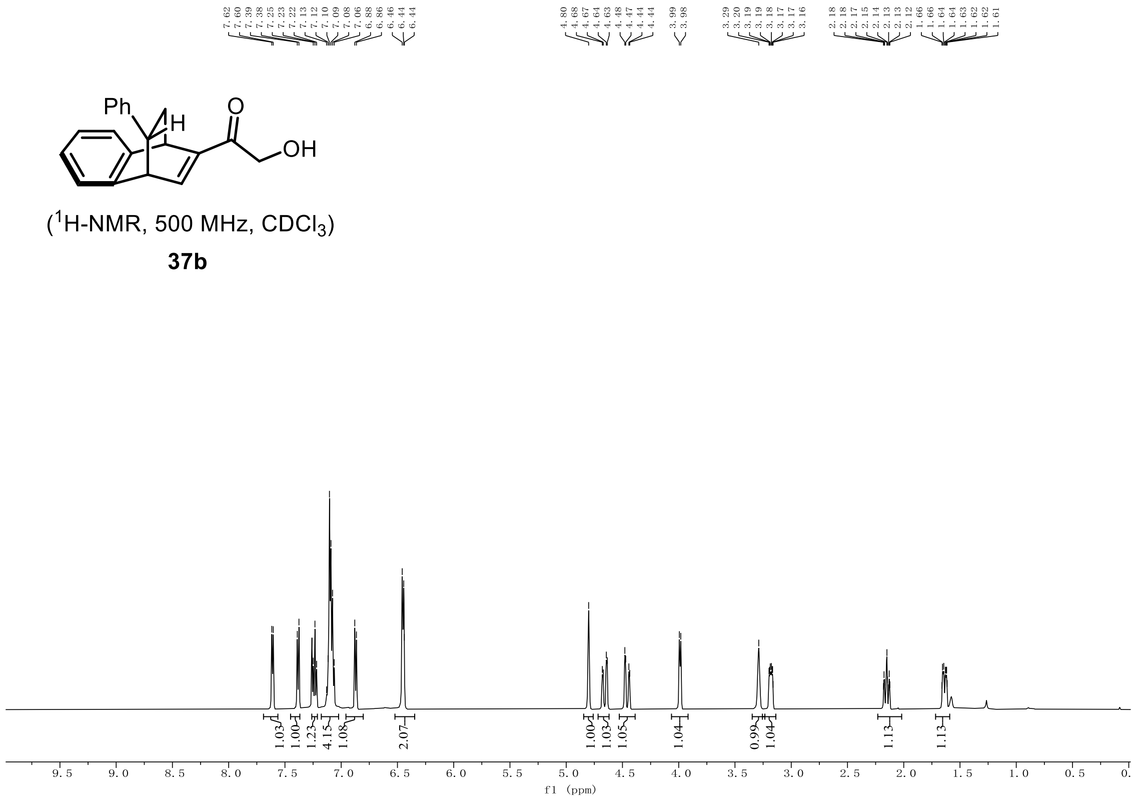

^{1}H-NMR Spectra of compound **37b.**

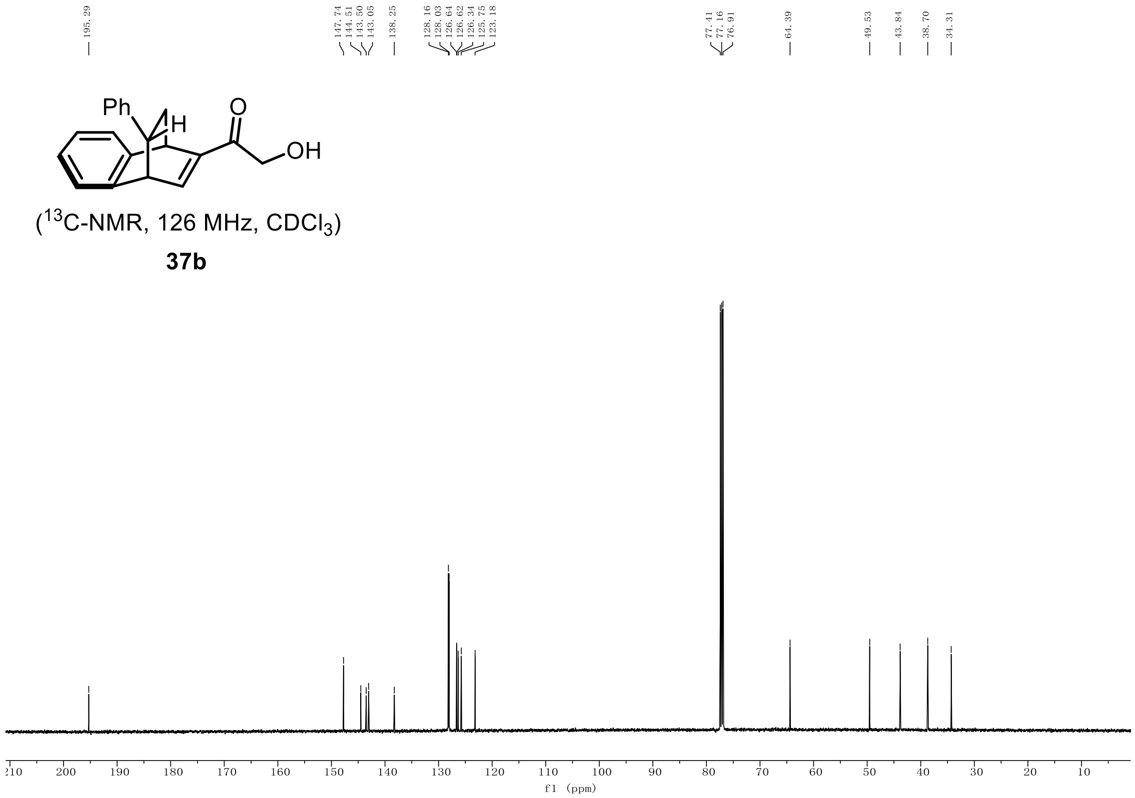

^{13}C-NMR Spectra of compound **37b.**

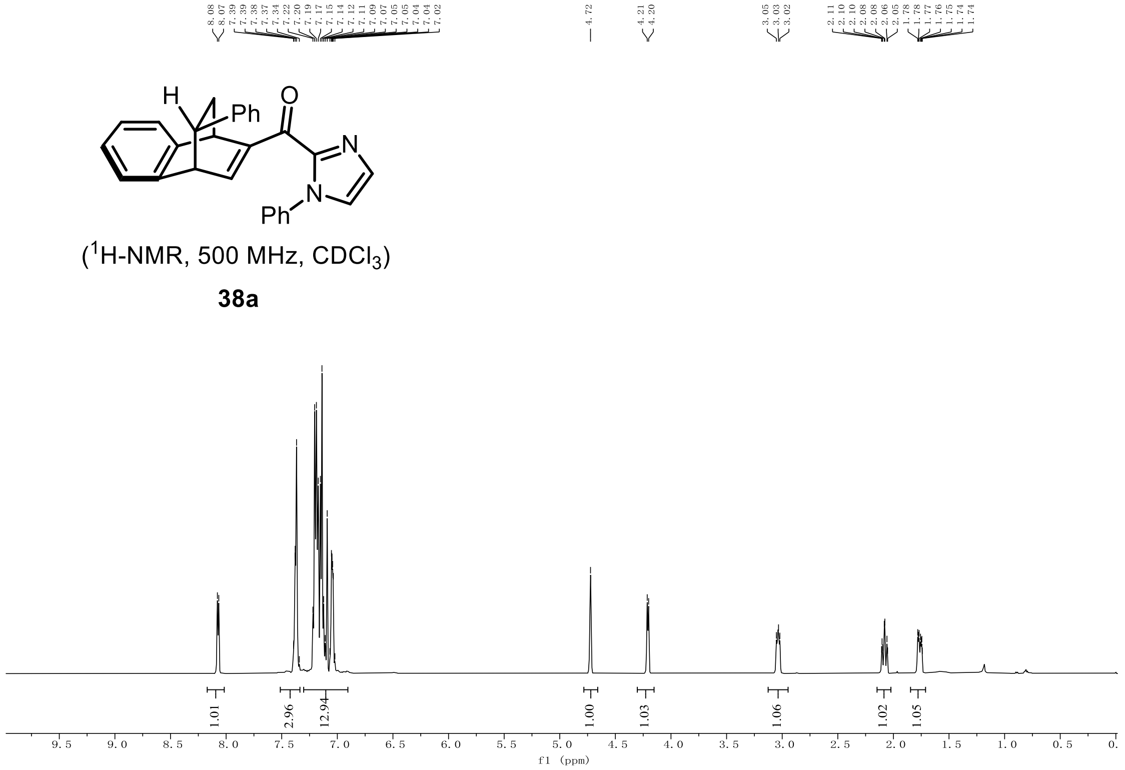

^{1}H-NMR Spectra of compound **38a.**

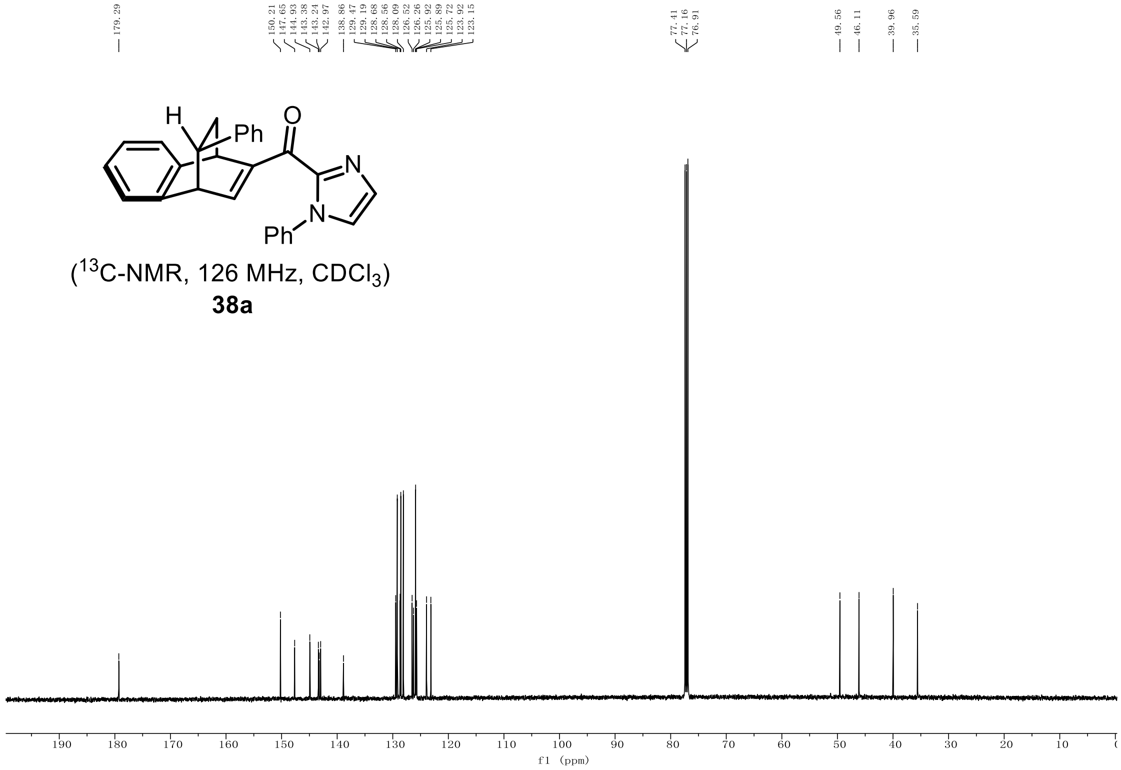

^{13}C-NMR Spectra of compound **38a.**

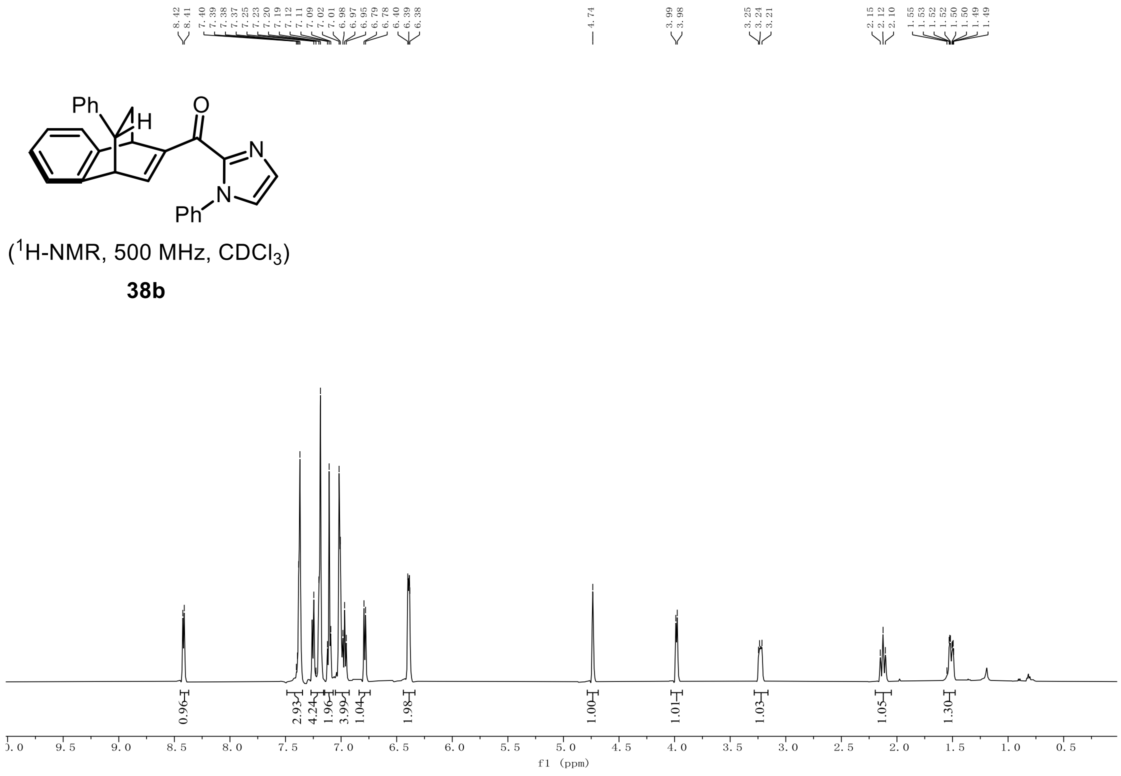

^{1}H-NMR Spectra of compound **38b.**

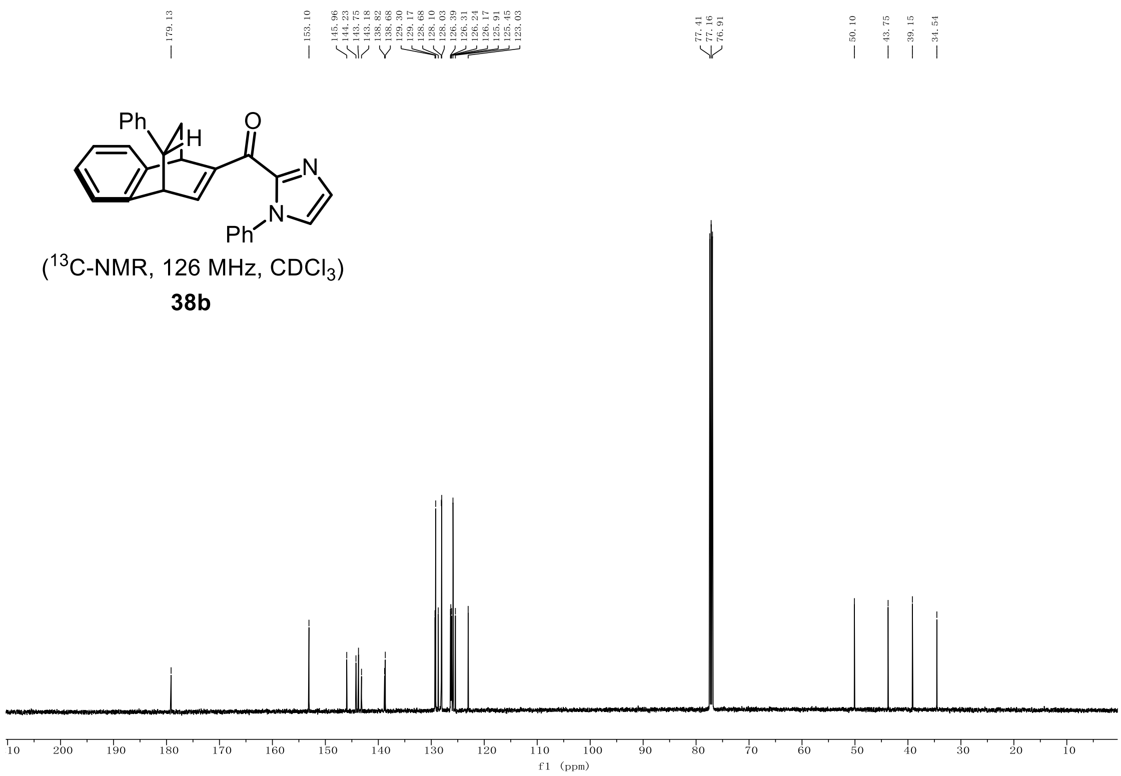

^{13}C-NMR Spectra of compound **38b.**

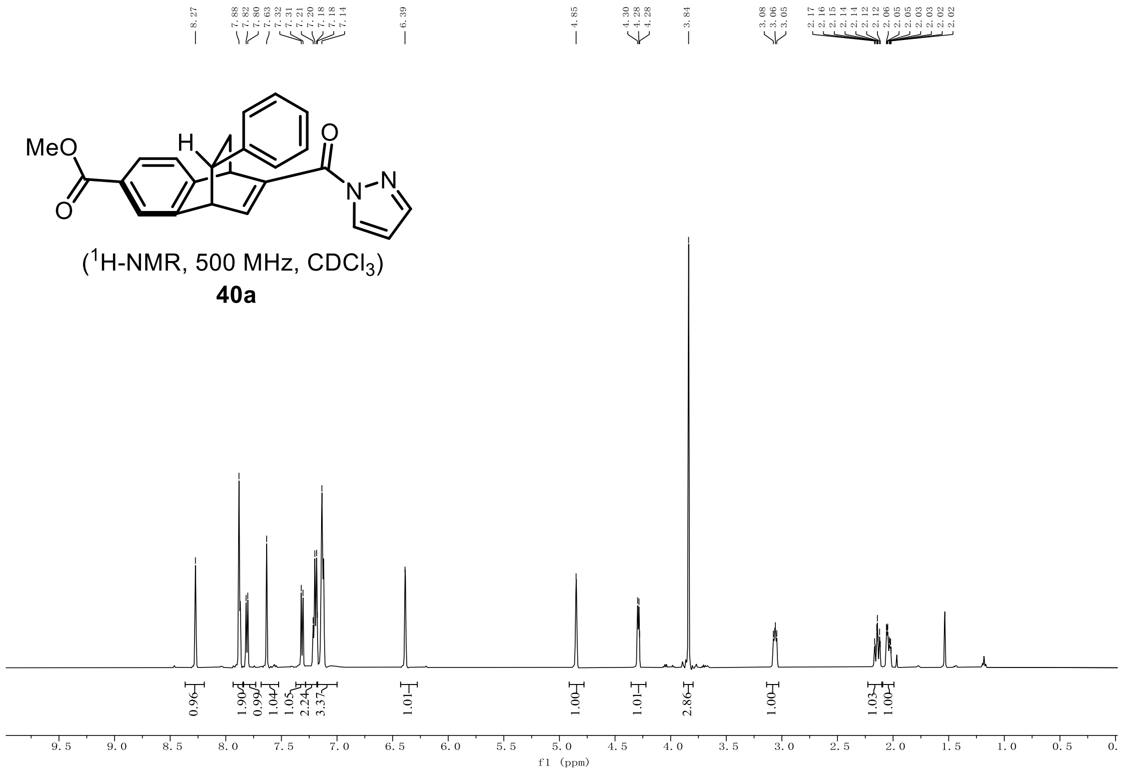

^{1}H-NMR Spectra of compound **40a.**

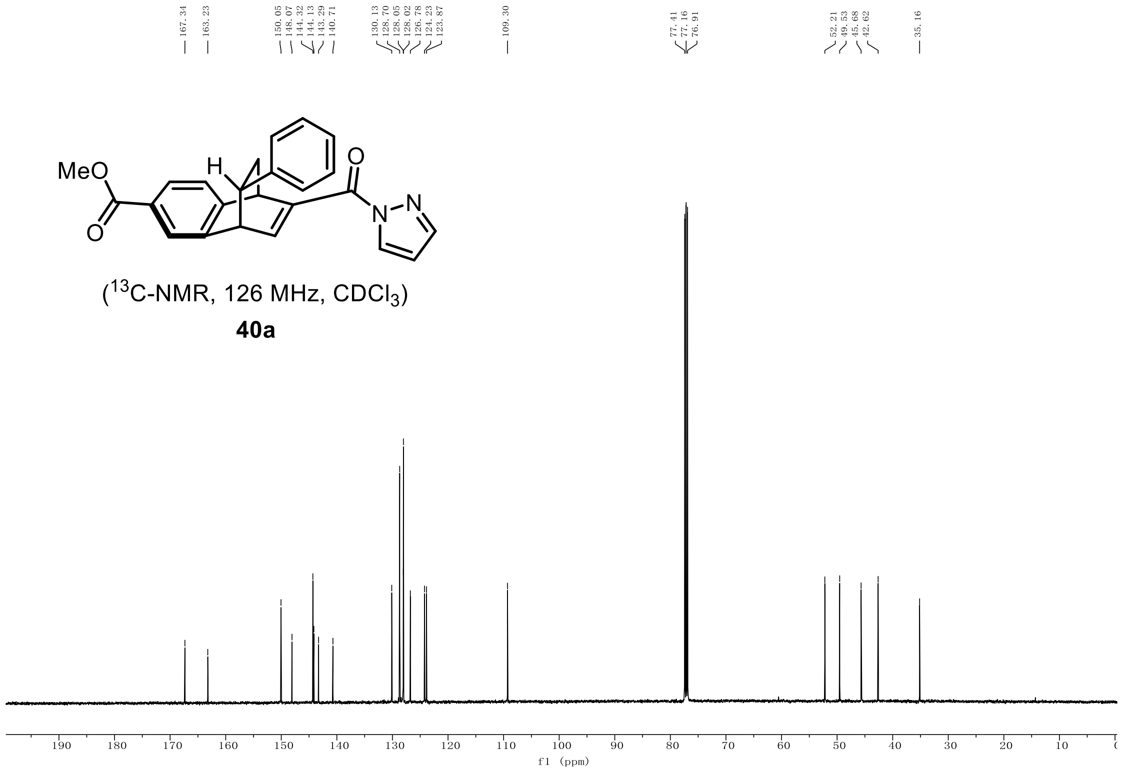

^{13}C-NMR Spectra of compound **40a.**

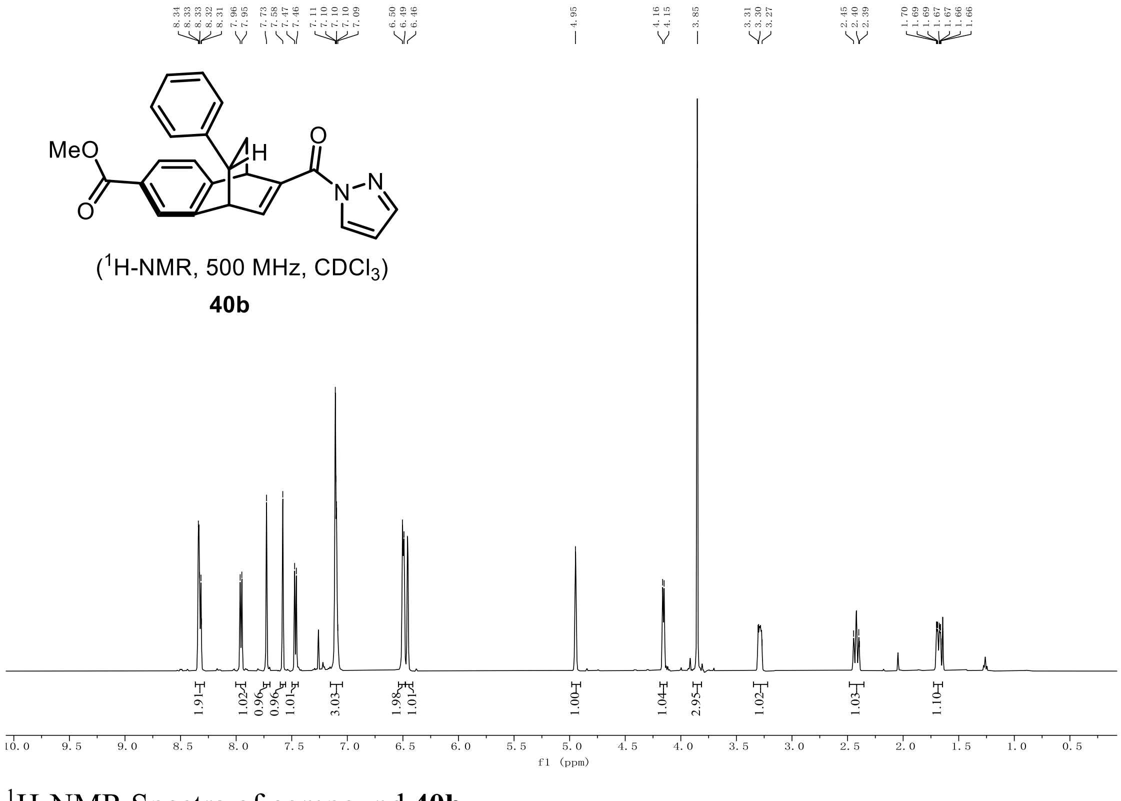

^{1}H-NMR Spectra of compound **40b.**

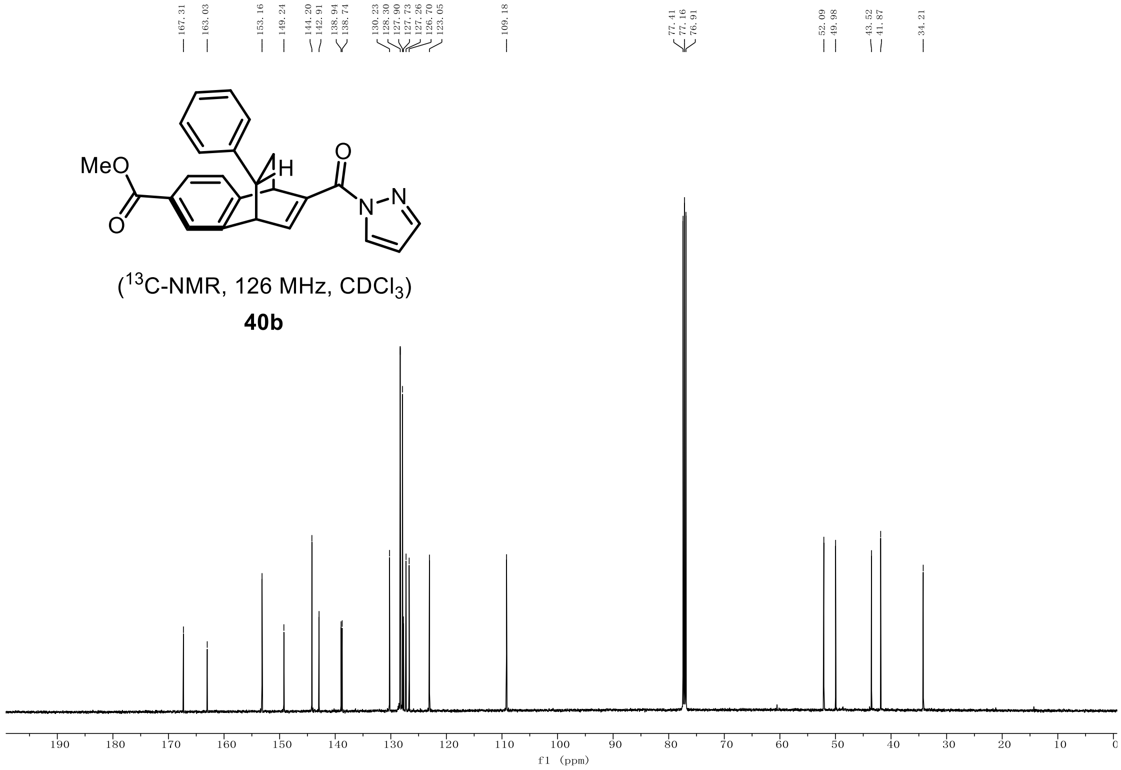

^{13}C-NMR Spectra of compound **40b.**

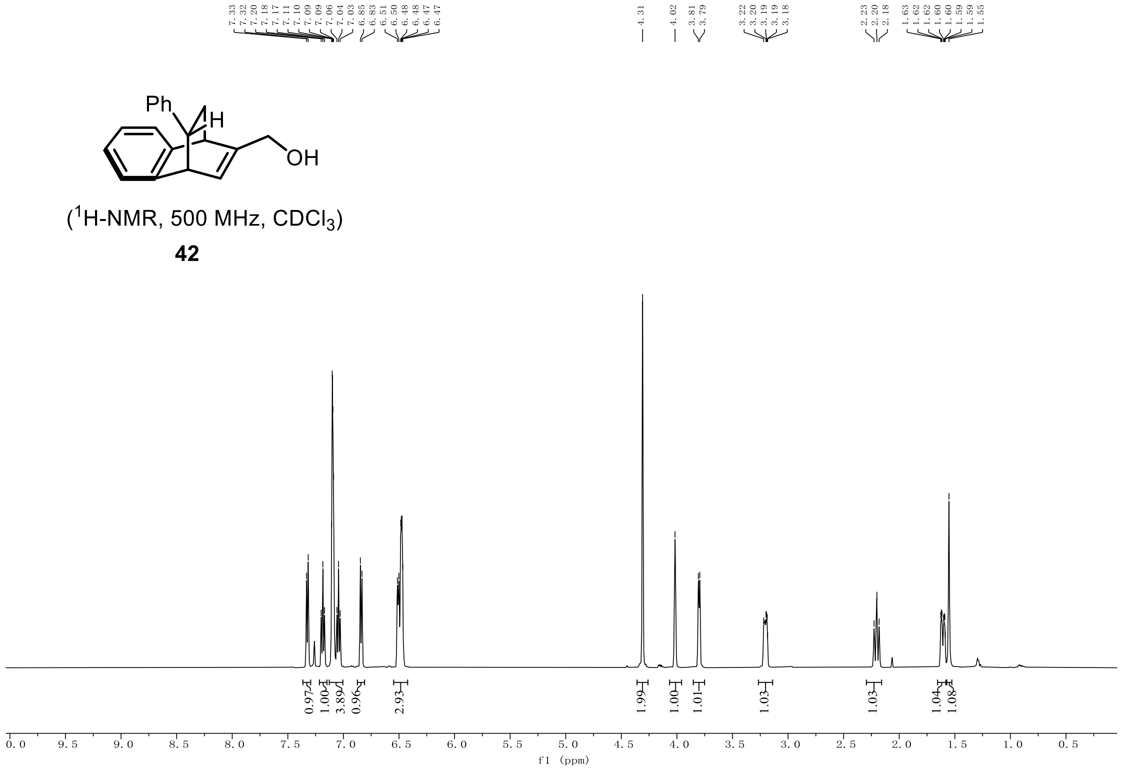

^{1}H-NMR Spectra of compound **42.**

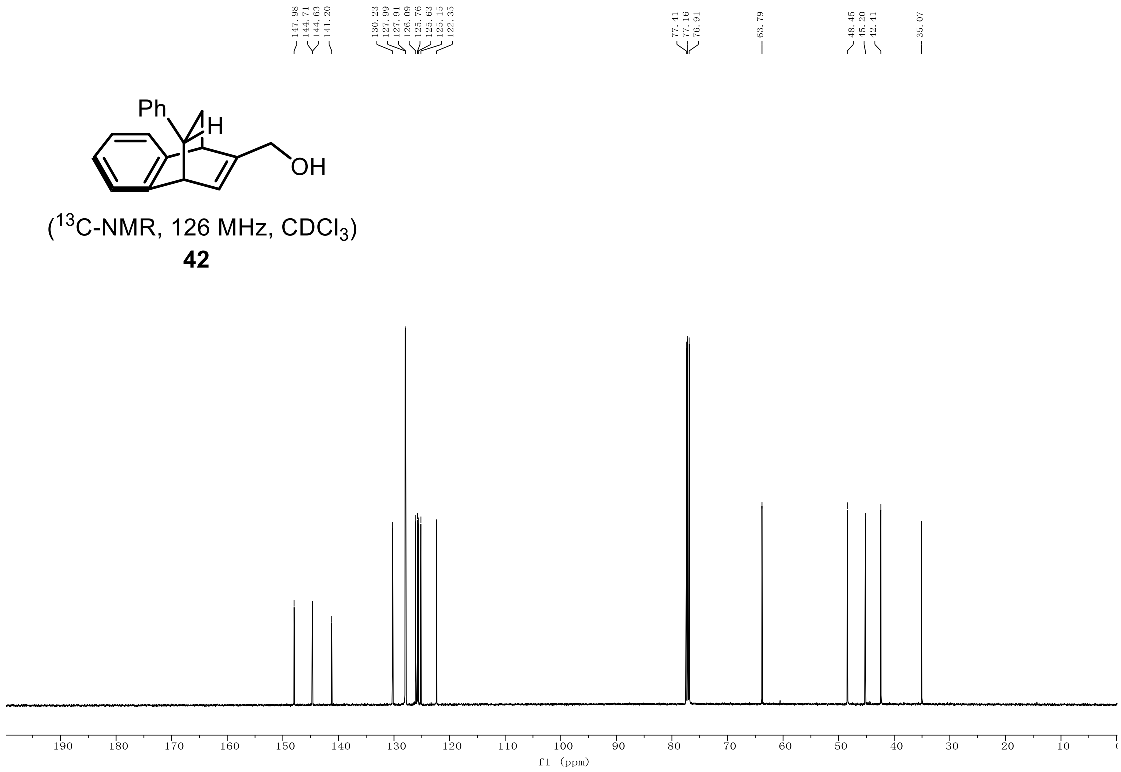

^{13}C-NMR Spectra of compound **42.**

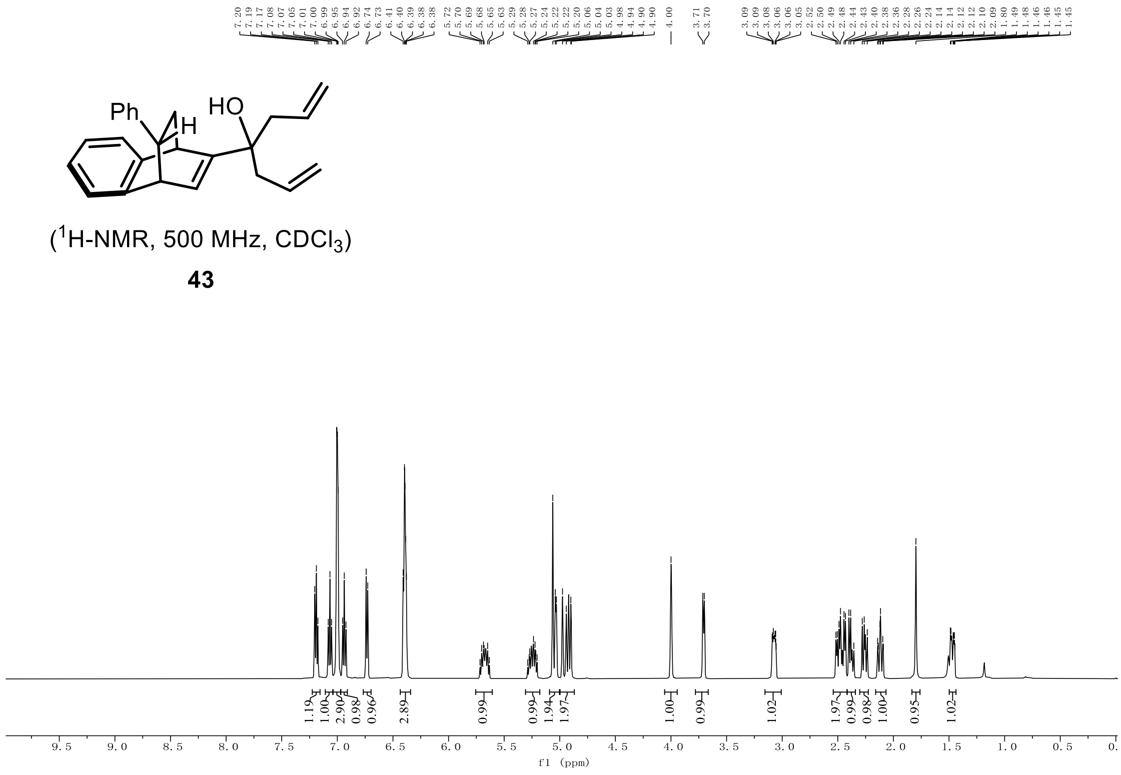

^{1}H-NMR Spectra of compound **43.**

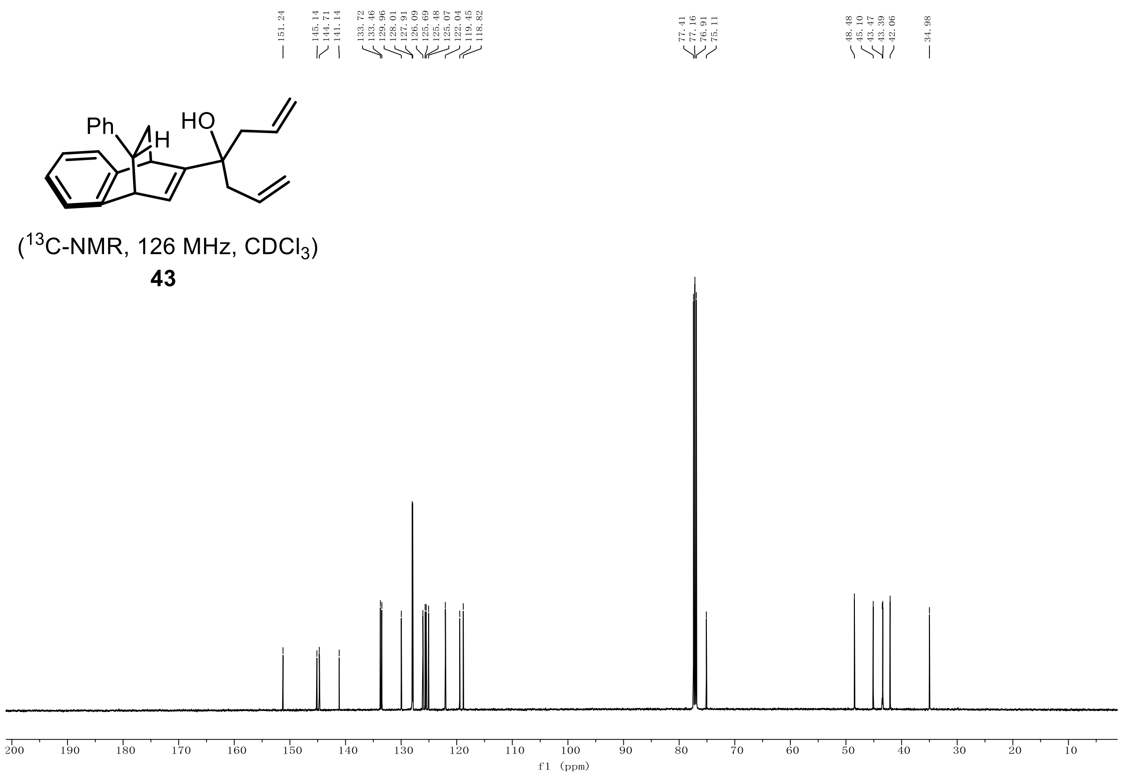

^{13}C-NMR Spectra of compound **43.**

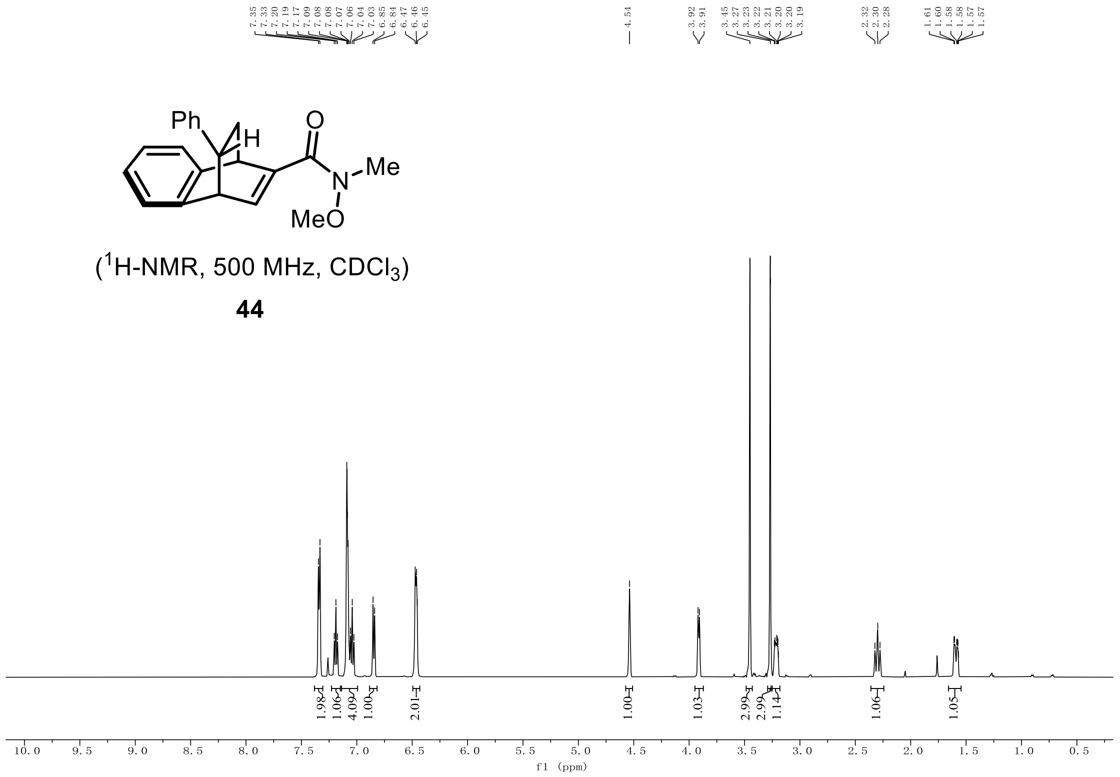

^{1}H-NMR Spectra of compound **44.**

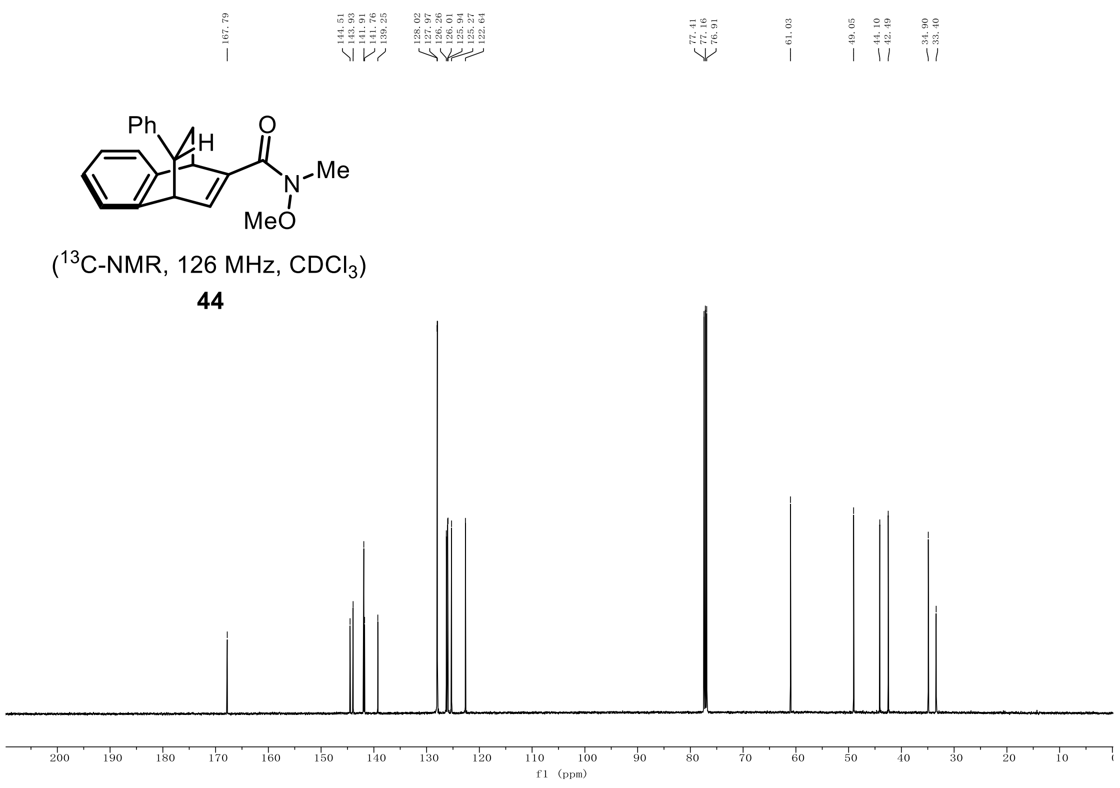

^{13}C-NMR Spectra of compound **44.**

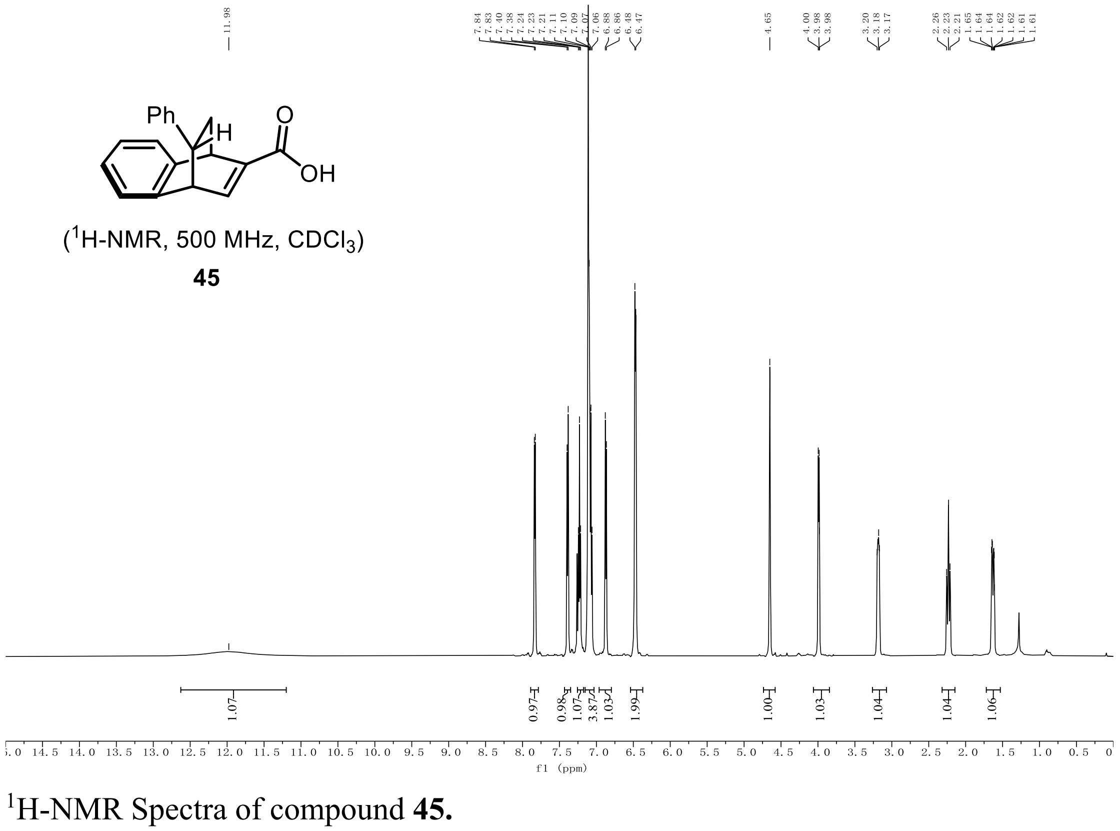

^{1}H-NMR Spectra of compound **45.**

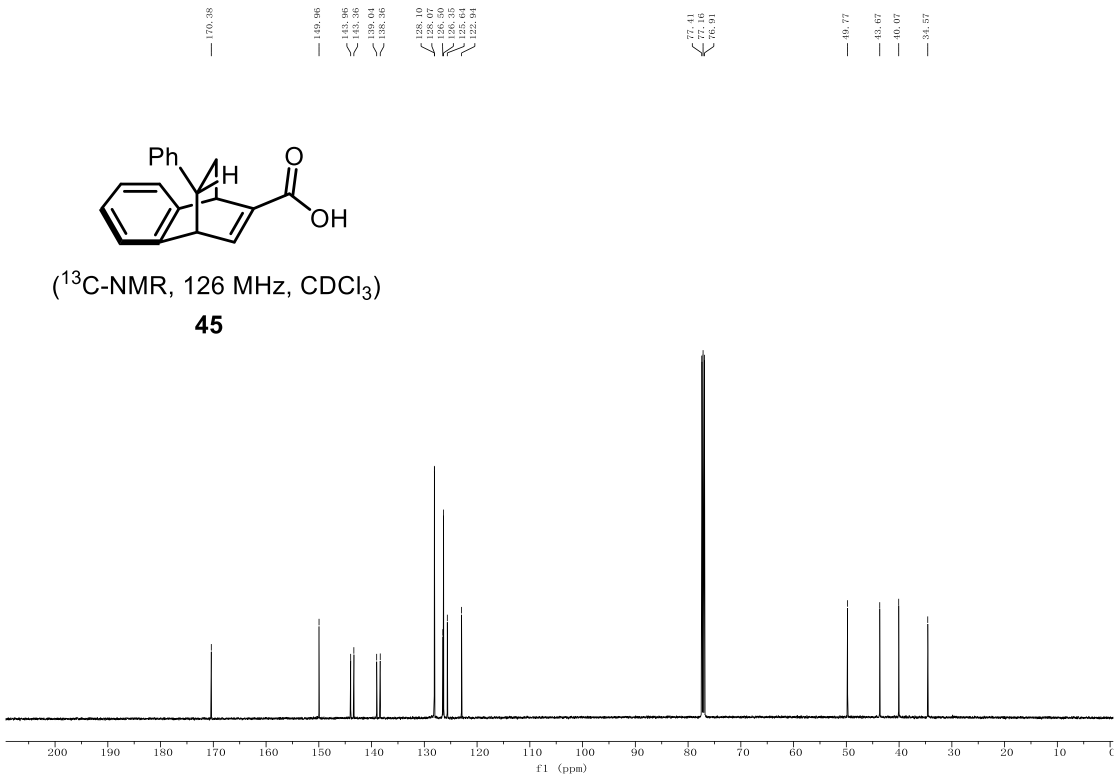

^{13}C-NMR Spectra of compound **45.**

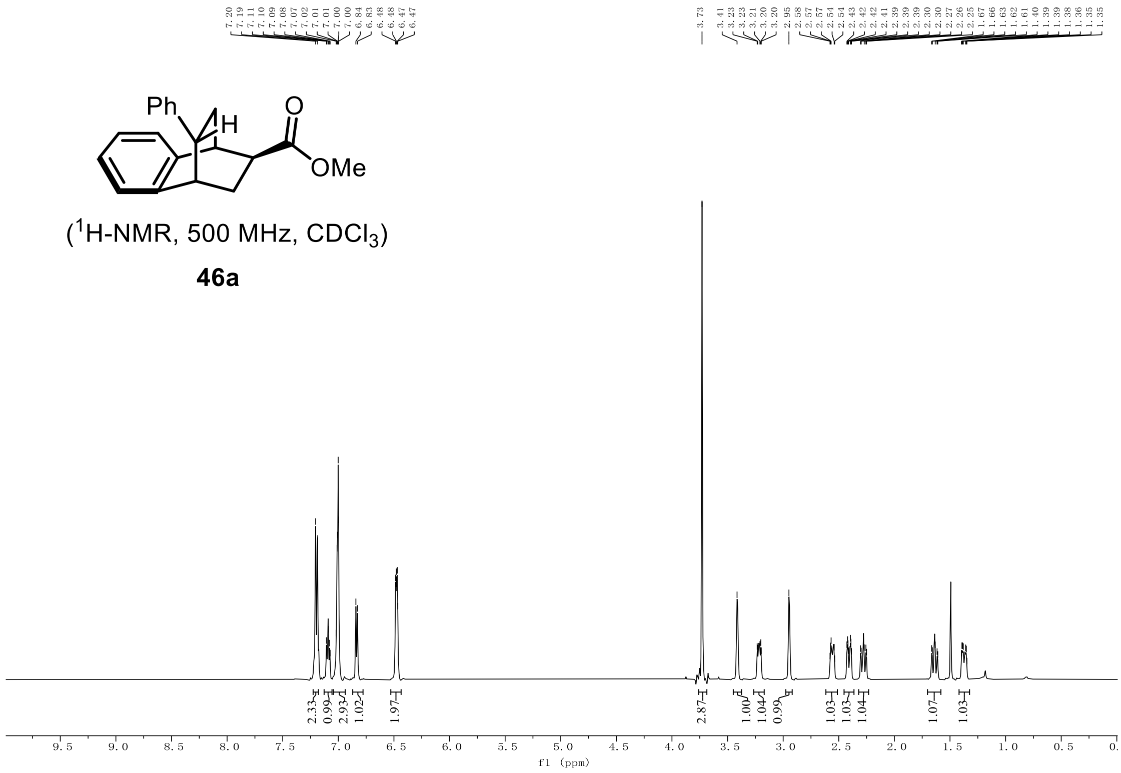

^{1}H-NMR Spectra of compound **46a.**

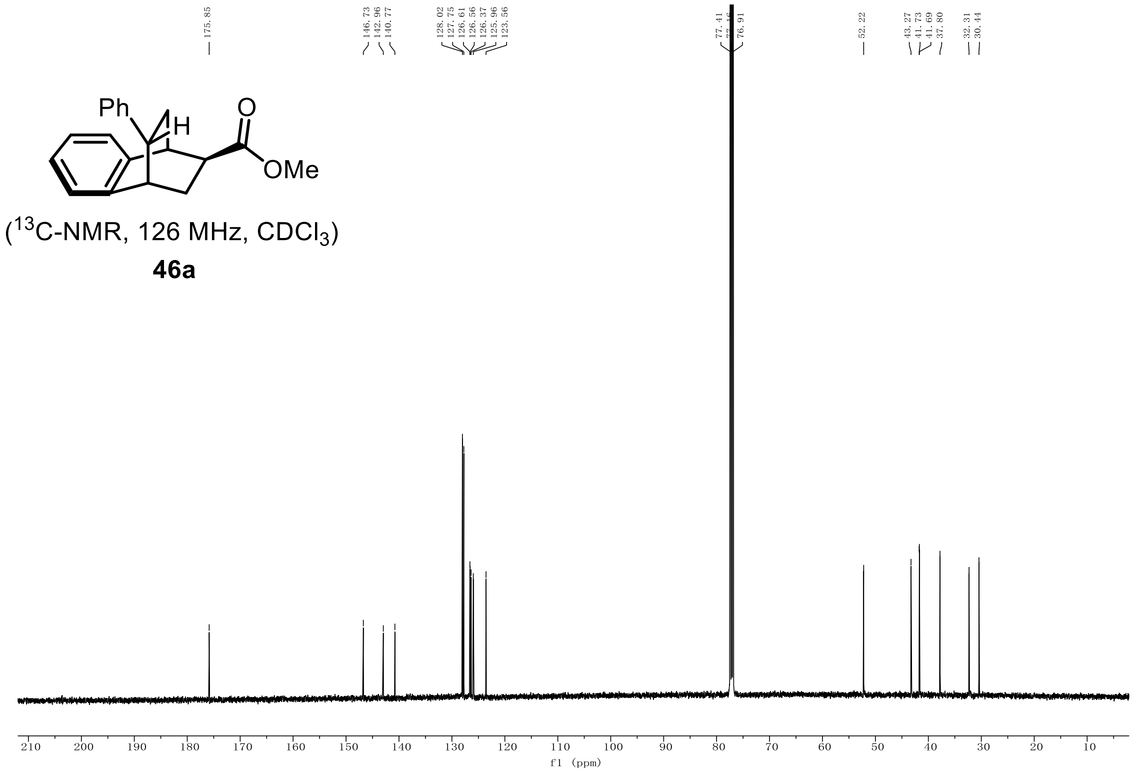

^{13}C-NMR Spectra of compound **46a.**

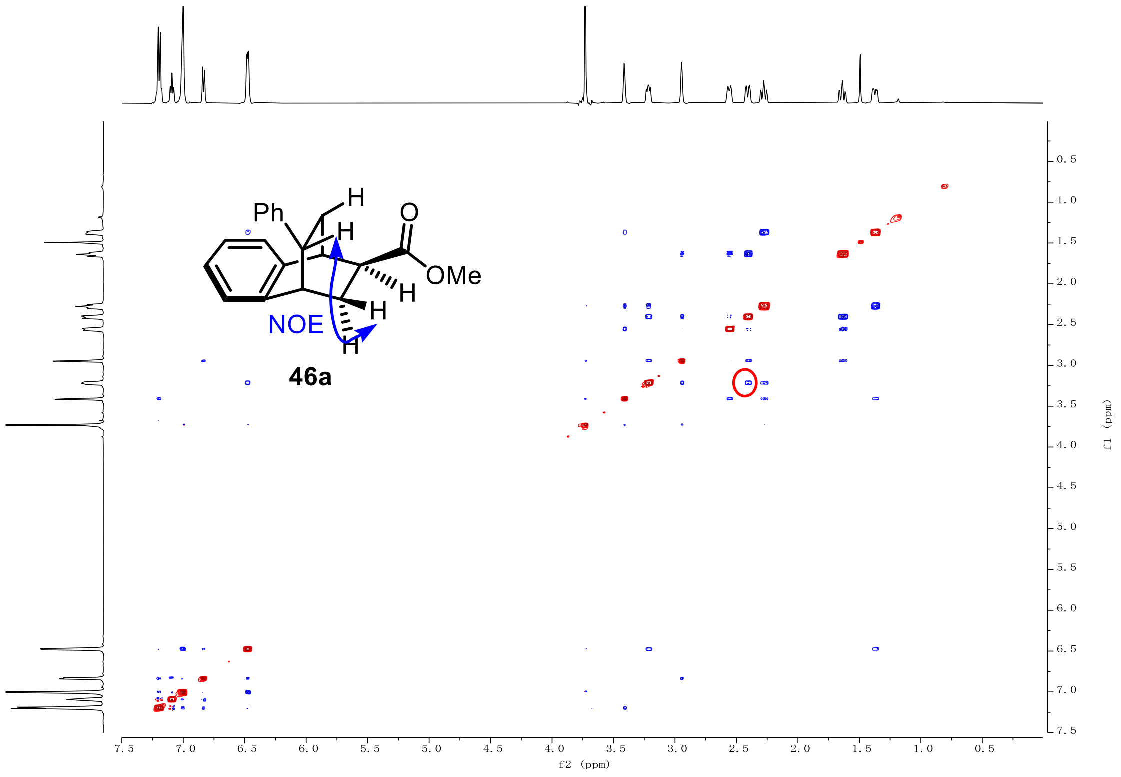

Noesy Spectra of compound **46a.**

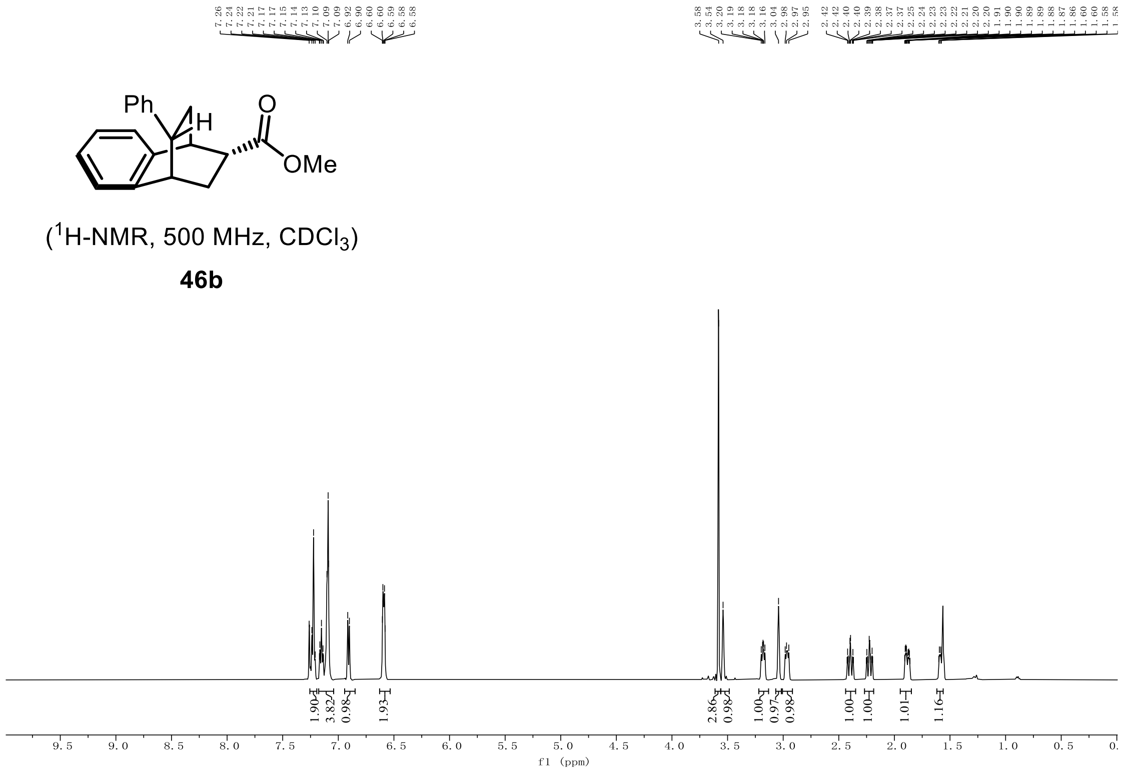

^{1}H-NMR Spectra of compound **46b.**

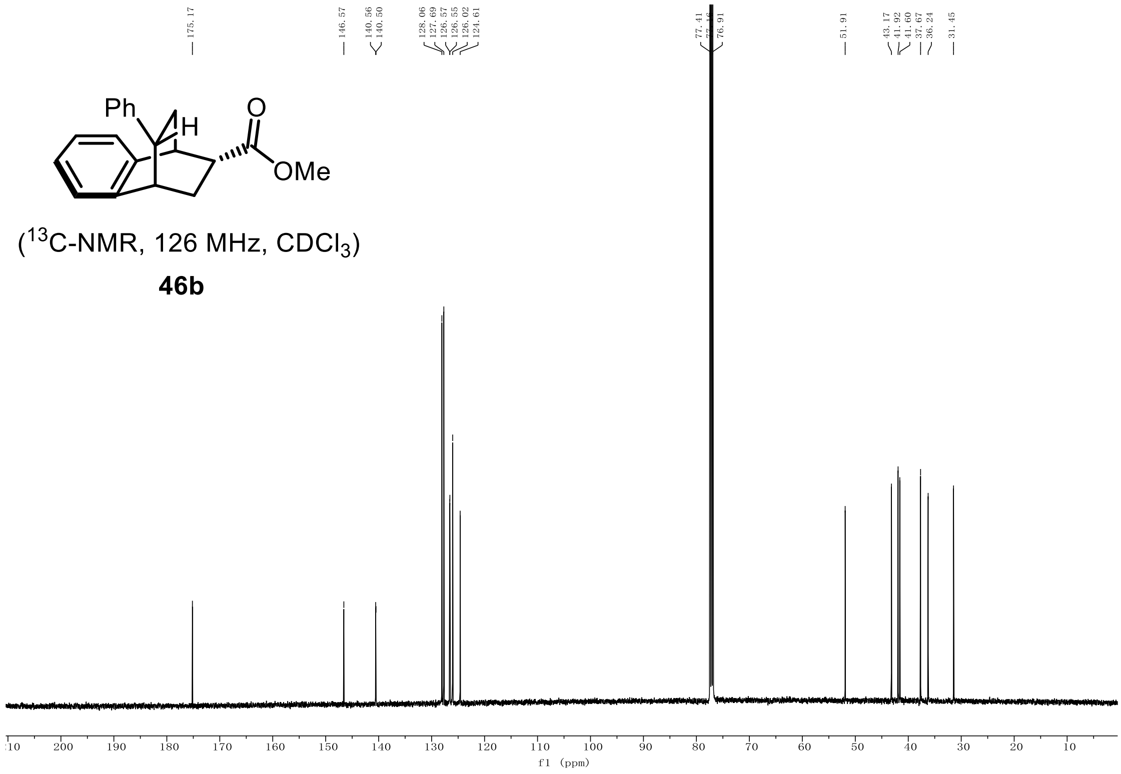

^{13}C-NMR Spectra of compound **46b.**

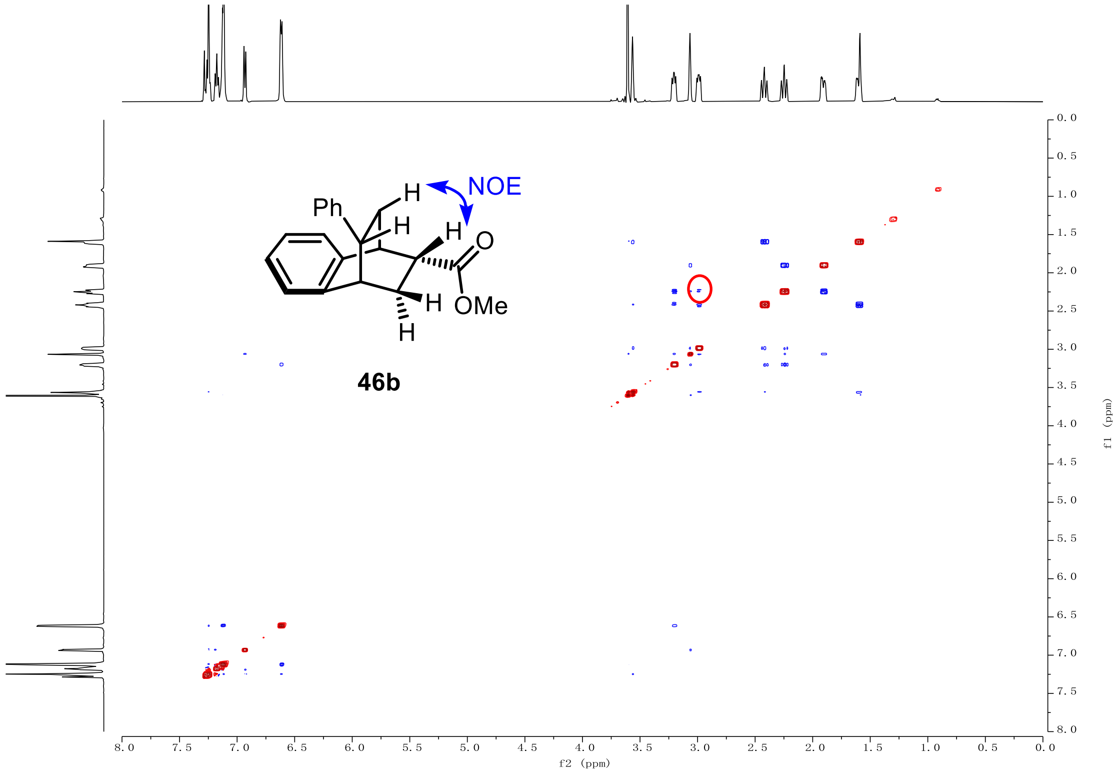

Noesy Spectra of compound **46b.**

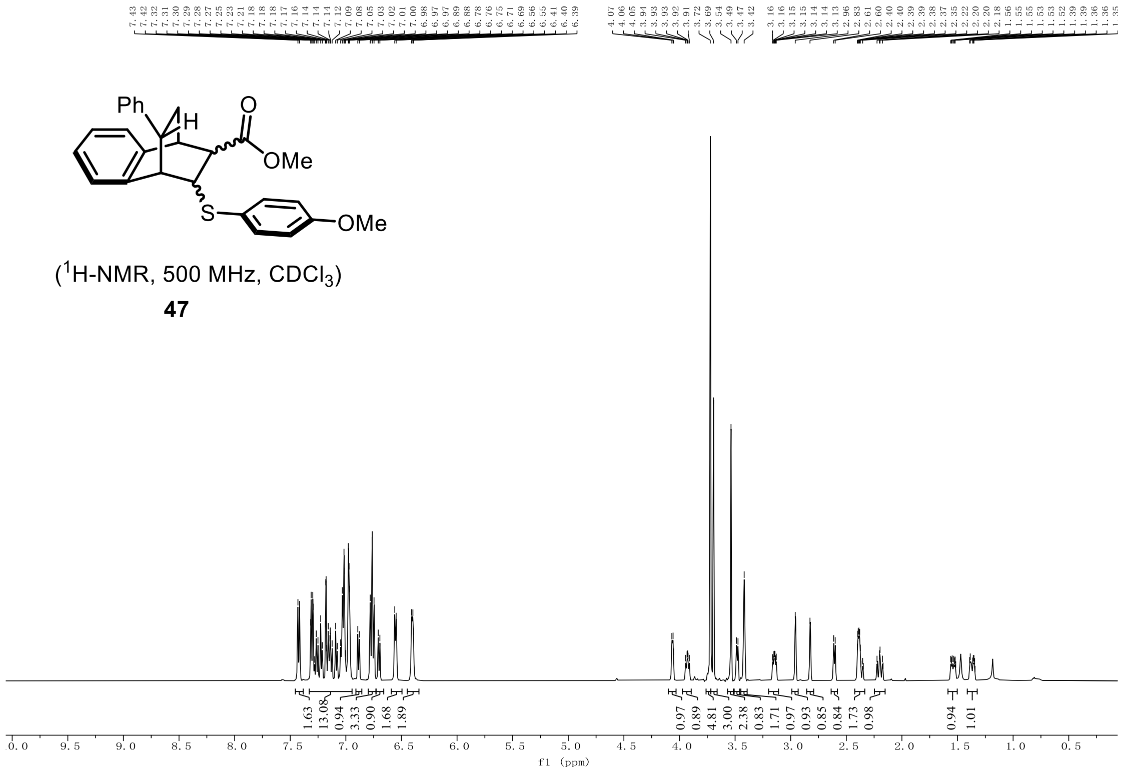

^{1}H-NMR Spectra of compound **47.**

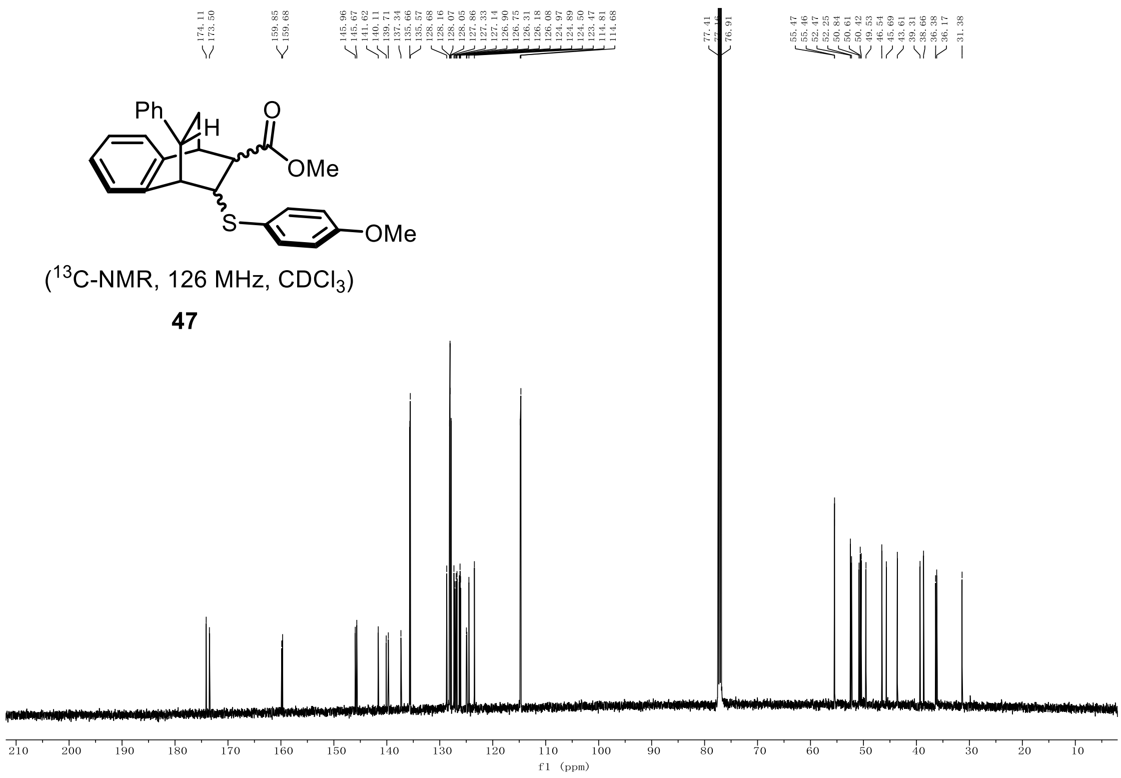

^{13}C-NMR Spectra of compound **47.**

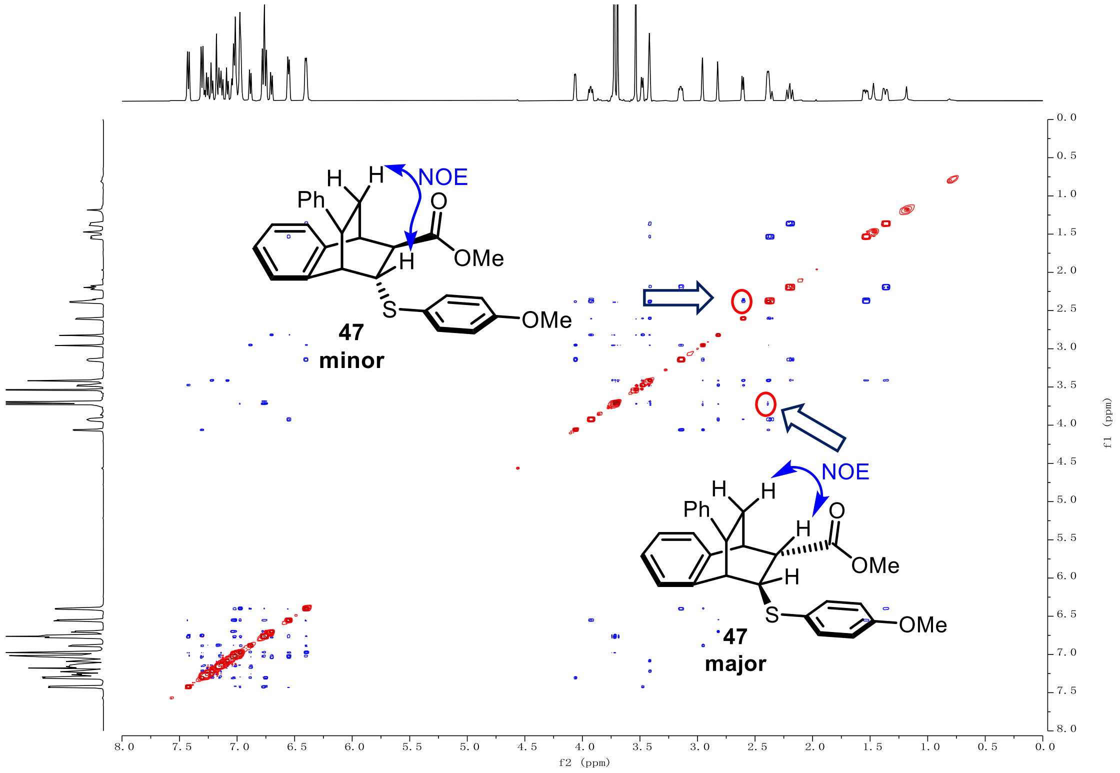

Noesy Spectra of compound **47.**

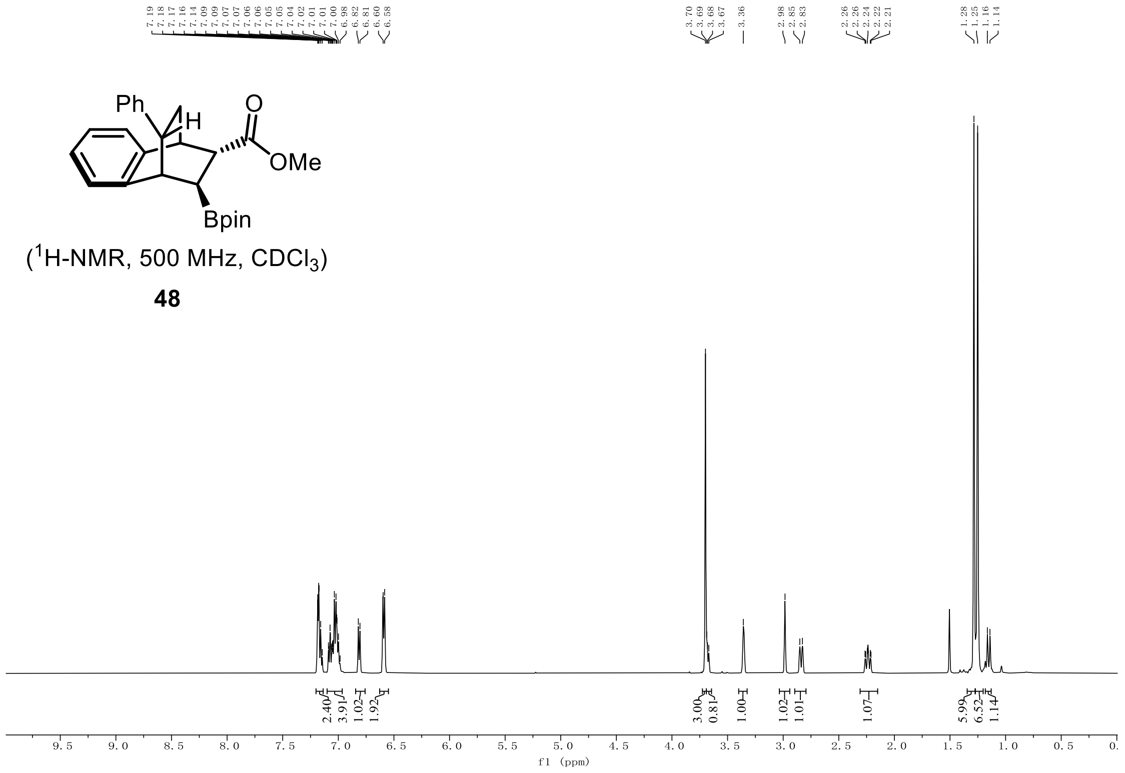

^{1}H-NMR Spectra of compound **48.**

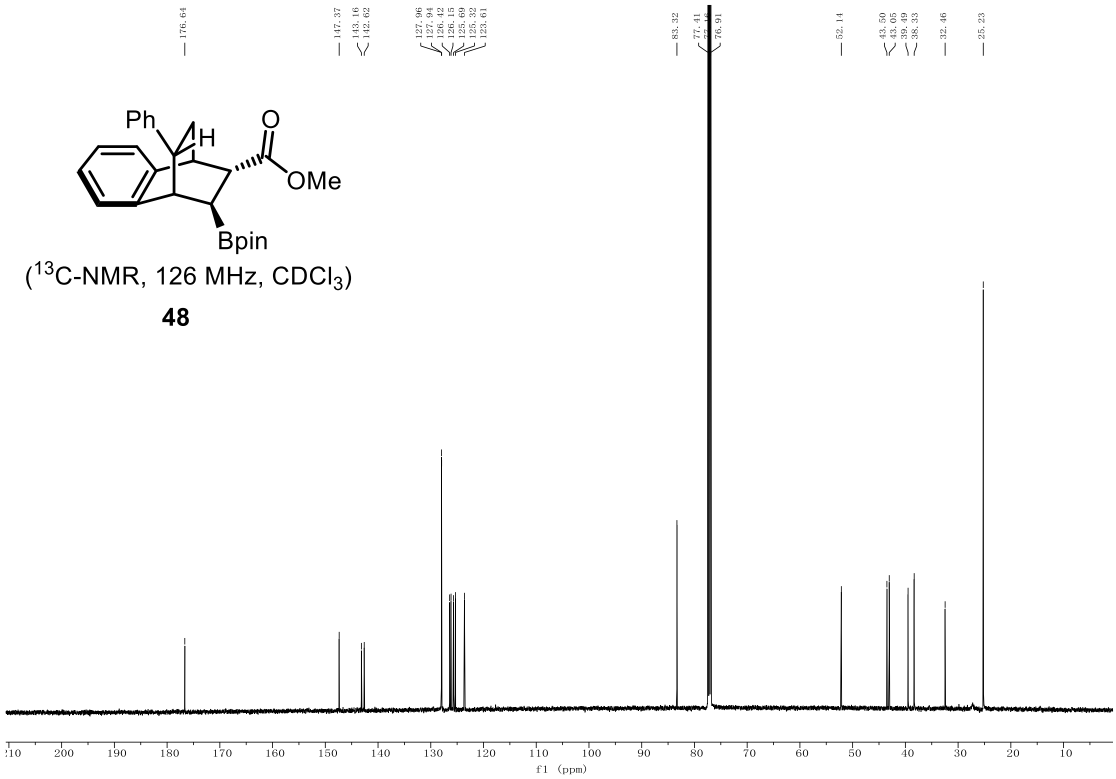

^{13}C-NMR Spectra of compound **48.**

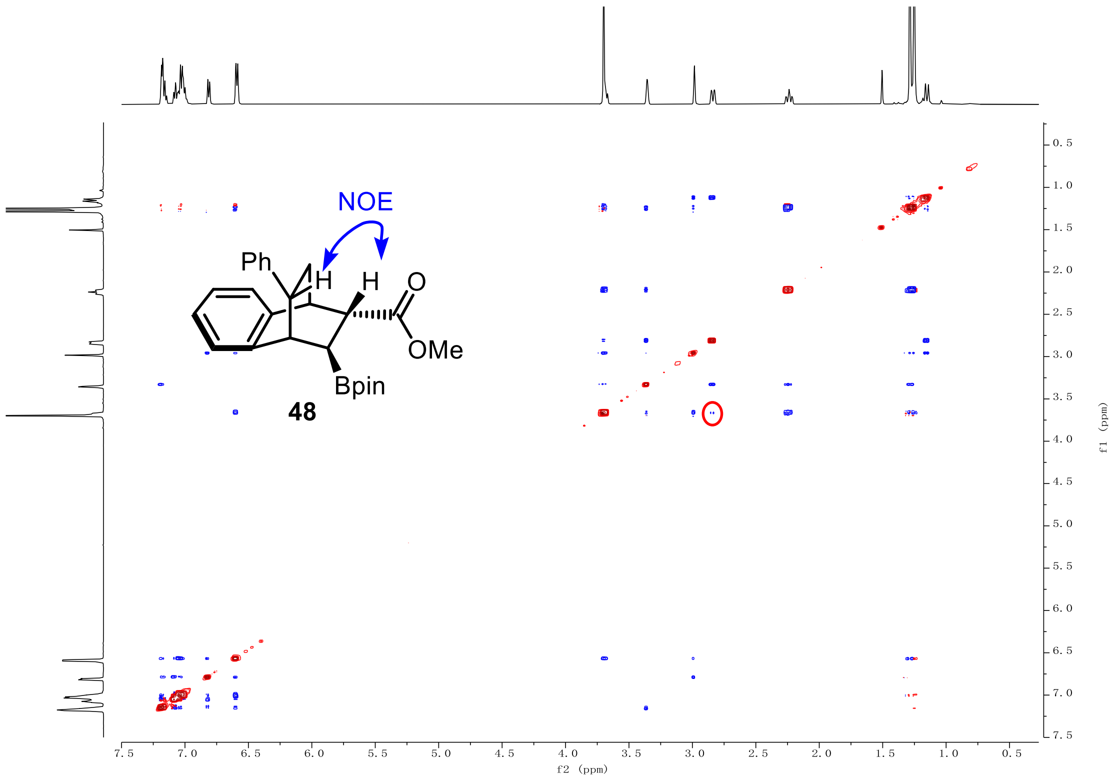

Noesy Spectra of compound **48.**

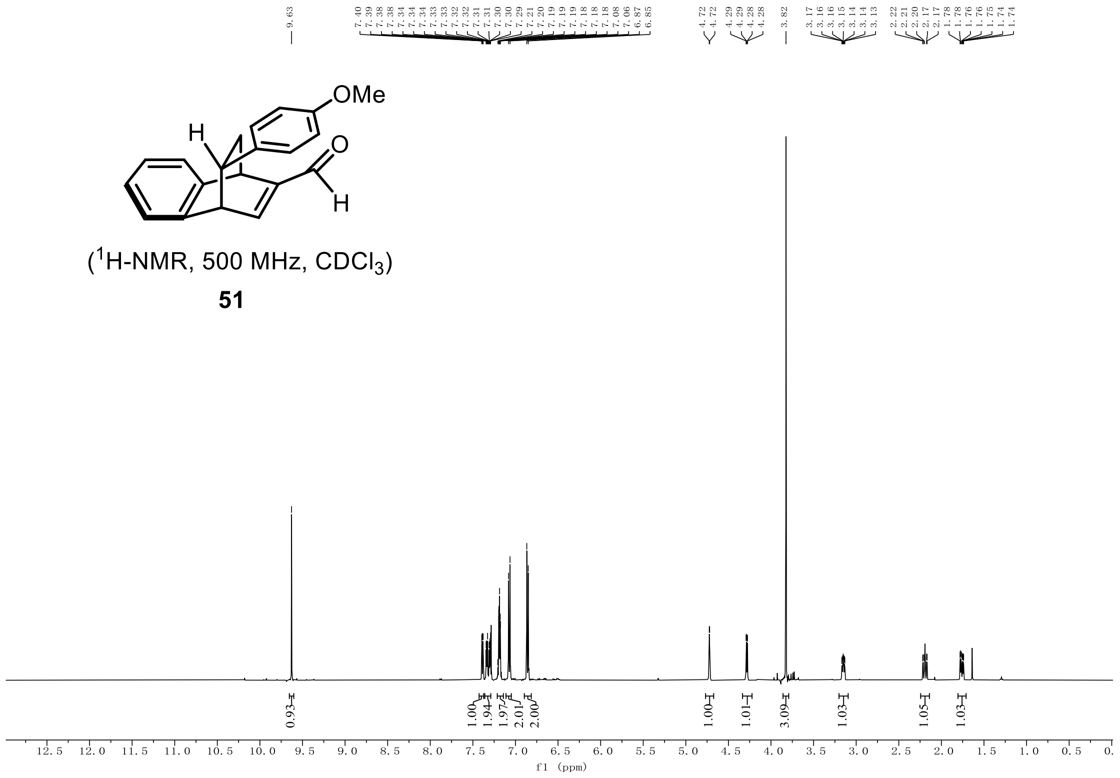

^{1}H-NMR Spectra of compound **51.**

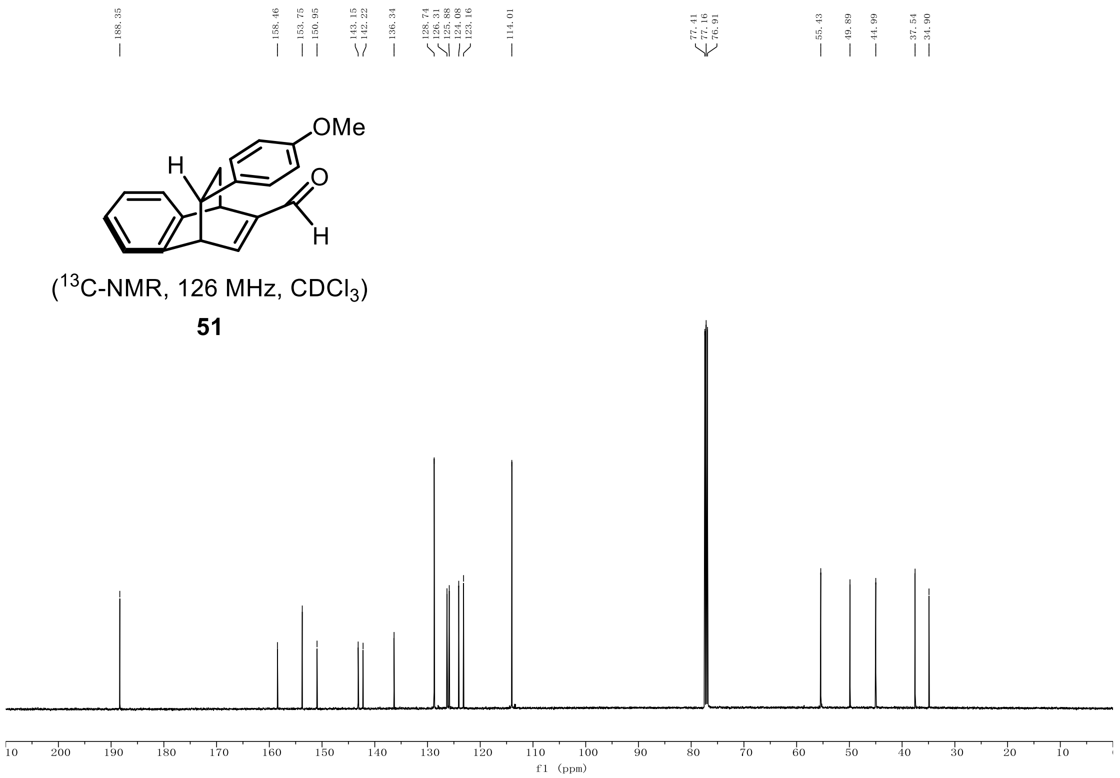

^{13}C-NMR Spectra of compound **51.**

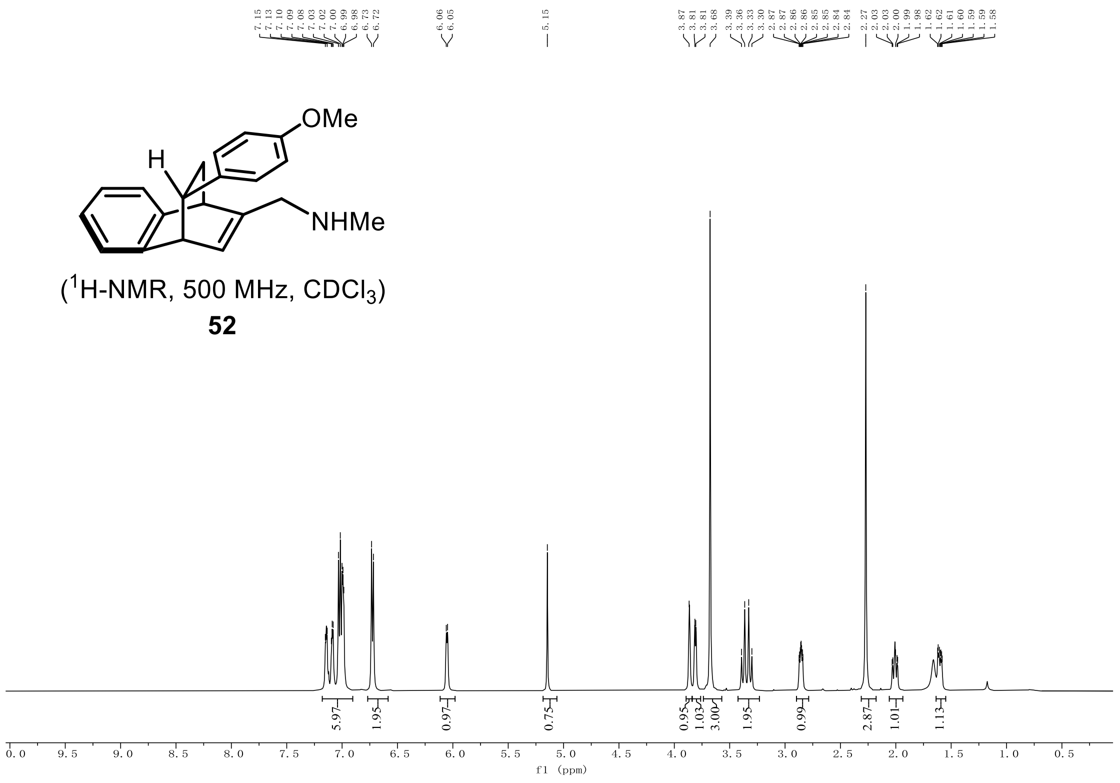

^{1}H-NMR Spectra of compound **52.**

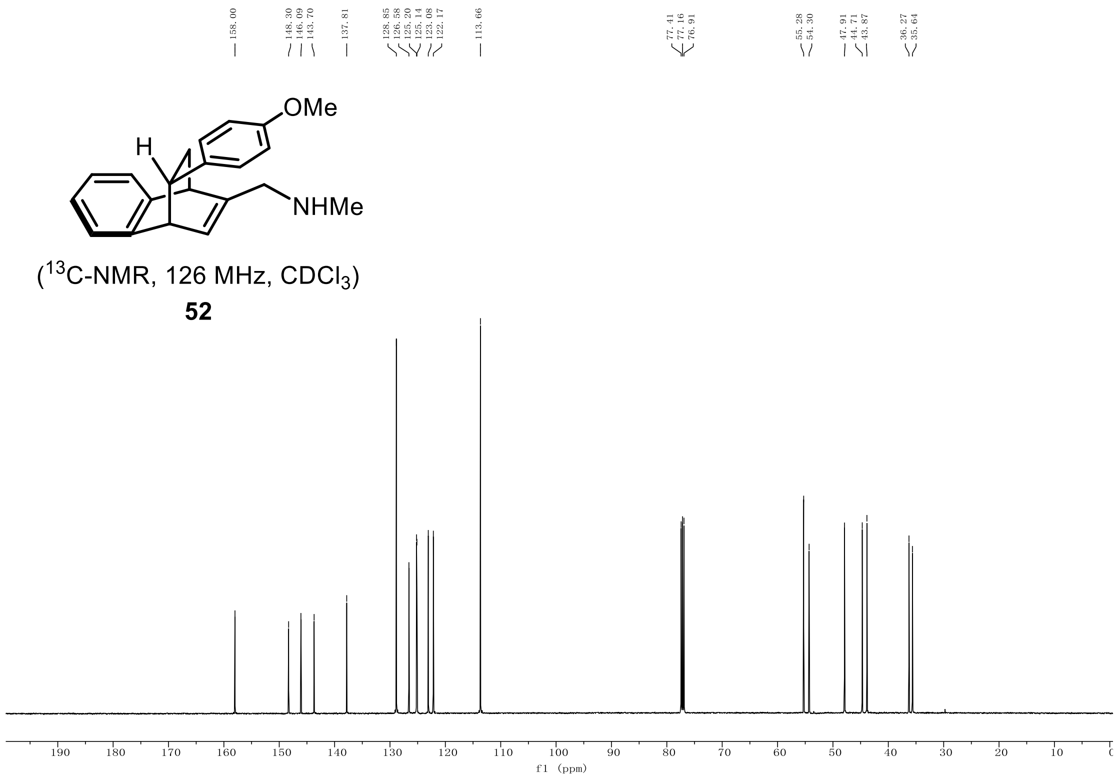

^{13}C-NMR Spectra of compound **52.**

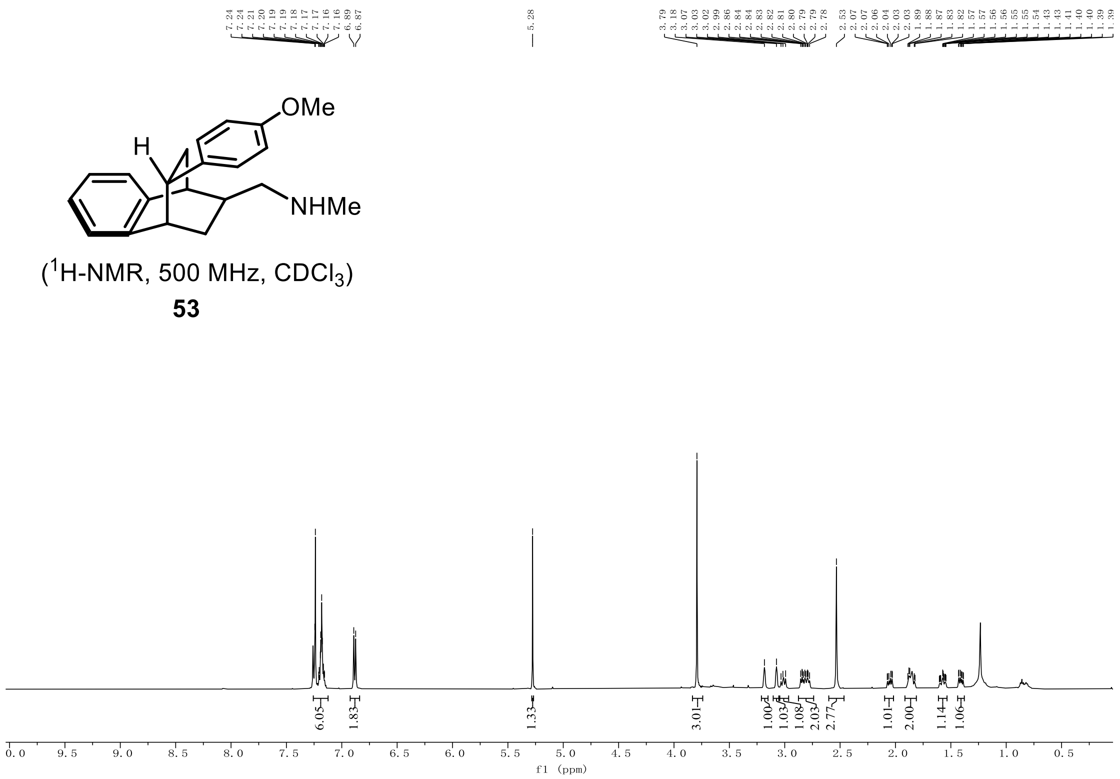

^{1}H-NMR Spectra of compound **53.**

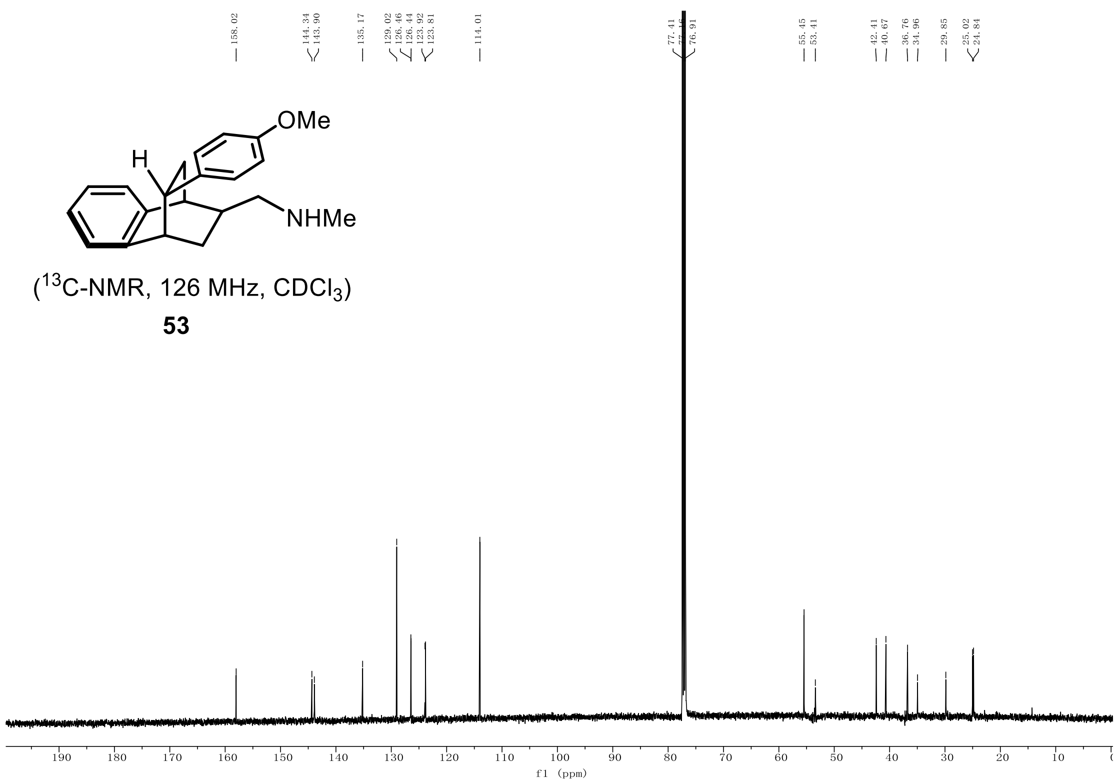

^{13}C-NMR Spectra of compound **53.**

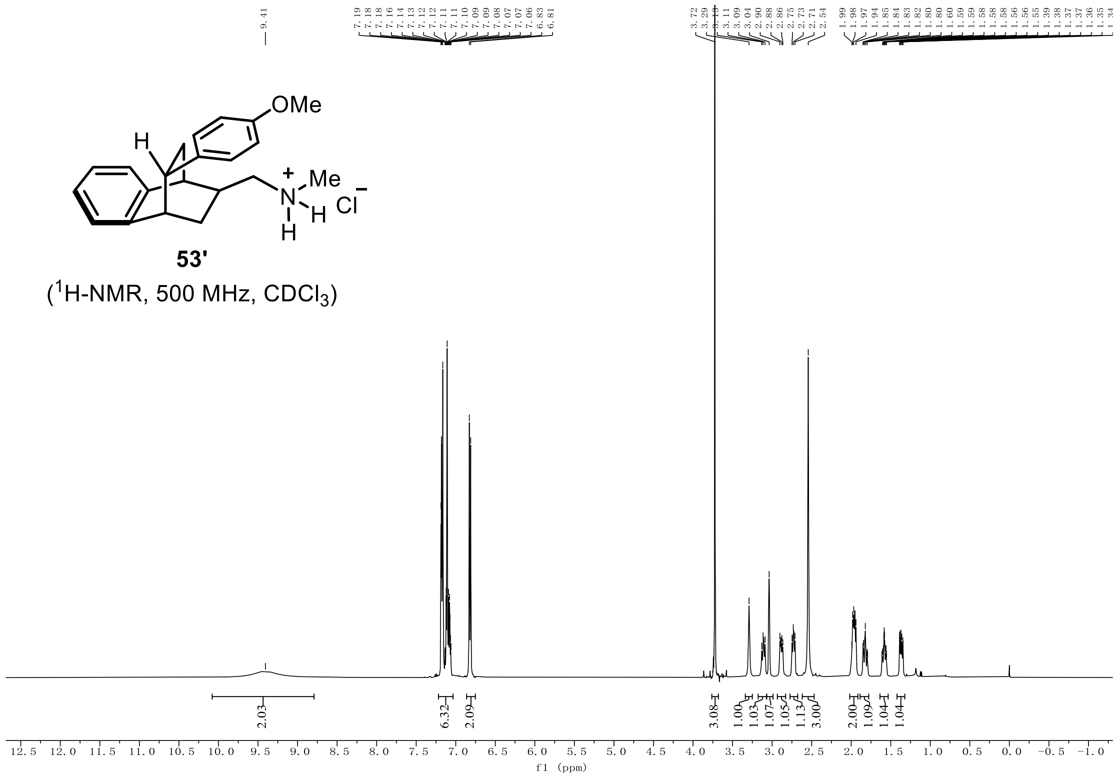

^{1}H-NMR Spectra of compound **53’.**

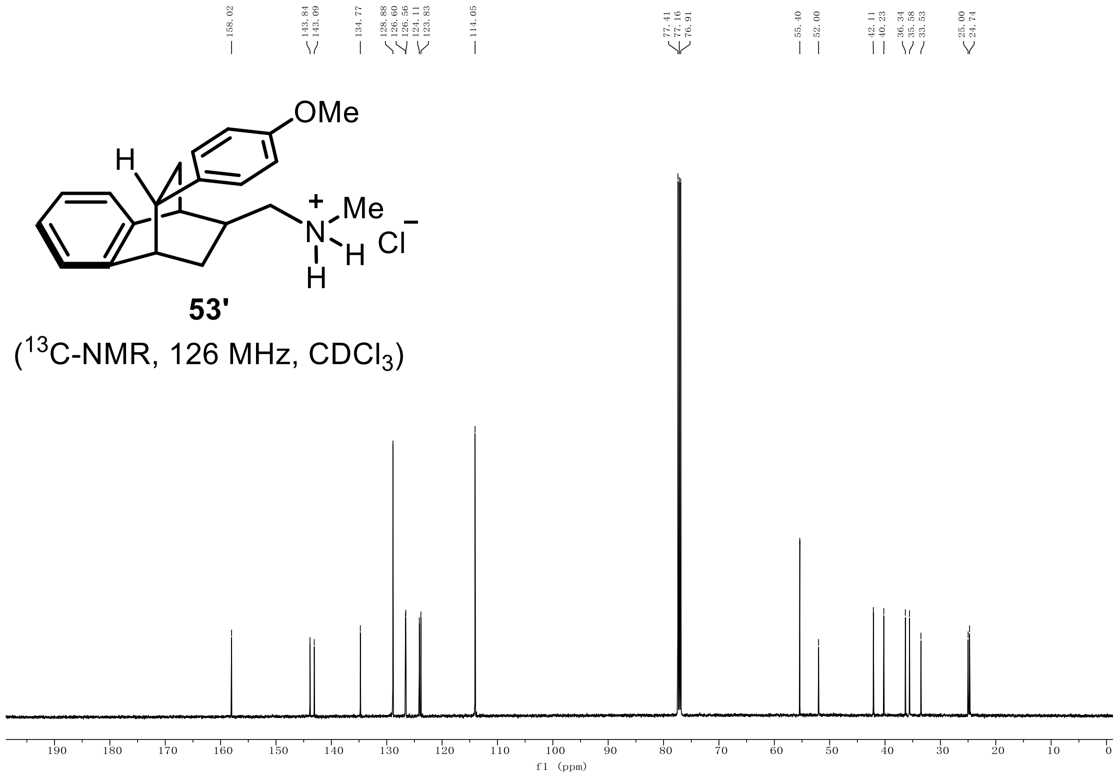

^{13}C-NMR Spectra of compound **53’.**

Supplementary References

[1] For details of the light information, see: Sapphire Blue Waterproof LED Flex Strips LED Tape Lights - Low power consumption, infinite uses. Blue LEDs - Beautiful LED Sapphire Blue Color! Most popular color offered. (creativelightings.com)

[2] For details of the light information, see: realUV™ LED Strip Lights – Waveform Lighting

[3] Details of the light information, see: High power LEDs - Vapourtec.

[4] Ir cat. Triplet energy: (a) K. Teegardin, J. I. Day, J. Chan, J. Weaver, *Org. Process Res. Dev.* **2016**, 20, 1156-1163. (b) M. E. Daub, H. Jung, B. J. Lee, J. Won, M. H. Baik, T. P. Yoon, *J. Am. Chem. Soc.* **2019**, 141, 9543-9547.

[5] T. R. Blum, Z. D. Miller, D. M. Bates, I. A. Guzei, T. P. Yoon, *Science* **2016**, 354, 1391-1395.

[6] J. Zhao, J. L. Brosmer, Q. Tang, Z. Yang, K. N. Houk, P. L. Diaconescu, O. Kwon, *J. Am. Chem. Soc.* **2017**, 139, 9807-9810.

[7] M. J. Frisch, G. W. Trucks, H. B. Schlegel, G. E. Scuseria, M. A. Robb, J. R. Cheeseman, G. Scalmani, V. Barone, G. A. Petersson, H. Nakatsuji, X. Li, M. Caricato, A. V. Marenich, J. Bloino, B. G. Janesko, R. Gomperts, B. Mennucci, H. P. Hratchian, J. V. Ortiz, A. F. Izmaylov, J. L. Sonnenberg, F. Ding, F. Lipparini, F. Egidi, J. Goings, B. Peng, A. Petrone, T. Henderson, D. Ranasinghe, V. G. Zakrzewski, J. Gao, N. Rega, G. Zheng, W. Liang, M. Hada, M. Ehara, K. Toyota, R. Fukuda, J. Hasegawa, M. Ishida, T. Nakajima, Y. Honda, O. Kitao, H. Nakai, T. Vreven, K. Throssell, Jr. J. A. Montgomery, J. E. Peralta, F. Ogliaro, M. J. Bearpark, J. J. Heyd, E. N. Brothers, K. N.

Kudin, V. N. Staroverov, T. A. Keith, R. Kobayashi, J. Normand, K. Raghavachari, A. P. Rendell, J. C. Burant, S. S. Iyengar, J. Tomasi, M. Cossi, J. M. Millam, M. Klene, C. Adamo, R. S82 Cammi, J. W. Ochterski, R. L. Martin, K. Morokuma, O. Farkas, J. B. Foresman, D. J. Fox, *Gaussian 16 Rev. C.01*, Wallingford, CT, 2016.
[8] A .D. Becke, *J. Chem. Phys*. **1993**, 98, 5648-5652.
[9] (a) A. D. McLean, G. S. Chandler, *J. Chem. Phys.* **1980**, 72, 5639-5648. (b) K. Raghavachari, J. S. Binkley, R. Seeger, J. A. Pople, *J. Chem. Phys* **1980**, 72, 650-654.
[10] H. Kim, J. Park, J. G. Kim, S. Chang, S *Org. Lett.* **2014**, 16, 5466-5469.
[11] A. Singh, K. Teegardin, M. Kelly, K. S. Prasad, S. Krishnan, J. D. Weaver, *Journal of Organometallic Chemistry* **2015**, 776, 51-59.
[12] C. Wan, R. Song, J. Li, *Org. Lett.* **2019**, 21, 2800-2803.
[13] S. J. Plamondon, J. M. Warnica, D. Kaldre, J. L. Gleason, *Angew. Chem. Int. Ed.* 2020, **59**, 253-258.
[14] J. Waser, B. Gaspar, H. Nambu, E. M. Carreira, *J. Am. Chem. Soc.* **2006**, 128, 11693-11712.
[15] S. N. Gockel, S. Lee, B. L. Gay, K. L. Hull, *ACS Catal.* **2021**, 11, 9, 5166-5171.
[16] H. Seo, A. Liu, T. F. Jamison, *J. Am. Chem. Soc.* **2017**, 139, 13969-13972.
[17] T. Yamamoto, T. Yamakawa, *J. Org. Chem.* **2009**, 74, 3603-3605.
[18] J. Zhang, Y.mTang, *Adv. Synth. Catal.* **2016**, 358, 752-764.
[19] C. Wan, R. Song, J. Li, *Org. Lett.* **2019**, 21, 2800-2803.
[20] P. Klahn, H. Erhardt, A. Kotthaus, S. F. Kirsch, *Angew. Chem. Int. Ed.* **2014**, 53, 7913-7917.
[21] P. Rai, S. Waiba, K. Maji, D. Sahoo, B. Maji, *Org. Lett.* **2021**, 23, 8888-8893.
[22] J. Ma, S. Chen, P.,Bellotti, R. Guo, F. Schafer, A. Heusler, X. Zhang, C. Daniliuc, M. K. Brown, K. N. Houk, F. Glorius, *Science* **2021**, 371, 1338-1345.
[23] Y. Deng, G. K. H. Wiberg, A. Zana, S. Sun, M. Arenz, *ACS Catal.* **2017**, 7, 1, 433-437.
[24] J. Ma, K. Harmsa, E. Meggers, *Chem. Commun.* **2016**, 52, 10183-10186.
[25] T. Fukuyama, T. Kippo, K. Hamaoka, I. Ryu, *Sci. China Chem.* **2019**, 62, 1525-1528.
[26] C. B. Aakeröy, P. D. Chopade, *Org. Lett.* **2011**, 13, 134-137.
[27] T. Pan, X. Gao, S. Yang, L. Wang, Y. Hu, M. Liu, W. Wang, Y. Wu, B. Zheng, H. Guo, *Org. Lett.* **2021**, 23, 5750-5754.
[28] K. Ravindar, P. Caron, P. Deslongchamps, *Org. Lett.* **2013**, 15, 6270-6273.
[29] H. Shi, L. Fang, C. Tan, L. Shi, W. Zhang, C. Li, T. Luo, Z. Yang, *J. Am. Chem. Soc.* **2011**, 133, 14944-14947.
[30] Q. Zhang, J. Xie, X. Yang, J. Xie, Q. Zhou, *Org. Lett.* **2012**, 14, 6158-6161.
[31] X. Wu, H. Ji, *J. Org. Chem.* **2018**, 83, 19, 12094-12102.
[32] K. Ravindar, P. Caron, P. Deslongchamps, *J. Org. Chem.* **2014**, 79, 17, 7979-7999.
[33] L. Jin, J. Wang, G. Dong, *Angew. Chem. Int. Ed.* **2018**, 57, 12352-12355.
[34]J. Xie, S. Lin, S. Qiao, G. Li, *Org. Lett.* **2016**, 18, 3926-3929.
[35] SAINT, V8.30A, Bruker Analytical X-Ray Systems, Madison, WI, 2012.
[36] SADABS, 2.03, Bruker Analytical X-Ray Systems, Madison, WI, 2016.
[37] G. M. Sheldrick, Acta Cryst. **2008**, A64, 112 - 122. Sheldrick, G.M.. *Acta Cryst.*

2015, A71, 3.
[38] O. V. Dolomanov, L. J. Bourhis, R. J. Gildea, J. A. K. Howard, H. Puschmann, *J. Appl. Crystallogr*. **2009**, 42, 339-341.
